# Sonelokimab, an IL-17A/IL-17F-inhibiting nanobody for active psoriatic arthritis: a randomized, placebo-controlled phase 2 trial

**DOI:** 10.1038/s41591-025-03971-6

**Published:** 2025-10-06

**Authors:** Iain B. McInnes, Laura C. Coates, Philip J. Mease, Alexis Ogdie, Arthur Kavanaugh, Lihi Eder, Georg Schett, Alan Kivitz, Dennis McGonagle, Nuala Brennan, Alex Godwood, Eva Cullen, Kristian Reich, Christopher T. Ritchlin, Joseph F. Merola

**Affiliations:** 1 College of Medical Veterinary & Life Sciences (MVLS), University of Glasgow, Glasgow, UK; 2 Nuffield Department of Orthopaedics Rheumatology and Musculoskeletal Sciences, University of Oxford, Oxford, UK; 3 Providence Swedish Medical Center and University of Washington, Seattle, WA USA; 4 University of Pennsylvania, Philadelphia, PA USA; 5 University of California, San Diego, La Jolla, CA USA; 6 Department of Medicine, Women’s College Hospital and University of Toronto, Toronto, Ontario Canada; 7 Friedrich-Alexander-Universität Erlangen-Nürnberg, Erlangen, Germany; 8 Altoona Center for Clinical Research, Duncansville, PA USA; 9 Leeds Institute of Rheumatic and Musculoskeletal Medicine (LIRMM), University of Leeds, Leeds, UK; 10 MoonLake Immunotherapeutics AG, Zug, Switzerland; 11 Translational Research in Inflammatory Skin Diseases, Institute for Health Services Research in Dermatology and Nursing, University Medical Center Hamburg-Eppendorf, Hamburg, Germany; 12 Allergy, Immunology and Rheumatology Division, University of Rochester Medical School, Rochester, NY USA; 13 Department of Dermatology and Department of Medicine Division of Rheumatology, UT Southwestern Medical Center, Dallas, TX USA

**Keywords:** Inflammatory diseases, Rheumatic diseases

## Abstract

Psoriatic arthritis (PsA) is a progressive, multidomain and interleukin-17 (IL-17)-linked disease that results in substantial quality-of-life deficits. Thereby, we conducted a phase 2 randomized, double-blind, placebo (PBO)-controlled trial of sonelokimab (SLK), a nanobody that binds with a similarly high affinity to IL-17A and IL-17F, inhibiting all dimers. Overall, 207 patients with active PsA were randomized to SLK 120-mg or 60-mg every 4 weeks (Q4W; both with induction (WI)), or to 60-mg Q4W with no induction, PBO or adalimumab (reference arm). The primary endpoint of American College of Rheumatology (ACR) 50 at week 12 was met for SLK 60-mg and 120-mg WI (60-mg WI = 46.3% (19/41; odds ratio (OR) = 3.6; 95% confidence interval (CI) = 1.3–9.9; *P* < 0.05); 120-mg WI = 46.5% (20/43; OR = 4.0; 95% CI = 1.4–11.3; *P* < 0.01) versus PBO = 20.0% (8/40)). SLK resulted in significant benefits across the key secondary endpoints of ACR20 (60-mg WI = 78.0% (32/41; *P* < 0.001) and 120-mg WI = 72.1% (31/43; *P* = 0.002) versus PBO = 37.5% (15/40)) and Psoriasis Area and Severity Index (PASI) 90 at week 12 (60-mg WI = 76.9% (20/26; *P* < 0.001) and 120-mg WI = 59.3% (16/27; *P* = 0.003) versus PBO = 15.4% (4/26)). Robust responses were observed among patients randomized to SLK at week 24 for the high-threshold composite endpoints of ACR70 + PASI 100 (exploratory) and minimal disease activity (secondary), achieved by up to 48% (13/27; 120-mg WI) and 61% (25/41; 60-mg WI), respectively. SLK was well-tolerated; the most common treatment-emergent adverse events were nasopharyngitis (60 mg = 6.1%; 120 mg = 5.2%), upper respiratory tract infection (60 mg = 6.1%; 120 mg = 4.1%), injection-site erythema (60 mg = 3.7%; 120 mg = 3.1%) and headache (60 mg = 2.4%; 120 mg = 4.1%). Four cases of mild to moderate oral candidiasis occurred (60 mg = 2.4%; 120 mg = 2.1%). Overall, SLK delivered substantial improvements in the signs and symptoms of PsA across various outcomes and domains. ClinicalTrials.gov registration: NCT05640245.

## Abstract (web summary)

In the double-blind, placebo-controlled phase 2 ARGO trial, patients with active psoriatic arthritis treated with a nanobody targeting IL-17A and IL-17F showed substantially better disease response rates compared with those receiving placebo treatment.

## Main

Psoriatic arthritis (PsA) is a progressive, inflammatory and multidomain disease characterized by damage across heterogeneous tissues, including skin, articular and extra-articular manifestations^1–3^. PsA is associated with restricted mobility and function, together with a reduced quality of life and work productivity^4,5^. First-line treatment options include nonsteroidal anti-inflammatory drugs to provide relief of musculoskeletal symptoms, glucocorticoid injections and conventional disease-modifying antirheumatic drugs (DMARDs)^6,7^. For patients with intolerance to or lack of response to DMARDs, biologic agents are strongly recommended^6,7^. Despite the availability of multiple biologic treatment options, only one-third of patients achieve minimal disease activity (MDA) within 6 months of initiating biologic DMARDs (bDMARDs) or targeted synthetic DMARDs (tsDMARDs)^8–11^. PsA pathogenesis is driven by various cellular, molecular and tissue mechanisms, which contribute to heterogeneous disease phenotypes involving different tissue domains^12^. However, increasing evidence emphasizes the central importance of the interleukin-17 (IL-17) family cytokines in PsA^1,12–16^.

IL-17A and IL-17F are isoforms in the IL-17 cytokine family that are overexpressed across multiple tissues in PsA and form pro-inflammatory homodimers and heterodimers^17–22^. While IL-17A is more potent and was the initial target of therapeutic interventions in PsA, IL-17F is preferentially elevated in psoriatic tissues, suggesting that inhibiting both IL-17A and IL-17F may be critical to exerting optimal therapeutic effects. Clinically, dual blockade of IL-17A and IL-17F with the monoclonal antibody (mAb) bimekizumab has been shown to be superior to inhibiting IL-17A alone with secukinumab in plaque psoriasis^23^. While no such head-to-head studies have been performed in PsA, bimekizumab also demonstrated strong clinical efficacy in PsA, with similar levels of efficacy observed even in patients with inadequate response or intolerance to tumor necrosis factor (TNF) inhibitors^19,24^; however, even with the dual inhibition of IL-17A and IL-17F using a traditional mAb, fewer than half of patients achieved MDA in the BE-OPTIMAL study^25^. Therefore, while IL-17A and IL-17F dual blockade shows substantial promise, further optimization may be able to maximize disease control. One limitation with current biologics may be the size of the therapeutic immunoglobulin G1 antibodies (typically around 150 kDa) that block inflammatory signaling. Delivery of mAbs into even the typically well-vascularized synovial joints is subject to size-dependent restrictions, and antibody concentration in synovial fluid is substantially less than in plasma^26–28^. Although few studies have investigated antibody access across PsA tissue types, access to less vascular tissues such as the entheses may be further restricted, potentially limiting efficacy in low-bioavailability tissues^26–28^.

**Extended Data Fig. 1:**
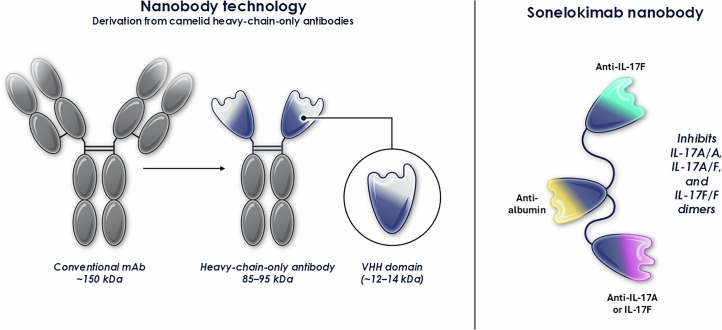
Sonelokimab is a novel nanobody that binds to both IL-17A and IL-17F with a similarly high affinity and is designed to target difficult-to-reach sites of inflammation. IL-17, interleukin-17; mAb, monoclonal antibody.

Nanobodies represent a class of antibody-derived targeted therapies that were originally developed from naturally occurring heavy-chain-only antibodies found in camelids. Heavy-chain-only antibodies have a reduced size of 85–95 kDa, owing to the absence of both light chains and the CH1 domain. The variable domain of heavy-chain-only antibodies (VHH) comprises the entire antigen-binding potential of the molecule and can be used to create standalone 12–14 kDa single-domain antibodies, known as nanobodies (Extended Data Fig. 1). Nanobodies have multiple potential advantages over traditional antibodies, including their smaller size and multi-target design potential^29–32^. In addition to the small size of nanobodies—potentially allowing greater tissue penetration compared with traditional mAbs^33^—the inclusion of an albumin-binding domain has been shown to further improve accumulation within inflamed target tissues in preclinical studies compared with mAbs or nonalbumin-binding nanobodies^33,34^. These features provide a strong rationale for the potential of nanobodies to preferentially target the heterogeneous tissues involved in PsA; however, to date, no global clinical trial results have been described for this therapy class.

Sonelokimab (SLK) is a nanobody consisting of three VHH domains that mediate binding of IL-17A, IL-17F and albumin (Extended Data Fig. 1). This design allows SLK to bind with a similarly high affinity to both IL-17A and IL-17F, blocking signaling through all IL-17A and IL-17F dimer combinations. The third albumin-binding domain increases half-life and targets albumin-rich sites of chronic inflammation and edema^35,36^. The lower predicted molecular weight of SLK (~40 kDa) relative to conventional mAbs (150 kDa) is hypothesized to enable enhanced accumulation within difficult-to-reach sites of inflammation^29,33,35^. SLK demonstrated clinical efficacy in a phase 2b study in patients with plaque psoriasis, as well as a phase 2 study in patients with hidradenitis suppurativa—a disease characterized by difficult-to-reach deep dermal sites of inflammation^35,37^.

Here we report the results of the phase 2, global, randomized, double-blind, placebo (PBO)-controlled ARGO trial that evaluated the efficacy and safety of the nanobody SLK in patients with active PsA.

## Results

### Trial design

The ARGO trial enrolled patients ≥18 years of age, with a confirmed diagnosis of PsA, active disease (defined by a 68-tender joint count (TJC68) ≥3 and a 66-swollen joint count (SJC66) ≥3)), and either currently active psoriasis or a dermatologist-confirmed history of psoriasis. Key exclusion criteria included having prior exposure to >2 biologics of any type for the treatment of PsA (for example, IL-17, IL-23 and TNF inhibitors), previous failure of IL-17 or TNF inhibitors or being unsuitable for IL-17 or TNF inhibitors. A full list of eligibility criteria can be found in Methods. The primary endpoint was the proportion of patients achieving an American College of Rheumatology (ACR) 50 response, and the key secondary endpoints were the proportion of patients achieving ACR20 and Psoriasis Area and Severity Index (PASI) 90 responses, all assessed versus PBO at week 12. Additional secondary endpoints presented included ACR20, ACR50 and PASI 90 at time points other than week 12; ACR70, PASI 75, PASI 100 and MDA; change from baseline in enthesitis (Leeds enthesitis index (LEI) and Spondyloarthritis Research Consortium of Canada (SPARCC)), dactylitis (Leeds dactylitis index (LDI)), nail disease (modified Nail Psoriasis Severity Index (mNAPSI)) and patient-reported outcomes (including Patient’s Global Assessment of Disease Activity (PGA), 12-item Psoriatic Arthritis Impact of Disease (PsAID-12), Patient’s Assessment of Arthritis Pain (PtAAP), Bath Ankylosing Spondylitis Disease Activity Index (BASDAI) and Health Assessment Questionnaire Disability Index (HAQ-DI)). The composites ACR50 + PASI 100 and ACR70 + PASI 100 were prespecified exploratory endpoints. Complete resolution of nail disease (mNAPSI = 0) was assessed post hoc. Safety endpoints were also assessed; further information on study endpoints can be found in Methods.

**Extended Data Fig. 2:**
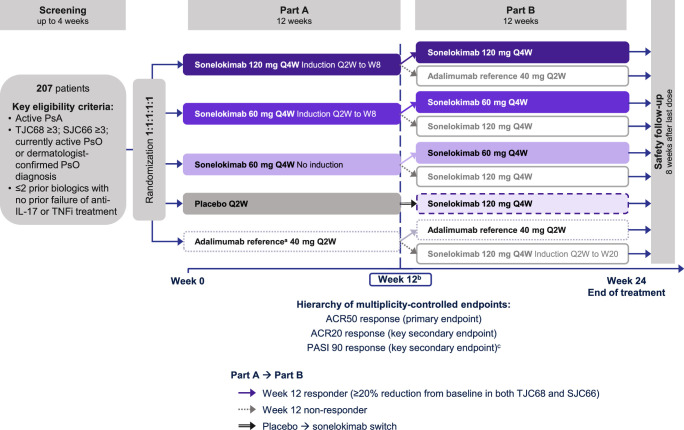
ARGO study design. Induction dosing at weeks 0, 2, 4 and 6 of treatment, followed by Q4W treatment from week 8. ^a^No formal statistical comparisons were made with the ADA ref arm. ^b^Patients in the USA completed the study at week 12. ^c^In a subgroup of patients with PsO involving ≥3% of BSA at baseline. ACR, American College of Rheumatology; ADA ref, adalimumab reference; BSA, body surface area; IL-17, interleukin-17; PASI, Psoriasis Area and Severity Index; PsA, psoriatic arthritis; PsO, psoriasis; Q2W, every 2 weeks; Q4W, every 4 weeks; SJC66, 66-swollen joint count; TJC68, 68-tender joint count; TNFi, tumor necrosis factor inhibitor; W, week.

Patients received SLK 120 mg, SLK 60 mg (both with induction (WI) doses at weeks 0, 2, 4, 6 and every 4 weeks (Q4W) from week 8 onwards), SLK 60 mg Q4W with no induction (NI), PBO or adalimumab (ADA) 40 mg every 2 weeks (Q2W) (reference (ref) arm, not powered for statistical comparisons). After a 12-week PBO-controlled period (part A), all patients receiving PBO switched to SLK 120-mg NI until the end of study at week 24 (part B). Patients with a minimal TJC68/SJC66 response (<20% improvement) at the end of part A (week 12) were re-allocated to a higher dose or treatment-arm switch (Extended Data Fig. 2).

Detailed information on the statistical analyses is presented in Methods. All efficacy analyses are presented according to the original randomization assignments. A nonresponder imputation (NRI) method was used to handle missing data within the intention-to-treat (ITT) population for the dichotomous primary and key secondary endpoints. Continuous endpoints were analyzed using a mixed model for repeated measures (MMRM) to account for missing data when estimating treatment effects and standard errors. For the part B analyses, the same NRI method as for the week 12 analyses was used, except for patients who switched treatment due to a lack of joint response or for patients in the United States who were required to complete the study at week 12. For these patients, their last observation during part A (that is, the week 12 assessment) was carried forward to all time points during part B.

### Patient disposition and baseline characteristics

**Fig. 1:**
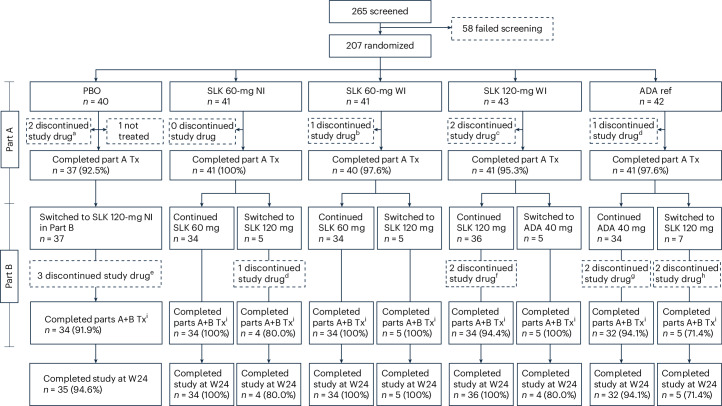
Consort diagram showing patient numbers and disposition throughout the ARGO trial. Superscript letter ‘a’ indicates patient withdrawal (*n* = 1), lack of efficacy (*n* = 1). Superscript letter ‘b’ indicates met protocol withdrawal criteria (*n* = 1). Superscript letter ‘c’ indicates AE not related to treatment (*n* = 1), patient withdrawal (*n* = 1). Superscript letter ‘d’ indicates patient withdrawal (*n* = 1). Superscript letter ‘e’ indicates AE (*n* = 3). Superscript letter ‘f’ indicates AE (*n* = 1), patient withdrawal (*n* = 1). Superscript letter ‘g’ indicates deviated from protocol (*n* = 1), patient withdrawal (*n* = 1). Superscript letter ‘h’ indicates AE (*n* = 1), lack of efficacy (*n* = 1). Superscript letter ‘i’ indicates patients in the United States completed the study at week 12. Tx, treatment; W, week.

Of 265 patients screened between 13 December 2022 and 23 May 2023, 207 were randomized to SLK 60-mg NI (*n* = 41), SLK 60-mg WI (*n* = 41), SLK 120-mg WI (*n* = 43), PBO (*n* = 40) or an ADA 40-mg Q2W ref arm (*n* = 42). All but one patient, who was randomized to the PBO group, received treatment (Fig. 1). Most patients (*n* = 187; 90.3%) completed the study treatment and discontinuation rates were low—six patients (<3%) discontinued treatment in part A and ten patients (4.8%) discontinued treatment in part B. Four patients (1.9%) were enrolled in the United States and completed the study at week 12; therefore, data after this point for these four patients were imputed using the last observation during part A (last observation carried forward).

Baseline characteristics were similar across treatment arms (Table 1). Overall, 102 (49.3%) patients were of female sex, 36 (17.4%) had previously received at least one bDMARD (including an IL-17A inhibitor (5.8%), IL-23p19i (7.7%) or TNF inhibitor (8.2%)), 9 (4.3%) patients had received two prior bDMARDs, and 65 (31.6%) and 24 (11.7%) evaluated patients had enthesitis and dactylitis, respectively. Overall, 147 (71.0%) patients were receiving concomitant methotrexate (MTX) at baseline.

**Table 1:** Baseline characteristics

Patient characteristics	PBO, *n* = 40	SLK 60-mg NI, *n* = 41	SLK 60-mg WI, *n* = 41	SLK 120-mg WI, *n* = 43	ADA ref, *n* = 42
Age (years), mean (s.d.)	47.0 (14.3)	50.3 (13.9)	47.5 (12.7)	50.2 (10.3)	47.9 (12.4)
Female sex, *n* (%)	19 (47.5)	21 (51.2)	20 (48.8)	21 (48.8)	21 (50.0)
White, *n* (%)	39 (97.5)	40 (97.6)	41 (100)	43 (100)	41 (97.6)
BMI (kg m^−^^2^), mean (s.d.)	27.9 (5.2)	29.6 (5.4)	27.7 (4.8)	30.3 (6.2)	29.3 (6.6)
Duration of PsA (years), mean (s.d.)	5.7 (6.6)^a^	6.0 (5.7)	6.2 (7.4)	4.9 (4.6)	4.1 (4.2)
**Number of prior biologics, %**					
0	85.0	80.5	82.9	81.4	83.3
1	12.5	19.5	12.2	7.0	14.3
2	2.5	0	4.9	11.6	2.4
**Prior biologic use, %**	15.0	19.5	17.1	18.6	16.7
IL-17Ai	5.1^a^	7.3	2.4	9.3	4.8
IL-23p19i	5.1^a^	9.8	7.3	9.3	7.1
TNFi	7.7^a^	2.4	12.2	11.6	7.1
**Concomitant nonbiologic DMARD at baseline, %**	75.0	82.9	68.3	72.1	73.8
Concomitant MTX at baseline, %	72.5	80.5	58.5	72.1	71.4
TJC68, mean (s.d.)	16.9 (11.6)	18.0 (12.2)	16.9 (14.1)	17.2 (12.8)	16.1 (11.6)
SJC66, mean (s.d.)	8.5 (6.0)	10.7 (8.2)	9.1 (7.1)	8.9 (5.4)	10.0 (8.5)
**Affected BSA** **≥** **3%,** ***n*** **(%)**	26 (66.7)^a^	32 (78.0)	26 (63.4)	27 (62.8)	32 (76.2)
PASI (BSA ≥ 3%), mean (s.d.)	7.1 (5.6)	6.7 (5.7)	8.0 (9.7)	7.2 (5.8)	7.3 (6.0)
**Nail psoriasis (mNAPSI > 0),** ***n*** **(%)**	21 (55.3)^b^	24 (58.5)	22 (53.7)	17 (39.5)	28 (66.7)
mNAPSI, mean (s.d.)^c^	15.2 (15.7)	16.0 (17.2)	11.5 (11.2)	14.4 (12.9)	10.5 (9.7)
**Presence of enthesitis (LEI** **>** **0),** ***n*** **(%)**	14 (35.9)^a^	14 (34.1)	16 (39.0)	11 (25.6)	10 (23.8)
LEI score, mean (s.d.)^c^	1.9 (1.3)	2.9 (1.8)	2.9 (1.9)	2.7 (1.5)	1.6 (0.7)
**Presence of dactylitis,** ***n*** **(%)**	5 (12.8)^a^	4 (9.8)	5 (12.2)	5 (11.6)	5 (11.9)
LDI score, mean (s.d.)^c^	32.7 (19.4)	238.8 (266.9)^d^	21.3 (6.4)	20.9 (13.7)	62.9 (48.7)
PsAID-12 score, mean (s.d.)	3.9 (1.7)^a^	4.3 (2.0)	4.6 (1.8)	3.9 (2.0)	4.5 (1.7)
PGA score, mean (s.d.)	60.1 (24.3)^a^	62.5 (24.0)	60.1 (20.1)	56.3 (19.2)	63.3 (20.3)
PtAAP score, mean (s.d.)	55.8 (23.6)^a^	59.8 (26.2)	60.1 (20.0)	54.8 (21.1)	58.0 (22.5)
DAPSA score, mean (s.d.)	37.9 (16.8)^a^	41.5 (20.5)	38.5 (19.6)	37.8 (16.8)	38.9 (20.8)
hs-CRP (mg l^−1^), mean (s.d.)	6.0 (6.0)^a^	6.3 (11.6)	4.7 (8.5)	5.9 (6.7)	6.5 (8.3)

BMI, body mass index; hs-CRP, high-sensitivity C-reactive protein.

^a^Calculated for *n* = 39 (excluding one patient with missing data).

^b^Calculated for *n* = 38 (excluding two patients with missing data).

^c^The mean score of patients with the presence of the symptom.

^d^One patient with baseline LDI = 602.2 (dactylitis with tenderness on seven fingers and four toes).

### Efficacy

**Fig. 2:**
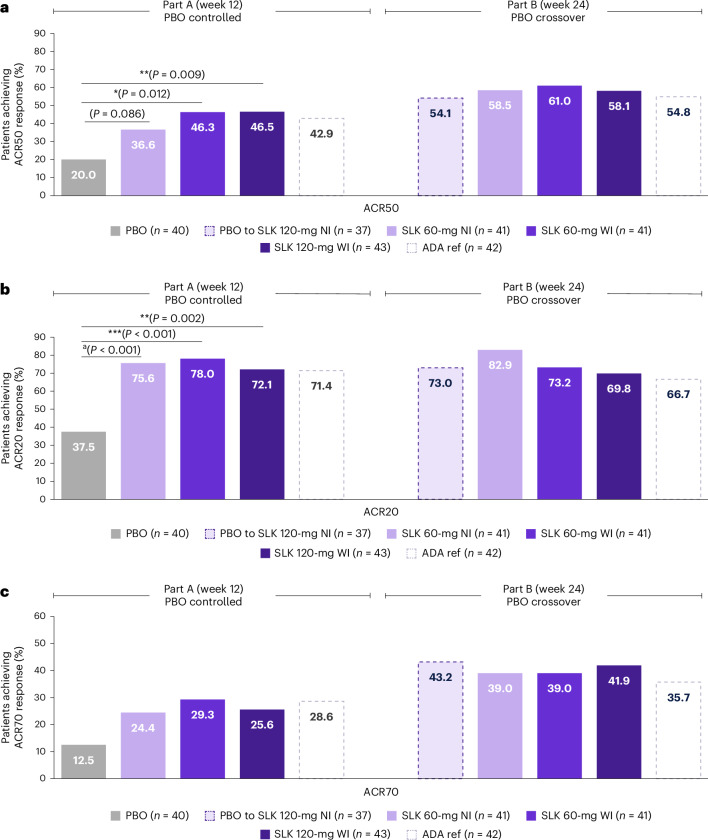
Responses on different ACR endpoint thresholds across treatment arms at week 12 and week 24. **a**, Proportion of patients achieving ACR50 response at week 12 (primary endpoint) and week 24 (ITT–NRI). **b**, Proportion of patients achieving ACR20 at week 12 (key secondary endpoint) and week 24 (ITT–NRI). **c**, Proportion of patients achieving ACR70 at week 12 and week 24 (ITT–NRI). Data are NRI, except for part B for patients who switched treatment due to the lack of joint response or for patients in the United States who completed the study at week 12, where the last observation during part A was carried forward. Percentage based on all patients in the ITT population. Statistical test results are shown for comparisons versus PBO where *P* < 0.05. *P < 0.05; **P < 0.01; ***P < 0.001 versus PBO. Two-sided *P* values were estimated using a logistic regression model including fixed effects for treatment and stratification factors (sex and prior biologic exposure). *P* values shown for SLK 120-mg WI and 60-mg WI are multiplicity controlled (**a**,**b**). ^a^A raw *P* value for SLK 60-mg NI is provided but was not statistically significant due to the nonsignificant result for the primary endpoint of ACR50 at week 12 in this group (**b**).

The ARGO trial met the primary endpoint, with a significantly greater proportion of patients treated with either SLK 120-mg or 60-mg WI achieving ACR50 compared with PBO at week 12 (SLK 120-mg WI, 46.5%, odds ratio (OR) = 4.0 (95% confidence interval (CI) = 1.4–11.3); *P* < 0.01; SLK 60-mg WI, 46.3%, OR = 3.6 (95% CI = 1.3–9.9); *P* < 0.05). For patients randomized to ADA, the ACR50 response rate at week 12 was 42.9%. A numerically higher proportion of patients treated with SLK 60-mg NI versus PBO achieved ACR50, but this difference did not reach statistical significance (36.6% versus 20.0%; *P* = 0.086; Fig. 2a and Extended Data Table 1). Statistical testing of the 60-mg NI arm therefore stopped at ACR50, in line with the sequential testing procedure used.

ACR50 response rates continued to improve through week 24 in patients receiving SLK, with 58.1–61.0% of patients randomized to SLK achieving an ACR50 response. Robust joint efficacy was also observed in patients who switched from PBO to SLK 120-mg NI at week 12, with 54.1% achieving ACR50 response at week 24, an increase of 34.1% from week 12 to week 24. For patients randomized to ADA, the ACR50 response rate at week 24 was 54.8% (Fig. 2a).

The key secondary endpoint of ACR20 at week 12 was also met, with a significantly greater proportion of patients treated with SLK 60-mg WI (78.0%, OR = 6.5 (95% CI = 2.4–18.1); *P* < 0.001) or SLK 120-mg WI (72.1%, OR = 4.7 (95% CI = 1.8–12.2); *P* < 0.01) achieving ACR20 at week 12 compared with PBO (37.5%; Extended Data Table 1). Additionally, 75.6% of patients randomized to SLK 60-mg NI (OR = 6.1 (95% CI = 2.2–16.8)) and 71.4% of patients randomized to ADA achieved ACR20 at week 12 (Fig. 2b). At week 24, ACR20 was achieved by 69.8–82.9% of patients randomized to the SLK arms and 66.7% of patients randomized to ADA. Of patients who switched from PBO to SLK 120-mg NI at week 12, 73.0% achieved an ACR20 response at week 24, representing a 35.5% increase from week 12. SLK treatment resulted in a numerically higher proportion of patients achieving the higher clinical threshold of ACR70 at week 12 compared with PBO (Fig. 2c). Notably, ACR70 response rates increased further to week 24, at which point 39.0–41.9% of patients randomized to SLK and 35.7% of patients randomized to ADA achieved this outcome. Of patients who switched from PBO to SLK 120-mg NI at week 12, 43.2% achieved an ACR70 response at week 24, an increase of 30.7% from week 12.

**Fig. 3:**
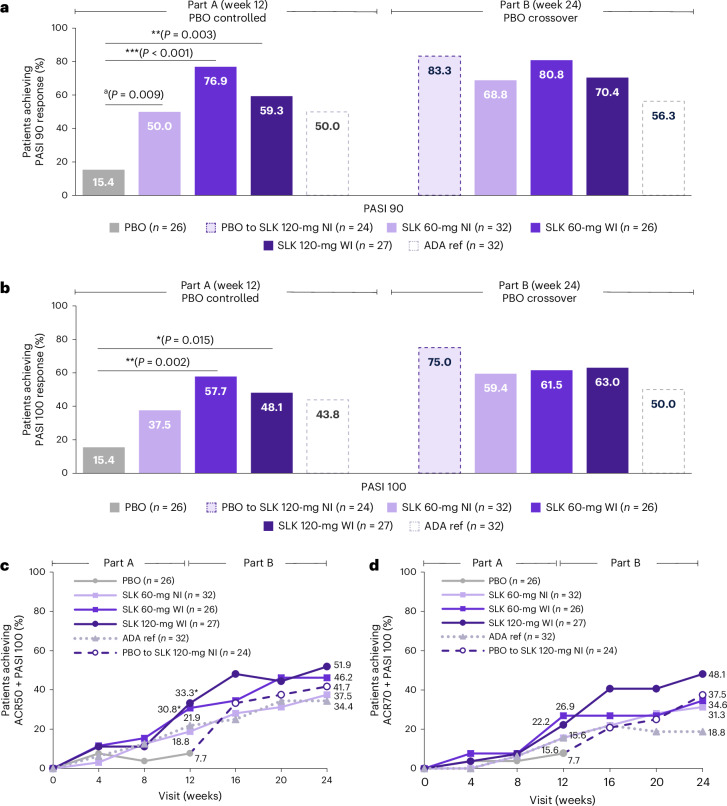
Responses on skin and concomitant skin and joint efficacy endpoints across treatment arms through week 24. **a**, Proportion of patients achieving PASI 90 response at week 12 (key secondary endpoint) and week 24 (ITT–NRI). ^a^A raw *P* value for SLK 60-mg NI is provided but was not statistically significant due to the nonsignificant result for the primary endpoint of ACR50 at week 12 in this group. *P* values shown for SLK 120-mg WI and 60-mg WI are multiplicity controlled. **b**, Proportion of patients achieving PASI 100 response at week 12 and week 24 (ITT–NRI). **c**, Proportion of patients achieving ACR50 + PASI 100 composite response through week 24 (ITT–NRI). *P* = 0.036 for SLK 120-mg WI at week 12; *P* = 0.048 for SLK 60-mg WI at week 12. **d**, Proportion of patients achieving ACR70 + PASI 100 composite response through week 24. Data are NRI, except for part B for patients who switched treatment due to the lack of joint response or for patients in the United States who completed the study at week 12, where the last observation during part A was carried forward. Percentage based on patients with ≥3% BSA at baseline in the ITT population. Statistical test results are shown for comparisons versus PBO where *P* < 0.05. *P < 0.05; **P < 0.01; ***P < 0.001 versus PBO. Two-sided *P* values were estimated using a logistic regression model including fixed effects for treatment and stratification factors (sex and prior biologic exposure) (**a**–**d**). Statistical analyses were exploratory, and *P* values are nominal (no adjustments to *P* values or significance levels were performed) (**b**–**d**).

**Extended Data Fig. 3:**
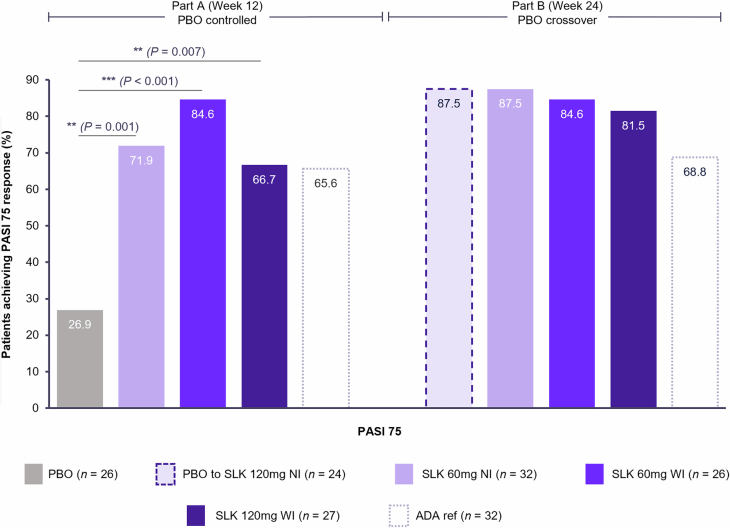
Proportion of patients achieving PASI 75 response at week 12 and week 24 (ITT-NRI). **P < 0.01; ***P < 0.001 vs. placebo. Two-sided *P*-values were estimated using a logistic regression model including fixed effects for treatment and stratification factors (sex and prior biologic exposure). Statistical analyses for these endpoints were exploratory, and *P*-values are nominal (no adjustments to *P*-values or significance levels were performed). NRI, except for part B for patients who switched treatment due to a lack of joint response or for patients in the USA who completed the study at week 12, where the last observation during part A was carried forward. Percentage based on patients with ≥3% BSA at baseline in the ITT population. ADA ref, adalimumab reference; BSA, body surface area; ITT, intention-to-treat; NI, no induction; NRI, non-responder imputation; PASI, Psoriasis Area and Severity Index; PBO, placebo; PsO, psoriasis; SLK, sonelokimab; WI, with induction.

The key secondary endpoint of PASI 90 response at week 12 was met, with a significantly greater proportion of patients treated with SLK 60-mg WI (76.9%, OR = 25.8 (95% CI = 5.4–122.8); *P* < 0.001) or 120-mg WI (59.3%, OR = 8.3 (95% CI = 2.1–33.3); *P* < 0.01) achieving PASI 90 at week 12 compared with PBO (15.4%; Extended Data Table 1). Additionally, 50.0% of patients randomized to SLK 60-mg NI (OR = 5.7 (95% CI = 1.6–20.8)) and 50.0% of patients randomized to ADA achieved PASI 90 at week 12 (Fig. 3a). SLK also demonstrated substantial benefits compared with PBO on PASI 75 (Extended Data Fig. 3) as well as at the highest clinical threshold of PASI 100, with complete skin clearance achieved by 37.5–57.7% of patients treated with SLK at week 12 (Fig. 3b). PASI responses were sustained or improved with SLK through week 24 (Fig. 3a,b), at which point 59.4–63.0% of patients randomized to SLK achieved PASI 100. Of patients who switched from PBO to SLK 120-mg NI at week 12, 75.0% achieved PASI 100 at week 24, representing a 59.6% increase from week 12. In the ADA arm, 50.0% of patients achieved PASI 100 at week 24.

**Fig. 4:**
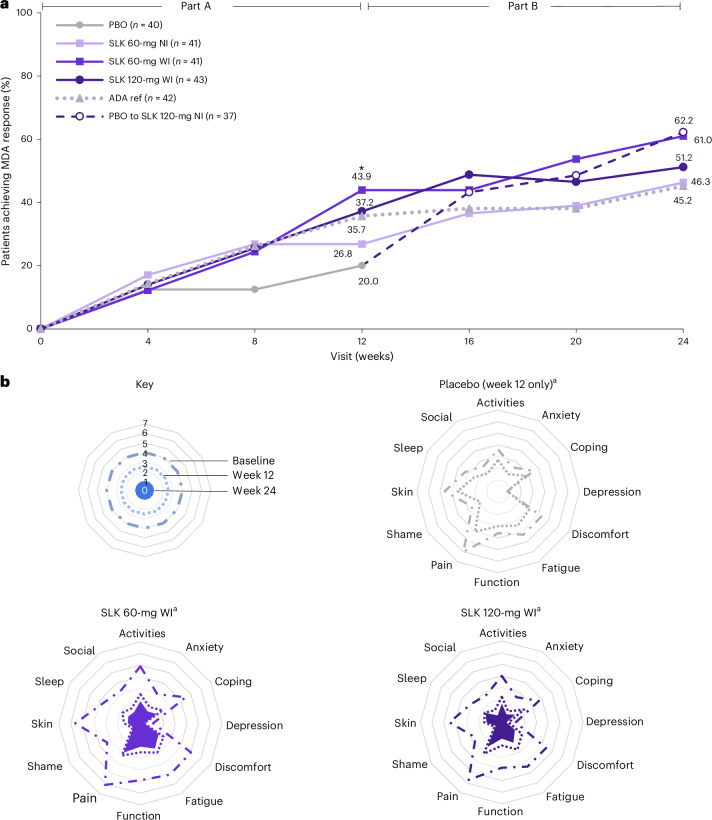
Clinical and patient-reported response across PsA domains by treatment arm. **a**, Proportion of participants achieving MDA over time through week 24 (ITT–NRI). *P < 0.05 versus PBO (*P* = 0.022 for SLK 60-mg WI at week 12). Two-sided *P* values were estimated using a logistic regression model including fixed effects for treatment and stratification factors (sex and prior biologic exposure). Statistical analyses for these endpoints were exploratory, and *P* values are nominal (no adjustments to *P* values or significance levels were performed). NRI, except for patients who switched treatment due to the lack of joint response or for patients in the United States who completed the study at week 12, where the last observation during part A was carried forward. MDA is defined as meeting at least five of the following criteria: TJC68 ≤ 1, SJC66 ≤ 1, PASI ≤ 1 or psoriasis ≤ 1% of BSA, PtAAP ≤ 15, PGA ≤ 20, HAQ-DI ≤ 0.5 and LEI ≤ 1. **b**, Mean PsAID-12 item scores at baseline, week 12 and week 24. ^a^LOCF imputation was used for patients who switched treatment due to the lack of joint response or for patients in the United States who completed the study at week 12, where the last observation during part A was carried forward. All other missing data were imputed using LOCF. Data represent mean values. LOCF, last observation carried forward.

A greater proportion of patients treated with SLK achieved MDA at week 12 compared with PBO (43.9% with SLK 60-mg WI versus 20.0% with PBO; nominal *P* = 0.022). Notably, the proportion of patients achieving MDA continued to increase to week 24, reaching 61.0% and 51.2% in the SLK 60-mg and 120-mg WI arms, 46.3% in the SLK 60-mg NI arm and 45.2% in the ADA ref arm. Of patients who switched from PBO to SLK 120-mg NI at week 12, 62.2% achieved MDA at week 24, representing a 42.2% increase from week 12 (Fig. 4a).

SLK demonstrated benefits in patient-reported outcomes, with improvements in PsAID-12 scores versus PBO at week 12 (least squares mean (LSM) change from baseline; MMRM, nominal *P* < 0.01 for all doses versus PBO; Extended Data Table 2). Improvements were sustained across multiple PsAID domains, including activities, anxiety, coping, depression, discomfort, fatigue, functional capacity, pain, shame, skin problems, sleep disturbance and social participation to week 24 (Fig. 4b). SLK treatment also resulted in improvements versus PBO across additional patient-reported outcomes, including PGA and PtAAP at week 12 (LSM change from baseline; MMRM, nominal *P* < 0.05 for all doses versus PBO). Reductions in HAQ-DI scores from baseline were observed in all arms (nominal *P* > 0.05 for LSM change from baseline at week 12 (MMRM) versus PBO); these scores continued to improve in all SLK arms (mean change from baseline at week 24 of −0.47 (−47.4%) to −0.60 (−59.1%) points; Extended Data Table 2).

### Outcomes across additional domains

SLK treatment resulted in substantial improvements in nail disease compared with PBO, as measured by LSM reductions in mNAPSI scores from baseline (nominal *P* < 0.01 for all doses versus PBO). Complete resolution of nail disease (mNAPSI = 0; post hoc assessment) at week 12 was observed for 33.3–47.1% of patients randomized to SLK, 19.0% of those treated with PBO and 46.4% of patients treated with ADA. Rates of nail clearance continued to increase, with 58.3–72.7% of patients randomized to SLK achieving complete resolution at week 24. Reductions from baseline in LEI and SPARCC enthesitis scores (Extended Data Table 3) were observed after SLK treatment; however, the small patient numbers in these groups and variability of scores at baseline limited further analysis or interpretation. Few patients had dactylitis (as per the LDI) at baseline (≤5 patients in each study arm (9.8–12.8%)), limiting assessment of this domain; however, reductions from baseline in LDI score were observed in all study arms. SLK resulted in reductions in BASDAI score, which includes measurement of axial symptoms. In patients with baseline BASDAI ≥ 4 (63.3% across all study arms), SLK resulted in meaningful changes from baseline (LSM and MMRM) with SLK 60-mg WI (−3.0 (nominal *P* = 0.072 versus PBO)), 120-mg WI (−3.0 (nominal *P* = 0.138 versus PBO)) and 60-mg NI (−3.3 (nominal *P* = 0.024 versus PBO)) at week 12 (−2.2 for PBO; Extended Data Table 3). For BASDAI Question 2, which specifically relates to neck, back and hip pain, similar reductions were observed from baseline (LSM and MMRM) with SLK 60-mg WI (−3.1 (nominal *P* = 0.429 versus PBO)), 120-mg WI (−2.9 (nominal *P* = 0.696 versus PBO)) and 60-mg NI (−3.7 (nominal *P* = 0.099 versus PBO)) at week 12 (−2.7 for PBO; Extended Data Table 3).

### Safety

SLK was well-tolerated in both part A and part B, with a safety profile consistent with that known for the inhibition of the IL-17A and IL-17F family cytokines. Any-grade treatment-emergent adverse events (TEAEs) occurred in a similar proportion of patients in each arm during the 12-week PBO-controlled part A of the study (29.3–39.5% across the SLK arms, 38.5% with PBO and 35.7% with ADA). After the full 24-week study period, the number of patients with any-grade TEAEs remained comparable across SLK dose groups (pooled patients exposed in either part A or part B), with 45.1% and 58.8% of patients experiencing TEAEs in the 60-mg (*n* = 82) and 120-mg (*n* = 97) dose groups, respectively. The number of patients with any-grade TEAEs in the ADA arm was 46.8% through week 24. The most frequently occurring TEAEs (the proportion of patients with an event) in the SLK 60-mg and 120-mg exposed groups through week 24 were nasopharyngitis (60 mg, 6.1%; 120 mg, 5.2%), upper respiratory tract infection (URTI; 60 mg, 6.1%; 120 mg, 4.1%), injection-site erythema (60 mg, 3.7%; 120 mg, 3.1%) and headache (60 mg, 2.4%; 120 mg, 4.1%). Serious TEAEs and TEAEs leading to treatment discontinuations were infrequent (Table 2), and no serious TEAEs in either the SLK 60-mg or the 120-mg dose groups were deemed to be treatment-related. Six patients (6.2%) who received SLK 120 mg (as their initial assignment or after switching in part B) discontinued due to TEAEs; three of these TEAEs were considered related to treatment (furuncle, epididymitis and tonsillar inflammation, *n* = 1 each). Overall, four cases of mild or moderate oral candidiasis were reported (60 mg, 2.4%; 120 mg, 2.1%); no cases of recurrent candidiasis or esophageal candidiasis were observed. Adverse events (AEs) of aspartate aminotransferase (AST)/alanine aminotransferase (ALT) elevations were infrequent and typically mild, with no AEs of AST or ALT elevations >3 × the upper limit of normal (ULN) occurring with SLK treatment. Additionally, no signal of liver toxicity was observed when evaluating laboratory results for AST or ALT in the study, with no elevations >5 × ULN occurring in any arm and only one laboratory finding >3 × ULN with SLK (which occurred with a nonrelated AE of exercise-induced muscle injury). There were no events of inflammatory bowel disease (IBD), major adverse cardiovascular events, depression, or suicidal ideation and behavior (SIB; Table 2).

**Table 2:** Safety through week 24

Patients with events, *n* (%)	Part A (weeks 0–12)					Parts A and B (weeks 0–24)		
	PBO, *n* = 39	SLK 60-mg NI, *n* = 41	SLK 60-mg WI, *n* = 41	SLK 120-mg WI, *n* = 43	ADA ref, *n* = 42	SLK 60 mg, *n* = 82	SLK 120 mg, *n* = 97	ADA ref, *n* = 47
Any TEAE	15 (38.5)	12 (29.3)	14 (34.1)	17 (39.5)	15 (35.7)	37 (45.1)	57 (58.8)	22 (46.8)
Any serious TEAE	0	0	1 (2.4)^a^	0	0	1 (1.2)^a^	4 (4.1)^a^	0
Any TEAE leading to treatment discontinuation	0	0	0	1 (2.3)^b^	0	0	6 (6.2)^b^	0
Fatal TEAE	0	0	0	0	0	0	0	0
**Most frequent TEAEs**^**c**^								
Nasopharyngitis	1 (2.6)	0	1 (2.4)	0	3 (7.1)	5 (6.1)	5 (5.2)	4 (8.5)
URTI	1 (2.6)	3 (7.3)	2 (4.9)	1 (2.3)	1 (2.4)	5 (6.1)	4 (4.1)	2 (4.3)
Injection-site erythema	0	0	2 (4.9)	3 (7.0)	1 (2.4)	3 (3.7)	3 (3.1)	1 (2.1)
Headache	0	0	1 (2.4)	2 (4.7)	1 (2.4)	2 (2.4)	4 (4.1)	1 (2.1)
**AEs of special interest**								
IBD	0	0	0	0	0	0	0	0
Diarrhea	0	0	1 (2.4)	0	1 (2.4)	1 (1.2)	2 (2.1)	1 (2.1)
Candidiasis	0	1 (2.4)	1 (2.4)	0	0	2 (2.4)	2 (2.1)	0
Oral candidiasis	0	1 (2.4)	1 (2.4)	0	0	2 (2.4)	2 (2.1)	0
**Other AEs of interest**								
Serious hypersensitivity	0	0	0	0	0	0	0	0
Serious infection	0	0	1 (2.4)	0	0	1 (1.2)^a^	1 (1.0)^a^	0
MACE	0	0	0	0	0	0	0	0
Liver ALT/AST >3× ULN	0	0	0	0	1 (2.4)	0	0	0
Depression (or suicidal ideation)	0	0	0	0	0	0	0	0
SIB	0	0	0	0	0	0	0	0

MACE, major adverse cardiovascular events; SAE, serious adverse event.

^a^No SAEs were judged to be treatment related.

^b^TEAEs leading to discontinuation included tonic-clonic seizure, *n* = 1 (part A); furuncle, *n* = 1 (part B); pharyngeal abscess and subcutaneous emphysema, *n* = 1 (part B); tonsillar inflammation, *n* = 1 (part B); epididymitis, *n* = 1 (part B) and arthritis, *n* = 1 (part B). The furuncle, epididymitis and tonsillar inflammation events were considered related to treatment.

^c^Top four most frequent TEAEs in the SLK groups.

### Exploratory composite disease measures

Responses on composite disease measures incorporating concomitant joint and skin outcomes were observed in a notable proportion of patients treated with SLK. The composite of ACR50 + PASI 100 was achieved by up to 51.9% of patients treated with SLK at week 24 (34.4% in the ADA arm; Fig. 3c). Additionally, up to 48.1% of patients treated with SLK achieved the higher threshold composite of ACR70 + PASI 100 at week 24 (18.8% in the ADA ref arm; Fig. 3d).

### Post hoc subgroup analyses at week 24

Clinical benefit on ACR responses at week 24 with SLK appeared to be maintained at similar levels irrespective of sex (male versus female), weight (<100 kg versus ≥100 kg) or concomitant MTX treatment (yes versus no) at baseline. Additionally, robust ACR responses were observed in patients with more severe disease at baseline (PASI ≥ 10 or Disease Activity Index for Psoriatic Arthritis (DAPSA) > 28; Extended Data Table 4).

Within the limitations of a phase 2 study, subgroup analyses revealed that clinical benefit, as indicated by PASI 90/PASI 100 responses, appeared to be maintained regardless of sex (male versus female) and baseline weight (<100 kg versus ≥100 kg). Robust levels of PASI 90/PASI 100 response were also observed in patients with more severe disease at baseline (PASI ≥ 10 and DAPSA > 28; Extended Data Table 4). Among patients with active moderate-to-severe psoriasis (PASI ≥ 10) at baseline, responses were higher in the SLK 120-mg WI dose arm, with 57.1% of patients achieving PASI 100 at week 24 compared with 33.3–42.9% of patients in the 60-mg WI or NI arms.

When considering pooled data across all SLK arms, a consistent rate of MDA was seen across key subgroups, including female patients (51.6%) and patients with a weight ≥100 kg (52.6%), prior bDMARD exposure (47.8%) and ≥3% body surface area (BSA) involvement at baseline (55.3%). Notably, 40.0% of patients across SLK arms who had active moderate-to-severe psoriasis at baseline (PASI ≥ 10) achieved MDA at week 24 (20.0% in the ADA ref arm).

## Discussion

The IL-17 cytokine superfamily has been implicated in PsA pathogenesis, with an increasing awareness of the role of IL-17F as well as IL-17A^12–16,38^. In this randomized, double-blind, PBO-controlled phase 2 trial, dual inhibition of IL-17A and IL-17F with SLK demonstrated substantial improvements in the signs and symptoms of PsA across a range of target tissues. High and sustained levels of clinical response were observed for both skin and musculoskeletal outcomes, with significant benefits compared with PBO observed for both the primary and key secondary endpoints at week 12, as well as substantial improvements on more stringent outcomes such as ACR70, PASI 100 and nail clearance (mNAPSI = 0). Robust levels of response were observed for previously defined high-hurdle composite endpoints^39^, including concomitant achievement of ACR70 + PASI 100, with consistent benefits noted across key subgroups of interest, including female patients and those not receiving concomitant MTX. Responses were also observed for endpoints encompassing multiple tissue domains and outcome measures, such as MDA, which was achieved by up to 62% of patients receiving SLK at week 24.

Given the profound impact of PsA on patients’ quality of life, patient-reported outcomes are an integral component for assessing disease impact and therapy response in PsA trials and are recognized as the most important outcomes to patients receiving treatment for PsA^40,41^. Substantial reductions in patient-reported disease impact were observed with SLK, with improvements observed across a range of symptoms and quality-of-life items. These findings support the efficacy observed with SLK on clinical endpoints and align with the high rate of MDA achievement in this study—an outcome previously linked to better health-related quality of life and productivity^42^. Improvements in nail psoriasis, enthesitis and dactylitis assessment scores were also observed during the study; however, considering the small patient numbers in these groups and variability of scores at baseline, further investigation at phase 3 with a larger patient population is required to more comprehensively define efficacy on these measures.

In this study, patients randomized to SLK treatment for the full 24-week study period achieved ACR20 rates of 69.8–82.9%, ACR50 rates of 58.1–61.0% and ACR70 rates of 39.0–41.9%. While differences in study design and varying PBO rates limit comparisons across studies, these results are encouraging when considered alongside those previously published for IL-17A-targeting biologics^43,44^. To contextualize the results observed with SLK treatment, ADA—a standard-of-care treatment for PsA^45^ that has been included in multiple previous clinical trials—was selected as the ref arm. Although the results observed with ADA in this study are favorable compared with those from pivotal studies of ADA conducted more than 20 years ago^46^, they are broadly consistent with contemporary studies that included an ADA active ref arm. For example, 42.9% of patients randomized to ADA in this study achieved the primary endpoint of ACR50 at week 12, consistent with results from SELECT-PsA 1 (37.5% at week 12)^47^ and BE-OPTIMAL (45.7% at week 16)^25^. In ARGO, the proportion of patients achieving ACR20, ACR50 and ACR70 responses (as well as skin and composite outcome responses) was consistently numerically higher in the SLK arms than in the ADA ref arm. Although this ref arm was not powered for comparison, these findings remain encouraging given that few clinical studies in PsA, including those of anti-IL-17 antibodies, have demonstrated superior and/or numerically higher results than ADA on joint-focused outcomes.

Larger phase 3 studies will be required to define more precisely the level of clinical response achieved with SLK. If confirmed, the response rates observed in this study could be in part explained by blocking both IL-17A and IL-17F. Preferential elevation of IL-17F compared with IL-17A has been described in psoriatic tissue^48^, and existing evidence suggests that IL-17A neutralization alone may not be sufficient for full disease control in PsA^21,48^. This theoretical advantage of dual IL-17A and IL-17F inhibition is consistent with a network meta-analysis of clinical trial data, which demonstrated superior outcomes with bimekizumab compared with inhibiting IL-17A alone, particularly in patients with an inadequate response or prior intolerance to TNF inhibitors^49^. Beyond dual IL-17A and IL-17F targeting, the inherent structural differences of nanobodies compared with traditional mAbs may also confer additional advantages. SLK uses a unique design that allows binding to both IL-17A and IL-17F with a similarly high affinity, a property not always possible with mAbs that rely on a single cross-reactive site to inhibit multiple proteins^50^. Additionally, the considerably smaller size and albumin-binding domain of SLK may facilitate enhanced accumulation within difficult-to-reach sites of inflammation. For example, an exponential relationship between molecular weight and tissue biodistribution values has been described for antibody fragments, whereby a 35-kDa increase in molecular weight leads to a 50% reduction in the biodistribution coefficient^33^. Similarly, the incorporation of an albumin-binding domain has been shown to enhance nanobody targeting to inflamed joints in a mouse model of collagen-induced arthritis^51^. Therefore, the unique structural design of SLK, compared with traditional mAbs, may also contribute to the high response rates observed in this study.

Previous studies revealed occurrences of IBD, infections, liver enzyme elevations and suicide-related and depression-related events with IL-17 inhibition^18,44,52–54^. In this study, there were no cases of IBD, depression, SIB or AEs of elevated liver AST/ALT >3× ULN in patients treated with SLK. Animal model data have suggested mechanistic reasons for differential AE profiles when IL-17F, as well as IL-17A, is inhibited^55–57^; however, this has not always been consistent with clinical findings, and further data from phase 3 studies will be required to determine the incidence of these AEs in larger patient populations. Rates of infection were limited with SLK 60 mg and 120 mg through week 24 (nasopharyngitis = SLK 60 mg 6.1% (*n* = 5), SLK 120 mg 5.2% (*n* = 5); URTI = SLK 60 mg 6.1% (*n* = 5), SLK 120 mg 4.1% (*n* = 4)). IL-17 has a role in host immunity against extracellular bacteria and fungi, and clinical IL-17 inhibition has been strongly associated with an increased risk of candidiasis in prior studies^58,59^. Oral candidiasis occurred in 2.1–2.4% of patients treated with SLK in this study; all cases were mild or moderate in severity and were not recurrent. Overall, SLK was well-tolerated, with those AEs observed consistent with the inhibition of IL-17 family cytokines and previously reported clinical trials of SLK in plaque psoriasis and hidradenitis suppurativa^35,37,60–62^.

ARGO was the first study in PsA to stratify patients by sex, an increasingly recognized factor that has been shown to affect treatment response^63^, with ACR response rates commonly 10–20% lower in female versus male patients in contemporary bDMARD studies^64,65^. It is therefore notable that the efficacy of SLK was largely comparable across key subgroups of clinical interest, with female patients achieving key clinical outcomes (ACR50, ACR70, PASI 90 and PASI 100) at similar or numerically higher rates than male patients. High rates of PASI 100 were also observed in patients with active moderate-to-severe psoriasis (PASI ≥ 10; *n* = 36), with responses in this population most common with the SLK 120-mg WI dose (57.1% complete skin clearance at week 24)—a finding consistent with a phase 2b study of SLK in moderate-to-severe psoriasis^35^.

Responses were similar across doses when considering all endpoints. As expected, induction dosing led to quicker responses, with noninduction dosing not meeting the primary endpoint of ACR50 at week 12; however, similar overall response rates were observed in the noninduction dosing group by week 24. Given that no single SLK dose regimen was clearly superior to the other SLK dose regimens tested, all three doses will be further evaluated in phase 3 studies.

Although this was a comparatively large and well-controlled phase 2 study, the relatively small number of patients included in each arm remains a limitation when evaluating efficacy across subgroups and in disease domains such as dactylitis and enthesitis, which were only present in a smaller subgroup of patients at baseline. Therefore, although numerical improvements were observed for dactylitis and enthesitis, no statistically significant impact of SLK treatment could be shown on these disease domains. However, this study provided strong initial evidence of robust multidomain efficacy with SLK, and phase 3 studies in larger patient populations are ongoing to more comprehensively assess efficacy across all disease domains (IZAR-1 (NCT06641076) and IZAR-2 (NCT06641089)). It is known that axial inflammation can affect up to 40% of patients with PsA^66^. While this study did not include clinical assessment of axial inflammation or collect information on clinical diagnosis of axial PsA, the impact of SLK on axial symptom activity was assessed using the BASDAI questionnaire. Although preliminary evidence of reduced axial symptoms was observed with SLK, definitive conclusions on axial efficacy with SLK cannot be drawn in this phase 2 PsA population. Finally, while the study population is typical of other phase 2 PsA studies, the population may not be completely applicable to real-world clinical practice.

In conclusion, this phase 2 study demonstrated that SLK yielded robust ACR50 responses compared with PBO at week 12. Highly encouraging response rates were also observed for higher threshold outcomes, such as ACR70 and PASI 100, as well as for multidomain measures of efficacy across different disease domains. SLK was well-tolerated, with a safety profile consistent with the inhibition of the IL-17 cytokine family, as observed in previous studies in plaque psoriasis and hidradenitis suppurativa. The overall study results warrant further exploration of SLK in two ongoing phase 3 trials, the IZAR-1 study (NCT06641076) in patients with biologic-naive PsA, and the IZAR-2 study (NCT06641089) in patients with PsA and prior inadequate response or intolerance to biologic TNF inhibitors, to establish the potential of SLK as a future treatment for PsA.

## Methods

### Study design and patient eligibility

ARGO (NCT05640245) was a global, randomized, prospective, parallel-group, double-blind, PBO-controlled trial conducted at 42 sites in Bulgaria, Czechia, Estonia, Germany, Hungary, Poland, Spain and the United States. This study comprised part A (weeks 0–12), part B (weeks 12–24) and a safety follow-up period (weeks 24–32; Extended Data Fig. 2).

Eligible patients were ≥18 years of age, with a confirmed diagnosis of PsA per 2006 Classification Criteria for Psoriatic Arthritis with symptoms ≥6 months before the screening visit, active disease (defined by TJC68 of ≥3 and SJC66 of ≥3), and either currently active psoriasis or a dermatologist-confirmed history of psoriasis. Patients must have been negative for rheumatoid factor and anticyclic citrullinated peptide antibodies at the screening visit and be suitable for treatment with ADA (in the opinion of the investigator) per approved local product information. If a chest X-ray or CT scan for tuberculosis (TB) screening was required per local guidance, this must have been carried out within 3 months before the screening visit. Patients were required to have had an inadequate response (defined as a lack of efficacy after a ≥12-week duration of therapy) to previous or current treatment with ≥1 nonbiologic DMARD (at the maximum tolerated dose), or intolerance or contraindication for DMARDs as defined by the investigator. Patients must have been of nonchildbearing potential or, if of childbearing potential, must have agreed to use highly effective methods of contraception; women of childbearing potential must have had a negative serum human chorionic gonadotropin pregnancy test at the screening visit and a negative urine pregnancy test at week 0/day 1 before the first administration of study treatment; male patients must have been willing to use a condom when sexually active with a partner of childbearing potential during the study and for 12 weeks after the last dose of study treatment, unless surgically sterile. Included patients had to be considered reliable and capable of adhering to the protocol, visit schedule or medication intake according to the judgment of the investigator, and were able to understand and provide signed informed consent.

Exclusion criteria included having prior exposure to >2 biologics of any type (for example, IL-17, IL-23 and TNF inhibitors); previous failure of IL-17 or TNF inhibitor therapy (defined as inadequate clinical response according to the investigator’s judgment after at least 16 weeks of treatment), or considered unsuitable for IL-17 or TNF inhibitor therapy for any other reason according to the investigator’s discretion; or had a diagnosis of chronic inflammatory conditions other than psoriasis or PsA, or a diagnosis of arthritis mutilans. Patients must not have had known hypersensitivity to SLK or ADA, or any of their excipients, or used or planned to use one or more prohibited treatment specified in the protocol. Patients were excluded if they had an active infection or history of infections, including any of the following: any infection (exception of common cold) requiring systemic treatment within 14 days before initiation of study treatment; serious infection (defined as infection requiring hospitalization or intravenous anti-infective) within 2 months before initiation of study treatment; history of opportunistic infections caused by uncommon pathogens or severe infections caused by common pathogens; history of other opportunistic, recurrent or chronic infections that (in the opinion of the investigator) might cause study participation to be detrimental to the patient; *Candida* infection requiring systemic therapy for ≥7 days in the last 12 months before study treatment initiation; any history of esophageal or systemic candidiasis; current active candidiasis or *Candida* infection within the last 1 month before the screening visit; concurrent acute or chronic viral hepatitis B or C or HIV; or confirmed severe acute respiratory syndrome coronavirus 2 infection at the screening visit.

Patients must not have received a live (including attenuated) vaccination within 8 weeks before study treatment initiation, or planned to receive a live vaccination during the study up to at least 12 weeks after the last dose of study treatment; or received a Bacillus Calmette–Guérin vaccination within 1 year before study treatment initiation. Patients with a history of active TB (that is, those having received combination treatment for active TB), or evidence of TB infection as defined by a positive QuantiFERON TB-Gold test (or interferon-γ release assay equivalent) at screening, were excluded, unless the following criteria applied: a full TB work-up (according to local practice/guidelines) completed within 12 weeks before randomization established that there was no evidence of active or latent TB; or patients positive for latent TB per work-up completed sufficient treatment according to local routine clinical practice at least 4 weeks before randomization. Patients with any current nontuberculous mycobacterial infection or any history of pulmonary nontuberculous mycobacterial infection at the screening visit, or evidence of acute ocular inflammation, including active anterior uveitis, within the last 4 weeks before study treatment initiation were excluded. Patients with concurrent malignancy or history of malignancy during the past 5 years before the screening visit were excluded, with the following exceptions: ≤3 excised or ablated basal cell carcinomas of the skin; one squamous cell carcinoma of the skin not worse than stage T1 that has been successfully excised or ablated (no other previous treatments allowed), with no signs of recurrence or metastases for at least the past 2 years before study treatment initiation; actinic keratosis; squamous cell carcinoma in situ of the skin successfully excised or ablated >6 months before study treatment initiation; and localized carcinoma in situ of the cervix, treated and considered cured. Patients must not have had fibromyalgia, osteoarthritis symptoms or any other condition that, in the investigator’s opinion, may have potentially interfered with efficacy assessments; erythrodermic, guttate or pustular form of psoriasis or drug-induced psoriasis; a history of a lymphoproliferative disorder, including lymphoma, or current signs and symptoms suggestive of lymphoproliferative disease; primary immunodeficiencies, prior splenectomy or suppressive conditions, including patients taking immunosuppressive therapy after organ transplants; major surgery (including joint surgery) within 6 months before the screening visit or were planning to have major surgery during the study; severe cardiovascular comorbidities, including history of myocardial infarction, stroke, unstable angina pectoris, heart failure (New York Heart Association classification III or IV) or uncontrolled hypertension (characterized by two blood pressure (BP) measurements separated by at least 15 min with systolic BP of >160 mm Hg or diastolic BP of >100 mm Hg); clinically significant electrocardiogram (ECG) abnormalities or centrally read ECG at the screening visit; other clinically significant medical conditions or any other reason, including any physical, psychologic or psychiatric condition that, in the opinion of the investigator, would compromise the safety or interfere with participation in the study, would make the patient an unsuitable candidate to receive study treatment or would put the patient at risk. Patients with a presence of active suicidal ideation or positive suicidal behavior at the screening visit, as evidenced by the Columbia-Suicide Severity Rating Scale (C-SSRS) assessment that showed any history of suicidal attempt, or suicidal ideation in the past 6 months as indicated by a positive response to either question 4 or 5 on the C-SSRS at screening; or of moderately severe depression or severe depression, indicated by a score ≥15 using the screening Patient Health Questionnaire-9, were excluded. Patients were allowed to use one medication to treat depression provided the dose was stable for 4 weeks before initiation of study treatment, but patients with multiple medications for depression were excluded. Patients with laboratory abnormalities at the screening visit were excluded, including AST, ALT or alkaline phosphatase of >3× ULN, serum direct bilirubin of >1.5× ULN (in the absence of known Gilbert’s syndrome), white blood cell count of <3.0 × 10^9^ l^−1^, absolute neutrophil count of <1.5 × 10^9^ l^−1^, absolute lymphocyte count of <0.8 × 10^9^ l^−1^, platelet count of <100 × 10^9^ l^−1^, hemoglobin of <85 g l^−1^, creatinine clearance of <60 ml min^−1^ (by Cockcroft–Gault formula) or any other laboratory abnormality which, in the opinion of the investigator, might have compromised the patient’s safety, prevented them from completing the study or interfered with the interpretation of the study results. Patients must not have been enrolled in another interventional investigational device or drug study, or been on another investigational study treatment, in the last 28 days before the screening visit or within five half-lives of the other investigational device or study treatment before the screening visit (whichever is greater); must not have a history of chronic alcohol or drug abuse in the past year before the screening visit; must not have been pregnant or breastfeeding, or planned to become pregnant, while enrolled in the study and up to 12 weeks after the last dose of study treatment; and must not have been an employee, or direct relative of an employee, of the sponsor at a study site, or of a third-party organization involved in the study.

The study was conducted in accordance with the Declaration of Helsinki. All patients provided written informed consent. The study protocol, amendments and all recruitment materials were reviewed and approved before the study was conducted by an institutional review board/independent ethics committee. The institutional review board/independent ethics committee were as follows: Ethics Committee for Clinical Trials under the Minister of Health (Bulgaria), EC St. Anne’s University Hospital in Brno and the Ethics Committee of the Institute for Clinical and Experimental Medicine and the Thomayer University Hospital (Czechia), Ethics Committee for Medicinal Products (Estonia), Ethics Committee of the Medical Faculty (Germany), Ethics Committee for Clinical Pharmacology (Hungary), Bioethics Committee at the Wielkopolska Medical Chamber in Poznań (Poland), Medicinal Research Ethics Committee (CEIm Parc Tauli; Spain) and Advarra (the United States).

### Randomization and masking

Patients were randomized 1:1:1:1:1 to SLK 120-mg WI, SLK 60-mg WI, SLK 60-mg NI, PBO or ADA 40-mg Q2W (active ref arm). Randomization was stratified by sex (male/female) and prior exposure to biologic agents (yes/no). Web-based interactive response technology was used to assign patients to treatment arms following a predetermined, computer-generated randomization scheme (with a randomization block size of 10) that was approved by the sponsor’s biostatistician. The total number of patients with previous exposure to biologic agents was capped at 30%. To maintain study blinding, the prefilled syringes for SLK and PBO were identical in appearance. The ADA injector was different in appearance from SLK and PBO; however, the study was blinded at the carton level. Patients were also asked to wear an eye mask for all injections. All study personnel were blinded until week 12.

### Study procedures

The study included a screening period of up to 28 days, followed by a 24-week treatment period (part A and part B) and a safety follow-up period of 8 weeks after the last dose of study treatment (Extended Data Fig. 2). Part A ended at week 12 when the primary efficacy analysis was performed, comparing each of the SLK arms with PBO. Patients in the United States completed the study at week 12. Treatment allocation in part B (week 12) was based on whether patients showed response in part A. A responder was defined as a patient who achieved ≥20% reduction in each of the TJC68 and SJC66 assessments at week 12 compared with baseline. A nonresponder was defined as a patient who did not achieve ≥20% reduction in each of the TJC68 and SJC66 assessments at week 12 compared with baseline. SLK and PBO were administered as subcutaneous injections. In the SLK 120-mg arm, patients received SLK 120-mg Q2W until week 8 (induction dosing, previously shown to be effective in patients with psoriasis^35^) and then Q4W thereafter; patients who did not meet the response requirements at week 12 switched to ADA 40-mg Q2W through week 24, and those who met the response criteria continued SLK 120-mg Q4W through week 24. In the SLK 60-mg arm, patients received SLK 60-mg Q2W until week 8 (induction dosing) and then Q4W thereafter; patients who did not meet the response requirements at week 12 switched to SLK 120-mg Q4W through week 24, and those who met the response criteria continued SLK 60-mg Q4W through week 24. In the SLK 60-mg NI arm, patients received SLK 60-mg Q4W (NI, included to further characterize alternative dosing schedules); patients who did not meet the response requirements at week 12 switched to SLK 120-mg Q4W through week 24, and those who met the response criteria continued SLK 60-mg Q4W through week 24. Patients on active treatment received PBO when relevant to maintain a blinded Q2W dosing schedule. In the ADA arm, patients received a dose of 40-mg Q2W in accordance with the approved label; patients who did not meet the response requirements at week 12 switched to SLK 120-mg WI Q2W until week 20, and then Q4W thereafter, those who met the response criteria continued ADA 40-mg Q2W through week 24. In the PBO arm, patients received PBO Q2W; all patients then switched to SLK 120-mg Q4W (NI) during weeks 12–24.

### Efficacy assessments

ACR20/ACR50/ACR70 response was defined as ≥20%/50%/70% improvement in TJC68 and in SJC66, and ≥20%/50%/70% improvement in ≥3/5 additional variables (PGA, Physician’s Global Assessment of Disease Activity, PtAAP, HAQ-DI and high-sensitivity C-reactive protein) and was measured using the standard ACR response criteria^67^. The degree of a patient’s psoriasis was measured using the standard PASI score^68^.

The LDI was used to measure the ratio of the circumference of an affected digit to the circumference of the digit on the opposite hand or foot, with a minimum difference of 10% defining a dactylitic digit. If dactylitis was present in any finger or toe, the patient was considered to have dactylitis. Enthesitis count was carried out by an investigator or qualified individual, in which tenderness upon examination was recorded as either present or absent per site. The enthesitis count was then used to derive the SPARCC and LEI. If enthesitis was present in any of the six sites included in the LEI, the patient was counted as having enthesitis for the purpose of calculating enthesitis responders.

The mNAPSI tool was used to assess nail disease involvement and scored on a scale of 0–130 for all fingernails in patients with PsA with evidence of nail involvement, and it was assessed by a qualified individual. MDA was defined as meeting five of the following seven criteria: TJC68 ≤ 1, SJC66 ≤ 1, PASI ≤ 1 or psoriasis affecting ≤1% of BSA, PtAAP ≤15 on a 0–100 visual analog scale (VAS), PGA ≤ 20 on a 0–100 VAS, HAQ-DI ≤ 0.5 and LEI ≤ 1.

### Patient-reported outcomes

The PsAID-12 (ref. ^69^) was self-administered and included 12 health domains related to PsA (pain, fatigue, skin problems, work and/or leisure activities, functional capacity, discomfort, sleep disturbance, coping, anxiety, embarrassment and/or shame, social participation and depression).

The PtAAP and PGA were self-administered and performed using a VAS. For PtAAP, the VAS ranged from 0 (no pain) to 100 (severe pain), which patients chose in response to the question ‘Please indicate the most pain you had from your PsA today’. For PGA, the VAS ranged from 0 (very well) to 100 (very poor), which patients selected to answer the question ‘Considering all the ways PsA affects you, please indicate how well you are doing today’.

The HAQ-DI was self-administered and included 20 items that assessed current physical function/disability across eight categories (dressing and grooming, hygiene, arising, reach, eating, grip, walking and common daily activities). HAQ-DI scores ranged from 0 (no difficulty) to 3 (unable to do).

The BASDAI was self-administered and consisted of six patient numerical rating scales (NRS) to measure severity of fatigue, axial involvement, peripheral articular involvement, localized tenderness/enthesopathy, severity of morning stiffness and time of morning stiffness. Each NRS ranged from 0 (none) to 10 (very severe). The composite BASDAI score was the mean of the six individual NRS scores.

### Safety assessments

Safety was assessed throughout the study, and the incidence, severity and seriousness of AEs were determined according to Common Terminology Criteria for Adverse Events Version 5.0. TEAEs were defined as any AE occurring or worsening on or after the first dose of study treatment. AEs of special interest were defined for the purposes of this study as *Candida* infections, IBD (including Crohn’s disease or ulcerative colitis) and diarrhea.

### Study outcomes

The primary endpoint of the study was the proportion of patients achieving ACR50 at week 12 compared with PBO. The key secondary endpoints were the proportion of patients achieving ACR20 at week 12 compared with PBO, and the proportion of patients achieving PASI 90 at week 12 compared with PBO in the subgroup of patients with psoriasis involving ≥3% BSA at baseline.

Additional secondary endpoints presented included ACR20, ACR50 and PASI 90 at time points other than week 12; ACR70, PASI 75, PASI 100 and MDA; change from baseline in enthesitis (LEI and SPARCC), dactylitis (LDI), nail disease (mNAPSI) and patient-reported outcomes (including PGA, PsAID-12, PtAAP, BASDAI and HAQ-DI). The composites ACR50 + PASI 100 and ACR70 + PASI 100 were prespecified (exploratory) endpoints added to the statistical analysis plan after the protocol was finalized. Complete resolution of nail disease (mNAPSI = 0) was assessed post hoc. Safety endpoints were also assessed. A full list of endpoints is provided in the accompanying protocol and statistical analysis plan, available in Supplementary Note.

### Statistical analysis

Sample size was calculated based on the assumed ACR50 response rates of 40% with each SLK dose regimen and 10% with PBO at week 12. Based on these assumptions, a sample size of 40 patients in each of the SLK arms and in the PBO arm resulted in a power of more than 80%, using an overall two-sided *α* of 0.025. The ADA arm was used as an active ref arm to validate clinical findings in ARGO. The active ref arm was not powered for any statistical comparisons with SLK or PBO and was not included in the testing hierarchy.

The efficacy analyses were based on the full analysis set, comprising all randomized patients (ITT population). The safety analysis set included all randomized patients who received at least one dose of the study treatment.

For the primary endpoint and key secondary endpoints, a logistic regression model was used to test the pairwise comparisons (PBO and either SLK 120-mg WI, SLK 60-mg WI or SLK 60-mg NI), including fixed effects for treatment and the stratification factors (sex and prior biologic use) before screening. The ORs, risk difference, 95% CIs and estimated two-sided *P* values were derived from the logistic regression model.

To adjust for multiple testing and control the family-wise error rate in ARGO, the primary endpoint (ACR50 at week 12) and key secondary endpoints (ACR20 and PASI 90 at week 12) were tested using a fixed-sequence hierarchical order. Once a null hypothesis within this hierarchy is not rejected, no further confirmatory statistical testing can be conducted. Additionally, given the multiple dose arms included in ARGO, the Bonferroni–Holm procedure was applied to control for testing both induction arms (120 mg and 60 mg) across the same endpoint. In this procedure, the induction arm with the smallest *P* value was compared to a significance level of 0.025. If significant, the arm with the largest *P* value was then compared to a significance level of 0.05. If the procedure passed all key secondary endpoints for both SLK 120-mg WI and SLK 60-mg WI, then SLK 60-mg NI was compared with the *α* level of 0.05 for the primary endpoint and then sequentially through the key secondary endpoints.

For other secondary endpoints, no confirmatory statistical testing was performed, but statistical tests were used for exploratory purposes only. Continuous endpoints were summarized descriptively and analyzed using an MMRM including treatment arm, visit, sex, prior exposure to biologic agents and treatment by visit interaction as fixed effects. Using this model, adjusted LSM in each arm, LSM difference between arms, 95% CIs and two-sided *P* values were estimated. Dichotomous endpoints were also summarized descriptively and analyzed using logistic regression.

An NRI method was used to handle missing data for the dichotomous primary and key secondary endpoints, whereby patients were considered nonresponders if they discontinued the study before week 12 due to any reason, had a missing score at baseline or week 12 due to any reason or took prohibited medication before week 12. Continuous endpoints were analyzed using an MMRM to account for missing data when estimating treatment effects and standard errors.

For the part B analysis, no confirmatory statistical testing was performed, and all efficacy endpoints were considered secondary. Descriptive statistics were presented by visit from weeks 0 to 24 and repeated by visit from weeks 12 to 24. For dichotomous endpoints, an NRI method was used, except for patients who switched treatment due to the lack of joint response or for patients in the United States who completed the study at week 12, for whom the last observation during part A was carried forward to all time points during part B.

Safety analyses were performed separately for part A and part B. AEs were assigned to either part A or part B based on the event start date and time. AEs were summarized by treatment arm using descriptive statistics and coded according to the Medical Dictionary for Regulatory Activities. Incidence rates of TEAEs during part B and for the combined analysis of part A and part B were adjusted for exposure duration to the study treatment.

### Reporting summary

Further information on research design is available in the Nature Portfolio Reporting Summary linked to this article.

## Online content

Any methods, additional references, Nature Portfolio reporting summaries, source data, extended data, supplementary information, acknowledgements, peer review information; details of author contributions and competing interests; and statements of data and code availability are available at 10.1038/s41591-025-03971-6.

## Supplementary information

Supplementary Information Supplementary Note (Protocol and Statistical Analysis Plan (SAP)).

Reporting Summary

## Figures and Tables

**Extended Data Table 1:**
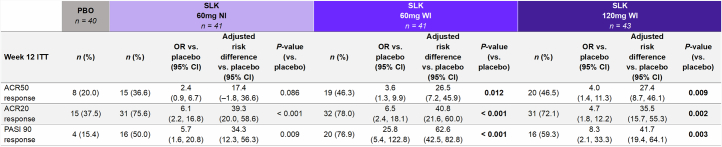
Primary and key secondary endpoints at week 12

Two-sided *P*-values were estimated using a logistic regression model including fixed effects for treatment and stratification factors (sex and prior biologic exposure). *P*-values shown for sonelokimab 120 mg WI and 60 mg WI are multiplicity-controlled. Raw *P*-values for sonelokimab 60 mg NI are provided but were not statistically significant due to the non-significant result for the primary endpoint of ACR50 at week 12 in this group. For ACR, percentages were based on all patients in the ITT population. For PASI, percentage based on patients with ≥3% BSA at baseline in the ITT population.

ACR, American College of Rheumatology; BSA, body surface area; CI, confidence interval; ITT, intention-to-treat; NI, no induction; OR, odds ratio; PASI, Psoriasis Area and Severity Index; PBO, placebo; SLK, sonelokimab; WI, with induction.

**Extended Data Table 2:**
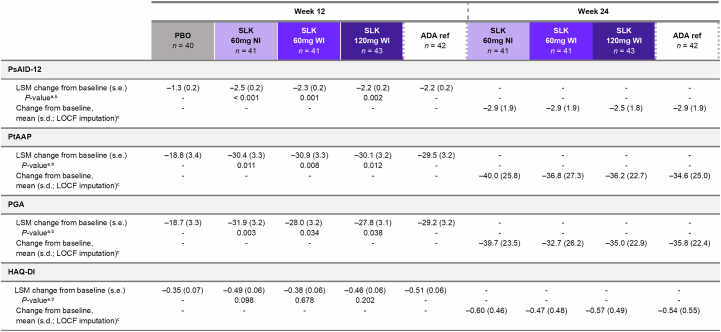
Additional patient-reported outcomes at week 12 and week 24

^a^Two-sided *P*-values were estimated using a MMRM analysis including treatment arm, baseline, visit, sex, prior exposure to biologic agents, and treatment by visit interaction as fixed effects and patient as random effect. Statistical analyses for these endpoints were exploratory.

^b^*P*-values are nominal (no adjustments to *P*-values or significance levels were performed).

^c^LOCF imputation was used to account for potential treatment switching in part B. LOCF was applied for patients who switched treatment due to a lack of joint response or for patients in the USA who completed the study at week 12 where the last observation during part A was carried forward. All other missing data were imputed using LOCF.

ADA ref, adalimumab reference; HAQ-DI, Health Assessment Questionnaire Disability Index; LOCF, last observation carried forward; LSM, least squares mean; MMRM, mixed model for repeated measures; NI, no induction; PBO, placebo; PGA, Patient’s Global Assessment of Disease Activity; PsAID-12, 12-item Psoriatic Arthritis Impact of Disease; PtAAP, Patient’s Assessment of Arthritis Pain; s.d., standard deviation; s.e., standard error; SLK, sonelokimab; WI, with induction.

**Extended Data Table 3:**
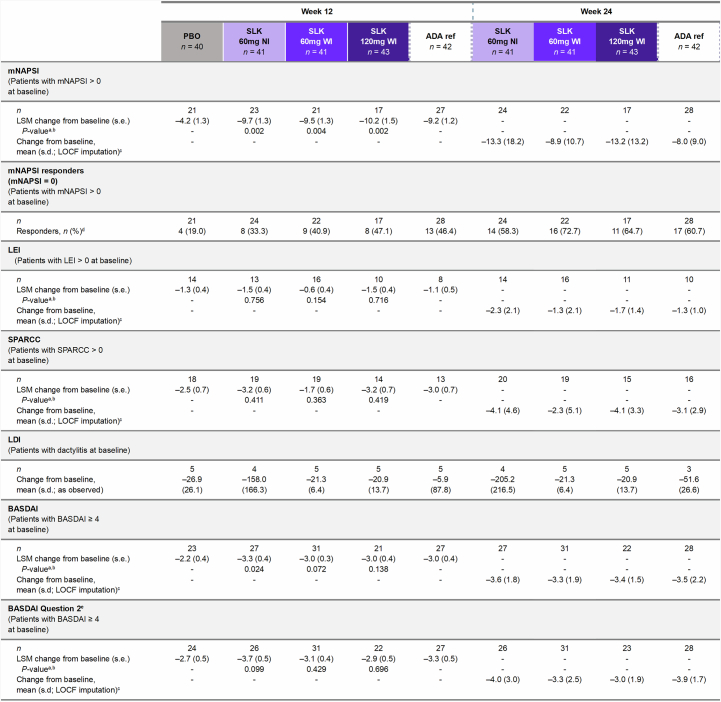
Additional outcomes at week 12 and week 24

^a^Two-sided *P*-values were estimated using a MMRM analysis including treatment arm, baseline, visit, sex, prior exposure to biologic agents, and treatment by visit interaction as fixed effects and patient as random effect. Statistical analyses for these endpoints were exploratory.

^b^*P*-values are nominal (no adjustments to *P*-values or significance levels were performed).

^c^LOCF imputation used for patients who switched treatment due to a lack of joint response or for patients in the USA who completed the study at week 12 where the last observation during part A was carried forward. All other missing data were imputed using LOCF.

^d^LOCF from week 12 to 24 has been applied to patients who either switched treatment arm at week 12 or completed the study at week 12 (US patients). NRI has been used to account for all other missing data between baseline and week 24.

^e^Relates to neck, back, and hip pain.

ADA ref, adalimumab reference; BASDAI, Bath Ankylosing Spondylitis Disease Activity Index; LDI, Leeds Dactylitis Index; LEI, Leeds Enthesitis Index; LOCF, last observation carried forward; LSM, least squares mean; MMRM, mixed model for repeated measures; mNAPSI, modified Nail Psoriasis Severity Index; NI, no induction; NRI, non-responder imputation; PBO, placebo; s.d., standard deviation; s.e., standard error; SLK, sonelokimab; SPARCC, Spondyloarthritis Research Consortium of Canada; WI, with induction.

**Extended Data Table 4:**
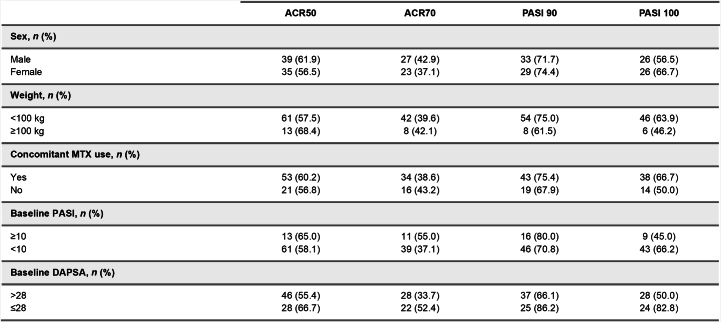
Week 24 ACR and PASI outcomes by subgroup (pooled sonelokimab dose groups)

LOCF from weeks 12 to 24 was applied to patients who switched treatment due to a lack of joint response or for patients in the USA who completed the study at week 12. NRI has been used to account for all other missing data between weeks 0 and 24. For PASI, percentages were based on patients with PsO involving ≥3% BSA at baseline.

ACR, American College of Rheumatology; BSA, body surface area; DAPSA, Disease Activity Index for Psoriatic Arthritis; LOCF, last observation carried forward; MTX, methotrexate; NRI, non-responder imputation; PASI, Psoriasis Area and Severity Index; PsO, psoriasis.

## Data Availability

MoonLake Immunotherapeutics AG is committed to sharing clinical trial data to support legitimate scientific research. Requests from qualified scientific researchers will be promptly reviewed by an internal committee of subject matter experts and/or an independent review panel to assess the feasibility and scientific validity of the request. Data that may be requested include nonidentifiable patient-level and study-level clinical trial data, clinical study reports and protocols. All data provided will be anonymized to respect the privacy of trial participants and are subject to protection of patient privacy and informed consent. A data sharing agreement will need to be signed. Data from this manuscript can be requested by qualified researchers 6 months after product approval in the United States and Europe (or after global development is discontinued) and 24 months after trial completion. Requests should be submitted to the corresponding author or to dataaccessrequests@moonlaketx.com. Responses to such requests can be expected within 60 days. The trial protocol and statistical analysis plan can be found in the Supplementary Materials.
