# Supplementary material for: Sonelokimab, an IL-17A/IL-17F-inhibiting nanobody for active psoriatic arthritis: a randomized, placebo-controlled phase 2 trial
Source: Nat Med. 2025 Oct 6;31(12):4160–71. doi: 10.1038/s41591-025-03971-6 (PMC12705426; doi:10.1038/s41591-025-03971-6)
Supplement: Supplementary file 1 — Supplementary Note (Protocol and Statistical Analysis Plan (SAP)). [file 41591_2025_3971_MOESM1_ESM.pdf]

nature medicine

Supplementary information https://doi.org/10.1038/s41591-025-03971-6

Sonelokimab, an IL-17A/IL-17F-inhibiting nanobody for active psoriatic arthritis: a randomized, placebo-controlled phase 2 trial

In the format provided by the authors and unedited

TITLE PAGE

CLINICAL STUDY PROTOCOL TITLE:

Phase 2, Randomized, Parallel-group, Double-blind, Placebo-controlled Study of Sonelokimab in Patients with Active Psoriatic Arthritis

Protocol Number:	M1095-PSA-201
Test Product:	Sonelokimab
Indication:	Active psoriatic arthritis
Development Phase:	2
Short Title:	Evaluation of Sonelokimab in Patients with Active Psoriatic Arthritis
Sponsor:	MoonLake Immunotherapeutics AG
Address:	Dorfstrasse 29
	6300 Zug
	Switzerland
Regulatory Agency Identifying Numbers:	
EudraCT:	2021-005947-58
IND:	159028
Version:	1.1
Approval Date:	12 Jul 2022

The confidential information in this document is provided to you as an investigator, potential investigator, or consultant for review by you, your staff, and applicable Independent Ethics Committee and/or Institutional Review Board. It is understood that the information will not be disclosed to others without written authorization from MoonLake Immunotherapeutics AG except to the extent necessary to obtain informed consent from those persons, or their legally authorized representative, to whom the medication may be administered.

SPONSOR SIGNATURE PAGE

PROTOCOL TITLE:

Phase 2, Randomized, Parallel-group, Double-blind, Placebo-controlled Study of Sonelokimab in Patients with Active Psoriatic Arthritis

PROTOCOL NUMBER: M1095-PSA-201, Version 1.1

MoonLake Immunotherapeutics AG

[redacted]

[redacted]

Name

[redacted]

Title

1 GENERAL INFORMATION

PROTOCOL TITLE:

Phase 2, Randomized, Parallel-group, Double-blind, Placebo-controlled Study of Sonelokimab in Patients with Active Psoriatic Arthritis

Protocol Number:	M1095-PSA-201
Version:	Version 1.1
Approval Date:	12 Jul 2022
Sponsor:	MoonLake Immunotherapeutics AG<br>Dorfstrasse 29<br>6300 Zug<br>Switzerland
Clinical Research Organization:	[redacted]
Sponsor Signatory:	
Sponsor Medical Expert:	

PROTOCOL AMENDMENT SUMMARY OF CHANGES TABLE

**DOCUMENT HISTORY**	
**Document**	**Approval Date**
Version 1.0	01 Jul 2022
Version 1.1	12 Jul 2022

2 STUDY SYNOPSIS

**Name of Sponsor/Company:**<br>MoonLake Immunotherapeutics AG	
**Name of Product:**<br>Sonelokimab	
**Name of Active Ingredient:**<br>Sonelokimab	
**Title of Study:**<br>Phase 2, Randomized, Parallel-group, Double-blind, Placebo-controlled Study of Sonelokimab in Patients with Active Psoriatic Arthritis	
**Study Center(s):**<br>It is planned that approximately 60 centers will be initiated in multiple countries.	
**Planned Study Period:**<br>Oct 2022 to Feb 2024	**Development Phase:**<br>Phase 2
**Disclosure Statement:**<br>This is a randomized, parallel-group, placebo-controlled study with 5 arms that is participant and investigator blinded.	
**Objectives:**<br>Primary Objective:<br>• To evaluate the efficacy of 3 different dose regimens of sonelokimab (120 mg once every 2 weeks [Q2W], 60 mg Q2W, and 60 mg once every 4 weeks [Q4W]) compared with placebo in the treatment of participants with active psoriatic arthritis (PsA).<br>Secondary Objectives:<br>• To evaluate the safety and tolerability of 3 different dose regimens of sonelokimab (120 mg Q2W, 60 mg Q2W, and 60 mg Q4W) compared with placebo in the treatment of participants with active PsA;<br>• To assess the pharmacokinetics (PK) and immunogenicity of 3 different dose regimens of sonelokimab (120 mg Q2W, 60 mg Q2W, and 60 mg Q4W) in the treatment of participants with active PsA.<br>[redacted]	**Efficacy Endpoints:**<br>Primary Endpoint:<br>• Response rate of at least 50% improvement in the American College of Rheumatology (ACR50) response criteria at Week 12 compared with baseline.<br>Key Secondary Endpoints:<br>1. ACR20 response rate at Week 12 compared with baseline;<br>2. Psoriasis Area and Severity Index (PASI) 90 at Week 12 (in the subgroup of participants with psoriasis [PsO] involving at least 3% body surface area [BSA] at baseline).<br>Other Secondary Endpoints:<br>• Response rate compared with baseline at specified timepoints other than Week 12 for the following:<br>– ACR20;<br>– ACR50;<br>– PASI90.<br>• Response rate compared with baseline at specified timepoints for the following:<br>– ACR70;<br>– Minimal disease activity at Week 12, defined as meeting 5 of the 7 following criteria:<br>o Tender joint count (TJC) 68 ≤1;<br>o Swollen joint count (SJC) 66 ≤1;<br>o PASI ≤1, or PsO affecting ≤1% of BSA;<br>o Patient's Assessment of Arthritis Pain (PtAAP) ≤15 on a 0 to 100 visual analog scale (VAS);<br>o Patient's Global Assessment of Disease Activity (PtGADA) ≤20 on a 0 to 100 VAS;

[redacted]	o Health Assessment Questionnaire Disability Index [HAQ-DI] ≤0.5;<br>o Leeds Enthesitis Index (LEI) ≤1.<br>– PASI75 (in the subgroup of participants with PsO involving at least 3% BSA at baseline);<br>– PASI100 (in the subgroup of participants with PsO involving at least 3% BSA at baseline);<br>– Response rate of at least 50% improvement in the Bath Ankylosing Spondylitis Disease Activity Index (BASDAI);<br>[redacted]<br>• The change from baseline at specified timepoints for the following:<br>– TJC68;<br>– SJC66;<br>– Enthesitis evaluation (LEI and Spondyloarthritis Research Consortium of Canada [SPARCC] enthesitis index);<br>– Leeds Dactylitis Index (LDI);<br>– Modified Nail Psoriasis Severity Index (mNAPSI);<br>– High sensitivity C-reactive protein (hs-CRP);<br>– PtGADA;<br>– Physician's Global Assessment of Disease Activity (PhGADA);<br>– Psoriatic Arthritis Impact of Disease (PsAID)-12;<br>– PtAAP;<br>– HAQ-DI;<br>– Functional Assessment of Chronic Illness Therapy for Fatigue (FACIT-Fatigue);<br>– Short-Form-36 Health Survey Questionnaire-Version 2 (SF-36v2) Mental Component Summary (MCS);<br>– SF-36v2 Physical Component Summary (PCS);<br>– BASDAI;<br>[redacted]<br>**PK and Immunogenicity Endpoints:**<br>• PK of sonelokimab (trough levels);<br>• Anti-drug antibodies (ADA).

[redacted]

Safety Endpoints:

- Incidence, relatedness, severity, and seriousness of adverse events (AE);
- Withdrawal due to AE;
- Clinically relevant abnormalities in vital signs (blood pressure [BP] and heart rate) and body weight;
- Standard 12-lead electrocardiogram (ECG) intervals (RR, PR, QRS, QT, and QT intervals corrected for heart rate using Bazett's and Fridericia's formulas [QTcB and QTcF, respectively]), including clinically relevant abnormalities in ECG variables;
- Clinically relevant abnormalities in clinical laboratory variables (hematology, biochemistry, and urinalysis).

Study Design:

This is a Phase 2 multi-center, randomized, parallel-group, double-blind, placebo-controlled, 2-part study evaluating the efficacy, safety, PK, and immunogenicity of sonelokimab in participants with active PsA. The study includes adalimumab treatment as an active reference arm; however, no formal comparison of sonelokimab versus (vs) adalimumab is planned. Part A will be a 12-week, randomized, parallel-group, double-blind, placebo-controlled period with 5 treatment arms: sonelokimab 120 mg Q2W, sonelokimab 60 mg Q2W, sonelokimab 60 mg Q4W, placebo, or adalimumab 40 mg Q2W. Part B will be a 12-week, parallel-group, double-blind period with 4 treatment arms: sonelokimab 120 mg Q2W, sonelokimab 120 mg Q4W, sonelokimab 60 mg Q4W, or adalimumab 40 mg Q2W.

At the Screening Visit, each participant will provide informed consent, be assigned a unique participant number via the Interactive Response Technology (IRT), be assessed for eligibility with the inclusion and exclusion criteria, and perform study activities as described in the Schedule of Activities (SOA) in Table 1. Participants will undergo screening for up to 4 weeks before randomization to establish eligibility.

On the first day of Part A (Day 1/Week 0) eligible participants will be randomized (1:1:1:1:1) to 1 of 5 treatment arms. Randomization will be stratified by sex (Male/Female) and exposure to biologic agents prior to the Screening Visit (Yes/No). The total number of participants with previous use of biologic agents will be capped at 30%. Treatment arms are as follows:

Treatment Assignment in Part A
Arm 1: Sonelokimab 120 mg Q2W
Arm 2: Sonelokimab 60 mg Q2W
Arm 3: Sonelokimab 60 mg Q4W
Arm 4: Placebo
Arm 5: Adalimumab 40 mg Q2W

Dosing in sonelokimab arms will continue through Week 8. Adalimumab will be given through Week 10. All treatment arms will receive placebo as required to maintain the blinding of the study. Part A will end at Week 12 when the primary efficacy analysis will be performed, comparing each of the sonelokimab treatment arms (sonelokimab 120 mg Q2W, sonelokimab 60 mg Q2W, sonelokimab 60 mg Q4W) vs placebo.

At the beginning of Part B (Week 12) TJC68 and SJC66 response will be assessed in all participants. A responder is defined as a participant who achieves at least a 20% reduction in each of the TJC68 and SJC66 assessments at Week 12 compared with baseline. A non-responder is defined as a participant who does not achieve at least a 20% reduction in each of the TJC68 and SJC66 assessments at Week 12 compared with baseline. In Part B participants will be allocated to treatment via the IRT. Treatment assignments in Part A and the allocation for Part B by responders and non-responders at Week 12 are described below.

Treatment Assignment in Part A	Treatment Allocation in Part B (Based Upon Response at Week 12)	
	Responder	Non-responder
Arm 1: Sonelokimab 120 mg Q2W	Sonelokimab 120 mg Q4W	Adalimumab 40 mg Q2W
Arm 2: Sonelokimab 60 mg Q2W	Sonelokimab 60 mg Q4W	Sonelokimab 120 mg Q4W
Arm 3: Sonelokimab 60 mg Q4W	Sonelokimab 60 mg Q4W	Sonelokimab 120 mg Q4W
Arm 4: Placebo	Sonelokimab 120 mg Q4W	Sonelokimab 120 mg Q4W
Arm 5: Adalimumab 40 mg Q2W	Adalimumab 40 mg Q2W	Sonelokimab 120 mg Q2W

All participants will be followed for safety for 8 weeks after the last dose administration. For participants who complete the study as scheduled and described in the SOA in Table 1, the last dose is to be administered at Week 22, the End-of-Treatment (EOT) Visit at Week 24, and the Safety Follow-up at Week 30. Participants will be regularly evaluated for clinical response throughout the study. On an ongoing basis, safety data will be reviewed by an independent Data and Safety Monitoring Board (DSMB; Section 11.3.12).

Number of Participants:

Approximately 200 participants will be assigned to randomized treatment, for a planned number of approximately 40 participants in each of the 5 treatment arms (with a randomization ratio of 1:1:1:1:1). The total number of participants with previous use of biologic agents will be capped at 30%.

Diagnosis and Main Criteria for Inclusion/Exclusion:

Inclusion Criteria:

To receive randomized treatment assignment, participants must fulfill the following inclusion criteria at the Screening Visit and prior to the initiation of study treatment, unless another time is specified:

1. Participant is ≥18 years of age;
2. Participant has a confirmed diagnosis of PsA per the 2006 ClASsification criteria for Psoriatic ARthritis (CASPAR; see Appendix 1 in Section 21.1) with symptoms for ≥6 months prior to the Screening Visit;
    - *Note*: New X-ray images of hands and/or feet are not required. Results of the most recent X-ray images of hands and/or feet will be captured in the electronic case report form (eCRF). If no previous images exist, X-rays may be obtained as part of routine management at the discretion of the investigator.
3. Participant has active disease (defined by a TJC68 of ≥3 and a SJC66 of ≥3);
4. Participant has either current active PsO or a dermatologist confirmed history of PsO;
5. Participant tests negative for rheumatoid factor (RF) at the Screening Visit;
6. Participant tests negative for anti-cyclic citrullinated peptide (CCP) antibodies at the Screening Visit;
7. Participant must be, in the opinion of the investigator, a suitable candidate for treatment with adalimumab per approved local product information. If a chest X-ray or computerized tomography (CT) scan for tuberculosis (TB) screening is required per local guidance, the X-ray or CT scan must be taken within 3 months prior to the Screening Visit;
8. Participant has had an inadequate response at the Screening Visit (lack of efficacy after ≥12-week duration of therapy) to previous or current treatment with ≥1 non-biologic disease-modifying anti-rheumatic drug (DMARD) at maximally tolerated dose, or participant has an intolerance to or contraindication for DMARDs as defined by the investigator;
9. If the participant is female, she must be of non-childbearing potential or, if of childbearing potential, participant must agree to use highly effective methods of contraception. See Appendix 2 in Section 21.2 for the definition of non-childbearing potential, childbearing potential, and highly effective methods of contraception;
10. Women of childbearing potential (as defined in Appendix 2 in Section 21.2) must have a negative serum human chorionic gonadotropin (hCG) pregnancy test at the Screening Visit and a negative urine pregnancy test at Week 0/Day 1 prior to the first administration of study treatment;

11. If male, participant must be willing to use a condom when sexually active with a partner of childbearing potential (as defined in Appendix 2 in Section 21.2) during the study and for 12 weeks after the last dose of study treatment, unless surgically sterile;
12. Participant is considered reliable and capable of adhering to the protocol, visit schedule, or medication intake according to the judgment of the investigator;
13. Participant is able to understand and provide signed informed consent.

Exclusion Criteria:

Participants who fulfill any of the following exclusion criteria at the Screening Visit or prior to study treatment initiation, unless another time is specified, must be excluded from receiving randomized treatment:

1. Participant with known hypersensitivity to sonelokimab or any of its excipients;
2. Participant with known hypersensitivity to adalimumab or any of its excipients;
3. Participant who currently uses or plans to use 1 or more prohibited treatments specified in this protocol. Prohibited treatments and washout periods are provided in Section 10.5;
4. Participant who has previously failed on anti-interleukin (IL)-17 therapy, defined as inadequate clinical response, according to the investigator's judgment, after at least 16 weeks of treatment; or is unsuitable for anti-IL-17 therapy for any reason according to the investigator's discretion;
5. Participant who has previously failed on anti-tumor necrosis factor alpha (TNFα) therapy, defined as inadequate clinical response, according to the investigator's judgment, after at least 16 weeks of treatment; or is unsuitable for anti-TNFα therapy for any other reason according to the investigator's discretion;
6. Participant who has had previous exposure to more than 2 biologic agents of any type to treat PsA prior to the Screening Visit, including but not limited to IL-17 inhibitors, IL-23 inhibitors, TNFα inhibitors, etc.
   - *Note*: The total number of participants with previous use of biologic agents will be capped at 30% of the total participant population.
7. Participant who has a diagnosis of chronic inflammatory conditions other than PsO or PsA, including but not limited to rheumatoid arthritis, sarcoidosis, systemic lupus erythematosus, Crohn's disease, or ulcerative colitis;
8. Participant who has a diagnosis of arthritis mutilans;
9. Participant who has an active infection or history of infections, including any of the following:
   a. Any infection (exception: common cold) requiring systemic treatment within 14 days before initiation of study treatment;
   b. Serious infection, defined as infection requiring hospitalization or intravenous anti-infective, within 2 months before initiation of study treatment;
   c. History of opportunistic infections caused by uncommon pathogens (e.g., *Pneumocystis jirovicii*, Blastomyces, aspergillus, cryptococcosis), or severe infections caused by common pathogens (e.g., cytomegalovirus, severe herpes zoster e.g. multidermatomal herpes zoster, herpes zoster with organ involvement, ophthalmic herpes, or recurrent herpes zoster [recurrent is defined as 2 episodes within 2 years prior to study treatment initiation]);
   d. History of other opportunistic, recurrent, or chronic infections that, in the opinion of the investigator, might cause study participation to be detrimental to the participant;
   e. *Candida* infection requiring systemic therapy for ≥7 days in the last 12 months prior to study treatment initiation;
   f. Any history of esophageal or systemic candidiasis;
   g. Current active candidiasis or *Candida* infection within the last 1 month prior to the Screening Visit;
   h. Concurrent acute or chronic viral hepatitis B or C, or human immunodeficiency virus (HIV);
   i. Confirmed SARS-CoV-2 infection at the Screening Visit.
   - *Note*: A participant with an initial positive SARS-CoV-2 result may enter the study provided they are asymptomatic and a negative test (polymerase chain reaction [PCR] or antigen) has been provided prior to study treatment initiation.

10. Participant who received a live (including attenuated) vaccination within 8 weeks before study treatment initiation, or planned to receive a live vaccination during the study and up to at least 12 weeks after the last dose of study treatment. Examples of restricted vaccinations include, but are not limited to:
    a. Zoster vaccine live (Zostavax);
    b. Measles-mumps-rubella or measles-mumps-rubella-varicella;
    c. Monovalent live attenuated influenza A (intranasal);
    d. Oral polio;
    e. Rotavirus;
    f. Seasonal trivalent live attenuated influenza (intranasal);
    g. Smallpox;
    h. Oral typhoid;
    i. Varicella (chicken pox);
    j. Yellow fever.
    - *Note*: See Appendix 3 in Section 21.3 for instructions regarding vaccinations to SARS-CoV-2.
11. Participant who received a *Bacillus* Calmette-Guérin (BCG) vaccination within 1 year before study treatment initiation;
12. Participant with:
    a. A history of active TB, i.e., participants having received combination treatment for active TB;
    b. Evidence of TB infection as defined by a positive QuantiFERON® TB Gold test (or interferon-gamma release assay [IGRA] equivalent) at screening, unless the following criteria apply:
        i. A full TB work-up (according to local practice/guidelines) completed within 12 weeks prior to randomization establishes conclusively that the participant has no evidence of active or latent TB;
        ii. Participants positive for latent TB per work-up must have completed sufficient treatment according to local routine clinical practice at least 4 weeks before randomization.
    - *Note 1*: If a QuantiFERON TB Gold test (or IGRA equivalent) at screening is indeterminate, it can be repeated once. The results of the second test are to be used. If the second QuantiFERON TB-Gold test is also indeterminate, the participant should be managed as for participants with positive QuantiFERON TB-Gold test (or IGRA equivalent);
    - *Note 2*: If there is reasonable medical doubt about a potentially false-positive QuantiFERON TB-Gold test (or IGRA equivalent) result, the test can be repeated once after obtaining Medical Monitor approval, and the results of the second test will prevail.
13. Participant with any current nontuberculous mycobacterial (NTM) infection or any history of pulmonary NTM infection at the Screening Visit;
14. Participant with evidence of acute ocular inflammation, including active anterior uveitis (i.e., acute episode), within the last 4 weeks before study treatment initiation;
15. Participant with a concurrent malignancy or a history of malignancy during the past 5 years of the Screening Visit, with the following exceptions:
    a. ≤3 excised or ablated basal cell carcinomas of the skin;
    b. One squamous cell carcinoma of the skin not worse than Stage T1 that has been successfully excised or ablated (no other previous treatments allowed), with no signs of recurrence or metastases for at least the past 2 years before study treatment initiation;
    c. Actinic keratosis;
    d. Squamous cell carcinoma in situ of the skin successfully excised or ablated at >6 months before study treatment initiation;
    e. Localized carcinoma in situ of the cervix, treated and considered cured.
16. Participant with fibromyalgia, osteoarthritis symptoms, or any other condition that in the investigator's opinion may potentially interfere with efficacy assessments;

17. Participant with erythrodermic, guttate, or pustular form of PsO or drug-induced PsO;
18. Participant with a history of a lymphoproliferative disorders, including lymphoma, or current signs and symptoms suggestive of lymphoproliferative disease;
19. Participant with primary immunodeficiencies, prior splenectomy, or suppressive conditions, including participants taking immunosuppressive therapy following organ transplants;
20. Participant who had major surgery (including joint surgery) within 6 months before the Screening Visit, or is planning to have major surgery during the study;
21. Participant with the presence of active suicidal ideation, or positive suicidal behavior at the Screening Visit, as evidenced by Columbia-Suicide Severity Rating Scale (C-SSRS™) assessment that shows any history of suicidal attempt (including an actual attempt, interrupted attempt, or aborted attempt), or suicidal ideation in the past 6 months as indicated by a positive response to either Question 4 or 5 of the C-SSRS at the Screening Visit;
22. Participant has presence of moderately severe depression or severe depression, indicated by a score of ≥15 using the screening PHQ-9. Participants are permitted to use 1 medication to treat depression provided dose is stable for 4 weeks prior to initiation of study treatment. Participants on multiple medications for depression are excluded from the study;
23. Participant with severe cardiovascular comorbidities including history of myocardial infarction, unstable angina pectoris, stroke, heart failure (New York Heart Association [NYHA] classification III or IV), or uncontrolled hypertension (characterized by 2 BP measurements separated by at least 15 minutes with systolic BP >160 mmHg or diastolic BP >100 mmHg);
24. Participant with clinically significant ECG abnormalities on centrally read ECG at the Screening Visit;
25. Participant with any other clinically significant medical conditions or any other reason, including any physical, psychological, or psychiatric condition, that in the opinion of the investigator would compromise the safety or interfere with participation in the study, would make the participant an unsuitable candidate to receive study treatment, or would put the participant at risk;
26. Participant with laboratory abnormalities at the Screening Visit, including any of the following:
    a. Aspartate aminotransferase (AST), alanine aminotransferase (ALT), or alkaline phosphatase >3 times the upper limit of normal (ULN);
    b. Serum direct bilirubin >1.5 × ULN (in the absence of known Gilbert's syndrome);
    c. White blood cell count $<3.0 \times 10^9$/L;
    d. Absolute neutrophil count $<1.5 \times 10^9$/L;
    e. Absolute lymphocyte count $<0.8 \times 10^9$/L;
    f. Platelet count $<100 \times 10^9$/L;
    g. Hemoglobin <85 g/L;
    h. Creatinine clearance <60 mL/min (by Cockcroft Gault formula);
    i. Any other laboratory abnormality which, in the opinion of the investigator compromise the participant's safety, might prevent the participant from completing the study, or might interfere with the interpretation of the study results.
   – *Note*: An initial laboratory result may be retested one time after consultation with the medical monitor. The result of the second test will then be used.
27. Participant is enrolled in another interventional investigational device or drug study, or has been in another investigational device or drug study in the last 28 days prior to the Screening Visit or within 5 half-lives of the investigational study treatment prior to the Screening Visit, whichever is greater;
28. Participant is pregnant or breastfeeding, or plans to become pregnant while enrolled in the study and up to 12 weeks after the last dose of study treatment;
29. Participant has a history of chronic alcohol or drug abuse in the past year prior to the Screening Visit;
30. Participant is an employee, or direct relative of an employee, of the sponsor, at a study site, or of a third-party organization involved in the study.

**Test Product, Dose, and Mode of Administration:**<br>In Part A, the 3 sonelokimab treatment arms are as follows :<br>• Arm 1 (sonelokimab 120 mg Q2W): Sonelokimab 120 mg will be given as subcutaneous (SC) injection at Weeks 0, 2, 4, 6, and 8. Participants will receive a placebo injection at Week 10 to maintain the blind;<br>• Arm 2 (sonelokimab 60 mg Q2W): Sonelokimab 60 mg will be given as SC injection at Weeks 0, 2, 4, 6, and 8. Participants will receive a placebo injection at Week 10 to maintain the blind;<br>• Arm 3 (sonelokimab 60 mg Q4W): Sonelokimab 60 mg will be given as SC injection at Weeks 0, 4, and 8. Participants will receive a placebo injection at Weeks 2, 6, and 10 to maintain the blind.<br>In Part B, after allocation of responders and non-responders, the sonelokimab treatment arms are as follows:<br>• Responders from Arm 1, non-responders from Arm 2, non-responders from Arm 3, and participants from Arm 4 will receive sonelokimab 120 mg Q4W until Week 20. Placebo injections will be given at Weeks 14, 18, and 22 to maintain the blind;<br>• Responders from Arm 2 and responders from Arm 3 will receive sonelokimab 60 mg Q4W until Week 20. Placebo injections will be given at Weeks 14, 18, and 22 to maintain the blind;<br>• Non-responders in Arm 5 will receive sonelokimab 120 mg Q2W at Weeks 12, 14, 16, 18, and 20. Participants will receive a placebo injection at Week 22 to maintain the blind.
**Reference Therapies, Doses, and Durations of Administration:**<br>In Part A, the 2 reference arms are as follows :<br>• Arm 4 (Placebo): Sonelokimab-matching placebo will be given as SC injection at Weeks 0, 2, 4, 6, 8, and 10;<br>• Arm 5 (adalimumab 40 mg Q2W): Adalimumab will be given as 40 mg by SC injection at Weeks 0, 2, 4, 6, 8, and 10.<br>In Part B, adalimumab will be given as 40 mg Q2W administered by SC injection at Weeks 12, 14, 16, 18, 20, and 22 to:<br>• Non-responders from Arm 1;<br>• Responders from Arm 5.
**Duration of Treatment:**<br>The planned study duration for individual participants will be up to 34 weeks, including a screening period of up to 4 weeks, a 24-week treatment period, and an 8-week safety follow-up period after the administration of the last dose, which is scheduled to be administered at Week 22.
**Statistical Methods and Sample Size Calculation:**<br>A sample size of n = 40 participants in each of the sonelokimab 120 mg Q2W, sonelokimab 60 mg Q2W, sonelokimab 60 mg Q4W, or placebo arms (therefore 160 participants in total) results in a power of more than 80%, while assuming an overall 2-sided alpha of 0.025, a placebo ACR50 response rate of 10% and a difference in ACR50 response rate of 30% between placebo and each sonelokimab dose regimen. An additional 40 participants will be randomized in the adalimumab arm. The adalimumab treatment will be used as an active reference arm, however, no formal comparison of sonelokimab or placebo vs adalimumab is planned. Eligible participants will be randomized in a 1:1:1:1:1 ratio to sonelokimab 120 mg Q2W, sonelokimab 60 mg Q2W, sonelokimab 60 mg Q4W, placebo, or adalimumab 40 mg Q2W. The total number of participants with previous use of biologic agents will be capped at 30%. Applicable washout periods for previous biologic agents are outlined in Table 5 of Section 10.5.1. Due to the use of the full analysis set (FAS) with non-responder imputation, no accounting for dropout is required, therefore a total of approximately 200 participants will be randomized.<br>**Analysis Sets**<br>The following populations are defined:<br>• The safety analysis set (SAF) will include all randomized participants who received at least one dose of study treatment.<br>• The FAS will include all randomized participants.<br>For the Part B exploratory analysis, further analysis sets may be defined in the Statistical Analysis Plan (SAP).

Efficacy Analyses

For the primary analysis of the primary endpoint, the hypotheses to be tested are that the proportion of participants who achieve ACR50 at Week 12 compared with baseline in sonelokimab 120 mg Q2W (Arm 1), sonelokimab 60 mg Q2W (Arm 2), or sonelokimab 60 mg Q4W (Arm 3) are different from placebo (Arm 4). A logistic regression model will be used to test the pairwise comparisons (placebo [Arm 4] and either sonelokimab 120 mg Q2W [Arm 1], sonelokimab 60 mg Q2W [Arm 2], or sonelokimab 60 mg Q4W [Arm 3]) and will include fixed effects for treatment and the stratification factors (sex and exposure to biologic agents prior to the Screening Visit). Results will be presented in terms of odds ratios, as well as risk difference and associated 95% CI and estimated p-value. To adjust for multiple testing and to control the family-wise error rate, the Bonferroni-Holm method will be applied first to sonelokimab 120 mg Q2W (Arm 1) and sonelokimab 60 mg Q2W (Arm 2) for the primary endpoint and key secondary endpoints, followed by a step-down procedure to test for sonelokimab 60 mg Q4W (Arm 3).

For the primary analysis of the key secondary endpoints the hypotheses to be tested are that the proportion of participants who achieve the endpoints (ACR20, PASI90) at Week 12 in sonelokimab 120 mg Q2W (Arm 1), sonelokimab 60 mg Q2W (Arm 2), or sonelokimab 60 mg Q4W (Arm 3) are different from placebo (Arm 4). These hypotheses will be tested using the same statistical methods as described for the primary endpoint.

The primary analysis of both primary and key secondary endpoints will be performed for the FAS.

For other secondary and exploratory endpoints, no confirmatory statistical testing will be performed, but statistical tests may be used for exploratory purposes only. Serum concentration data may be used for other analyses (e.g., population PK modeling), which will be reported separately.

For the dichotomous primary and key secondary endpoints non-responder imputation method will be used. For continuous endpoints, no imputation is planned.

Immunogenicity Analysis

Serum immunogenicity (i.e., ADA) data for sonelokimab will be listed and summarized using descriptive statistics. Further analyses may defined in the SAP.

Pharmacokinetic Analyses

Serum concentration data for sonelokimab will be listed and summarized using descriptive statistics. Results may be used for other analyses (e.g., population PK modeling), which will be reported separately.

[redacted]

Safety Analyses

Safety analyses will be done separately for Part A and Part B. Safety data will be summarized by treatment arm using descriptive statistics. No formal statistical testing will be performed for any of the safety endpoints. AEs will be coded using the current version of the Medical Dictionary for Regulatory Activities (MedDRA). SAF will be used for safety analysis.

Date of the Protocol: 12 Jul 2022

3 SCHEDULE OF ACTIVITIES

The Schedule of Activities (SOA) is presented in Table 1.

Table 1 Schedule of Activities

**Period**	**Screening**	**Part A**							**Part B**						**Safety Follow-up**
**Scheduled Week**	--	0	2	4	6	8	10	**12**[a]	14	16	18	20	22	24/EOT[b]	30
**Scheduled Day**	-28 Day to -1 Day	1	15	29	43	57	71	85	99	113	127	141	155	169	8 weeks after last dose
**Visit Window (days)**	--	--	±2	±2	±2	±2	±2	±2	±2	±2	±2	±2	±2	±2	±7
Informed consent	X														
Eligibility criteria[c]	X	X[d]													
Demographics[e]	X														
PsA medical history and previous PsA therapies[f]	X														
Relevant medical history, including current medical conditions	X														
Drug and alcohol history	X														
Smoking history	X														
AE/SAE evaluation[g]	X	X	X	X	X	X	X	X	X	X	X	X	X	X	X
Inquiries for extra-musculoskeletal PsA manifestation								X						X	X
Concomitant medication review	X	X	X	X	X	X	X	X	X	X	X	X	X	X	X
[redacted]															
PtGADA		X	X	X		X		X	X	X		X		X	
PtAAP		X	X	X		X		X	X	X		X		X	
HAQ-DI		X	X	X		X		X	X	X		X		X	
SF-36v2		X		X				X		X		X			

Period	Screening	Part A							Part B						Safety Follow-up
**Scheduled Week**	--	0	2	4	6	8	10	**12[a]**	14	16	18	20	22	24/EOT[b]	30
**Scheduled Day**	-28 Day to -1 Day	1	15	29	43	57	71	85	99	113	127	141	155	169	8 weeks after last dose
**Visit Window (days)**	--	--	±2	±2	±2	±2	±2	±2	±2	±2	±2	±2	±2	±2	±7
FACIT-Fatigue		X		X				X		X		X			
PsAID-12		X		X		X		X		X		X		X	
BASDAI		X		X		X		X		X		X		X	
[redacted]															
TJC68 and SJC66[h]	X	X	X	X		X		X[h]	X	X		X		X	
PhGADA		X	X	X		X		X	X	X		X		X	
BSA affected by PsO (BSA palm method)		X						X						X	
PASI		X		X		X		X		X		X		X	
LDI		X		X		X		X		X		X		X	
Enthesitis evaluation (LEI+SPARCC enthesitis index)		X		X		X		X		X		X		X	
mNAPSI		X						X						X	
12-lead ECG	X							X							X
Height	X														
Weight	X	X						X						X	X
Vital signs[i]	X	X	X	X	X	X	X	X	X	X	X	X	X	X	X
Complete physical exam	X	X		X				X						X	X
TB screening[j]	X														
HIV screening	X														
Hepatitis B/C screening	X														
SARS-CoV-2 virus screening[k]	X														

Period	Screening	Part A							Part B						Safety Follow-up
**Scheduled Week**	--	0	2	4	6	8	10	**12**[a]	14	16	18	20	22	24/EOT[b]	30
**Scheduled Day**	-28 Day to -1 Day	1	15	29	43	57	71	85	99	113	127	141	155	169	8 weeks after last dose
**Visit Window (days)**	--	--	±2	±2	±2	±2	±2	±2	±2	±2	±2	±2	±2	±2	±7
RF, CCP antibodies	X														
FSH testing[l]	X														
Serum pregnancy testing[m]	X														
Urine pregnancy testing[m]		X		X		X		X		X		X		X	
Urinalysis[n]	X	X		X				X		X				X	X
Safety laboratory testing (hematology and chemistry)	X	X		X		X		X		X		X		X	X
ESR	X	X		X		X		X		X		X		X	X
Fasting lipid panel and glucose		X												X	
hs-CRP		X	X	X		X		X	X	X		X		X	
ADA sampling[o]		X	X	X		X		X	X	X		X		X	X
PK sampling[o]		X	X	X		X		X	X	X		X		X	X
[redacted]															
Randomization		X													
Responder/non-responder assessment and treatment allocation[p]								X							
Study treatment administration[q]		X	X	X	X	X	X	X	X	X	X	X	X		

[redacted]

[redacted]

[redacted]

4 TABLE OF CONTENTS

1 GENERAL INFORMATION ... 3
2 STUDY SYNOPSIS ... 5
3 SCHEDULE OF ACTIVITIES ... 14
4 TABLE OF CONTENTS ... 19
4.1 List of Tables ... 24
4.2 List of Figures ... 25
4.3 List of Appendices ... 25
5 LIST OF ABBREVIATIONS AND DEFINITION OF TERMS ... 26
6 INTRODUCTION ... 30
6.1 Background ... 30
6.1.1 Psoriatic Arthritis ... 30
6.1.2 Sonelokimab ... 31
6.2 Study Rationale ... 32
6.3 Benefit – Risk Assessment ... 32
6.3.1 Potential Risks of Sonelokimab ... 33
6.3.2 Risks of Adalimumab ... 34
6.3.3 Overall Benefit – Risk Conclusion ... 34
7 STUDY OBJECTIVES, ENDPOINTS, AND ESTIMANDS ... 36
7.1 Objectives ... 36
7.1.1 Primary Objective ... 36
7.1.2 Secondary Objectives ... 36
7.1.3 Exploratory Objectives ... 36
7.2 Endpoints ... 36
7.2.1 Primary Endpoint and Estimand ... 36
7.2.1.1 Primary Estimand ... 36
7.2.1.2 Sensitivity Estimand ... 37
7.2.2 Key Secondary Endpoints and Estimands ... 38
7.2.2.1 Key Secondary Estimand ... 38
7.2.2.2 Sensitivity Estimand ... 39
7.2.3 Other Secondary Endpoints ... 39
7.2.4 Pharmacokinetic and Immunogencity Endpoints ... 41
7.2.5 Exploratory Endpoint ... 42
7.2.6 Safety Endpoints ... 42

8 OVERALL STUDY DESIGN AND PLAN 43
8.1 Overview of the Study Design 43
8.2 Start of Study Definition 45
8.3 Visits 45
8.3.1 Screening Visit 45
8.3.2 Randomization and Visits During the Treatment Period 45
8.3.3 Week 12 Visit 47
8.3.4 End-of-Treatment Visit 47
8.3.5 Safety Follow-up Visit 47
8.4 End of Study Definition 48
8.5 Study Treatment After the End of Study 48
8.6 Rescheduling Visits 48
8.7 Unscheduled Visits 48
8.8 Protocol Deviations 49
8.9 Rationale for the Study Design and Selection of Dose 49
9 STUDY POPULATION 51
9.1 Eligibility Criteria 51
9.1.1 Inclusion Criteria 51
9.1.2 Exclusion Criteria 52
9.2 Screen Failures 56
9.3 Strategies for Recruitment and Retention 57
9.4 Study Treatment Discontinuation 57
9.4.1 Individual Participant Study Treatment Stopping Rules 57
[redacted]
9.5 Safety Follow-up After Treatment Discontinuation 60
9.6 Withdrawal of Participants 60
9.6.1 Withdrawal of Consent 60
9.6.2 Non-Compliance 61
9.7 Lost to Follow-up 61
9.8 Discontinuation of Study Sites 61
9.9 Discontinuation of Study 61
10 STUDY TREATMENT 62
10.1 Administration of Study Treatment(s) 62
10.1.1 Sonelokimab 64
10.1.2 Placebo 65
10.1.3 Adalimumab 65
10.2 Study Treatment, Packaging, and Labeling 66

10.2.1 Packaging 66
10.2.2 Labeling 66
10.2.3 Storage 66
10.3 Study Treatment Accountability 66
10.4 Missed Dose 67
10.5 Prior and Concomitant Medications 67
10.5.1 Prohibited Medications and Therapies 68
10.5.2 Permitted Concomitant Therapy 69
10.6 Lifestyle Considerations 69
10.7 Blinding and Randomization of Study Treatment(s) 70
10.7.1 Part A 70
10.7.2 Part B 70
10.7.3 Maintenance of Blinding 70
10.8 Procedure for Breaking the Randomization Code 71
11 STUDY ASSESSMENTS AND PROCEDURES 72
11.1 Screening Assessments 72
11.1.1 Psoriatic Arthritis Medical History and Prior Medications 72
11.1.2 Medical History and Prior Medications 72
11.1.3 Demographics 72
11.1.4 QuantiFERON TB Gold Testing 72
11.1.5 Hepatitis B and Hepatitis C Testing 73
11.1.6 Human Immunodeficiency Virus Testing 74
11.1.7 SARS-CoV-2 Testing 74
11.1.8 Height and Weight 74
11.2 Efficacy Assessments 74
11.2.1 Composite Disease Assessments 74
11.2.1.1 American College of Rheumatology 20/50/70 74
[redacted]
11.2.1.3 Minimal Disease Activity 76
[redacted]
11.2.2 Patient Reported Outcomes 76
[redacted]
11.2.2.2 Patient's Global Assessment of Disease Activity 77
11.2.2.3 Patient's Assessment of Arthritis Pain 77
11.2.2.4 Health Assessment Questionnaire Disability Index 77
11.2.2.5 Short-Form-36 Health Survey-Version 2 78

11.2.2.6 Functional Assessment of Chronic Illness Therapy-Fatigue 78
11.2.2.7 Psoriatic Assessment Impact of Disease-12 79
11.2.2.8 Bath Ankylosing Spondylitis Disease Activity Index 79
11.2.3 Physician Assessments 80
11.2.3.1 Tender Joint Count and Swollen Joint Count 80
11.2.3.2 Physician's Global Assessment of Disease Activity 80
11.2.3.3 Psoriasis Area and Severity Index 80
11.2.3.4 Body Surface Area Affected by Psoriasis 82
11.2.3.5 Leeds Dactylitis Index 82
11.2.3.6 Enthesitis Evaluation (Leeds Enthesitis Index and Spondyloarthritis Research Consortium of Canada Enthesitis Index) 83
11.2.3.7 Modified Nail Psoriasis Severity Index 84
11.3 Safety Assessments 86
11.3.1 Definitions 86
11.3.1.1 Adverse Events 86
11.3.1.2 Events Meeting the Adverse Events Definition 86
11.3.1.3 Events Not Meeting the Adverse Events Definition 87
11.3.1.4 Serious Adverse Event 87
11.3.1.5 Treatment-Emergent Adverse Event 88
11.3.1.6 Adverse Event of Special Interest 88
11.3.1.7 Extra-musculoskeletal Manifestations for Additional Monitoring 89
11.3.1.8 Pregnancy 89
11.3.1.9 Deaths 89
11.3.2 Time Period and Frequency for Collecting Adverse Events and Serious Adverse Events Information 89
11.3.3 Method of Detecting Adverse Events and Serious Adverse Events 90
11.3.4 Recording of Adverse Events and Serious Adverse Events 90
11.3.4.1 Assessment of Intensity 90
11.3.4.2 Assessment of Causality 91
11.3.4.3 Other Data to Be Recorded for Adverse Events 92
11.3.5 Follow-up of Adverse Events or Serious Adverse Events 92
11.3.6 Reporting of Serious Adverse Events 92
11.3.6.1 Safety Reporting to Health Authorities, Independent Ethics Committees/Institutional Review Boards, and Investigators 93

11.3.6.2 24/7 Medical Emergency Coverage for Urgent Protocol-related Medical Questions 93
11.3.7 Laboratory Assessments 93
11.3.8 Electrocardiogram Assessments 95
11.3.9 Physical Examination 95
11.3.10 Vital Signs 95
[redacted]
11.3.12 Data and Safety Monitoring Board 96
11.4 Overdose 96
11.5 Hypersensitivity Reactions and Anaphylaxis 97
11.6 Immunogenicity Assessments 97
11.7 Pharmacokinetics 97
[redacted]
12 STATISTICAL CONSIDERATIONS 98
12.1 Sample Size and Power 98
12.2 Populations for Analysis 99
12.2.1 Analysis Sets 99
12.2.2 Participant Disposition 99
12.3 Statistical Analyses 99
12.3.1 General Considerations 100
12.3.2 Primary Endpoints 101
12.3.2.1 Primary Analysis 101
12.3.2.2 Type I Error Control for the Primary and Key Secondary Endpoints 101
12.3.3 Secondary Endpoints 102
12.3.3.1 Key Secondary Endpoints 102
12.3.3.2 Other Secondary Endpoints 102
12.3.3.3 Pharmacokinetic Analyses 103
12.3.3.4 Immunogenicity Analyses 103
12.3.3.5 Part B Analysis 103
[redacted]
12.3.4 Safety Analyses 103
12.3.4.1 Adverse Events 104
12.3.4.2 Other Safety Endpoints 105
12.4 Subgroup Analyses 105
12.5 Handling of Missing Data 106
12.6 Primary Analysis Timing 106

13 ETHICS 107
13.1 Independent Ethics Committee/Institutional Review Board 107
13.2 Written Informed Consent 107
14 QUALITY CONTROL AND QUALITY ASSURANCE 108
14.1 Conduct of the Study 108
14.2 Site Monitoring 108
15 DATA HANDLING AND RECORD KEEPING 110
15.1 Case Report Forms/Source Data Handling 110
15.2 Data Protection 110
15.3 Dissemination of Clinical Study Data 110
15.4 Retention of Essential Documents 111
16 FINANCING AND INSURANCE 112
16.1 Contractual and Financial Details 112
16.2 Insurance, Indemnity, and Compensation 112
16.3 Financial Disclosure 112
17 PUBLICATION POLICY 113
18 CONFLICT OF INTEREST POLICY 114
19 SIGNATURE OF INVESTIGATOR 115
20 REFERENCE LIST 116
21 APPENDICES 121
21.1 Appendix 1 ClASsification criteria for Psoriatic ARthritis (CASPAR) 121
21.2 Appendix 2 Women of Childbearing Potential and Highly Effective Contraception 122
21.3 Appendix 3 SARS-CoV-2 (COVID-19) Guidance 124
21.4 Appendix 4 Clinical Criteria for Diagnosing Anaphylaxis 125

4.1 List of Tables

Table 1 Schedule of Activities 14
Table 2 Randomized Treatment Assignment in Part A 62
Table 3 Treatment Allocation in Part B Following Response Assessment at Week 12 .63
Table 4 Composition of the Placebo and Sonelokimab Drug Product 60 mg/mL and 120 mg/mL 64
Table 5 Prohibited Medications and Therapies and Washout Periods Prior to Initiating Study Treatment 68
Table 6 Reference Circumference for Hands (measured in cm) 82
Table 7 Reference Circumference for Feet (measured in cm) 83
Table 8 mNAPSI Scores for Onycholysis 85
Table 9 mNAPSI Scores for Number of Pits 85
Table 10 mNAPSI Scores for Percent of Nail With Crumbling Present 85
Table 11 Definition of Causality Terms for Adverse Events 91

Table 12 Clinical Laboratory Tests 94
Table 13 Populations for Analysis 99

4.2 List of Figures

Figure 1 Study Design 44
Figure 2 Interpretation and Management of HBV Serologic Test Results....................73

4.3 List of Appendices

Appendix 1 ClASsification criteria for Psoriatic ARthritis (CASPAR)

Appendix 2 Women of Childbearing Potential and Highly Effective Contraception

Appendix 3 SARS-CoV-2 (COVID-19) Guidance

Appendix 4 Clinical Criteria for Diagnosing Anaphylaxis

5 LIST OF ABBREVIATIONS AND DEFINITION OF TERMS

ACR	American College of Rheumatology
ACR50	response rate of participants achieving at least a 50% improvement in the American College of Rheumatology criteria
ADA	anti-drug antibody
AE	adverse event
AESI	adverse event of special interest
ALCOAC	attributable, legible, contemporaneous, original, accurate, and complete
ALT	alanine aminotransferase
AST	aspartate aminotransferase
BASDAI	Bath Ankylosing Spondylitis Disease Activity Index
BCG	*Bacillus* Calmette-Guérin
BP	blood pressure
BSA	body surface area
CASPAR	ClASsification criteria for Psoriatic ARthritis
CCP	cyclic citrullinated peptide
CONSORT	Consolidated Standards of Reporting Trials
C-SSRS	Columbia-Suicide Severity Rating Scale
CT	computerized tomography
CTCAE	Common Terminology Criteria for AEs
[redacted]	
DMARD	disease-modifying anti-rheumatic drug
DSMB	Data and Safety Monitoring Board
ECG	electrocardiogram
eCOA	electronic clinical outcome assessment
eCRF	electronic case report form
EDC	electronic data capture
EMA	European Medicines Agency
EOT	End-of-Treatment
ePRO	electronic patient reported outcome
ESR	erythrocyte sedimentation rate
EudraCT	European Union Drug Regulatory Agency Clinical Trial
FACIT-Fatigue	Functional Assessment of Chronic Illness Therapy for Fatigue
FAS	full analysis set
FDA	Food and Drug Administration
FSH	follicle stimulating hormone
GCP	Good Clinical Practice
GGT	gamma-glutamyl transferase
GLMM	generalized linear mixed model

GMP	Good Manufacturing Practice
HAQ-DI	Health Assessment Questionnaire Disability Index
HBc Ab	hepatitis B core antibody
HBs AG	hepatitis B surface antigen
HBV	hepatitis B virus
hCG	human chorionic gonadotropin
HCV	hepatitis C virus
HIV	human immunodeficiency virus
HRQoL	health-related quality of life
HS	hidradenitis suppurativa
hs-CRP	high sensitivity C-reactive protein
IB	Investigator's Brochure
IBD	inflammatory bowel disease
ICF	informed consent form
ICH	International Council for Harmonisation of Technical Requirements for Pharmaceuticals for Human Use
IEC	Independent Ethics Committee
IGRA	interferon-gamma release assay
IL	interleukin
IRB	Institutional Review Board
IRT	interactive response technology
ITT	intention-to-treat
JAKi	Janus kinase inhibitor
LDI	Leeds Dactylitis Index
LEI	Leeds Enthesitis Index
mAb	monoclonal antibody
MAR	missing at random
MCS	Mental Component Summary of the Short-Form-36 Health Survey, Version 2
MedDRA	Medical Dictionary for Regulatory Activities
MMRM	mixed model for repeated measures
mNAPSI	modified Nail Psoriasis Severity Index
NCT	National Clinical Trial
NF	National Formulary
NRS	numerical rating scale
NSAID	non-steroidal anti-inflammatory drug
NTM	nontuberculous mycobacterial
NYHA	New York Heart Association
PASI	Psoriasis Area and Severity Index
PCR	Polymerase Chain Reaction
PCS	Physical Component Summary of the Short-Form-36 Health Survey, Version 2

PEF	peak expiratory flow
Ph. Eur.	European Pharmacopoeia
PhGADA	Physician's Global Assessment of Disease Activity
PHQ-9	Patient Health Questionnaire-9
PK	pharmacokinetic
PRO	patient reported outcome
PsA	psoriatic arthritis
PsAID	Psoriatic Arthritis Impact of Disease
PsO	psoriasis
PT	preferred term
PtAAP	Patient's Assessment of Arthritis Pain
PtGADA	Patient's Global Assessment of Disease Activity
PVSS	Pharmacovigilance and Safety Services
Q2W	once every 2 weeks
Q4W	once every 4 weeks
QoL	quality of life
q.s.	as much as is sufficient
QTcB	heart rate corrected qt interval using Bazett's formula
QTcF	heart rate corrected qt interval using Fridericia's formula
R/L	right/left
RBC	red blood cell
RF	rheumatoid factor
SAE	serious adverse event
SAF	safety analysis set
SAP	Statistical Analysis Plan
SC	subcutaneous
SD	standard deviation
SF-36	Short-Form-36 Health Survey Questionnaire
SF-36v2	Short-Form-36 Health Survey Questionnaire-Version 2
SJC	Swollen Joint Count
SOA	schedule of activities
SOC	system organ class
SOP	standard operating procedure
SPARCC	Spondyloarthritis Research Consortium of Canada
SUSAR	suspected, unexpected serious adverse reaction
TB	tuberculosis
TEAE	treatment-emergent adverse event
TJC	Tender Joint Count
TNF	tumor necrosis factor
TNFα	tumor necrosis factor alpha

ULN	upper limit of normal
USP	United States Pharmacopeia
VAS	visual analog scale
vs	versus
WBC	white blood cell
WOCBP	women of childbearing potential

6 INTRODUCTION

6.1 Background

6.1.1 Psoriatic Arthritis

Psoriatic arthritis (PsA) is a chronic inflammatory arthritis associated with psoriasis (PsO) that primarily affects the peripheral joints. The clinical features of PsA are heterogeneous, comprising a range of musculoskeletal manifestations such as peripheral arthritis, oligoarthritis, axial involvement, dactylitis, and enthesitis [Ogdie A. et al., 2020]. In addition to PsO, patients with PsA may develop other extra-musculoskeletal manifestations such as ocular inflammation (e.g., uveitis) and inflammatory bowel disease (IBD) [Ritchlin C.T. et al., 2017]. PsA occurs in up to 30% of patients with PsO and typically presents in those aged between 30 to 40 years, with a similar prevalence observed in males and females [FitzGerald O. et al., 2021; Ritchlin C.T. et al., 2017]. Skin lesions usually precede arthritic symptoms by an average of 10 years, although in ~15% of cases, arthritis occurs simultaneously or is the first disease manifestation [Mease P.J. et al., 2018; Ritchlin C.T. et al., 2017]. The overall prevalence of PsA in Western countries is approximately 0.2% to 0.5% [Scotti L. et al., 2018].

Around 40% to 60% of patients with PsA develop erosive and deforming arthritis with resultant severe functional impairment due to joint pain, restricted mobility, and fatigue [Duarte G.V. et al., 2012; Gottlieb A. et al., 2008]. PsA is associated with increased incidence of cardiovascular, metabolic and psychiatric comorbidities, including hypertension, diabetes mellitus, obesity, depression, anxiety, fibromyalgia, and others [FitzGerald O. et al., 2021; Gossec L. et al., 2014; Ogdie A. et al., 2015]. Depression and anxiety affect an estimated 10% to 30% of patients with PsA [FitzGerald O. et al., 2021]. The symptom burden of PsA and associated comorbidities has a substantial negative impact on patient quality of life (QoL) [FitzGerald O. et al., 2021; Gossec L. et al., 2020]. Patients with PsA self-report significant functional impairment and restrictions to activities of daily living [Kavanaugh A. et al., 2016]. PsA greatly impacts patients' professional lives; up to 50% become unemployed and many patients report reduced work productivity, which contributes to the significant economic burden of PsA [Husni M.E. et al., 2017].

Despite the profound burden for patients and evidence that delaying the initiation of appropriate treatment by 6 months or more worsens disease outcomes [Gladman D.D. et al., 2011; Haroon M. et al., 2015], PsA remains underdiagnosed and undertreated [Armstrong A.W. et al., 2013; Villani A.P. et al., 2015]. Non-steroidal anti-inflammatory drugs (NSAIDs), intra-articular glucocorticoids and oral disease-modifying anti-rheumatic drugs (DMARDs) remain conventional first-line treatment options and can provide effective symptom relief in many patients with PsA [Gossec L. et al., 2020]. Patients with inadequate response or intolerance to oral DMARDs are candidates for targeted biologic agents, most commonly tumor necrosis factor (TNF) inhibitors [Coates L.C. et al., 2016]. More recently, the American College of Rheumatology 2018 guidelines recommended TNF inhibitors as first-line agents in preference to oral DMARDs for some treatment-naïve patients with active PsA [Singh J.A. et al., 2019].

While TNF inhibitors such as adalimumab are still perceived as the gold standard in the targeted therapy of PsA, in clinical studies they only induce a 20% improvement of the tender and swollen joint count (and improvement in at least 3 out of 5 American College of Rheumatology [ACR] disease criteria [i.e., ACR20 response]) in approximately 50% to 60% of patients [Mease P. et al., 2005]. Other treatments that may be considered in patients with PsA include the interleukin (IL)-17A inhibitors, IL-23 inhibitors, Janus kinase inhibitors (JAKi), or apremilast. IL-17A inhibitors such as secukinumab or ixekizumab have demonstrated non-inferiority to adalimumab in head-to-head studies in PsA with higher levels of skin improvement [McInnes I.B. et al., 2020; Mease P.J. et al., 2020] and are most frequently prescribed in patients with concomitant moderate to severe PsO and/or contraindications to TNF inhibitors [Coates L.C. et al., 2016; Singh J.A. et al., 2019]. Recent large multi-national real-world studies show that, despite treatment with biologic agents, substantial pain, and/or fatigue persist in patients with PsA and that these are associated with reduced health-related quality of life (HRQoL), physical function, and work productivity suggesting an unmet need for additional PsA therapies [Conaghan P.G. et al., 2020]. No drug currently approved for PsA has shown superiority to adalimumab in clinical studies. There are no dual IL-17A and IL-17F inhibitors currently licensed for use in PsA.

The etiology of PsA is not fully elucidated; however, evidence suggests that disease pathophysiology is underpinned by IL-17 and Th17-driven inflammation [Raychaudhuri S.P. et al., 2012]. IL-17 expression is elevated in the serum and synovial fluid of PsA patients, and its level strongly correlates with disease activity [McGonagle D.G. et al., 2019]. Targeted IL-17A agents, such as secukinumab, have successfully demonstrated efficacy in improving PsA disease outcome measures and functional scores [Hueber W. et al., 2010]. IL-17F shares ~50% sequence homology and overlapping pro-inflammatory functions with IL-17A [Hymowitz S.G. et al., 2001; Tsukazaki H. et al., 2020]. Preclinical data indicates that dual IL-17A and IL-17F blockade may decrease inflammation in PsA more than IL-17A inhibition alone. Specifically, combined blockade of IL-17A and IL-17F decreased in vitro production of pro-inflammatory cytokines significantly more than IL-17A alone in Th17-stimulated synoviocytes from patients with PsA and primary normal human dermal fibroblasts [Glatt S. et al., 2018].

6.1.2 Sonelokimab

Sonelokimab is a tri-specific nanobody that selectively inhibits IL-17A and IL-17F. The central moiety binds to human serum albumin to extend the half-life *in vivo*. Sonelokimab is expressed in the yeast *Pichia pastoris* and with a molecular weight of 40.1 kDa; it is therefore around a quarter of the size of conventional monoclonal antibodies (150 kDa).

In conjunction with selective inhibition of IL-17A and IL-17F, potential advantages that can differentiate sonelokimab from conventional monoclonal antibodies include its smaller size and albumin-binding capacity. The smaller size of the sonelokimab nanobody compared with conventional monoclonal antibodies (40 kDa versus [vs] 150 kDa) may enable differential deep tissue penetration [Li Z. et al., 2016; Sun S. et al., 2021]. Furthermore, the albumin-binding domain

provides a mechanism for enrichment of sonelokimab at sites of chronic inflammation associated with edema and accumulation of albumin-rich fluid. As the latter underlies joint swelling in arthritis, the relatively small size and the albumin-binding capacity in combination are characteristics predicting enhanced joint penetration [Coppieters K. et al., 2006; Jovčevska I. et al., 2020].

In Phase 1 (M1095-PSO-101) and Phase 2 (M1095-PSO-201) studies, in patients with moderate to severe plaque-type PsO, sonelokimab demonstrated a rapid improvement with considerable numbers of patients achieving high levels of clinical response with no unexpected safety findings [Papp K.A. et al., 2021; Svecova D. et al., 2019]. In the Phase 1 (M1095-PSO-101) in patients who received sonelokimab a decrease to normal skin levels was observed in the expression of several disease-related genes encoding key markers such as IL-17A, IL-17F, IL-8, CCL20 and keratin-16 [Svecova D. et al., 2019].

Further details are provided in the Investigator's Brochure (IB).

6.2 Study Rationale

Sonelokimab is 1 of 2 dual IL-17A and IL-17F blockers in development for PsA, alongside UCB's bimekizumab. In a Phase 2 study in PsA, bimekizumab has demonstrated high levels of ACR50 response rates with absolute numbers above those previously observed in similar studies with TNF or IL-17A inhibition [Ritchlin C.T. et al., 2017]. Compared to bimekizumab, it is hypothesized that the low molecular weight of sonelokimab and albumin-binding site may enhance drug penetration into inflamed joints. In a murine arthritis model, it was demonstrated that albumin-binding nanobodies enrich at sites of joint inflammation compared to nanobodies without an albumin-binding domain correlating with higher levels of disease control [Coppieters K. et al., 2006].

This current PsA Phase 2 study will be a randomized, parallel-group, double-blind, placebo-controlled study of sonelokimab in patients with active PsA. This study design is appropriate for providing proof-of-concept by administering 3 different dose regimens (120 mg once every 2 weeks [Q2W], 60 mg Q2W, and 60 mg once every 4 weeks [Q4W]) and assessing the safety and efficacy of sonelokimab compared with placebo in patients with active PsA. The study will include adult patients with a confirmed diagnosis of PsA per the 2006 ClASsification criteria for Psoriatic ARthritis (CASPAR) [Taylor W. et al., 2006] with symptoms for ≥6 months prior to the Screening Visit. Results from this study will be used to inform future development of sonelokimab for the treatment of PsA and related immunological diseases.

Further details on the rationale for the study design and dose selection are available in Section 8.9.

[redacted]

[redacted]

[redacted]

6.3.2 Risks of Adalimumab

The active reference therapy in this clinical study is adalimumab a tumor necrosis factor alpha (TNFα) blocker approved by the United States Food and Drug Administration (FDA) and European Medicines Agency (EMA) and indicated for a variety of immune-related disorders in adult and pediatric patients, including reducing signs and symptoms in adult patients with PsA. The most common adverse reactions (>10%) are upper respiratory infection, headache, rash, and sinusitis. Warnings and precautions include serious infections including TB, malignancies, hypersensitivity reactions, hepatitis B reactivation, neurologic reactions, hematological reactions, heart failure, increased risk of infection when used with other TNFα blockers, and autoimmunity.

6.3.3 Overall Benefit – Risk Conclusion

Patients with PsA have high symptom burden which has a profound impact on patient QoL and contributes to a significant deterioration in physical and mental health. Although sonelokimab has not been studied in patients with PsA, studies have been conducted in healthy volunteers and patients with PsO and sonelokimab has been well tolerated and safe in these populations. Preliminary clinical evidence indicates that blockade of IL-17A and IL-17F has the potential to induce high levels of clinical response in patients with PsA.

The study has been designed to minimize potential risks to participants. All participants will undergo screening procedures aimed at reducing the likelihood and impact of any such risks. Regular safety monitoring during the treatment period for all participants will ensure that any unanticipated effects of study participation are identified promptly and managed appropriately. In addition, safety data will be reviewed on an ongoing basis by an independent DSMB.

Based on the current knowledge, the benefit-risk evaluation for patients participating in this clinical study with sonelokimab is considered acceptable.

7 STUDY OBJECTIVES, ENDPOINTS, AND ESTIMANDS

7.1 Objectives

7.1.1 Primary Objective

The primary objective is to evaluate the efficacy of 3 different dose regimens of sonelokimab (120 mg Q2W, 60 mg Q2W, and 60 mg Q4W) compared with placebo in the treatment of participants with active PsA.

7.1.2 Secondary Objectives

The secondary objectives are as follows:

- To evaluate the safety and tolerability of 3 different dose regimens of sonelokimab (120 mg Q2W, 60 mg Q2W, and 60 mg Q4W) compared with placebo in the treatment of participants with active PsA;
- To assess the pharmacokinetics (PK) and immunogenicity of 3 different dose regimens of sonelokimab (120 mg Q2W, 60 mg Q2W, and 60 mg Q4W) in the treatment of participants with active PsA.

[redacted]

7.2 Endpoints

7.2.1 Primary Endpoint and Estimand

7.2.1.1 Primary Estimand

The primary estimand assesses treatment success at Week 12 defined as achieving an ACR50 response. A treatment failure at Week 12 will be defined as not achieving an ACR50 response, discontinuing treatment for any reason prior to Week 12, or receiving prohibited medication. The attributes of the primary estimand are as follows:

- Target population: adults with a confirmed diagnosis of PsA per the 2006 CASPAR [Taylor W. et al., 2006] with symptoms for ≥6 months prior to the Screening Visit;
- Treatment: sonelokimab at 3 different regimens: sonelokimab 120 mg Q2W, sonelokimab 60 mg Q2W, or sonelokimab 60 mg Q4W;
- Primary endpoint: is percentage of participants achieving ACR50, i.e., at least 50% improvement in the ACR response criteria at Week 12 compared with baseline;

- Intercurrent events: intercurrent events include the following:
  - Use of prohibited concomitant medication (Section 10.5): participants will be analyzed as a treatment failure after taking prohibited medication, i.e., non-responder imputation will be performed after the first episode of prohibited medication for all future assessments (composite strategy);
  - Withdrawal from treatment due to any reason (Section 9.6): participants will be considered non-responders to treatment if they discontinue the study prior to the Week 12 Visit (composite strategy);
  - Study treatment non-adherence (missing doses) or drug administration error: participants will be analyzed the same if the event occurs or not (treatment policy strategy).
- Population level summary: the primary estimator will be the improvement compared to placebo during the 12-week Part A based on the primary endpoint.

7.2.1.2 Sensitivity Estimand

The sensitivity estimand will include data from participants in the target population of the primary estimand plus data collected after the withdrawal of study treatment through Week 12 from participants who discontinue study treatments prior to Week 12 for reasons other than lack of efficacy or response.

The attributes of the sensitivity estimand of the primary endpoint are as follows:

- Target population: adults with a confirmed diagnosis of PsA per the 2006 CASPAR [Taylor W. et al., 2006] with symptoms for ≥6 months prior to the Screening Visit;
- Treatment: sonelokimab at 3 different regimens: sonelokimab 120 mg Q2W, sonelokimab 60 mg Q2W, or sonelokimab 60 mg Q4W;
- Primary endpoint: is percentage of participants achieving ACR50, i.e., at least 50% improvement in the ACR response criteria at Week 12 compared with baseline;
- Intercurrent events: intercurrent events include the following:
  - Use of prohibited concomitant medication (Section 10.5): participants will be analyzed as a treatment failure after taking prohibited medication, i.e., non-responder imputation will be performed after the first episode of prohibited medication for all future assessments (composite strategy);
  - Withdrawal from treatment due to lack of efficacy (Section 9.6): participants will be considered non-responders to treatment if they discontinue the study prior to the Week 12 Visit (composite strategy);

  - Withdrawal from treatment due to any reason other than lack of efficacy (Section 9.6): participants will continue to be followed and use their observed Week 12 Visit (treatment policy strategy). Any missing data for participants who do not have a Week 12 visit will be assumed to be missing at random;
  - Study treatment non-adherence (missing doses) or drug administration error: participants will be analyzed the same if the event occurs or not (treatment policy strategy).

7.2.2 Key Secondary Endpoints and Estimands

7.2.2.1 Key Secondary Estimand

The attributes of the key secondary estimand are as follows:

- Target population: adults with a confirmed diagnosis of PsA per the 2006 CASPAR [Taylor W. et al., 2006] with symptoms for ≥6 months prior to the Screening Visit;
- Treatment: sonelokimab at 3 different regimens: sonelokimab 120 mg Q2W, sonelokimab 60 mg Q2W, or sonelokimab 60 mg Q4W;
- Key secondary endpoints: The key secondary endpoints are as follows:

1. ACR20 response rate at Week 12 compared with baseline;
2. Psoriasis Area and Severity Index (PASI) 90 at Week 12 compared with baseline in the subgroup of participants with PsO involving at least 3% body surface area (BSA) at baseline.

- Intercurrent events: intercurrent events include the following:
  - Use of prohibited concomitant medication (Section 10.5): participants will be analyzed as a treatment failure after taking prohibited medication, i.e., non-responder imputation will be performed after the first episode of prohibited medication for all future assessments (composite strategy);
  - Withdrawal from treatment due to any reason (Section 9.6): participants will be considered non-responders to treatment if they discontinue the study prior to the Week 12 Visit due to any reason (composite strategy);
  - Study treatment non-adherence (missing doses) or drug administration error: participants will be analyzed the same if the event occurs or not (treatment policy strategy).
- Population level summary: the estimator will be the improvement compared to placebo during the 12-week Part A based on the endpoint of interest.

7.2.2.2 Sensitivity Estimand

The sensitivity estimand will include data from participants in the target population of the key secondary estimand plus data collected after the withdrawal of study treatment through Week 12 from participants who discontinue study treatments prior to Week 12 for reasons other than lack of efficacy or response.

The attributes of the sensitivity estimand of the key secondary endpoints are as follows:

- Target population: adults with a confirmed diagnosis of PsA per the 2006 CASPAR [Taylor W. et al., 2006] with symptoms for ≥6 months prior to the Screening Visit;
- Treatment: sonelokimab at 3 different regimens: sonelokimab 120 mg Q2W, sonelokimab 60 mg Q2W, or sonelokimab 60 mg Q4W;
- Key secondary endpoints: The key secondary endpoints are as follows:

1. ACR20 response rate at Week 12 compared with baseline;
2. PASI90 at Week 12 compared with baseline in the subgroup of participants with PsO involving at least 3% BSA at baseline.

- Intercurrent events: intercurrent events include the following:
  - Use of prohibited concomitant medication (Section 10.5): participants will be analyzed as a treatment failure after taking prohibited medication, i.e., non-responder imputation will be performed after the first episode of prohibited medication for all future assessments (composite strategy);
  - Withdrawal from treatment due to lack of efficacy (Section 9.6): participants will be considered non-responders to treatment if they discontinue the study prior to the Week 12 Visit (composite strategy);
  - Withdrawal from treatment due to any reason other than lack of efficacy (Section 9.6): participants will continue to be followed and use their observed Week 12 Visit (treatment policy strategy). Any missing data for participants who do not have a Week 12 visit will be assumed to be missing at random;
  - Study treatment non-adherence (missing doses) or drug administration error: participants will be analyzed the same if the event occurs or not (treatment policy strategy).

7.2.3 Other Secondary Endpoints

These endpoints will be assessed at time points as outlined in the Schedule of Activities (SOA) in Section 3, Table 1, if not indicated otherwise.

- Response rate compared with baseline at specified timepoints other than Week 12 for the following:
  - ACR20;
  - ACR50;
  - PASI90.
- Response rate compared with baseline at specified timepoints for the following:
  - ACR70;
  - Minimal disease activity at Week 12, defined as meeting 5 of the 7 following criteria:
    - Tender joint count (TJC) 68 ≤1;
    - Swollen joint count (SJC) 66 ≤1;
    - PASI ≤1, or PsO affecting ≤1% of BSA;
    - Patient's Assessment of Arthritis Pain (PtAAP) ≤15 on a 0 to 100 visual analog scale (VAS);
    - Patient's Global Assessment of Disease Activity (PtGADA) ≤20 on a 0 to 100 VAS;
    - Health Assessment Questionnaire Disability Index [HAQ-DI] ≤0.5;
    - Leeds Enthesitis Index (LEI) ≤1.
  - PASI75 at specified timepoints compared with baseline (in the subgroup of participants with PsO involving at least 3% BSA at baseline);
  - PASI100 at specified timepoints compared with baseline (in the subgroup of participants with PsO involving at least 3% BSA at baseline);
  - Response rate of at least 50% improvement compared with baseline in the Bath Ankylosing Spondylitis Disease Activity Index (BASDAI);

[redacted]

- The change from baseline at specified timepoints for the following:
  - TJC68;
  - SJC66;
  - Enthesitis evaluation (LEI and Spondyloarthritis Research Consortium of Canada [SPARCC] enthesitis index);
  - Leeds Dactylitis Index (LDI);
  - Modified Nail Psoriasis Severity Index (mNAPSI);
  - High sensitivity C-reactive protein (hs-CRP);
  - PtGADA;
  - Physician's Global Assessment of Disease Activity (PhGADA);
  - Psoriatic Arthritis Impact of Disease (PsAID)-12;
  - PtAAP;
  - HAQ-DI;
  - Functional Assessment of Chronic Illness Therapy for Fatigue (FACIT-Fatigue);
  - Short-Form-36 Health Survey Questionnaire-Version 2 (SF-36v2) Mental Component Summary (MCS);
  - SF-36v2 PCS;
  - BASDAI;
  - Patient Health Questionnaire-9 (PHQ-9).

[redacted]

7.2.4 Pharmacokinetic and Immunogencity Endpoints

- PK of sonelokimab (trough levels);
- ADAs.

[redacted]

7.2.6 Safety Endpoints

The following safety endpoints will be assessed:

- Incidence, relatedness, severity, and seriousness of AEs;
- Withdrawal due to AEs;
- Clinically relevant abnormalities in vital signs (blood pressure [BP] and heart rate) and body weight;
- Standard 12-lead electrocardiogram (ECG) intervals (RR, PR, QRS, QT, and QT intervals corrected for heart rate using Bazett's and Fridericia's formulas [QTcB and QTcF, respectively]), including clinically relevant abnormalities in ECG variables;
- Clinically relevant abnormalities in clinical laboratory variables (hematology, biochemistry, and urinalysis).

8 OVERALL STUDY DESIGN AND PLAN

8.1 Overview of the Study Design

This is a Phase 2 multi-center, randomized, parallel-group, double-blind, placebo-controlled, 2-part study evaluating the efficacy, safety, PK, and immunogenicity of sonelokimab in participants with active PsA (Figure 1). The study includes adalimumab treatment as an active reference arm; however, no formal comparison of sonelokimab vs adalimumab is planned.

The study will include screening period of up to 28 days, a treatment period of up to 24 weeks, and a safety follow-up period of 8 weeks after the last dose of study treatment. The treatment period will be divided into 2 parts:

- Part A: a 12-week, randomized, parallel-group, double-blind, placebo-controlled period;
- Part B: a 12-week, crossover, parallel-group, double-blind period.

On the first day of Part A (Day 1/Week 0), eligible participants will be randomized 1:1:1:1:1 to one of 5 treatment arms as described in Figure 1 and Table 2.

Randomization will be stratified by sex (Male/Female) and exposure to biologic agents prior to the Screening Visit (Yes/No). The total number of participants with previous use of biologic agents will be capped at 30%.

Dosing in sonelokimab arms will continue through Week 8. Adalimumab will be given through Week 10. All treatment arms will receive placebo as required to maintain the blinding of the study. Part A will end at Week 12 when the primary efficacy analysis will be performed, comparing each of the sonelokimab treatment arms (sonelokimab 120 mg Q2W, sonelokimab 60 mg Q2W, sonelokimab 60 mg Q4W) vs placebo.

At the beginning of Part B (Week 12) TJC68 and SJC66 response will be assessed in all participants. A responder is defined as a participant who achieves at least a 20% reduction in each of the TJC68 and SJC66 assessments at Week 12 compared with baseline. A non-responder is defined as a participant who does not achieve at least a 20% reduction in each of the TJC68 and SJC66 assessments at Week 12 compared with baseline.

In Part B participants will be allocated to treatment based upon response at Week 12 via the Interactive Response Technology (IRT). Treatment assignments in Part A and the allocation for Part B by responders and non-responders at Week 12 are described in Figure 1 and Table 3.

The last dose of study treatment is scheduled to occur at Week 22. An End-of-Treatment (EOT) Visit will be performed at Week 24. A Safety Follow-up Visit will be performed 8 weeks (±7 days) after the administration of the last dose of study treatment.

On an ongoing basis, safety data will be reviewed by an independent DSMB (Section 11.3.12).

Figure 1 Study Design

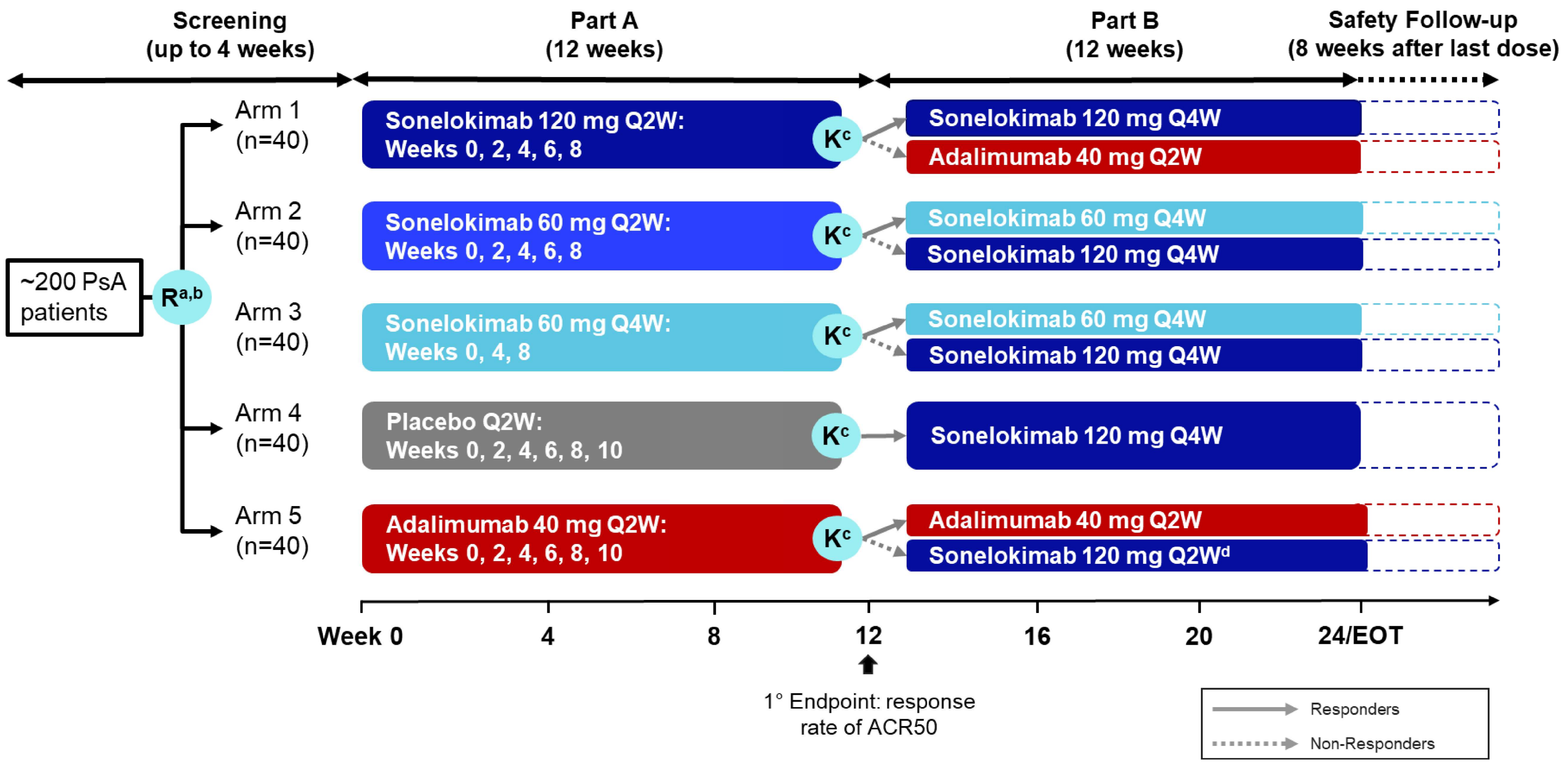

Abbreviations: ACR50 = response rate of at least 50% improvement in the American College of Rheumatology criteria; EOT = End-of-Treatment; IRT = Interactive Response Technology; K = crossover; PBO = placebo; PsA = psoriatic arthritis; Q2W = once every 2 weeks; Q4W = once every 4 weeks; R = randomization; SC = subcutaneous; SJC = swollen joint count; TJC = tender joint count.

a Randomization stratified by sex (Male/Female) and prior exposure to biologic agents (Yes/No).

b At the beginning of the treatment period at Week 0/Day 1, all eligible participants will be randomized 1:1:1:1:1.

c A responder is defined as a participant who achieves at least a 20% reduction in each of the TJC68 and SJC66 assessments at Week 12 compared with baseline. A non-responder is defined as a participant who does not achieve at least a 20% reduction in each of the TJC68 and SJC66 assessments at Week 12 compared with baseline. Treatment assignments in Part B will be allocated via the IRT based upon response at Week 12 as indicated in the study design diagram.

d In Part B non-responders in Arm 5 will receive sonelokimab 120 mg Q2W with SC injections at Weeks 12, 14, 16, 18, and 20.

8.2 Start of Study Definition

The start of the study is defined as the date the first participant provides informed consent.

8.3 Visits

Participants are to adhere to the visit schedule as described in the SOA in Section 3, Table 1. Each visit date (with its window) is to be counted from Day 1.

8.3.1 Screening Visit

At the Screening Visit, each participant will first provide informed consent. A participant is considered to be enrolled in the study immediately after they provide informed consent. Enrolled participant should be immediately registered as screened in the IRT system.

The participant will then be assigned a unique participant number via the IRT system, be assessed for eligibility with the inclusion and exclusion criteria, and perform study activities as described in the SOA in Section 3, Table 1.

Screening begins immediately after the participant provides informed consent and must be completed with 28 days; i.e., all results of assessments, including laboratory results, must be available to evaluate a participant's eligibility against the inclusion criteria (Section 9.1.1) and exclusion criteria (Section 9.1.2) within 28 days of signing informed consent.

A participant who does not fulfill the criteria for participation in this study is considered a screen failure (Section 9.2) and is to be registered as such with the IRT. Screen failures are not to be re-screened. An initial laboratory result obtained at the Screening Visit may be retested one time prior to the randomization after consultation with the medical monitor. The result of the second test will then be used to assess a participant's eligibility as indicated in Section 9.1.

8.3.2 Randomization and Visits During the Treatment Period

After eligibility is confirmed, randomization via IRT will be performed at the Day 1/Week 0 Visit as described in Section 10.1. Once randomized treatment is assigned to a participant, the study activities as described in the SOA in Section 3, Table 1 are to be performed. The treatment period is planned to continue for 24 weeks.

At each scheduled study visit, study procedures and activities are to be performed as listed in the SOA in Section 3, Table 1.

The following patient reported outcomes (PRO) assessments are to be completed by the participant on their own in a quiet area/room before blood draws and other visit assessments, and, if possible, before any interaction with the investigator or other members of the study team. The recommended order of completion for PROs will be as follows, at relevant visits according to the SOA in Section 3, Table 1:

[redacted]

[redacted]

Disease assessments performed by the investigator or a qualified designee are as follows:

1. TJC68 and SJC66;
2. PhGADA;
3. PsO-affected BSA;
4. PASI;
5. LDI;
6. Enthesitis evaluation (LEI and SPARCC enthesitis index);
7. mNAPSI.

Investigators will collect clinical assessments and record data in an electronic clinical outcome assessment (eCOA) system (Section 15.1). Calculations where required will be performed by the system.

Measurement of vital signs, physical examinations, evaluation of AEs, review of concomitant medications, and all blood draws will also be performed.

[redacted]

Composite disease assessments ACR20/50/70, [redacted] minimal disease activity, [redacted] used in this study are automatically calculated and do not need to be calculated manually by the investigator or study personnel.

8.3.3 Week 12 Visit

Assessments at Week 12 are both the final assessments for Part A and the first assessments for Part B. The dose administered at Week 12 is the first dose for Part B. The last dose administration is scheduled for Week 22.

At Week 12 (at the beginning of Part B) TJC68 and SJC66 response will be assessed in all participants. A responder is defined as a participant who achieves at least a 20% reduction in each of TJC68 and SJC66 assessments at Week 12 compared with baseline. A non-responder is defined as a participant who does not achieve at least a 20% reduction in each of TJC68 and SJC66 assessments at Week 12 compared with baseline.

Treatment assignments in Part B will be allocated based upon response at Week 12 via the IRT as indicated in Figure 1 and described in Section 10.1.

In the event a participant does not attend Week 12, please refer to Rescheduling Visits in Section 8.6.

8.3.4 End-of-Treatment Visit

An EOT Visit will be performed at Week 24 for a participant who completes the study. If a participant discontinues prematurely, the EOT will be performed at the time of treatment discontinuation. Please refer to Section 9.4 for Treatment Discontinuation.

The activities to be performed at the EOT are listed in the SOA in Section 3, Table 1.

8.3.5 Safety Follow-up Visit

Participants are to attend a Safety Follow-up Visit 8 weeks (±7 days) after the administration of the last dose of study treatment.

If the participant no longer wants to take part in the study (i.e., they prematurely discontinue the study and/or withdraw consent), they will attend the EOT Visit as described in the SOA in Section 3, Table 1, and be discontinued from the study. Every reasonable effort will be made to have discontinued participants attend the Safety Follow-up Visit.

Note this exception to scheduling the Safety Follow-up Visit: Participants who decide to discontinue the treatment in Part A should continue to attend all future scheduled visits through Week 12 for safety and efficacy assessments (as described in Section 9.4). A separate Safety Follow-up Visit is not required in this case if the participant attends a scheduled visit at 8 weeks after treatment discontinuation.

8.4 End of Study Definition

A participant is considered to have completed the study if they have completed all parts of the study, including the post-treatment safety follow-up period, which concludes with the Safety Follow-up Visit 8 weeks (±7 days) after the administration of the last dose of study treatment.

The end of the study is defined as the date of the last visit for the last participant in the study globally.

8.5 Study Treatment After the End of Study

No additional study treatment will be available after completion of the study or early discontinuation. After study completion, or after completion of the EOT Visit, participants are to be treated as per the local standard of care.

8.6 Rescheduling Visits

If any visit has to be rescheduled, subsequent visits will follow the original visit date schedule. Visits can take place on the scheduled day ±2 days. If a visit is unavoidably delayed outside of this window, it may be rescheduled up to Day +7 after approval by the medical monitor.

The activities performed at the rescheduled visit are to be those activities scheduled for the visit that was delayed, including the administration of any study treatment, as applicable

If a visit cannot take place by Day +7 then the visit should be skipped and the dose from the missed visit will not be administered. The participant is to proceed to the next scheduled visit. For further information on missed doses, see Section 10.4.

If the Week 12 visit does not take place, the responder/non-responder assessment should be performed at Week 14 and entered into the IRT. The patient will be assigned to the correct treatment arm according to responder/non-responder status and allocated corresponding Week 14 treatment. The Week 12 dose will be recorded as missed.

Doses of study treatment are to be administered at least 7 days apart. Thus, care will be taken to avoid scheduling a visit within 7 days of the previous or next scheduled dose.

Additional unscheduled visits for the purpose of re-testing of laboratory parameters or safety monitoring may be included as deemed necessary by the investigator.

8.7 Unscheduled Visits

An unscheduled visit is defined as any visit to the investigator site outside of the protocol-specified timepoints due to safety reasons or when a repeated measurement is required (e.g., obvious measurement errors, confirmation of out-of-range results), where the participant is seen by study personnel.

All unscheduled visits and assessments performed during the visits will be recorded in the participant's electronic case report form (eCRF). During any unscheduled visits the investigator

or qualified designee will record any AE and concomitant medications as well as performing any assessments or collecting samples deemed necessary at the discretion of the investigator.

8.8 Protocol Deviations

All deviations from planned study procedures are to be recorded. Protocol deviations will be reviewed as part of the ongoing data cleaning process.

Prospective approval of protocol deviations, also known as protocol waivers or exemptions, are not permitted.

If there are restrictions to the clinical study as a result of the COVID-19 pandemic, it may be necessary to adjust the visit schedule, convert in-person visits to telephone contacts, and postpone study procedures until the next available study visit. All temporary mechanisms utilized, and deviations from planned study procedures in response to COVID-19 are to be documented as being related to COVID-19 and will remain in effect only for the duration of the public health emergency.

8.9 Rationale for the Study Design and Selection of Dose

This current PsA Phase 2 study will be a randomized, parallel-group, double-blind, placebo-controlled study to evaluate the efficacy and safety of 3 dose regimens of sonelokimab in participants with active PsA.

ACR50 response rate at Week 12 compared with baseline will be the primary endpoint in this study. While ACR20 has historically been the primary endpoint in most PsA clinical programs over the past years, there is precedent in more recent studies (e.g., bimekizumab's BE OPTIMAL and BE COMPLETE) to target a higher ACR50 endpoint [Ritchlin C.T. et al., 2020]. This is driven by the established principle that, across inflammatory diseases, patient benefit correlates with higher levels of measured clinical improvement [Houghton K. et al., 2021]. Notably, in PsA, an inflammatory phase during the course of the disease is often followed by a phase with irreversible tissue destruction demanding therapies with an optimal ("as high as possible") anti-inflammatory effect to potentially provide disease-modifying effects [Eyerich K. et al., 2021].

The primary endpoint will be measured at Week 12 and participants randomized to the placebo group will receive placebo dosing until this timepoint.

Studies with other IL-17 agents indicate that improvement in disease activity may be seen after 12 weeks of treatment. For example, in a Phase 2 randomized, double-blind, placebo-controlled study of bimekizumab for treatment of PsA (NCT02969525), after 12 weeks of Q4W administration, significantly more patients in the active treatment groups of bimekizumab achieved an ACR50 response compared with the placebo group: bimekizumab 16 mg (odds ratio [OR] 4.2 [95% CI 1.1 to 15.2]; $p = 0.032$), bimekizumab 160 mg (8.1 [2.3 to 28.7]; $p = 0.0012$), and loading dose of bimekizumab 160 mg (9.7 [2.7 to 34.3]; $p = 0.0004$) [Ritchlin C.T. et al., 2020].

Sonelokimab has not been tested before this clinical study in a population of patients with PsA. This study will evaluate sonelokimab dose regimens within the range shown to be safe and well tolerated in Phase 1 studies and which have demonstrated efficacy in patients with PsO.

In a Phase 1 PsO study of sonelokimab (NCT02156466) in 44 patients with cutaneous PsO, a significant improvement in PASI100 score in all 4 cohorts of subjects who received 30 mg, 60 mg, 120 mg, or 240 mg sonelokimab was observed, compared with the placebo group. All doses were well tolerated. PASI100 response rates between arms indicated a dose-response relationship up to the 120 mg dose. PASI100 rates between the 120 mg (50.0%) and 240 mg (55.6%) arms did not substantially differ and were both significantly higher than placebo (0%).

In a subsequent Phase 2 PsO study of sonelokimab (NCT03384745) in 313 patients with cutaneous PsO, sonelokimab 120 mg demonstrated a greater clinical response (PASI100) at Week 24 compared with sonelokimab 60 mg, with no unexpected safety findings observed.

Based on clinical programs of IL-17 inhibitors in PsO and PsA, it is hypothesized that clinical efficacy (assessed by the proportion of participants achieving ACR response criteria) may be obtained with a lower dose level in PsA as opposed to PsO [Baraliakos X. et al., 2017; McInnes I.B. et al., 2015; Mease P.J. et al., 2017; Ritchlin C.T. et al., 2020]. For example, in a Phase 3 PsA trial (FUTURE 2) in 397 patients, secukinumab 300 mg (PsO optimal dose) achieved 54% ACR20 response at Week 24, which was not substantially different to the 51% ACR20 response rate with secukinumab 150 mg [McInnes I.B. et al., 2015]. The approved dose of secukinumab is 300 mg Q4W for PsO vs 150 mg Q4W for PsA patients (with no prior anti-TNF therapy).

This study will test sonelokimab at 3 dose regimens for the initial 12 weeks: sonelokimab 120 mg Q2W, sonelokimab 60 mg Q2W, and sonelokimab 60 mg Q4W. These doses are expected to enable preliminary efficacy in PsA to be evaluated and to allow evaluation of an induction regime vs no induction regime approach. From Week 12 onward, the ability of sonelokimab Q4W to maintain response in those participants achieving at least a 20% improvement in their baseline tender and swollen joint count will be evaluated.

The study also has an active reference arm of adalimumab treatment, an approved anti-TNFα drug; however, no formal comparison of sonelokimab vs adalimumab is planned. Alongside the placebo arm, the adalimumab reference arm will be used to calibrate this study's patient population as compared to other clinical programs. After Week 12, participants receiving adalimumab not achieving at least a 20% improvement in baseline tender and swollen joint count will switch to sonelokimab 120 mg Q2W. This change in study treatment will allow initial assessment of the response to sonelokimab in anti-TNFα non-responders.

Inclusion and exclusion criteria have been developed to ensure a well-defined population where efficacy can be determined without introducing any unacceptable risks or confounding effects. The study design incorporates frequent visits and evaluations to optimize assessment of endpoints and collection of safety data.

9 STUDY POPULATION

9.1 Eligibility Criteria

9.1.1 Inclusion Criteria

To receive randomized treatment assignment, participants must fulfill the following inclusion criteria at the Screening Visit and prior to the initiation of study treatment, unless another time is specified:

1. Participant is ≥18 years of age;
2. Participant has a confirmed diagnosis of PsA per the 2006 CASPAR with symptoms for ≥6 months prior to the Screening Visit (see Appendix 1 in Section 21.1);

   Note: New X-ray images of hands and/or feet are not required. Results of the most recent X-ray images of hands and/or feet will be captured in the eCRF. If no previous images exist, X-rays may be obtained as part of routine management at the discretion of the investigator.
3. Participant has active disease (defined by a TJC68 of ≥3 and a SJC66 of ≥3);
4. Participant has either current active PsO or a dermatologist confirmed history of PsO;
5. Participant tests negative for rheumatoid factor (RF) at the Screening Visit;
6. Participant tests negative for anti-cyclic citrullinated peptide (CCP) antibodies at the Screening Visit;
7. Participant must be, in the opinion of the investigator, a suitable candidate for treatment with adalimumab per approved local product information. If a chest X-ray or computerized tomography (CT) scan for TB screening is required per local guidance, the X-ray or CT scan must be taken within 3 months prior to the Screening Visit;
8. Participant has had an inadequate response at the Screening Visit (lack of efficacy after ≥12-week duration of therapy) to previous or current treatment with ≥1 non-biologic DMARD at maximally tolerated dose, or participant has an intolerance to or contraindication for DMARDs as defined by the investigator;
9. If the participant is female, she must be of non-childbearing potential or, if of childbearing potential, participant must agree to use highly effective methods of contraception. See Appendix 2 in Section 21.2 for the definition of non-childbearing potential, childbearing potential, and highly effective methods of contraception;
10. Women of childbearing potential (as defined in Appendix 2 in Section 21.2) must have a negative serum human chorionic gonadotropin (hCG) pregnancy test at the Screening Visit and a negative urine pregnancy test at Week 0/Day 1 prior to the first administration of study treatment;

11. If male, participant must be willing to use a condom when sexually active with a partner of childbearing potential (as defined in Appendix 2 in Section 21.2) during the study and for 12 weeks after the last dose of study treatment, unless surgically sterile;
12. Participant is considered reliable and capable of adhering to the protocol, visit schedule, or medication intake according to the judgment of the investigator;
13. Participant is able to understand and provide signed informed consent.

9.1.2 Exclusion Criteria

Participants who fulfill any of the following exclusion criteria at the Screening Visit or Day 1/Week 0 prior to the initiation of study treatment, unless another time is specified, must be excluded from receiving randomized treatment:

1. Participant with known hypersensitivity to sonelokimab or any of its excipients;
2. Participant with known hypersensitivity to adalimumab or any of its excipients;
3. Participant who currently uses or plans to use one or more prohibited treatments specified in this protocol. Prohibited treatments and washout periods are provided in Section 10.5;
4. Participant who has previously failed on anti-IL-17 therapy, defined as inadequate clinical response, according to the investigator’s judgment, after at least 16 weeks of treatment; or is unsuitable for anti-IL-17 therapy for any other reason according to the investigator’s discretion;
5. Participant who has previously failed on anti-TNFα therapy, defined as inadequate clinical response, according to the investigator’s judgment, after at least 16 weeks of treatment; or is unsuitable for anti-TNFα therapy for any other reason according to the investigator’s discretion;
6. Participant who has had previous exposure to more than 2 biologic agents of any type to treat PsA prior to the Screening Visit, including but not limited to IL-17 inhibitors, IL-23 inhibitors, TNFα inhibitors, etc.

   Note: The total number of participants with previous use of biologic agents will be capped at 30% of the total participant population.
7. Participant who has a diagnosis of chronic inflammatory conditions other than PsO or PsA, including but not limited to rheumatoid arthritis, sarcoidosis, systemic lupus erythematosus, Crohn’s disease, or ulcerative colitis;
8. Participant who has a diagnosis of arthritis mutilans;

9. Participant who has an active infection or history of infections, including any of the following:
    a. Any infection (exception: common cold) requiring systemic treatment within 14 days before initiation of study treatment;
    b. Serious infection, defined as infection requiring hospitalization or intravenous anti-infective, within 2 months before initiation of study treatment;
    c. History of opportunistic infections caused by uncommon pathogens (e.g., *Pneumocystis jirovicii*, Blastomyces, aspergillus, cryptococcosis), or severe infections caused by common pathogens (e.g., cytomegalovirus, severe herpes zoster e.g. multidermatomal herpes zoster, herpes zoster with organ involvement, ophthalmic herpes, or recurrent herpes zoster [recurrent is defined as 2 episodes within 2 years prior to study treatment initiation]);
    d. History of other opportunistic, recurrent, or chronic infections that, in the opinion of the investigator, might cause study participation to be detrimental to the participant;
    e. *Candida* infection requiring systemic therapy for ≥7 days in the last 12 months prior to study treatment initiation;
    f. Any history of esophageal or systemic candidiasis;
    g. Current active candidiasis or *Candida* infection within the last 1 month prior to the Screening Visit;
    h. Concurrent acute or chronic viral hepatitis B or C, or human immunodeficiency virus (HIV);
    i. Confirmed SARS-CoV-2 infection at the Screening Visit;

    Note: A participant with an initial positive SARS-CoV-2 result may enter the study provided they are asymptomatic and a negative test (polymerase chain reaction [PCR] or antigen) has been provided prior to study treatment initiation.
10. Participant who received a live (including attenuated) vaccination within 8 weeks before study treatment initiation, or planned to receive a live vaccination during the study and up to at least 12 weeks after the last dose of study treatment. Examples of restricted vaccinations include, but are not limited to:
    a. Zoster vaccine live (Zostavax);
    b. Measles-mumps-rubella or measles-mumps-rubella-varicella;
    c. Monovalent live attenuated influenza A (intranasal);
    d. Oral polio;
    e. Rotavirus;
    f. Seasonal trivalent live attenuated influenza (intranasal);

g. Smallpox;

h. Oral typhoid;

i. Varicella (chicken pox);

j. Yellow fever.

Note: See Appendix 3 in Section 21.3 for instructions regarding vaccinations to SARS-CoV-2.

11. Participant who received a *Bacillus* Calmette-Guérin (BCG) vaccination within 1 year before study treatment initiation;

12. Participant with:

    a. A history of active TB, i.e., participants having received combination treatment for active TB;

    b. Evidence of TB infection as defined by a positive QuantiFERON® TB-Gold test (or interferon-gamma release assay [IGRA] equivalent) at screening, unless the following criteria apply:

        i. A full TB work-up (according to local practice/guidelines) completed within 12 weeks prior to randomization establishes conclusively that the participant has no evidence of active or latent TB;

        ii. Participants positive for latent TB per work-up must have completed sufficient treatment according to local routine clinical practice at least 4 weeks before randomization.

    Note 1: If a QuantiFERON TB-Gold test (or IGRA equivalent) at screening is indeterminate, it can be repeated once. The results of the second test are to be used. If the second QuantiFERON TB-Gold test is also indeterminate, the participant should be managed as for participants with positive QuantiFERON TB-Gold test (or IGRA equivalent);

    Note 2: If there is reasonable medical doubt about a potentially false-positive QuantiFERON TB Gold test (or IGRA equivalent) result, the test can be repeated once after obtaining Medical Monitor approval, and the results of the second test will prevail.

13. Participant with any current nontuberculous mycobacterial (NTM) infection or any history of pulmonary NTM infection at the Screening Visit;

14. Participant with evidence of acute ocular inflammation, including active anterior uveitis (i.e., acute episode), within the last 4 weeks before study treatment initiation;

15. Participant with a concurrent malignancy or a history of malignancy during the past 5 years of the Screening Visit, with the following exceptions:

    a. ≤3 excised or ablated basal cell carcinomas of the skin;

b. One squamous cell carcinoma of the skin not worse than Stage T1 that has been successfully excised or ablated (no other previous treatments allowed), with no signs of recurrence or metastases for at least the past 2 years before study treatment initiation;

c. Actinic keratosis;

d. Squamous cell carcinoma in situ of the skin successfully excised or ablated at >6 months before study treatment initiation;

e. Localized carcinoma in situ of the cervix, treated and considered cured.

16. Participant with fibromyalgia, osteoarthritis symptoms, or any other condition that in the investigator's opinion may potentially interfere with efficacy assessments;

17. Participant with erythrodermic, guttate, or pustular form of PsO or drug-induced PsO;

18. Participant with a history of a lymphoproliferative disorders, including lymphoma, or current signs and symptoms suggestive of lymphoproliferative disease;

19. Participant with primary immunodeficiencies, prior splenectomy, or suppressive conditions, including participants taking immunosuppressive therapy following organ transplants;

20. Participant who had major surgery (including joint surgery) within 6 months before the Screening Visit, or is planning to have major surgery during the study;

21. Participant with the presence of active suicidal ideation, or positive suicidal behavior at the Screening Visit, as evidenced by the C-SSRS assessment that shows any history of suicidal attempt (including an actual attempt, interrupted attempt, or aborted attempt), or suicidal ideation in the past 6 months as indicated by a positive response to either Question 4 or 5 of the C-SSRS at screening;

22. Participant has presence of moderately severe depression or severe depression, indicated by a score of ≥15 using the screening PHQ-9. Participants are permitted to use 1 medication to treat depression provided dose is stable for 4 weeks prior to initiation of study treatment. Participants on multiple medications for depression are excluded from the study;

23. Participant with severe cardiovascular comorbidities including history of myocardial infarction, unstable angina pectoris, stroke, heart failure (New York Heart Association [NYHA] classification III or IV), or uncontrolled hypertension (characterized by 2 BP measurements separated by at least 15 minutes with systolic BP >160 mmHg or diastolic BP >100 mmHg);

24. Participant with clinically significant ECG abnormalities on centrally read ECG at the Screening Visit;

25. Participant with any other clinically significant medical conditions or any other reason, including any physical, psychological, or psychiatric condition, that in the opinion of the investigator would compromise the safety or interfere with participation in the study, would

make the participant an unsuitable candidate to receive study treatment, or would put the participant at risk;

26. Participant with laboratory abnormalities at the Screening Visit, including any of the following:
    a. Aspartate aminotransferase (AST), alanine aminotransferase (ALT), or alkaline phosphatase >3 times the upper limit of normal (ULN);
    b. Serum direct bilirubin >1.5 × ULN (in the absence of known Gilbert's syndrome);
    c. White blood cell count <3.0 × 10^9/L;
    d. Absolute neutrophil count <1.5 × 10^9/L;
    e. Absolute lymphocyte count <0.8 × 10^9/L;
    f. Platelet count <100 × 10^9/L;
    g. Hemoglobin <85 g/L;
    h. Creatinine clearance <60 mL/min (by Cockcroft Gault formula);
    i. Any other laboratory abnormality which, in the opinion of the investigator, might compromise the participant's safety, might prevent the participant from completing the study, or might interfere with the interpretation of the study results.

    Note: An initial laboratory result obtained at the Screening Visit may be retested one time prior to the initiation of study treatment after consultation with the medical monitor. The result of the second test will then be used.
27. Participant is enrolled in another interventional investigational device or drug study, or has been in another investigational study treatment in the last 28 days prior to the Screening Visit or within 5 half-lives of the other investigational device or study treatment prior to the Screening Visit, whichever is greater;
28. Participant is pregnant or breastfeeding, or plans to become pregnant while enrolled in the study and up to 12 weeks after the last dose of study treatment;
29. Participant has a history of chronic alcohol or drug abuse in the past year prior to the Screening Visit;
30. Participant is an employee, or direct relative of an employee, of the sponsor, at a study site, or of a third-party organization involved in the study.

9.2 Screen Failures

Screen failures are defined as participants who consent to participate in the clinical study but are not subsequently assigned randomized study treatment. A minimal set of screen failure information is required to ensure transparent reporting of screen failure participants to meet the Consolidated

Standards of Reporting Trials (CONSORT) publishing requirements and to respond to queries from regulatory authorities. Minimal information includes demography, screen failure details, eligibility criteria, and any SAE.

Clinical laboratories performed at the Screening Visit may be repeated once if they are outside the acceptable range indicated in the exclusion criteria values (Section 9.1). The second value will be used to assess eligibility provided it is received within 28 days of the participant signing the informed consent form (ICF). If the second laboratory value is not received within 28 days of the participant signing the ICF, the participant is considered a screen failure.

Participants who do not fulfill the criteria for participation in this study are not to be re-screened.

9.3 Strategies for Recruitment and Retention

All recruitment material will be approved by an Independent Ethics Committee (IEC) or Institutional Review Board (IRB) prior to implementation.

Regular study monitoring will enable identification of any potential issues related to participant retention.

9.4 Study Treatment Discontinuation

The investigator should discontinue study treatment of a given participant if, on balance, they believe that continuation would be detrimental to the participant's well-being or if the participant plans to initiate any therapies outlined in the prohibited medications list in Section 10.5.1. EOT procedures should be performed at the point when the decision to discontinue the treatment has been made. A Safety Follow-up Visit should be performed 8 weeks (±7 days) after their last dose of study treatment.

In addition to the EOT Visit, participants who decide to discontinue the treatment in Part A should continue to attend all future scheduled visits through Week 12 for safety and efficacy assessments. A separate Safety Follow-up Visit is not required if the participant attends a scheduled visit at 8 weeks after treatment discontinuation.

If the patient discontinues treatment in Part B, EOT procedures should be performed at the point when the decision to discontinue the treatment has been made. A Safety Follow-up Visit should be performed 8 weeks (±7 days) after their last dose of study treatment.

In case the participant does not agree to continue with all scheduled visits, the participant will be withdrawn from the study.

9.4.1 Individual Participant Study Treatment Stopping Rules

Study treatment must be discontinued under the following circumstances and further steps need to be discussed with the medical monitor:

1. New diagnosis (gastroenterologist confirmed) of Crohn's disease or ulcerative colitis (these events should be captured as AESIs; see Section 11.3.1.6).

2. Anaphylactic reaction or other severe systemic reaction to study treatment;
3. Diagnosis of malignancy during study;
4. Evidence of pregnancy;

[redacted]

- AE considered related to study treatment including:
    - Clinically significant systemic hypersensitivity reaction following administration of study treatment that does not respond to symptomatic treatment;
    - Severe (Grade 3 or higher Common Terminology Criteria for AEs [CTCAE] Version 5.0) anaphylaxis (symptomatic bronchospasm, with or without urticaria; parenteral intervention indicated; allergy-related edema/angioedema; hypotension);
    - Severe (Grade 3 or higher CTCAE Version 5.0) serum sickness (severe arthralgia or arthritis; extensive rash, with steroids or intravenous fluids indicated);
    - *Note*: If a participant experiences symptoms consistent with a Grade 2 serum sickness (moderate arthralgia, fever, rash, or urticaria, with antihistamine treatment indicated), and causality by the study treatment cannot be ruled out, the participant will be asked to return to site for an unscheduled visit. There is to be a documented assessment and physical examination concurrent with the event. Grade 2 serum sickness does not require discontinuation of study treatment, however the medical monitor will be informed;
- Any other severe (Grade 3 CTCAE Version 5.0) event that does not resolve with supportive care;

   - Any life-threatening (Grade 4 CTCAE Version 5.0) event.

7. Renal function values:
   - Discontinue study treatment for a participant if individual serum creatinine increases ≥50% compared to the value at the Screening Visit (and is considered clinically significant), or in the event of treatment-emergent proteinuria (albumin:creatinine ratio >300 mg/g or >30 mg/mmol; protein:creatinine ratio ≥500 mg/g or >50 mg/mmol), unless the event is not drug-related, or if the risk/benefit assessment supports continuing study treatment;

   Note: A renal event leading to participant discontinuation is to be followed until event resolution (serum creatinine within 10% of baseline, protein-creatinine ratio within 50% of baseline), stabilizes or becomes not clinically significant, or is assessed as being chronic.

8. Liver laboratory values:
   - Confirmed ALT or AST >8 × ULN; or
   - Confirmed ALT or AST >5 × ULN (for more than 2 weeks); or
   - Elevated transaminases (ALT or AST) >3 × ULN and associated with total bilirubin >2 × ULN (without accompanying alkaline phosphatase elevation >2 × ULN suggestive of cholestasis) – i.e., participant meets Hy's Law criteria; or
   - Confirmed ALT or AST >3 × ULN with the appearance of fatigue, nausea, vomiting, right upper quadrant pain or tenderness, fever, rash, and/or eosinophilia (>5%).

9. Active TB infection;

10. Any other protocol deviation that results in a significant risk to the participant's safety.

11. Participant noncompliance (significant violation of protocol requirements or missing 2 consecutive visits).

If a clinically significant laboratory abnormality of Grade 3 or higher is considered causally related to study treatment, study treatment will be permanently discontinued and the event must be reported as an AE. In cases in which a causal relationship to study treatment can be reasonably excluded, (i.e., an alternative cause is evident), study treatment will be postponed but it may be resumed when the laboratory abnormality is sufficiently normalized (Section 10.4). A decision to resume study treatment will be made jointly by the investigator and medical monitor (medical monitor's approval is required).

In addition to these requirements for study treatment discontinuation, the investigator will discontinue study treatment for a given participant if, on balance, continuation would be detrimental to the participant's well-being.

[redacted]

9.5 Safety Follow-up After Treatment Discontinuation

The investigator must determine the primary reason for the participant's premature discontinuation of study treatment and record this information in the eCRF. The participant should be requested to attend the Safety Follow-up Visit.

9.6 Withdrawal of Participants

9.6.1 Withdrawal of Consent

Participants may voluntarily withdraw consent to participate in the study for any reason at any time.

Withdrawal of consent occurs when a participant does not want to participate in the study anymore and does not want to attend any further visits or assessments, have further study-related contact, or allow analysis of already obtained biologic material.

If a participant withdraws consent, all study participation for that participant will cease and data to be collected at subsequent visits will be considered missing. Study treatments must be discontinued and no further assessments conducted. For a participant who has withdrawn consent, the investigator should establish if consent is also withdrawn for analysis of any samples taken and not tested or for future use of samples where this was initially granted. Any withdrawal of consent to sample analysis must recorded in the study site records and be communicated to the study monitor. If the participant withdraws consent for disclosure of future information, the sponsor may retain and continue to use any data collected before such withdrawal of consent.

If a participant withdraws consent, the investigator must make every effort to determine the primary reason for this decision and record this information in the eCRF.

9.6.2 Non-Compliance

Participants will be deemed non-compliant if they significantly violate protocol requirements or in the event that 2 doses are missed in either Part A or Part B, or 2 consecutive visits are missed.

The EOT Visit and the Safety Follow-up Visit, as described in the SOA in Section 3, Table 1, will be conducted in all withdrawn participants willing to undergo the assessments. The EOT Visit will occur as soon as possible after study treatment withdrawal, while the Safety Follow-up Visit will occur 8 weeks (±7 days) after the administration of the last dose of study treatment.

The investigator must also contact the IRT to register the participant's discontinuation from study treatment.

9.7 Lost to Follow-up

All reasonable efforts must be made to locate participants to determine and report their ongoing status. This includes follow-up with persons authorized by the participant. Lost to follow-up is defined by the inability to reach the participant after a minimum of 3 documented contacts (e.g., phone calls, text messages, faxes, or emails) not performed on the same day. All attempts will be documented in the participant's medical records.

9.8 Discontinuation of Study Sites

Study site participation may be discontinued if the sponsor or designee, the investigator or IEC/IRB of the study site judges it necessary for medical or safety reasons consistent with applicable laws, regulations, and Good Clinical Practice (GCP).

9.9 Discontinuation of Study

The study will be discontinued if the sponsor or designee judges it necessary for medical, safety, regulatory, or business reasons consistent with applicable laws, regulation, and GCP.

10 STUDY TREATMENT

Study treatment is defined as any investigational intervention(s), marketed product(s), or placebo intended to be administered to the study participant according to the study protocol.

Study treatments must not be used for any purpose other than as described in this protocol.

Only participants enrolled in the study may receive study treatment and only authorized site staff may prepare, supply, or administer study treatments.

10.1 Administration of Study Treatment(s)

On the first day of Part A (Day 1/Week 0), eligible participants will be randomized 1:1:1:1:1 to one of 5 treatment arms as describe in Table 2:

Table 2 Randomized Treatment Assignment in Part A

Treatment Assignment in Part A
Arm 1: Sonelokimab 120 mg Q2W
Arm 2: Sonelokimab 60 mg Q2W
Arm 3: Sonelokimab 60 mg Q4W
Arm 4: Placebo
Arm 5: Adalimumab 40 mg Q2W

Abbreviations: Q2W = once every 2 weeks; Q4W = once every 4 weeks

Randomization will be stratified by sex (Male/Female) and exposure to biologic agents prior to the Screening Visit (Yes/No). The total number of participants with previous use of biologic agents will be capped at 30%.

Dosing in sonelokimab arms will continue through Week 8. Adalimumab will be given through Week 10. All treatment arms will receive placebo as required to maintain the blinding of the study. Part A will end at Week 12 when the primary efficacy analysis will be performed, comparing each of the sonelokimab treatment arms (sonelokimab 120 mg Q2W, sonelokimab 60 mg Q2W, sonelokimab 60 mg Q4W) vs placebo.

At the beginning of Part B (Week 12) TJC68 and SJC66 response will be assessed in all participants. A responder is defined as a participant who achieves at least a 20% reduction in each of the TJC68 and SJC66 assessments at Week 12 compared with baseline. A non-responder is defined as a participant who does not achieve at least a 20% reduction in each of the TJC68 and SJC66 assessments at Week 12 compared with baseline.

In Part B participants will be allocated to treatment based upon response at Week 12 via the IRT. Treatment assignments in Part A and the allocation for Part B by responders and non-responders at Week 12 are described in Table 3.

Table 3 Treatment Allocation in Part B Following Response Assessment at Week 12

Treatment Assignment in Part A	Treatment Allocation in Part B (Based Upon Response at Week 12)	
	**Responder**	**Non-responder**
Arm 1: Sonelokimab 120 mg Q2W	Sonelokimab 120 mg Q4W	Adalimumab 40 mg Q2W
Arm 2: Sonelokimab 60 mg Q2W	Sonelokimab 60 mg Q4W	Sonelokimab 120 mg Q4W
Arm 3: Sonelokimab 60 mg Q4W	Sonelokimab 60 mg Q4W	Sonelokimab 120 mg Q4W
Arm 4: Placebo	Sonelokimab 120 mg Q4W	Sonelokimab 120 mg Q4W
Arm 5: Adalimumab 40 mg Q2W	Adalimumab 40 mg Q2W	Sonelokimab 120 mg Q2W

Abbreviations: Q2W = once every 2 weeks; Q4W = once every 4 weeks

Treatments will be administered as described below and as scheduled in the SOA in Section 3, Table 1. Each visit date (with its window) is to be counted from Day 1. If any visit has to be rescheduled, subsequent visits will follow the original visit date schedule.

See Section 8.6 for a discussion of rescheduling visits and Section 10.4 for a discussion of missed doses.

A detailed description of the composition of the drug product and placebo is shown in Table 4.

[redacted]

10.1.1 Sonelokimab

[redacted]

Sonelokimab will be administered by a site-approved, unblinded study treatment administrator. Blinded study personnel will not be in attendance while study treatment is being prepared or administered. Used syringes will be placed immediately out of sight in a sealed sharps container.

In Part A, the 3 sonelokimab treatment arms are as follows:

- Arm 1 (sonelokimab 120 mg Q2W): Sonelokimab 120 mg will be given as SC injection at Weeks 0, 2, 4, 6, and 8. Participants will receive a placebo injection at Week 10 to maintain the blind;

- Arm 2 (sonelokimab 60 mg Q2W): Sonelokimab 60 mg will be given as SC injection at Weeks 0, 2, 4, 6, and 8. Participants will receive a placebo injection at Week 10 to maintain the blind;
- Arm 3 (sonelokimab 60 mg Q4W): Sonelokimab 60 mg will be given as SC injection at Weeks 0, 4, and 8. Participants will receive a placebo injection at Weeks 2, 6, and 10 to maintain the blind.

In Part B, after allocation of responders and non-responders, the sonelokimab treatment arms are as follows:

- Responders from Arm 1, non-responders from Arm 2, non-responders from Arm 3, and participants from Arm 4 will receive sonelokimab 120 mg Q4W until Week 20. Placebo injections will be given at Weeks 14, 18, and 22 to maintain the blind;
- Responders from Arm 2 and responders from Arm 3 will receive sonelokimab 60 mg Q4W until Week 20. Placebo injections will be given at Weeks 14, 18, and 22 to maintain the blind;
- Non-responders in Arm 5 will receive sonelokimab 120 mg Q2W with SC injections at Weeks 12, 14, 16, 18, and 20. Participants will receive a placebo injection at Week 22 to maintain the blind.

10.1.2 Placebo

Placebo will be identical in appearance and packaging to sonelokimab in a fixed-dose, pre-filled syringe to be administered via SC injection.

As with the administration of sonelokimab, placebo will be administered by a site-approved, study treatment administrator, who is unblinded. Blinded study personnel will not be in attendance while study treatment is being prepared or administered. Used syringes will be placed immediately out of sight in a sealed sharps container.

During Part A for participants randomized to Arm 4, placebo will be given SC every 2 weeks, with injections at Week 0, 2, 4, 6, 8, and 10.

10.1.3 Adalimumab

Adalimumab will be provided in prefilled syringes (40 mg) as sterile solution for SC injection. Adalimumab syringes will be relabelled and repackaged to facilitate maintenance of the study blinding.

Adalimumab will be administered by a site-approved, study treatment administrator, who is unblinded. Blinded study personnel will not be in attendance while study treatment is being prepared or administered. Used syringes will be placed immediately out of sight in a sealed sharps container.

During Part A for participants randomized to Arm 5, adalimumab will be given as 40 mg SC every 2 weeks, with injections at Week 0, 2, 4, 6, 8, and 10 in accordance with the approved labeling.

In Part B, non-responders from Arm 1 and responders from Arm 5 will receive adalimumab 40 mg Q2W administered by SC injection at Weeks 12, 14, 16, 18, 20, and 22.

10.2 Study Treatment, Packaging, and Labeling

10.2.1 Packaging

Sonelokimab, placebo, and adalimumab will be provided in prefilled syringes packaged in individual tamper-sealed cartons. Study Treatments will be suitably packaged in such a way as to protect the product from deterioration during transport and storage. Further information regarding storage and transport conditions are provided in the pharmacy manual.

10.2.2 Labeling

Study treatment labels will comply with applicable Good Manufacturing Practices (GMP) and GCP and the legal requirements of each country. Labels will be printed in the local language. They will supply no information about the participants (other than the participant number).

[redacted]

10.3 Study Treatment Accountability

Site personnel will document the use of study treatment appropriately. Compliance for all treatment arms will be reviewed by the study monitor utilizing source documents, dispensing and inventory records, the Accountability Log, and study treatment eCRFs. Study treatment syringes will be disposed of immediately after administration in sealed sharps containers and cannot be checked. Accountability will be performed on the packaging cartons utilized for administration before destruction.

Records will be maintained of the delivery of study treatment(s) to the study center(s), the inventory at the study center(s), and the use of each carton.

These records shall include dates, quantities, appropriate manufacturing numbers, expiry dates, and the unique code numbers assigned to the study treatment and to the study participants. Further instructions for documenting study treatment records are provided in the pharmacy manual.

The investigator or qualified designee shall be responsible for ensuring that the records adequately document that the participants were provided the study treatment allocated by the IRT and that all study treatment received for the study is reconciled.

10.4 Missed Dose

Dose modifications are not permitted during the study.

A dose may be delayed if a visit has to be rescheduled. A dose that cannot be rescheduled within the permitted timeframe may be missed. See Section 8.6 regarding rescheduling visits.

If the investigator judges that at a particular visit the dose should be withheld for safety reasons (e.g., to allow for monitoring of an emerging AE) they should contact the medical monitor to discuss. All other study assessments should be completed per-protocol. A dose may be withheld/missed a maximum of once within Part A and once within Part B. See Section 9.6.2 regarding non-compliance.

10.5 Prior and Concomitant Medications

All medications taken from time of consent need to be recorded in the eCRF.

Any medication or vaccine (including over-the-counter or prescription medicines, cannabis/marijuana for medicinal use, vitamins, and/or herbal supplements) that the participant is receiving at the time of enrollment or receives during the study must be recorded along with the following:

- Reason for use;
- Dates of administration, including start and end dates;
- Dosage information, including dose and frequency.

Medications that were stopped prior to first dose of study treatment will be considered prior medications. Those that continue or start during the treatment period will be considered concomitant.

In addition, history of use of specific medications for the treatment of PsA will be captured as part of the PsA medical history (Section 11.1.1).

10.5.1 Prohibited Medications and Therapies

Combination treatment of any 2 or all of the following drugs: methotrexate, leflunomide, and sulfasalazine is prohibited during the study.

Medications prohibited during the study and the requisite washout period prior to initiating study treatment for prohibited medications are presented in Table 5.

Table 5 Prohibited Medications and Therapies and Washout Periods Prior to Initiating Study Treatment

Prohibited medication during study	Washout period to be considered before study treatment initiation
JAKi (e.g., tofacitinib, upadacitinib)	No prior exposure permitted
High-potency opioid analgesics including, but not limited to, methadone, hydromorphine, and morphine	2 weeks
Inhaled marijuana	2 weeks
Topical corticosteroids	2 weeks
Oral corticosteroids (except as allowed in permitted concomitant therapy, as described Section 10.5.2)	4 weeks
Intramuscular, intravenous, or intra-articular corticosteroids	4 weeks
Phototherapy	4 weeks
TNF inhibitors etanercept (or its biosimilars)	4 weeks
Live (including attenuated) vaccinations[a]	8 weeks
Methotrexate (unless requirements for permitted use of concomitant methotrexate are achieved)	12 weeks
Sulfasalazine (unless requirements for permitted use of concomitant sulfasalazine are achieved)	12 weeks
Any other non-biologic DMARDs (e.g., hydroxychloroquine, cyclosporine, etc.)	12 weeks
TNF inhibitors adalimumab, infliximab, golimumab or certolizumab (or their biosimilars)	12 weeks
Anti-IL-17 targeted therapy (participants with past exposure to sonelokimab are not eligible)	12 weeks
Any other biologic agent for PsA, including investigational products	12 weeks or 5 half-lives, whichever is longer
Systemic retinoids	3 months

Prohibited medication during study	Washout period to be considered before study treatment initiation
Intra-articular hyaluronic acid	6 months
Leflunomide (unless requirements for permitted use of concomitant leflunomide are achieved)	6 months (or 4 weeks with cholestyramine washout)

Abbreviations: DMARD = disease-modifying anti-rheumatic drug; IL = interleukin; JAKi = Janus kinase inhibitor; PsA = psoriatic arthritis; TNF = tumor necrosis factor

a See Appendix 3 in Section 21.3 for instructions regarding vaccinations against SARS-CoV-2.

10.5.2 Permitted Concomitant Therapy

All medications taken from time of consent are to be recorded in the eCRF.

The following medications are permitted if taken as defined below. The dose of any concomitant medications is to remain stable until the Safety Follow-up Visit:

- NSAIDs/COX-2 inhibitors, if taken at a stable dose for ≥2 weeks before study treatment initiation. No more than one NSAID/COX-2 may be taken at a given time;
- Use of analgesics (including e.g., paracetamol, low-potency opioids) is allowed if taken at a stable dose for ≥2 weeks before study treatment initiation;
- Oral corticosteroids (≤10 mg/day prednisone or equivalent), if taken at a stable dose for ≥2 weeks before study treatment initiation;
- Methotrexate (≤25 mg/week) is allowed if started ≥12 weeks before study treatment initiation and the dose has been stable for ≥8 weeks before study treatment initiation. Participants must not be taking methotrexate in combination with either leflunomide or sulfasalazine or both;
- Leflunomide (≤20 mg/day or an average of 20 mg/day if not dosed daily) is allowed if started ≥12 weeks before study treatment initiation and the dose has been stable for ≥8 weeks before study treatment initiation. Participants must not be taking leflunomide in combination with either methotrexate or sulfasalazine;
- Sulfasalazine (≤3 g/day) is allowed if started ≥12 weeks before study treatment initiation, with a stable dose for ≥8 weeks before study treatment initiation. Participants must not be taking sulfasalazine in combination with either methotrexate or leflunomide.

10.6 Lifestyle Considerations

General skin care measures standard for participants with plaque PsO (within the restrictions for topical treatments) are recommended. Participants should avoid excessive sun exposure and avoid risks that are known to provoke flare of PsO.

For the 48 hours prior to scheduled study visits, participants are to refrain from using a sauna, applying emollients to PsO plaques, or drinking alcohol excessively.

10.7 Blinding and Randomization of Study Treatment(s)

10.7.1 Part A

Randomization will take place on Day 1/Week 0 after confirmation that the participant continues to meet the inclusion/exclusion criteria. Participants will be randomized 1:1:1:1:1 to one of 5 treatment arms, stratified by sex and exposure to biologic agents prior to the Screening Visit (Figure 1 and Table 2).

The IRT will be responsible for the allocation of unique identification numbers to individual participants. Before the study is initiated, the log-in information and instructions for the IRT (including the process for emergency unblinding) will be provided to each site, plus a backup telephone number and call-in directions.

The blind will be maintained by the use of sonelokimab and matched placebo. The prefilled syringes used for all sonelokimab and placebo doses will be identical in appearance. The adalimumab injector will be different in appearance from syringes used for sonelokimab and placebo. However, the study will be blinded at the carton level. The outer packaging will be labeled in a blinded manner with no indication of the syringe contents (sonelokimab, placebo, or adalimumab). Participants will be asked to wear an eye mask for all injections.

10.7.2 Part B

At the beginning of Part B (Week 12) TJC68 and SJC66 response will be assessed in all participants. A responder is defined as a participant who achieves at least a 20% reduction in each of the TJC68 and SJC66 assessments at Week 12 compared with baseline. A non-responder is defined as a participant who does not achieve at least a 20% reduction in each of the TJC68 and SJC66 assessments at Week 12 compared with baseline.

In Part B participants will be allocated to treatment based upon response at Week 12 via the IRT. Treatment assignments in Part A and the allocation for Part B by responders and non-responders at Week 12 are described in Figure 1 and Table 3.

In Part B, the participants and investigators will remain double-blinded to participant treatment. Blinding of participants and investigators will be maintained in the same manner as in Part A.

10.7.3 Maintenance of Blinding

The primary analysis of 12-week data will occur at the completion of Part A, while the study is still ongoing. All sponsor personnel will remain blinded until Week 12. No unblinded participant-level data will be supplied to the study sites until the final database lock after the conclusion of the study.

Laboratory personnel performing the bioanalytical PK sample analysis may receive an open randomization list to enable analysis of relevant samples. In addition, a PK analyst and modeling/simulation scientist may receive an open randomization list to enable preparation of modeling/simulation activities. These individuals will not interact with site or contract research organization personnel.

10.8 Procedure for Breaking the Randomization Code

Emergency treatment code breaks are to be undertaken only when it is essential to treat the participant safely and efficaciously. Most often, study treatment discontinuation and knowledge of the possible treatment assignments are sufficient to treat a study participant who presents with an emergency condition. The investigator is encouraged to contact the medical monitor prior to code break. Emergency code breaks are performed using the IRT. When the investigator contacts the system to break a treatment code for a participant, they must provide the requested participant identifying information and confirm the necessity to break the treatment code for the participant. The investigator will then receive details of the study treatment for the specified participant and a fax or email confirming this information. [redacted]

It is the investigator's responsibility to ensure that there is a procedure in place to allow access to the IRT in case of emergency. The investigator will inform the participant how to contact their backup in cases of emergency when they are unavailable.

11 STUDY ASSESSMENTS AND PROCEDURES

11.1 Screening Assessments

11.1.1 Psoriatic Arthritis Medical History and Prior Medications

Relevant PsA medical history is to be collected at the Screening Visit, including time since PsA diagnosis, [redacted], and other relevant medical history. [redacted]

11.1.2 Medical History and Prior Medications

Relevant medical history is to be collected at Screening Visit, including history of SARS-CoV-2 vaccinations, drug and alcohol history, smoking history, and other relevant medical history.

11.1.3 Demographics

Demographic and disease characteristics will be collected including, but not limited to:

- Sex;
- Race;
- Ethnicity;
- Year of birth.

11.1.4 QuantiFERON TB Gold Testing

QuantiFERON TB Gold testing (or IGRA equivalent) will be conducted at screening to evaluate the participants' eligibility for the study. This blood-based assay is specific for *Mycobacterium tuberculosis* and is not influenced by previous BCG vaccination or by exposure to other mycobacterial species. The assay measures the production of interferon-gamma and presents it relative to a negative and a positive control sample. Details on the collection, shipment of samples and reporting of results by the central laboratory are provided to investigators in the study Laboratory Manual.

Participants with evidence of TB infection as defined by a positive QuantiFERON TB-Gold test (or IGRA equivalent) at screening are excluded unless the following criteria apply:

- A full TB work-up (according to local practice/guidelines) completed within 12 weeks prior to randomization establishes conclusively that the participant has no evidence of active or latent TB. Participants with evidence for active TB are excluded;
- Participants positive for latent TB per work-up must have completed sufficient treatment according to local routine clinical practice at least 4 weeks before randomization.

If a QuantiFERON TB Gold test (or IGRA equivalent) at screening is indeterminate, it can be repeated once. The results of the second test are to be used. If the second QuantiFERON TB Gold test is also indeterminate, the participant should be managed as for participants with positive QuantiFERON TB Gold test (or IGRA equivalent).

If there is reasonable medical doubt about a potentially false-positive QuantiFERON TB-Gold test (or IGRA equivalent) result, the test can be repeated once after obtaining Medical Monitor approval, and the results of the second test will prevail.

11.1.5 Hepatitis B and Hepatitis C Testing

Participants with known hepatitis B virus (HBV) or hepatitis C virus (HCV) infections will be excluded.

At screening, all participants will be tested for hepatitis B and C antibodies. Participants who are HCV core antibody positive (Ab+) must undergo further testing for HCV RNA by the central laboratory. HCV RNA must be negative per the central laboratory to allow further participation into the study.

Hepatitis B screening will follow the testing strategy illustrated in Figure 2.

Figure 2 Interpretation and Management of HBV Serologic Test Results

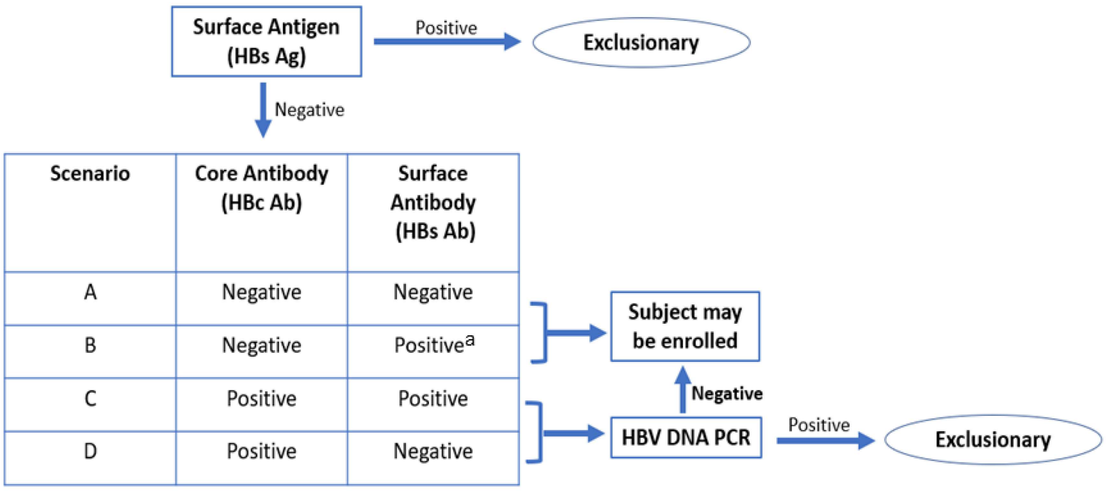

Abbreviations: HBc Ab = hepatitis B core antibody; HBs Ag = hepatitis B surface antigen; HBV = hepatitis B virus; PCR = polymerase chain reaction

a A positive test result for HBs Ab is expected for participants who have had a HBV vaccination. For participants without a history of HBV vaccination (and where mandated by local requirements), a positive result for HBs Ab requires HBV DNA PCR testing.

11.1.6 Human Immunodeficiency Virus Testing

Participants with known HIV infections at screening will be excluded. All participants will be tested at screening for HIV. Participants testing positive for HIV will be excluded from study participation. Samples for testing will be analyzed by the central laboratory. Details for the collection and handling of samples for HIV testing are in the Laboratory Manual.

11.1.7 SARS-CoV-2 Testing

At screening, all participants will be tested for SARS-CoV-2 (nasopharyngeal PCR test). The screening test will be performed by the central laboratory. If sample collection for the central laboratory is not possible at sites due to local procedures, a test may be obtained at a suitable laboratory as long as evidence is provided and retained as source data. The local test must be collected between 2 days prior and 3 days post-screening visit.

If a participant tests positive for SARS-CoV 2 infection at screening, another PCR or antigen test may be performed within the screening window. If the repeat test is negative and the participant is asymptomatic, they may enter the study.

11.1.8 Height and Weight

Height will be measured at screening. Weight will be measured at screening and at other study visits according to the SOA in Section 3, Table 1.

11.2 Efficacy Assessments

Efficacy assessments will be conducted according to the SOA in in Section 3, Table 1. For assessor-rated scales, the site will make every effort for each participant to be rated by the same site personnel at each time point.

11.2.1 Composite Disease Assessments

Composite disease assessments, such as ACR20/50/70, [redacted] minimal disease activity [redacted] used in this study are automatically calculated using results from assessment scales completed by participants or investigators (or qualified designees), and are not to be calculated manually by the investigator or study personnel.

11.2.1.1 American College of Rheumatology 20/50/70

The ACR response criteria [Felson D.T. et al., 1995] is a composite measure in which the improvement is observed in the number of swollen joints, number of tender joints, and the following 5 criteria: patient global assessment, physician's global assessment, functional ability measure (i.e., HAQ), visual analog pain scale, and hs-CRP.

ACR20 response is:

- At least a 20% improvement in TJC68;
- At least a 20% improvement in SJC66;

- At least a 20% improvement in at least 3 of the following 5 variables:
  - PtAAP;
  - PtGADA;
  - PhGADA;
  - HAQ-DI;
  - hs-CRP.

ACR50 response is:

- At least a 50% improvement in TJC68;
- At least a 50% improvement in SJC66;
- At least a 50% improvement in at least 3 of the following 5 variables:
  - PtAAP;
  - PtGADA;
  - PhGADA;
  - HAQ-DI;
  - hs-CRP.

ACR70 response is:

- At least a 70% improvement in TJC68;
- At least a 70% improvement in SJC66;
- At least a 70% improvement in at least 3 of the following 5 variables:
  - PtAAP;
  - PtGADA;
  - PhGADA;
  - HAQ-DI;
  - hs-CRP.

[redacted]

[redacted]

11.2.1.3 Minimal Disease Activity

Criteria for assessing minimal disease activity, listed below, covering all of the domains of the disease have been developed to determine whether the participant has reached minimal disease activity based on key outcome measures in PsA. Minimal disease activity is defined as meeting 5 of the 7 following criteria:

1. TJC68 ≤1;
2. SJC66 ≤1;
3. PASI ≤1, or PsO affecting ≤1% of BSA;
4. PtAAP ≤15 on a 0 to 100 VAS;
5. PtGADA ≤20 on a 0 to 100 VAS;
6. HAQ-DI ≤0.5;
7. LEI ≤1.

[redacted]

11.2.2 Patient Reported Outcomes

[redacted]

[redacted]

11.2.2.2 Patient's Global Assessment of Disease Activity

The PtGADA will be self-administered by the participant at visits indicated in the SOA in Section 3, Table 1.

The PtGADA will be performed using a horizontal 100 mm VAS, ranging from 0 (very well) to 100 (very poor) after the question: "Considering all the ways PsA affects you, please indicate with a vertical mark (|) through the horizontal line how well you are doing today."

11.2.2.3 Patient's Assessment of Arthritis Pain

The pain-VAS will be self-administered by the participant at visits as indicated in the SOA in Section 3, Table 1.

The PtAAP will be performed using a horizontal 100 mm VAS, ranging from 0 (no pain) to 100 (severe pain) after the question: "Please indicate with a vertical mark (|) through the horizontal line the most pain you had from your psoriatic arthritis today."

11.2.2.4 Health Assessment Questionnaire Disability Index

The HAQ-DI [Bruce B. et al., 2003] will be self-administered by the participant at visits as indicated in the SOA in Section 3, Table 1.

The HAQ-DI is a twenty-item PRO instrument that assesses current physical function/disability. The HAQ-DI covers 8 categories (dressing and grooming, hygiene, arising, reach, eating, grip, walking, and common daily activities). There are 4 response options, ranging from 0 (no difficulty) to 3 (unable to do). HAQ-DI score is reported as a mean score between 0 and 3 by dividing the total score by the number of items answered. The HAQ-DI has been the most-widely used instrument to assess physical function in clinical studies of treatments for rheumatoid and PsA and has extensive evidence of its validity and other psychometric properties in this context.

11.2.2.5 Short-Form-36 Health Survey-Version 2

The SF-36v2 [Ware J. et al., 1993] will be self-administered by the participant at visits as indicated in the SOA in Section 3, Table 1.

The SF-36v2 is a 36 item generic HRQoL instrument that uses a recall period of 4 weeks. Items are grouped into 8 domains as follows:

1. Limitations in physical activities because of health problems;
2. Limitations in social activities because of physical or emotional problems;
3. Limitations in usual role activities because of physical health problems;
4. Bodily pain;
5. General mental health (psychological distress and well-being);
6. Limitations in usual role activities because of emotional problems;
7. Vitality (energy and fatigue);
8. General health perceptions.

Each question has 5 answers scored 1 (unable to) to 5 (no difficulty). The scale score of each domain is calculated based on the summed score across items included in the domain and is rescaled to 0 to 100. Version 2 of the SF-36 questionnaire will be used in the study.

In addition to domain scores, the PCS and MCS scores are calculated from the 8 domains. Component scores appreciate the impact of each domain on physical and mental health status [Maruish M.E., 2011]. Each of the 8 domain scores and the component summary scores range from 0 to 100, with a higher score indicating a better health status. The 2 component summary scores are standardized with a mean of 50 and standard deviation (SD) of 10 in the general US population. Component summary scores for a study timepoint are to be calculated from the domains completed at that same timepoint.

The SF-36v2 PCS combines 4 scales: limitations on physical activities because of health problems, limitations in usual role activities because of physical health problems, bodily pain, and general health perceptions.

The SF-36v2 MCS combines the remaining 4 scales: limitations in social activities because of physical or emotional problems, general mental health (psychological distress and well-being), limitations in usual role activities because of emotional problems, and vitality (energy and fatigue).

11.2.2.6 Functional Assessment of Chronic Illness Therapy-Fatigue

The FACIT-Fatigue [Webster K. et al., 2003; Yellen S.B. et al., 1997] will be self-administered by the participant at visits as indicated in the SOA in Section 3, Table 1.

FACIT-Fatigue is a 13-item self-assessment of fatigue and the effect on daily activities and function over the previous 7 days. The 13 items use a 5-point Likert-type scale as follows:

- 0 = "Not at all;"
- 1 = "A little;"
- 2 = "Somewhat;"
- 3 = "Quite a bit;"
- 4 = "Very much."

The 13 scores are summed to create a single fatigue score.

11.2.2.7 Psoriatic Assessment Impact of Disease-12

The PsAID-12 [Gossec L. et al., 2014] will be self-administered by the participant at visits as indicated in the SOA in Section 3, Table 1.

PsAID-12 is a questionnaire including 12 health domains related to PsA. Each domain is evaluated on a scale of 0 (None) to 10 (Extreme). The 12 domains are: pain, fatigue, skin problems, work, and/or leisure activities, functional capacity, discomfort, sleep disturbance, coping, anxiety, embarrassment and/or shame, social participation, and depression.

11.2.2.8 Bath Ankylosing Spondylitis Disease Activity Index

The BASDAI [Garrett S. et al., 1994] will be self-administered by the participant at visits as indicated in the SOA in Section 3, Table 1.

The BASDAI is a validated composite index consisting of 6 participant numerical rating scales (NRS) to measure severity of the following:

- Fatigue;
- Axial involvement;
- Peripheral articular involvement;
- Localized tenderness/enthesopathy;
- Severity of morning stiffness;
- Time of morning stiffness.

Each NRS has a range of 0 to 10 where 0 represents "none" and 10 represents "very severe." For Question 6, 0 represents no stiffness and 10 represents 2 or more hours of stiffness.

The composite BASDAI score is the mean of the 6 individual NRS scores.

11.2.3 Physician Assessments

Investigators will collect clinical assessments and record data in an eCOA (Section 15.1). Calculations where required will be performed by the system.

11.2.3.1 Tender Joint Count and Swollen Joint Count

Joint counts [Duarte-García A. et al., 2019] will be performed by assessor(s) who must be well trained and part of the site personnel. Whenever possible, the same evaluator will perform these assessments at all visits.

Number of tender joints:

The 68 joints assessed for tenderness include the 2 temporomandibular, 2 sternoclavicular, 2 acromioclavicular joints, 2 shoulders, 2 elbows, 2 wrists, 10 metacarpophalangeal, 10 proximal interphalangeal, 8 distal interphalangeal joints of the hands, the 2 hips, 2 knees, 2 ankles, 2 mid-tarsal, 10 metatarsophalangeal, and 10 proximal interphalangeal joints of the feet.

Joints are to be scored as either tender (1) or not tender (0).

Number of swollen joints:

The 66 joints to be examined for swelling are the same as those examined for tenderness, however excluding both hip joints.

Joints are to be scored as either swollen (1) or not swollen (0).

Synovial fluid and/or soft tissue swelling but not bony overgrowth represent a positive result for SJC.

Data will be recorded for tender and swollen joints (right or left side), i.e., a box (no, yes, or not applicable) needs to be ticked for all joints. The total number of tender and swollen joints (right and left) will be automatically calculated.

11.2.3.2 Physician's Global Assessment of Disease Activity

PhGADA will be assessed by the investigator or a qualified designee at visits indicated in the SOA in Section 3, Table 1. PhGADA is assessed by rating the activity of PsA on the participant on a VAS of 0 (Very Well) to 100 (Very Poor).

11.2.3.3 Psoriasis Area and Severity Index

PASI [Carlin C.S. et al., 2004] will be assessed by an individual suitably qualified and experienced in the administration of this instrument, which may be the investigator or a qualified designee. This will occur at the visits indicated in the SOA in Section 3, Table 1. The PASI scores will be calculated automatically and investigators or study personnel are not to calculate it manually.

The PASI score is an established measure of clinical efficacy for PsO medications, which provides a numeric scoring for the participant's overall PsO disease state, ranging from 0 to 72. It is a linear

combination of percent of surface area of skin that is affected and the severity of erythema, infiltration, and desquamation over 4 body regions.

To calculate the PASI score, the 4 main body areas are assessed:

- Head (h);
- Trunk (t);
- Upper extremities (u);
- Lower extremities (l).

These correspond to 10%, 30%, 20%, and 40% of the total body area respectively.

The area of psoriatic involvement of these 4 areas (Ah, At, Au, and Al) is given a numerical value:

- 0 = No involvement;
- 1 = <10%;
- 2 = 10% to <30%;
- 3 = 30% to <50%;
- 4 = 50% to <70%;
- 5 = 70% to <90%;
- 6 = 90% to 100% involvement.

The signs of severity, erythema (E), infiltration (I), and desquamation (D) of lesions are assessed using a numeric scale 0 to 4 where 0 is a complete lack of cutaneous involvement and 4 is the severest possible involvement; scores are made independently for each of the areas, h, t, u, and l, and represent a composite score for each area.

An illustration of judging erythema follows:

- 0 = No erythema;
- 1 = Slight erythema;
- 2 = Moderate erythema;
- 3 = Striking erythema;
- 4 = Exceptionally striking erythema.

The PASI score is calculated according to the following formula:

$$PASI = 0.1(Eh+Ih+Dh)Ah + 0.3(Et+It+Dt)At + 0.2(Eu+Iu+Du)Au + 0.4(El+Il+Dl)Al$$

11.2.3.4 Body Surface Area Affected by Psoriasis

The BSA affected by PsO will be assessed by the investigator or a qualified designee at visits indicated in the SOA in Section 3, Table 1. The palm method will be used to estimate the amount of BSA as follows:

The participant's hand, including the palm, fingers, and thumb, is used as the reference point for measuring how much of their skin is affected by PsO, representing roughly 1% of the body's surface.

- Head and neck = 10% (10 palms);
- Upper extremities = 20% (20 palms);
- Trunk = 30% (30 palms);
- Lower extremities = 40% (40 palms).

Total BSA = 100%.

11.2.3.5 Leeds Dactylitis Index

LDI [Helliwell P.S. et al., 2005] will be assessed by an individual suitably qualified and experienced in the administration of this instrument, which may be the investigator or a qualified designee. This will occur at the visits indicated in the SOA in Section 3, Table 1.

The LDI basic measures the ratio of the circumference of the affected digit to the circumference of the digit on the opposite hand or foot, using a minimum difference of 10% to define a dactylitic digit. The ratio of circumference is multiplied by a tenderness score, using a modification of LDI which is a binary score (1 for tender, 0 for non-tender). If both sides are considered involved, or the circumference of the contralateral digit cannot be obtained, the number will be compared to data provided in the standard reference table for hands (Table 6) and feet (Table 7). This modification is referred to as LDI basic and will be applied in this study. The LDI requires a finger circumference gauge or a tape measure to measure digital circumference.

<u>Dactylitis count</u>

The dactylitis count is the number of fingers and toes with dactylitis, with a range of 0 to 20.

<u>Presence of dactylitis</u>

If dactylitis is present with any finger or toe, the participant is counted as a participant with dactylitis.

Table 6 Reference Circumference for Hands (measured in cm)

Digit	Men	Women
Thumb	7.0	5.8

Digit	Men	Women
Index	6.3	5.4
Middle	6.3	5.4
Ring	5.9	5.0
Little	5.2	4.4

Table 7 Reference Circumference for Feet (measured in cm)

Digit	Men	Women
Central toe	8.2	7.2
Second	5.2	4.6
Middle	5.0	4.4
Fourth	5.0	4.4
Little	5.2	4.5

11.2.3.6 Enthesitis Evaluation (Leeds Enthesitis Index and Spondyloarthritis Research Consortium of Canada Enthesitis Index)

Physical examinations to assess enthesial tenderness will be carried out by the investigator or a qualified designee at the visits indicated in the SOA in Section 3, Table 1.

Enthesitis count

The examiner will use a standardized assessment method for each entheseal site (use of the thumb or index finger, applying enough pressure to blanch the finger about a fifth of the way from the tip of the fingernail). Tenderness on examination will be recorded as either present (1) or absent (0) for each of the sites.

The sites to be assessed are as follows:

- Greater trochanter (right/left [R/L]);
- Quadriceps tendon insertion into the patella (R/L);
- Patellar ligament insertion into the patella or tibial tuberosity (counted as one site) (R/L);
- Achilles tendon insertion (R/L);
- Plantar fascia insertion (R/L);
- Medial epicondyle (R/L);
- Lateral epicondyle (R/L);
- Supraspinatus insertion into greater tuberosity of humerus (R/L);
- Medial condyle (R/L).

The enthesitis count will be used to derive the SPARCC and Leeds Enthesitis Indices.

Presence of enthesitis

If enthesitis is present with any of the 6 sites used for the LEI, the participant is counted as a participant with enthesitis for the purpose of calculating enthesitis responders per the LEI.

Indices

LEI [Healy P.J. et al., 2008] is a validated enthesitis index that uses 6 sites for evaluation of enthesitis (lateral epicondyle humerus [R/L], Achilles tendon insertion [R/L], and medial condyle femur [R/L]), giving an overall score range of 0 to 6. The LEI demonstrated substantial to excellent agreement with other scores in the indication of PsA.

The SPARCC Index [Maksymowych W.P. et al., 2009] uses 18 sites: greater trochanter (R/L), quadriceps tendon insertion into the patella (R/L), patellar ligament insertion into the patella or tibial tuberosity (R/L), achilles tendon insertion (R/L), plantar fascia insertion (R/L), medial epicondyle (R/L), lateral epicondyle (R/L) and supraspinatus insertion into greater tuberosity of humerus (R/L). The maximum score is 16 (distal patella and tibial tuberosity considered as one site). The SPARCC Index has demonstrated consistent ability to discriminate between treatment and placebo in clinical studies of PsA.

11.2.3.7 Modified Nail Psoriasis Severity Index

The mNAPSI [Cassell S.E. et al., 2007] is a tool to assess nail involvement in participants with PsA with an index score ranging from 0 to 130 for all fingernails. The mNAPSI will be assessed by an individual suitably qualified and experienced in the administration of the instrument, which may be the investigator or a qualified designee. This will occur at the visits indicated in the SOA in Section 3, Table 1. The mNAPSI will be calculated automatically and investigators or study personnel are not to calculate it manually.

The instructions for mNAPSI are:

If you question which grade to give, your answer should be the lower of the grades. Three features or groups of features (pitting, onycholysis and oil-drop dyschromia, and crumbling) of each fingernail will be graded on a scale from 0 to 3, according to the directions below. Four features (leukonychia, splinter hemorrhages, hyperkeratosis, and red spots in the lunula) will be graded as either present or absent for each fingernail.

1. **Onycholysis:** Separation of the nail plate from the nail bed. The separated part of the nail is opaque and can have white, yellow, or greenish tinge. If there is a piece of nail missing, estimate where the nail normally would have ended at the end of the nail bed, and count that missing part as involved in onycholysis.

   Oil-drop (salmon patch) dyschromia: Reddish-brown discoloration under the nail plate. Onycholysis and oil-drop dyschromia are considered together. When looking at the nail,

combine the total percentage area of the nail that is affected by either and use that combined total to score the nail (Table 8).

Table 8 mNAPSI Scores for Onycholysis

Score	Percent of nail with onycholysis or oil-drop dyschromia present
0	No onycholysis or oil-drop dyschromia present
1	1% to 10% of the nail has onycholysis or oil-drop dyschromia
2	11% to 30% of the nail has onycholysis or oil-drop dyschromia
3	>30% of the nail has onycholysis or oil-drop dyschromia

Abbreviations: mNAPSI = modified Nail Psoriasis Severity Index

2. **Pitting:** Small, sharply defined depressions in the nail surface. Pits are discrete abnormalities ("ice-pick-like"). If there is nail plate crumbling that is confluent with pits, do not score for pits. If the pits are separate from crumbling, they may be scored regardless of whether crumbling is present or not (Table 9).

Table 9 mNAPSI Scores for Number of Pits

Score	Number of Pits
0	0
1	1 to 10
2	11 to 49
3	>50

Abbreviations: mNAPSI = modified Nail Psoriasis Severity Index

3. **Nail plate crumbling:** Crumbling or fragmentation of friable nail plate which may be associated with confluent pitting. Crumbling involves alteration of the nail plate surface. Horizontal ridging of the nail, "wave-like" appearance, and horizontal lines are all features of crumbling (Table 10).

Table 10 mNAPSI Scores for Percent of Nail With Crumbling Present

Score	Number of Pits
0	No Crumbling
1	1 to 25% of the nail is crumbling
2	26 to 50% of the nail is crumbling
3	>50% of the nail is crumbling

Abbreviations: mNAPSI = Modified Nail Psoriasis Severity Index

The next 4 abnormalities are scored only by their presence or absence. A score of 1 indicates present and a score of zero indicates not present.

1. **Leukonychia:** White spots in the nail plate due to PsO in the mid-matrix. Leukonychia are just color changes. If it appears that there is depression or irregularity to the nail surface, this may be pitting or crumbling, not leukonychia. If the leukonychia is adjacent to, or confluent with crumbling or pits, it is counted as part of the crumbling or pitting and not as a separate abnormality.
2. **Splinter hemorrhages:** Small, longitudinal, linear, dark brown hemorrhage under the fingernail.
3. **Nail bed hyperkeratosis:** Thickened keratin in the nail bed.
4. **Red spots in the lunula:** Small pink or red macules in the lunula.

11.3 Safety Assessments

The timing and frequency of safety assessments are described in Section 3, Table 1.

11.3.1 Definitions

The definition of AEs, TEAE, AESIs, and SAEs is given below. The investigator and any qualified designees are responsible for detecting, documenting, and recording events that meet the definition of an AE, AESI, or SAE and for following up AEs throughout the study and to resolution or stabilization.

11.3.1.1 Adverse Events

An AE is defined as any untoward medical occurrence in a participant, or clinical investigation participant administered a pharmaceutical product, and which does not necessarily have a causal relationship with this treatment. An AE can therefore be any unfavorable and unintended sign, symptom, or disease temporally associated with the use of a medicinal (investigational) product, whether or not related to the medicinal (investigational) product.

11.3.1.2 Events Meeting the Adverse Events Definition

- Any abnormal laboratory test results (hematology, clinical chemistry, or urinalysis) or other safety assessments (e.g., ECG, radiological scans, vital signs measurements), including those that worsen from baseline, considered clinically significant in the medical and scientific judgment of the investigator (i.e., not related to progression of underlying disease);
- Exacerbation of a chronic or intermittent pre-existing condition including either an increase in frequency and/or intensity of the condition;
- New conditions detected or diagnosed after the participant has signed the consent form, though it may have been present before the start of the study;
- Signs, symptoms, or the clinical sequelae of a suspected drug-drug interaction;

- Signs, symptoms, or the clinical sequelae of a suspected overdose of either study treatment or a concomitant medication. [redacted]

11.3.1.3 Events Not Meeting the Adverse Events Definition

- "Lack of efficacy" or "failure of expected pharmacological action" per se will not be reported as an AE or SAE. Such instances will be captured in the efficacy assessments. However, the signs, symptoms, and/or clinical sequelae resulting from lack of efficacy will be reported as an AE or SAE if they fulfill the definition of an AE or SAE;
- Any clinically significant abnormal laboratory findings or other abnormal safety assessments that are associated with the underlying disease, unless judged by the investigator to be more severe than expected for the participant's condition;
- The disease/disorder being studied or expected progression, signs, or symptoms of the disease/disorder being studied, unless more severe than expected for the participant's condition;
- Medical or surgical procedure (e.g., endoscopy, appendectomy): the condition that leads to the procedure is the AE;
- Situations in which an untoward medical occurrence did not occur (social and/or convenience admission to a hospital);
- Anticipated day-to-day fluctuations of pre-existing disease(s) or condition(s) present or detected at the start of the study that do not worsen.

11.3.1.4 Serious Adverse Event

An SAE is defined as any untoward medical occurrence that:

- Results in death. The cause of death is the AE; death is an outcome;
- Is life-threatening. The term "life-threatening" in the definition of "serious" refers to an event in which the participant was at risk of death at the time of the event. It does not refer to an event, which hypothetically might have caused death, if it were more severe;
- Requires in-patient hospitalization or prolongs existing hospitalization. In general, hospitalization signifies that the participant has been detained (usually involving at least an overnight stay) at the hospital or emergency ward for observation and/or treatment that would not have been appropriate in the physician's office or out-patient setting. Complications that occur during hospitalization are AEs. If a complication prolongs hospitalization or fulfills any other serious criteria, the event is serious. When in doubt as to whether "hospitalization"

occurred or was necessary, the AE will be considered serious. Hospitalization for elective treatment of a pre-existing condition that did not worsen from baseline is not considered an SAE;

- Results in persistent or significant disability/incapacity. The term disability means a substantial disruption of a person's ability to conduct normal life functions. This definition is not intended to include experiences of relatively minor medical significance such as uncomplicated headache, nausea, vomiting, diarrhea, influenza, and accidental trauma (e.g., sprained ankle) which may interfere with or prevent everyday life functions but do not constitute a substantial disruption;
- Is a congenital anomaly/birth defect;
- Is an important medical event. Important medical events that may not result in death, be life-threatening, or require hospitalization may be considered an SAE, when based on appropriate medical judgment, they may jeopardize the participant or the participant may require medical or surgical intervention to prevent one of the outcomes listed in this definition. Examples of such events include invasive or malignant cancers, intensive treatment in an emergency room or at home for allergic bronchospasm, blood dyscrasias, or convulsions that do not result in hospitalization, or development of drug dependency or drug abuse;
- All malignant neoplasms will be assessed as serious under "medically significant" if other seriousness criteria are not met.

On an ongoing basis, safety data (including SAEs) will be reviewed by an independent DSMB (Section 11.3.12). Details on the DSMB's members, roles, and procedures to ensure trial integrity will be available in the DSMB charter.

11.3.1.5 Treatment-Emergent Adverse Event

TEAEs are defined as any AE occurring or worsening on or after the first dose of study treatment.

[redacted]

11.3.1.7 Extra-musculoskeletal Manifestations for Additional Monitoring

During the monitoring of AEs and SAEs at the Week 12 Visit, EOT Visit, and Safety Follow-up Visit, the investigator is to inquire whether the participant has experienced new onset or worsening of AEs during the study that might be related to extra-musculoskeletal manifestations of PsA, [redacted]

11.3.1.8 Pregnancy

Women who become pregnant during the study must be discontinued from study treatment immediately.

Pregnancy itself is not regarded as an AE unless there is a suspicion that the study treatment may have interfered with the effectiveness of a contraceptive medication. However, the outcome of all pregnancies (spontaneous miscarriage, elective termination, normal birth, or congenital abnormality) must be followed up and documented even after the participant has been withdrawn from the study.

All reports of congenital abnormalities/birth defects are SAEs. Spontaneous miscarriages, ectopic pregnancy, threatened abortion, intrauterine death, and still birth should also be reported and handled as SAEs. Elective abortions without complications should not be handled as SAEs, but should be reported as a follow-up report for the pregnancy. All outcomes of pregnancy must be reported to ICON on a Pregnancy Outcomes Report Form.

Pregnancy outcomes must be collected for the female partners of any male study participants who become pregnant within 12 weeks of their partner’s last dose of study treatment. Consent to report information regarding these pregnancy outcomes should be obtained from the female partner.

Pregnancies must be reported to ICON Pharmacovigilance and Safety Services (PVSS) within 24 hours of awareness, using the reporting details provided in Section 11.3.6.

11.3.1.9 Deaths

Any participant deaths that occur during the study will be considered SAEs and reported as per Section 11.3.6. If a death occurs, the investigator may be asked to provide a copy of any post-mortem findings, including histopathology.

11.3.2 Time Period and Frequency for Collecting Adverse Events and Serious Adverse Events Information

For the purposes of this study, any detrimental change in the participant's condition after signing the ICF and up to completion of the Safety Follow-up Visit (or until withdrawal from the study, whichever is later) will be considered an AE. AEs will be collected at every study visit (Section 3, Table 1). Any relevant observations made prior to the first dose of study treatment are to be

recorded on the AE eCRF but will not be considered TEAEs and will be reported separately from TEAEs.

At any time after completion of the Safety Follow-up, if an investigator learns of an SAE that can be reasonably related to study treatment, they will promptly notify the sponsor.

11.3.3 Method of Detecting Adverse Events and Serious Adverse Events

Care will be taken not to introduce bias when detecting AEs and SAEs. Open-ended and non-leading verbal questioning of the participant is the preferred method to enquire about AE occurrences.

See Section 11.3.1.7 for a discussion of extra-musculoskeletal manifestations of PsA for additional monitoring at the Week 12 Visit, EOT Visit, and Safety Follow-up Visit.

11.3.4 Recording of Adverse Events and Serious Adverse Events

Any AE is to be recorded starting immediately after the participant provides informed consent through the end of the safety follow-up period (or until withdrawal from the study, whichever is later).

When an AE/SAE occurs, it is the responsibility of the investigator to review all documentation (e.g., hospital progress notes, laboratory reports, and diagnostics reports) related to the event. The investigator will then record all relevant AE/SAE information in the eCRF. It is not acceptable for the investigator to send photocopies of the participant's medical records in lieu of completion of the AE/SAE eCRF page. There may be instances when copies of medical records for certain cases are requested. In this case, all participant identifiers, with the exception of the study participant number, will be redacted on the copies of the medical records before submission. The investigator will attempt to establish a diagnosis of the event based on signs, symptoms, and/or other clinical information. Whenever possible, the diagnosis (not the individual signs/symptoms) will be documented as the AE/SAE.

The following variables will be recorded for each AE: verbatim/AE description and date for AE start and stop, intensity (Section 11.3.4.1), seriousness (Section 11.3.1.4), causality rating (Section 11.3.4.2), whether or not the AE caused the participant to discontinue (Section 9.4), any other action (Section 11.3.4.3). Continuous events should be recorded at the maximum severity observed.

Expedited reporting, as described in Section 11.3.6, is required for SAEs, but not required for nonserious AEs or laboratory abnormalities.

11.3.4.1 Assessment of Intensity

The investigator will make an assessment of intensity for each AE and assign it to one of the following categories, and where appropriate, in accordance with the CTCAE Version 5.0:

- Grade 1 (Mild): An event that is easily tolerated by the participant, causing minimal discomfort, and not interfering with everyday activities;
- Grade 2 (Moderate): An event that causes sufficient discomfort and interferes with normal everyday activities;
- Grade 3 (Severe): An event that prevents normal everyday activities. An AE that is assessed as severe should not be confused with a SAE. Severe is a category utilized for rating the intensity of an event; and both AEs and SAEs can be assessed as severe;
- Grade 4 (Life-threatening): A life-threatening event is to be reported as an SAE;
- Grade 5 (Death): Death is to be reported as an SAE.

An event is defined as "serious" when it meets at least 1 of the predefined outcomes as described in the definition of an SAE, NOT when it is rated as severe.

11.3.4.2 Assessment of Causality

The investigator is obligated to assess the relationship between study treatment and each occurrence of each AE. The assessment will be "related," or "not related" as per Table 11 and captured in the eCRF.

A "reasonable possibility" of a relationship conveys that there are facts, evidence, and/or arguments to suggest a causal relationship, rather than a relationship cannot be ruled out. The investigator will use clinical judgment to determine the relationship.

Alternative causes, such as underlying disease(s), concomitant therapy, and other risk factors, as well as the temporal relationship of the event to study treatment administration will be considered and investigated.

The investigator will also consult the IB and/or Product Information, for marketed products, in his/her assessment. For each AE, the investigator must document in the medical notes that he/she has reviewed the AE and has provided an assessment of causality.

Table 11 Definition of Causality Terms for Adverse Events

Term	Definition
not related	Not suspected to be related to the study treatment. AE could not medically (pharmacologically/clinically) be attributed to the study treatment under study in this clinical trial protocol. A reasonable alternative explanation must be available.
related	Suspected to be related to the study treatment. AE could medically (pharmacologically/clinically) be attributed to the study treatment under study in this clinical trial protocol. In the absence of a reasonable alternative explanation, a relationship to the study treatment cannot be excluded.

Abbreviations: AE = adverse event

11.3.4.3 Other Data to Be Recorded for Adverse Events

In additions to intensity and causality the following should be recorded in the eCRF for each AE:

- Action taken with study treatment due to AE;
- Other action taken;
- AE outcome.

11.3.5 Follow-up of Adverse Events or Serious Adverse Events

All ongoing AEs should be followed up for 8 weeks after the last administration of study treatment. Any ongoing study treatment related AEs, which should be followed until resolution, or stabilization. Any new SAEs occurring up to 8 weeks after the last administration of study treatment should be reported to the sponsor or designee according to Section 11.3.6.

11.3.6 Reporting of Serious Adverse Events

All SAEs must be reported according to International Council for Harmonisation of Technical Requirements for Pharmaceuticals for Human Use (ICH) GCP or local regulations, applying the regulation with the stricter requirements.

- **Within 24 hours of becoming aware of a SAE:**

[redacted]

Initial notification via any other means than a completed SAE form does not replace the need for the investigator to provide a completed SAE report form within the designated reporting time frames.

There may be instances when copies of medical records for certain cases are requested by the sponsor. In this case, all participant identifiers, with the exception of the participant number, will be redacted on the copies of the medical records before submission to the sponsor or designee.

There may be situations in which an SAE has occurred and the investigator has minimal information to include in the initial report to the sponsor or designee. However, it is very important that the investigator always assess causality for every event before the initial transmission of the SAE data to the sponsor or designee.

11.3.6.1 Safety Reporting to Health Authorities, Independent Ethics Committees/Institutional Review Boards, and Investigators

[redacted]

A SUSAR is a subset of SAE. The sponsor or designee will report SUSARs to appropriate regulatory authorities, investigators, and IEC/IRBs according to local applicable laws and regulations, including European Directive 2001/20/EC, Regulation (EU) No 536/2014, and FDA Code of Federal Regulations 21 CFR Parts 312 and 320.

11.3.6.2 24/7 Medical Emergency Coverage for Urgent Protocol-related Medical Questions

[redacted]

On this internet page, a list of country-specific toll-free telephone numbers are provided. Note that not all countries globally have access to toll-free numbers as indicated on the "24/7 Medical Help-desk" index. Countries without toll-free numbers need to dial the chargeable number as indicated above. Furthermore, there may be restrictions when calling toll-free numbers from a mobile phone.

11.3.7 Laboratory Assessments

All chemistry and hematology parameters will be analyzed using a central laboratory. Urinalysis will be performed using the local laboratory. The parameters listed in Table 12 will be collected.

Table 12 Clinical Laboratory Tests

Laboratory Testing Profile	Tests Included
Laboratory tests required prior to first study treatment dose only	QuantiFERON TB-gold test (or equivalent IGRA), HIV testing, hepatitis B serology (HBV PCR if required) including hepatitis B surface antigen and antibody and hepatitis B core antibody, hepatitis C serology, HCV antibodies, confirmatory HCV PCR, SARS-CoV-2 PCR, RF, CCP antibodies, FSH, serum pregnancy
Blood chemistry	Sodium, potassium, chloride, bicarbonate, total protein, blood urea nitrogen, serum creatinine, albumin, alkaline phosphatase, AST, ALT, total bilirubin, direct bilirubin, indirect bilirubin, calcium, GGT, creatine kinase, hs-CRP
Hematology	Hemoglobin, hematocrit, WBC with differentials (monocytes, eosinophils, basophils, neutrophils, lymphocytes) as an absolute value, RBC count, platelet count, ESR
Urinalysis	Local urine testing<br>Routine urinalysis will be performed locally with urine dipstick test (semi-quantitative "dipstick" evaluation of specific gravity, pH, glucose, protein, bilirubin, ketones, leukocytes, blood).<br>If the dipstick urine test indicates any abnormal findings, the urine sample will be sent to the central laboratory for further assessments, as appropriate (reflex testing).<br>Central lab urine testing (reflex testing)<br>Urine chemistry (albumin:creatinine and protein:creatinine ratios) to be performed if dipstick indicates protein;<br>Microscopy and/or culture to be performed if clinically indicated or if urinalysis results positive (blood, protein or leukocyte esterase/WBC)<br>*Note*: Urine hCG pregnancy testing for WOCBP; may be repeated more frequently than indicated if required by local practice, if a menstrual cycle is missed, or if potential pregnancy is suspected
Fasting lipid panel and glucose to be performed at Day 1/Week 0 Visit and EOT Visit	Fasting: glucose, total cholesterol, low density lipoprotein, high density lipoprotein, triglycerides

Abbreviations: ALT = alanine aminotransferase; AST = aspartate aminotransferase; CCP = cyclic citrullinated peptide; EOT = End-of-Treatment; ESR = erythrocyte sedimentation rate; FSH = follicle stimulating hormone; GGT = gamma-glutamyl transferase; HBV = hepatitis B virus; hCG = human chorionic gonadotropin; HCV = hepatitis C virus; HIV = human immunodeficiency virus; hs-CRP = high sensitivity C-reactive protein; IGRA = interferon-gamma release assay; PCR = polymerase chain reaction; RBC = red blood cell; RF = rheumatoid factor; TB = tuberculosis; WBC = white blood cell; WOCBP = women of childbearing potential

11.3.8 Electrocardiogram Assessments

Computerized 12-lead ECG recordings will be obtained at scheduled study visits after the participant has rested for at least 5 minutes in the supine position. ECG data will be submitted to a central laboratory for measurement.

11.3.9 Physical Examination

A standard complete physical examination will be performed at the weeks specified in the SOA (Section 3, Table 1). The following parameters and body systems will be examined and any abnormalities described: height, weight, general appearance, skin (presence of rash), HEENT (head, ears, eyes, nose, throat), lungs (auscultation), heart (auscultation for presence of murmurs, gallops, rubs), lower extremity exam, abdomen (palpation and auscultation), neurologic (mental status, station, gait, reflexes, motor and sensory function, coordination) and lymph nodes. Any clinically significant changes from the Screening Visit will be recorded as AEs.

11.3.10 Vital Signs

Body temperature, respiration rate, heart rate (measured after at least 5 minutes of rest in the supine position), and systolic and diastolic cuff BP (measured after at least 5 minutes of rest in the supine position) will be recorded according to the SOA (Section 3, Table 1). Automatic or manual devices may be used, but the same device will be used for any given participant throughout the study. The same arm will be used for all BP measurements. All devices must hold valid calibration documentation at the time of use.

[redacted]

[redacted]

11.3.12 Data and Safety Monitoring Board

An independent DSMB will be formed to review the benefit-risk profile of sonelokimab during the study and to independently safeguard the interests of study participants.

The DSMB will review selected safety and efficacy data across the study at predefined intervals. The DSMB may also be called upon ad hoc, for example for the review of potential study stopping events (see Section 9.4.2).

The DSMB will consist of at least 3 voting members and include expertise in rheumatology, dermatology, and infectious diseases. DSMB members may not participate in this trial as investigators or in any other capacity.

An experienced unblinded biostatistician will be appointed to support the DSMB process. The DSMB unblinded biostatistician will be independent from all staff involved in the evaluation of the study data reviewed by the DSMB. Unblinded data will be reviewed only in closed sessions of the DSMB that are not attended by any personnel involved in the conduct of the study or evaluation of the data.

The DSMB may make recommendations to the sponsor regarding continuation, modification, or termination of the study. All recommendations of the DSMB will be made in writing, and minutes of all DSMB discussions will be kept. Minutes of closed sessions will be kept confidential until the study end.

Further details on the DSMB's members, roles, and procedures to ensure trial integrity will be described in the DSMB charter.

11.4 Overdose

A drug overdose is defined as the accidental or intentional use of a drug or medicine in an amount that is higher than is normally used. Every overdose must be reported to ICON PVSS within 24 hours of awareness, using the reporting method similar to reporting SAEs as detailed in Section 11.3.6, irrespective of whether the overdose was associated with an AE/SAE.

Overdose in this study is specifically defined as any dose greater than the intended protocol dose (Section 10.1). In case of overdose, it is recommended that the participant be monitored for any signs or symptoms of adverse reactions or effects and appropriate symptomatic treatment be administered immediately.

11.5 Hypersensitivity Reactions and Anaphylaxis

All protein therapeutics have the potential to be immunogenic. No anaphylactic reactions have been reported to date in patients exposed to sonelokimab.

As part of the safety monitoring strategy in this study, all sites will have immediate access to appropriate drugs and medical equipment to treat acute hypersensitivity and study personnel will be trained to recognize and treat hypersensitivity and anaphylaxis. Any potential cases of anaphylaxis will be defined and assessed using the criteria defined in the Second Symposium on the Definition and Management of Anaphylaxis in Appendix 4 in Section 21.4 [Sampson H.A. et al., 2006].

11.6 Immunogenicity Assessments

Antibodies to sonelokimab will be evaluated in serum samples collected from all participants according to the SOA (Section 3, Table 1). At dosing visits, the samples will be collected prior to dose administration. Details on the collection and shipping of immunogenicity samples are in the Laboratory Manual. These samples will be analyzed by the sponsor's designated laboratory.

The detection and characterization of antibodies to sonelokimab will be performed using a validated assay method by or under the supervision of the sponsor.

11.7 Pharmacokinetics

Blood samples will be collected for the measurement of trough (pre-dose) serum sonelokimab levels at the visits specified in the SOA (Section 3, Table 1). At dosing visits, the samples will be collected prior to dose administration. Details on the collection and shipping of PK samples are in the Laboratory Manual. Samples for PK will be analyzed by the sponsor's designated laboratory.

[redacted]

12 STATISTICAL CONSIDERATIONS

12.1 Sample Size and Power

The sample size calculation is based on the primary efficacy endpoint (i.e., ACR50 response rate at Week 12 compared with baseline) with the primary comparisons of interest being: sonelokimab 120 mg Q2W (Arm 1) vs placebo (Arm 4), sonelokimab 60 mg Q2W (Arm 2) vs placebo (Arm 4), and sonelokimab 60 mg Q4W (Arm 3) vs placebo (Arm 4), and determined based on assumed ACR50 response rates as follows:

- 40% with each sonelokimab dose regimen;
- 10% with placebo.

The estimated response rates for sample size were based on past IL-17 inhibitors for the treatment of patients with PsA. The Cosentyx FUTURE 2 study showed that ACR50 was achieved in 35% of secukinumab 300 mg and 37% of secukinumab 150 mg, vs 6% in the placebo arm [McInnes I.B. et al., 2015].

A sample size of n = 40 participants in each of the sonelokimab 120 mg Q2W, sonelokimab 60 mg Q2W, sonelokimab 60 mg Q4W, and placebo arms (therefore, 160 participants in total) results in a power of more than 80%, while assuming an overall 2-sided alpha of 0.025, a placebo ACR50 response rate of 10% and a difference in ACR50 response rate of 30% between placebo and each sonelokimab dose regimen. An additional 40 participants will be randomized in the adalimumab arm. The adalimumab treatment will be used as an active reference arm, however, no formal comparison of sonelokimab or placebo vs adalimumab is planned.

Eligible participants will be randomized in a 1:1:1:1:1 ratio to sonelokimab 120 mg Q2W (Arm 1), sonelokimab 60 mg Q2W (Arm 2), sonelokimab 60 mg Q4W (Arm 3), placebo (Arm 4), or adalimumab 40 mg Q2W (Arm 5). The total proportion of participants with previous use of biologic agents will be capped at 30%.

Due to the use of the full analysis set (FAS) with non-responder imputation, no accounting for dropout is required, therefore a total of approximately 200 participants will be randomized.

All calculations were performed in nQuery® Software version 8.5.1.0.

12.2 Populations for Analysis

12.2.1 Analysis Sets

The following populations for analysis are defined in Table 13.

Table 13 Populations for Analysis

Population	Description
Safety Analysis Set (SAF)	All randomized participants who received at least 1 dose of study treatment. Participants will be analyzed based on the treatment that they actually received.<br>The SAF will be used for all safety analyses.
Full Analysis Set (FAS)	All randomized participants. Participants will be analyzed in accordance to the ITT principle, i.e., based on randomized treatment, irrespective of the treatment they actually received.<br>The FAS will be used for all efficacy analyses on efficacy endpoints and will serve as the primary analysis set.

Abbreviations: FAS = full analysis set; ITT = intention-to-treat; SAF = safety analysis set

For the Part B exploratory analysis further analysis sets may be defined in the SAP.

12.2.2 Participant Disposition

The number of participants screened, randomized, randomized and treated, randomized and not treated, and the number of participants in the analysis sets will be summarized for all screened participants.

The number and percentage of participants will also be summarized for the following disposition categories:

1. Completed study treatment per-protocol;
2. Discontinued study treatment early and the reason for discontinuation;
3. Completed the study including post-treatment follow-up;
4. Discontinued from the study early and the reason for discontinuation.

The table will be presented by treatment arm using the FAS.

All participants' disposition data will be listed.

12.3 Statistical Analyses

The Statistical Analysis Plan (SAP) will be finalized prior to database lock for the primary analysis and will include a more technical and detailed description of the statistical analyses. This section

is a summary of the planned statistical analyses of the most important endpoints including primary and secondary endpoints.

12.3.1 General Considerations

For continuous variables, descriptive statistics (number of observations, mean, SD, minimum, first quartile, median, third quartile, maximum, and 95% CI, as appropriate) will be given. Categorical variables will be presented as counts and percentages with non-missing values in each category. The count of missing observations will be provided in all descriptive tables. For all statistical analyses, p-values and 95% CIs will be given.

For Part A, descriptive statistics will be presented by 5 treatment arms and by visits from Week 0 to Week 12:

- Arm 1: sonelokimab 120 mg Q2W;
- Arm 2: sonelokimab 60 mg Q2W;
- Arm 3: sonelokimab 60 mg Q4W;
- Arm 4: placebo;
- Arm 5: adalimumab 40 mg Q2W.

For Part B, descriptive statistics will be presented by 9 treatment arms and by visits from Week 0 to Week 24 and repeated by visits from Week 12 to Week 24:

- Sonelokimab 120 mg Q2W→sonelokimab 120 mg Q4W;
- Sonelokimab 120 mg Q2W→adalimumab 40 mg Q2W;
- Sonelokimab 60 mg Q2W→sonelokimab 60 mg Q4W;
- Sonelokimab 60 mg Q2W→sonelokimab 120 mg Q4W;
- Sonelokimab 60 mg Q4W→sonelokimab 60 mg Q4W;
- Sonelokimab 60 mg Q4W→sonelokimab 120 mg Q4W;
- Placebo→sonelokimab 120 mg Q4W;
- Adalimumab 40 mg Q2W→adalimumab 40 mg Q2W;
- Adalimumab 40 mg Q2W→sonelokimab 120 mg Q2W.

Baseline values will be defined as the last non-missing measurement prior to first dose of study treatment.

Change from baseline will be defined as the post-baseline visit value minus the baseline value.

All sensitivity analyses will be regarded as exploratory and no formal adjustment for multiplicity will be made. Details on sensitivity and planned subgroup analyses will be provided in the SAP.

All analyses will be performed using SAS® Software version 9.3 or later.

12.3.2 Primary Endpoints

The primary efficacy endpoint is described in Section 7.2.1.

12.3.2.1 Primary Analysis

For the primary analysis, the hypotheses to be tested are that the proportion of participants who achieve ACR50 at Week 12 compared with baseline in the sonelokimab 120 mg Q2W, sonelokimab 60 mg Q2W, or sonelokimab 60 mg Q4W arms are different from that of the placebo arm.

Only the sonelokimab 120 mg Q2W, sonelokimab 60 mg Q2W, and sonelokimab 60 mg Q4W arms will be compared pairwise with placebo. A comparison among dose arms (sonelokimab 120 mg Q2W, sonelokimab 60 mg Q2W, and sonelokimab 60 mg Q4W) is not planned. [redacted]

A logistic regression model will be used to test the pairwise comparisons (placebo and either sonelokimab 120 mg Q2W, sonelokimab 60 mg Q2W, or sonelokimab 60 mg Q4W) including fixed effects for treatment and the stratification factors (sex and exposure to biologic agents prior to the Screening Visit). Results will be presented in terms of odds ratios, as well as risk difference, 95% CI, and estimated p-value.

For the primary analysis, a non-responder imputation method will be used. Participants will be considered non-responders to treatment if they discontinue the study prior to the Week 12 Visit due to any reason, have a missing ACR50 score at baseline or at the Week 12 Visit due to any reason, or take prohibited medication prior to the Week 12 Visit.

The sensitivity estimand for the primary endpoint will be analysis using a Generalized Linear Mixed Model (GLMM) to account for any missing data due to withdrawals for reasons other than lack of efficacy or taking prohibited medications. The model will include fixed effects for treatment, sex, prior biologic use, visit, treatment by visit interaction. The correlations between repeated measures will be modeled using an unstructured covariance matrix.

The primary analysis will be performed using the FAS.

12.3.2.2 Type I Error Control for the Primary and Key Secondary Endpoints

To adjust for multiple testing and to control the family-wise error rate, the Bonferroni-Holm method will be applied first to sonelokimab 120 mg Q2W (Arm 1) and sonelokimab 60 mg Q2W (Arm 2), followed by a step-down procedure to test for sonelokimab 60 mg Q4W (Arm 3). The

smaller of the p-values $p_{SLK120mgQ2W}$ and $p_{SLK60mgQ2W}$ will be compared to alpha/2 = 0.025, and the greater p-value to alpha = 0.05, where $p_{SLK120mgQ2W}$ is the p-value resulting from the comparison between sonelokimab 120 mg Q2W (Arm 1) and placebo (Arm 4) and $p_{SLK60mgQ2W}$ is the p-value resulting from the comparison between sonelokimab 60 mg Q2W (Arm 2) and placebo (Arm 4). If both $p_{SLK120mgQ2W}$ and $p_{SLK60mgQ2W}$ are significant then the testing procedure will continue with the key secondary endpoints. The Bonferroni-Holm procedure used for the primary endpoint will be applied to each key secondary endpoint sequentially. If the procedure passes all the key secondary endpoints for both doses then sonelokimab 60 mg Q4W (Arm 3) will be compared to alpha = 0.05 firstly for the primary endpoint and then sequentially through the key secondary endpoints.

12.3.3 Secondary Endpoints

12.3.3.1 Key Secondary Endpoints

For the key secondary endpoints the hypotheses to be tested are that the proportion of participants who achieve the endpoints described in Section 7.2.2 at Week 12 in the sonelokimab 120 mg Q2W, sonelokimab 60 mg Q2W, or sonelokimab 60 mg Q4W arms are different from that of the placebo arm.

As per the primary analysis, only the sonelokimab 120 mg Q2W, sonelokimab 60 mg Q2W, and sonelokimab 60 mg Q4W arms will be compared pairwise with placebo. The adalimumab arm will serve as a reference arm only. A comparison among dose arms sonelokimab 120 mg Q2W, sonelokimab 60 mg Q2W, or sonelokimab 60 mg Q4W is not planned.

The statistical methods that will be used to test these hypotheses will be the same as described for the primary endpoint. The analysis of the key secondary endpoint PASI90 will only include the subgroup of participants with PsO involving at least 3% BSA at baseline. This is expected to be between 50% and 67% of the FAS.

A non-responder imputation method will be used as described for primary analysis of the primary endpoint.

The analysis will be performed using the FAS.

12.3.3.2 Other Secondary Endpoints

For the other secondary endpoints, listed in Section 7.2.3, no confirmatory statistical testing will be performed, but statistical tests may be used for exploratory purposes only. All endpoints will be assessed at time points as outlined in the SOA in Section 3, Table 1, if not indicated otherwise.

All continuous endpoints will be summarized descriptively and analyzed using a mixed model for repeated measures (MMRM) including treatment arm, visit, sex, prior exposure to biologic agents and treatment by visit interaction as fixed effects. An unstructured covariance matrix will be used for the MMRM analysis, unless the model does not converge, in which case the covariance matrix will be decided upon model convergence status and the Akaike information criterion. The

least-squares means in each arm, the least-squares mean difference between arms, and its 95% CIs and p-values will be estimated by the model. Treatment effect estimates will be also extracted by visit.

All dichotomous endpoints will be summarized descriptively and analyzed using logistic regression with odds ratios, as well as risk difference and associated 95% CIs and p-values.

For dichotomous endpoints, a non-responder imputation method will be done and details will be defined in the SAP. For continuous endpoints, no imputation is planned.

FAS will be used for all other secondary endpoints analysis.

12.3.3.3 Pharmacokinetic Analyses

Serum concentration data for sonelokimab will be listed and summarized using descriptive statistics. Serum sonelokimab concentration results may be used for other analyses (e.g., population PK modeling), which will be reported separately.

12.3.3.4 Immunogenicity Analyses

ADA data for sonelokimab will be listed and summarized using descriptive statistics. Results may be used to support other analyses (e.g., population PK modeling), which will be reported separately.

12.3.3.5 Part B Analysis

For the Part B analysis, no confirmatory statistical testing will be performed. All efficacy endpoints will be seen as secondary, and descriptive statistics will be presented by visit from Week 0 to Week 24 and repeated by visit from Week 12 to Week 24.

For other secondary dichotomous endpoints, a non-responder imputation method, accounting for visits missed due to COVID-19, will be used. For continuous endpoints, no imputation is planned.

[redacted]

12.3.4 Safety Analyses

For any of the safety endpoints, listed in Section 7.2.6, no formal statistical testing will be performed.

Safety analyses will be done separately for Part A and Part B. AEs will be assigned to either Part A or Part B using the event start date and time. AEs that start after first treatment of Part B will be used within the Part B analysis. For Part A, 5 arms will be considered:

- Arm 1: sonelokimab 120 mg Q2W;
- Arm 2: sonelokimab 60 mg Q2W;
- Arm 3: sonelokimab 60 mg Q4W;
- Arm 4: placebo;
- Arm 5: adalimumab 40 mg Q2W.

For Part B or for the whole treatment period, 9 arms will be considered:

- Sonelokimab 120 mg Q2W→sonelokimab 120 mg Q4W;
- Sonelokimab 120 mg Q2W→adalimumab 40 mg Q2W;
- Sonelokimab 60 mg Q2W→sonelokimab 60 mg Q4W;
- Sonelokimab 60 mg Q2W→sonelokimab 120 mg Q4W;
- Sonelokimab 60 mg Q4W→sonelokimab 60 mg Q4W;
- Sonelokimab 60 mg Q4W→sonelokimab 120 mg Q4W;
- Placebo→sonelokimab 120 mg Q4W;
- Adalimumab 40 mg Q2W→adalimumab 40 mg Q2W;
- Adalimumab 40 mg Q2W→sonelokimab 120 mg Q2W.

Participants will be followed for safety until the end of the Safety Follow-up Visit (or until withdrawal from the study, whichever is later).

The SAF will be used for all safety analyses.

12.3.4.1 Adverse Events

A summary table will be presented with total number and number of participants with the following:

- TEAEs;
- SAEs;
- Related TEAEs;
- Related SAEs;
- TEAEs leading to discontinuation;

- AESI;
- Fatal AEs.

TEAEs will be described using descriptive statistics and coded according to the Medical Dictionary for Regulatory Activities (MedDRA) system organ class (SOC) and MedDRA preferred term (PT), by treatment arms. Drug-related TEAEs and SAEs will also be summarized by treatment arms. Incidence rates of TEAEs during Part B and for the combined Part A + Part B analysis will be adjusted for exposure duration to study treatment (i.e., exposure adjusted incidence rates [EAIR]).

AEs will also be tabulated by intensity and relationship to treatment ("not related/related").

12.3.4.2 Other Safety Endpoints

Clinical safety laboratory (hematology, biochemistry, urinalysis); vital signs including body weight; ECGs; and physical examinations data will be presented by treatment arm and over time. For each visit, the actual result and the change from baseline will be presented. Shift tables for values outside the normal ranges will be presented, as appropriate.

Otherwise, safety data will be presented in tabular and/or graphical format and summarized descriptively, where appropriate. For continuous measurements (laboratory and vital signs data), change from baseline to each assessment timepoint will be additionally summarized by treatment arm. Participant listings will be produced for all safety parameters.

12.4 Subgroup Analyses

Subgroup analyses for the primary endpoint and key secondary endpoints may be considered for the following baseline variables:

- Age;
- Sex;
- Geographic region;
- Race;
- Body weight;
- Exposure to biologic agents prior to the Screening Visit;
- Concomitant methotrexate at baseline;
- Concomitant non-biologic DMARD at baseline;
- Concomitant corticosteroids at baseline;
- Baseline hs-CRP;
- Treatment-emergent ADA status.

All summaries will be based on imputed data as appropriate and will include descriptive statistics.

12.5 Handling of Missing Data

Missing data in this study may result from participants discontinuing from the study prematurely or missing intermediate visits or selected assessments while remaining on study. Every reasonable effort will be made to obtain the protocol-required data for all study assessments that are scheduled for all participants who have been enrolled.

For the dichotomous primary and key secondary endpoints, non-responder imputation method will be used for the primary estimand. For continuous endpoints, no imputation is planned.

For continuous efficacy endpoints, missing observations will not be formally imputed. MMRM analysis will use all available data, including participants with partial data (i.e., with missing data) in order to arrive at an estimate of the mean treatment effect. MMRM is based on the assumption of missing at random (MAR), i.e., dropout would behave similarly to other participants in the same treatment group, and possibly with similar covariate values, had they not dropped out.

Unless otherwise indicated, in other cases, the observed data will be used and no imputation will be performed.

12.6 Primary Analysis Timing

The primary analysis and unblinding of the data collected during Part A will be performed when all data up to and including Week 12 has been collected and the Part A database has been locked.

The study will be continued regardless of the results of the primary analysis.

13 ETHICS

13.1 Independent Ethics Committee/Institutional Review Board

Prior to the start of the study, the investigator is responsible for ensuring that the protocol and consent form have been reviewed and approved by a relevant IEC/IRB. The IEC/IRB shall be appropriately constituted and perform its functions in accordance with FDA, ICH GCP, and local requirements as applicable.

The IEC/IRB shall approve all protocol amendments (except for logistical or administrative changes), written informed consent documents and document updates, participant recruitment procedures (e.g., advertisements), written information to be provided to the participants, IB, available safety information, information about payment and compensation available to participants, the investigator's curriculum vitae and/or other evidence of qualifications, and any other documents requested by the IEC/IRB and Regulatory Authority (Competent Authority) as applicable.

13.2 Written Informed Consent

The nature and purpose of the study shall be fully explained to each participant. They must be informed that participation is voluntary.

Written informed consent must be obtained from each participant prior to any study procedures being performed. The process of obtaining informed consent must be documented in the participant’s source documents. The authorized person obtaining the informed consent must also sign the ICF, and a copy of the ICF must be provided to the participant.

The consent documents to be used for the study shall include all the elements of informed consent as outlined in accordance with FDA, ICH GCP, and local requirements as applicable and be reviewed and approved by the appropriate IEC/IRB prior to use.

14 QUALITY CONTROL AND QUALITY ASSURANCE

14.1 Conduct of the Study

The sponsor or designee shall implement and maintain quality control and quality assurance procedures with written standard operating procedure (SOP) to ensure that the study is conducted and data are generated, documented, and reported in compliance with the protocol, ICH GCP, and applicable regulatory requirements.

This study shall be conducted in accordance with the provisions of the Declaration of Helsinki (Oct 2013), FDA (CFR, Sections 312.50 and 312.56), EU (536/2014) and UK regulations (The Medicines for Human Use [Clinical Trials] Regulations 2004 [no.1031]), and with ICH GCP (CPMP 135/95).

The investigator will be responsible for the following:

1. Providing written summaries of the status of the study to the IEC/IRB annually or more frequently in accordance with the requirements, policies, and procedures established by the IEC/IRB;
2. Notifying the IEC/IRB of SAEs or other significant safety findings as required by IEC/IRB procedures.

The investigator may not deviate from the protocol without a formal protocol amendment having been established and approved by an appropriate IEC/IRB, except when necessary to eliminate immediate hazards to the participant or when the change(s) involve(s) only logistical or administrative aspects of the study. Any deviations may result in the participant having to be withdrawn from the study and render that participant non-evaluable.

The identification and reporting of serious breaches of ICH GCP or the protocol to the Regulatory Authorities and Ethics Committees will be conducted according to local SOPs and regulations.

14.2 Site Monitoring

The monitoring strategy for the study foresees a risk-based monitoring approach, in line with the relevant FDA and EMA recommendations, and will be described in detail by the study-specific risk-based Monitoring Plan.

Site monitoring includes both source data review and source data verification. Site monitors perform source data review of critical procedures to ensure that the safety and rights of participants are being protected and that the study is being conducted in accordance with the currently approved protocol, any other study agreements, ICH GCP, and all applicable regulatory requirements. Site monitors perform source data verification of critical data to confirm transcription of data entered into the eCRF by authorized site personnel are accurate, complete, and verifiable from source documents.

Monitoring details describing strategy (e.g., risk-based initiatives in operations/quality such as Risk Management, Mitigation Strategies, and Analytical Risk-Based Monitoring), methods, responsibilities and requirements, including handling of non-compliance issues and monitoring techniques (central, remote, or on-site monitoring) are provided in the Monitoring Plan.

The investigator, as part of his/her responsibilities, is expected to co-operate with ICON in ensuring that the study adheres to GCP requirements and local regulatory requirements. The investigator must maintain accurate documentation (source data) that supports the information entered in the eCRF. The investigator may not recruit participants into the study until such time that a visit, or with the agreement of the sponsor, attendance at the investigator meeting, has been made by a sponsor/ICON monitor to conduct a detailed review of the protocol, source documents, and eCRF.

The investigator shall grant direct access of original source documents and study records to the sponsor's or designee's Site Monitor in order to conduct source data review, to ensure that the participants' well-being is maintained, data are being recorded in an adequate manner according to ALCOAC principles (i.e., that they are attributable, legible, contemporaneous, original, accurate, and complete), that protocol and GCP adherence is satisfactory, and to verify accurate transcription of data to the eCRF.

15 DATA HANDLING AND RECORD KEEPING

15.1 Case Report Forms/Source Data Handling

Case report forms/external electronic data, such as the results from electronic PRO assessment tools, and central laboratory results will be entered/loaded into a validated electronic database.

All required study data must be entered in the eCRF created for the study. Some assessments will be captured by an eCOA/electronic patient reported outcome (ePRO) system which is synonymous with the eCRF. This data collection tool is a validated electronic data capture (EDC) system that contains a system generated audit trail. Data required according to this protocol are recorded by investigational site personnel via data entry into the internet based EDC software system. The investigator shall ensure that all data from participant visits are promptly entered into the eCRFs in accordance with the specific instructions given. The investigator must sign each eCRF to verify the integrity of the data recorded. All internal ICON and external investigational site personnel seeking access to the eCRF are supported by a Service Desk (if applicable). At the end of the study all data captured electronically will be provided to the investigator on CD ROM for archiving at the investigational site.

The investigator must maintain source documents, such as laboratory reports, X-rays, ECGs, consultation reports, and complete medical history and physical examination reports. All information in the eCRF must be traceable to the source documents in the participant’s file.

The investigator/institution shall provide direct access to source data/documents for study-related monitoring, audits, IEC/IRB review, and regulatory inspection.

The sponsor or designee is responsible for the data management of this study, including quality checking of the data.

15.2 Data Protection

Participants will be assigned a unique identifier by the IRT. Any participant records or datasets that are transferred to the sponsor or designee will contain the identifier only; participant names or any information which would make the participant identifiable will not be transferred. The participant must be informed that their personal study-related data will be used by the sponsor in accordance with local data protection law. The level of disclosure must also be explained to the participant. The participant must be informed that their medical records may be examined by Clinical Quality Assurance auditors or other authorized personnel appointed by the sponsor, by appropriate IEC/IRB members, and by inspectors from regulatory authorities.

15.3 Dissemination of Clinical Study Data

By signing this protocol, the investigator reaffirms to the sponsor or designee to maintain in confidence all information furnished or resulting from this study. The investigator will only divulge such information as may be necessary to the IRB, the members of the staff, and the participants who are involved in this study.

This study will be registered on ClinicalTrials.gov in accordance with applicable laws or publication policy and may also be registered on other publicly accessible websites as necessary.

The sponsor is responsible for preparing and providing the appropriate regulatory authorities with the clinical study report according to the applicable regulatory requirements. The sponsor will ensure that the clinical study report meets the standards of the ICH Guideline for Structure and Content of Clinical Study Reports (ICH E3).

15.4 Retention of Essential Documents

The investigator/institution will maintain the study documents as specified in the ICH guidelines on GCP and as required by the applicable regulatory requirements. The investigator/institution will take measures to prevent accidental or premature destruction of these documents.

Essential documents will be retained until at least 2 years after the last approval of a marketing application in an ICH region and until there are no pending or contemplated marketing applications in an ICH region or until at least 2 years have elapsed since the formal discontinuation of clinical development of the investigational product. These documents will be retained for a longer period, however, if required by the applicable regulatory requirements or by an agreement with the sponsor. It is the responsibility of the sponsor to inform the investigator/institution as to when these documents no longer need to be retained.

16 FINANCING AND INSURANCE

16.1 Contractual and Financial Details

The investigator (and/or, as appropriate, the hospital administrative representative) and the sponsor or designee will sign a clinical study agreement prior to the start of the study, outlining overall sponsor and investigator responsibilities in relation to the study. The contract will describe whether costs for pharmacy, laboratory, and other protocol-required services are being paid directly or indirectly.

16.2 Insurance, Indemnity, and Compensation

The sponsor will maintain an appropriate clinical study insurance policy.

16.3 Financial Disclosure

The investigator will provide the sponsor with sufficient, accurate financial information as requested to allow the sponsor to submit complete and accurate financial certification or disclosure statements to the appropriate regulatory authorities.

[redacted]

18 CONFLICT OF INTEREST POLICY

The independence of this study from any actual or perceived influence, such as by the pharmaceutical industry, is critical. Therefore any actual conflict of interest of persons who have a role in the design, conduct, analysis, publication, or any aspect of this study will be disclosed and managed. Furthermore, persons who have a perceived conflict of interest will be required to have such conflicts managed in a way that is appropriate to their participation in the study.

19 SIGNATURE OF INVESTIGATOR

I agree to conduct the study outlined above in accordance with the terms and conditions of the protocol, ICH guidelines on GCP, and with applicable regulatory requirements. All information pertaining to the study shall be treated in a confidential manner.

____ ________________________

Signature: Date (day/month/year)

____

Name:

____

Title:

20 REFERENCE LIST

Armstrong AW, Robertson AD, Wu J, et al. Undertreatment, treatment trends, and treatment dissatisfaction among patients with psoriasis and psoriatic arthritis in the United States: findings from the National Psoriasis Foundation surveys, 2003-2011. JAMA Dermatol 2013;149(10):1180-5.

Baraliakos X, Kiltz U, Peters S, et al. Efficiency of treatment with non-steroidal anti-inflammatory drugs according to current recommendations in patients with radiographic and non-radiographic axial spondyloarthritis. Rheumatology (Oxford) 2017;56(1):95-102.

Bruce B, Fries JF. The Stanford Health Assessment Questionnaire: a review of its history, issues, progress, and documentation. J Rheumatol 2003;30(1):167-78.

Carlin CS, Feldman SR, Krueger JG, et al. A 50% reduction in the Psoriasis Area and Severity Index (PASI 50) is a clinically significant endpoint in the assessment of psoriasis. J Am Acad Dermatol 2004;50(6):859-66.

Cassell SE, Bieber JD, Rich P, et al. The modified Nail Psoriasis Severity Index: validation of an instrument to assess psoriatic nail involvement in patients with psoriatic arthritis. J Rheumatol 2007;34(1):123-9.

Coates LC, Kavanaugh A, Mease PJ, et al. Group for Research and Assessment of Psoriasis and Psoriatic Arthritis 2015 Treatment Recommendations for Psoriatic Arthritis. Arthritis Rheumatol 2016;68(5):1060-71.

Conaghan PG, Alten R, Deodhar A, et al. Relationship of pain and fatigue with health-related quality of life and work in patients with psoriatic arthritis on TNFi: results of a multi-national real-world study. RMD Open 2020;6(2).

Coppieters K, Dreier T, Silence K, et al. Formatted anti-tumor necrosis factor alpha VHH proteins derived from camelids show superior potency and targeting to inflamed joints in a murine model of collagen-induced arthritis. Arthritis Rheum 2006;54(6):1856-66.

Duarte-García A, Leung YY, Coates LC, et al. Endorsement of the 66/68 joint count for the measurement of musculoskeletal disease activity: OMERACT 2018 psoriatic arthritis workshop report. J Rheumatol 2019;46(8):996-1005.

Duarte GV, Faillace C, Freire de Carvalho J. Psoriatic arthritis. Best Pract Res Clin Rheumatol 2012;26(1):147-56.

Eyerich K, Weisenseel P, Pinter A, et al. IL-23 blockade with guselkumab potentially modifies psoriasis pathogenesis: rationale and study protocol of a phase 3b, randomised, double-blind, multicentre study in participants with moderate-to-severe plaque-type psoriasis (GUIDE). BMJ Open 2021;11(9):e049822.

Felson DT, Anderson JJ, Boers M, et al. American College of Rheumatology. Preliminary definition of improvement in rheumatoid arthritis. Arthritis Rheum 1995;38(6):727-35.

FitzGerald O, Ogdie A, Chandran V, et al. Psoriatic arthritis. Nat Rev Dis Primers 2021;7(1):59.

Garrett S, Jenkinson T, Kennedy LG, et al. A new approach to defining disease status in ankylosing spondylitis: the Bath Ankylosing Spondylitis Disease Activity Index. J Rheumatol 1994;21(12):2286-91.

Gladman DD, Thavaneswaran A, Chandran V, et al. Do patients with psoriatic arthritis who present early fare better than those presenting later in the disease? Ann Rheum Dis 2011;70(12):2152-4.

Glatt S, Baeten D, Baker T, et al. Dual IL-17A and IL-17F neutralisation by bimekizumab in psoriatic arthritis: evidence from preclinical experiments and a randomised placebo-controlled clinical trial that IL-17F contributes to human chronic tissue inflammation. Ann Rheum Dis 2018;77(4):523-32.

Gossec L, Baraliakos X, Kerschbaumer A, et al. EULAR recommendations for the management of psoriatic arthritis with pharmacological therapies: 2019 update. Ann Rheum Dis 2020;79(6):700-12.

Gossec L, de Wit M, Kiltz U, et al. A patient-derived and patient-reported outcome measure for assessing psoriatic arthritis: elaboration and preliminary validation of the Psoriatic Arthritis Impact of Disease (PsAID) questionnaire, a 13-country EULAR initiative. Ann Rheum Dis 2014;73(6):1012-9.

Gottlieb A, Korman NJ, Gordon KB, et al. Guidelines of care for the management of psoriasis and psoriatic arthritis: Section 2. Psoriatic arthritis: overview and guidelines of care for treatment with an emphasis on the biologics. J Am Acad Dermatol 2008;58(5):851-64.

Gottlieb AB, Deodhar A, McInnes IB, et al. Long-term Safety of Secukinumab Over Five Years in Patients with Moderate-to-severe Plaque Psoriasis, Psoriatic Arthritis and Ankylosing Spondylitis: Update on Integrated Pooled Clinical Trial and Post-marketing Surveillance Data. Acta Dermato-Venereologica 2022;102:adv00698.

Haroon M, Gallagher P, FitzGerald O. Diagnostic delay of more than 6 months contributes to poor radiographic and functional outcome in psoriatic arthritis. Ann Rheum Dis 2015;74(6):1045-50.

Healy PJ, Helliwell PS. Measuring clinical enthesitis in psoriatic arthritis: assessment of existing measures and development of an instrument specific to psoriatic arthritis. Arthritis Rheum 2008;59(5):686-91.

Helliwell PS, Firth J, Ibrahim GH, et al. Development of an assessment tool for dactylitis in patients with psoriatic arthritis. J Rheumatol 2005;32(9):1745-50.

Houghton K, Patil D, Gomez B, et al. Correlation between change in psoriasis area and severity index and dermatology life quality index in patients with psoriasis: pooled analysis from four phase 3 clinical trials of secukinumab. Dermatol Ther (Heidelb) 2021;11(4):1373-84.

Hueber W, Patel DD, Dryja T, et al. Effects of AIN457, a fully human antibody to interleukin-17A, on psoriasis, rheumatoid arthritis, and uveitis. Sci Transl Med 2010;2(52):52ra72.

Husni ME, Merola JF, Davin S. The psychosocial burden of psoriatic arthritis. Semin Arthritis Rheum 2017;47(3):351-60.

Hymowitz SG, Filvaroff EH, Yin JP, et al. IL-17s adopt a cystine knot fold: structure and activity of a novel cytokine, IL-17F, and implications for receptor binding. EMBO J 2001;20(19):5332-41.

Jovčevska I, Muyldermans S. The Therapeutic Potential of Nanobodies. BioDrugs 2020;34(1):11-26.

Kavanaugh A, Helliwell P, Ritchlin CT. Psoriatic Arthritis and Burden of Disease: Patient Perspectives from the Population-Based Multinational Assessment of Psoriasis and Psoriatic Arthritis (MAPP) Survey. Rheumatol Ther 2016;3(1):91-102.

[redacted]

Li Z, Krippendorff BF, Sharma S, et al. Influence of molecular size on tissue distribution of antibody fragments. MAbs 2016;8(1):113-9.

Maksymowych WP, Mallon C, Morrow S, et al. Development and validation of the Spondyloarthritis Research Consortium of Canada (SPARCC) Enthesitis Index. Ann Rheum Dis 2009;68(6):948-53.

Maruish ME. User's Manual for the SF-36v2 Health Survey. Lincoln, RI: Quality Metric Incorporated; 2011. (Available upon request)

McGonagle DG, McInnes IB, Kirkham BW, et al. The role of IL-17A in axial spondyloarthritis and psoriatic arthritis: recent advances and controversies. Ann Rheum Dis 2019;78(9):1167-78.

McInnes IB, Behrens F, Mease PJ, et al. Secukinumab versus adalimumab for treatment of active psoriatic arthritis (EXCEED): a double-blind, parallel-group, randomised, active-controlled, phase 3b trial. Lancet 2020;395(10235):1496-505.

McInnes IB, Mease PJ, Kirkham B, et al. Secukinumab, a human anti-interleukin-17A monoclonal antibody, in patients with psoriatic arthritis (FUTURE 2): a randomised, double-blind, placebo-controlled, phase 3 trial. The Lancet 2015;386(9999):1137-46.

McInnes IB, Sieper J, Braun J, et al. Efficacy and safety of secukinumab, a fully human anti-interleukin-17A monoclonal antibody, in patients with moderate-to-severe psoriatic arthritis: a 24-

week, randomised, double-blind, placebo-controlled, phase II proof-of-concept trial. Ann Rheum Dis 2014;73(2):349-56.

Mease P, Goffe BS. Diagnosis and treatment of psoriatic arthritis. Journal of the American Academy of Dermatology 2005;52(1):1-19.

Mease PJ, McInnes IB, Kirkham B, et al. Secukinumab Inhibition of Interleukin-17A in Patients with Psoriatic Arthritis. N Engl J Med 2015;373(14):1329-39.

Mease PJ, Palmer JB, Liu M, et al. Influence of Axial Involvement on Clinical Characteristics of Psoriatic Arthritis: Analysis from the Corrona Psoriatic Arthritis/Spondyloarthritis Registry. J Rheumatol 2018;45(10):1389-96.

Mease PJ, Smolen JS, Behrens F, et al. A head-to-head comparison of the efficacy and safety of ixekizumab and adalimumab in biological-naïve patients with active psoriatic arthritis: 24-week results of a randomised, open-label, blinded-assessor trial. Ann Rheum Dis 2020;79(1):123-31.

Mease PJ, van der Heijde D, Ritchlin CT, et al. Ixekizumab, an interleukin-17A specific monoclonal antibody, for the treatment of biologic-naive patients with active psoriatic arthritis: results from the 24-week randomised, double-blind, placebo-controlled and active (adalimumab)-controlled period of the phase III trial SPIRIT-P1. Ann Rheum Dis 2017;76(1):79-87.

Ogdie A, Coates LC, Gladman DD. Treatment guidelines in psoriatic arthritis. Rheumatology (Oxford) 2020;59(Suppl 1):i37-i46.

Ogdie A, Yu Y, Haynes K, et al. Risk of major cardiovascular events in patients with psoriatic arthritis, psoriasis and rheumatoid arthritis: a population-based cohort study. Ann Rheum Dis 2015;74(2):326-32.

Papp KA, Weinberg MA, Morris A, et al. IL17A/F nanobody sonelokimab in patients with plaque psoriasis: a multicentre, randomised, placebo-controlled, phase 2b study. Presented at Elsevier. The Lancet 2021;397(10284):1564-75.

[redacted]

Raychaudhuri SP, Raychaudhuri SK, Genovese MC. IL-17 receptor and its functional significance in psoriatic arthritis. Mol Cell Biochem 2012;359(1-2):419-29.

Ritchlin CT, Colbert RA, Gladman DD. Psoriatic Arthritis. N Engl J Med 2017;376(10):957-70.

Ritchlin CT, Kavanaugh A, Merola JF, et al. Bimekizumab in patients with active psoriatic arthritis: results from a 48-week, randomised, double-blind, placebo-controlled, dose-ranging phase 2b trial. Lancet 2020;395(10222):427-40.

Sampson HA, Muñoz-Furlong A, Campbell RL, et al. Second symposium on the definition and management of anaphylaxis: summary report--Second National Institute of Allergy and Infectious

Disease/Food Allergy and Anaphylaxis Network symposium. J Allergy Clin Immunol 2006;117(2):391-7.

Schett G, Sticherling M, Neurath MF. COVID-19: risk for cytokine targeting in chronic inflammatory diseases? Nat Rev Immunol 2020;20(5):271-2.

[redacted]

Scotti L, Franchi M, Marchesoni A, et al. Prevalence and incidence of psoriatic arthritis: a systematic review and meta-analysis. Semin Arthritis Rheum 2018;48(1):28-34.

Singh JA, Guyatt G, Ogdie A, et al. Special Article: 2018 American College of Rheumatology/National Psoriasis Foundation Guideline for the Treatment of Psoriatic Arthritis. Arthritis Rheumatol 2019;71(1):5-32.

Clinical Study Report, M1095 Phase 2b psoriasis study. AV002, a Phase 2b randomized, double-blind, placebo-controlled, multi-center 12-week study with an additional 40-week follow-up assessment of efficacy, safety and tolerability of M1095 in subjects with moderate to severe chronic plaquetype psoriasis (M1095-PSO-201). MoonLake Immunotherapeutics; 2020. Document Control No.

Sun S, Ding Z, Yang X, et al. Nanobody: A Small Antibody with Big Implications for Tumor Therapeutic Strategy. Int J Nanomedicine 2021;16:2337-56.

Svecova D, Lubell MW, Casset-Semanaz F, et al. A randomized, double-blind, placebo-controlled phase 1 study of multiple ascending doses of subcutaneous M1095, an anti-interleukin 17A/F nanobody, in moderate-to-severe psoriasis. J Am Acad Dermatol 2019;81(1):196-203.

Taylor W, Gladman D, Helliwell P, et al. Classification criteria for psoriatic arthritis: development of new criteria from a large international study. Arthritis Rheum 2006;54(8):2665-73.

Tsukazaki H, Kaito T. The role of the IL-23/IL-17 pathway in the pathogenesis of spondyloarthritis. Int J Mol Sci 2020;21(17).

Villani AP, Rouzaud M, Sevrain M, et al. Prevalence of undiagnosed psoriatic arthritis among psoriasis patients: Systematic review and meta-analysis. J Am Acad Dermatol 2015;73(2):242-8.

Ware J, Snow K, Kosinski M, et al. SF36 Health Survey: Manual and interpretation guide. Lincoln, RI: Quality Metric Incorporated; 1993. (Available upon request)

Webster K, Cella D, Yost K. The Functional Assessment of Chronic Illness Therapy (FACIT) Measurement System: properties, applications, and interpretation. Health Qual Life Outcomes 2003;1:79.

Yellen SB, Cella DF, Webster K, et al. Measuring fatigue and other anemia-related symptoms with the Functional Assessment of Cancer Therapy (FACT) measurement system. J Pain Symptom Manage 1997;13(2):63-74.

21 APPENDICES

21.1 Appendix 1 ClASsification criteria for Psoriatic ARthritis (CASPAR)

Classification Criteria for Psoriatic Arthritis

CASPAR [Taylor W. et al., 2006] will be assessed by the investigator or a qualified designee at the Screening Visit indicated in the SOA in Section 3, Table 1 to determine eligibility.

To meet the CASPAR criteria, a participant must have inflammatory articular disease (joint, spine, or entheseal) with ≥3 points from the following 5 categories (CASPAR criteria have specificity of 98.7% and sensitivity of 91.4%):

1. Evidence of current PsO, a personal history of PsO, or a family history of PsO:
   - Current PsO is defined as psoriatic skin or scalp disease present today as judged by a rheumatologist or dermatologist (Current PsO is assigned a score of 2; all other features are assigned a score of 1);
   - A personal history of PsO is defined as a history of PsO that may be obtained from a patient, family physician, dermatologist, rheumatologist, or other qualified health care provider;
   - A family history of PsO is defined as a history of PsO in a first- or second-degree relative according to patient report.
2. Typical psoriatic nail dystrophy including onycholysis, pitting, and hyperkeratosis observed on current physical examination;
3. A negative test result for the presence of RF by any method except latex but preferably by enzyme-linked immunosorbent assay or nephelometry, according to the local laboratory reference range;
4. Either current dactylitis, defined as swelling of an entire digit, or a history of dactylitis recorded by a rheumatologist;
5. Radiographic evidence of juxta-articular new bone formation, appearing as ill-defined ossification near joint margins (but excluding osteophyte formation) on plain radiographs of the hand or foot.

21.2 Appendix 2 Women of Childbearing Potential and Highly Effective Contraception

Women of Childbearing Potential

Women of childbearing potential are defined as:

- Having experienced menarche; and
- Are not postmenopausal (as defined below); and
- Are not permanently surgically sterilized (as defined below); and
- Are not otherwise incapable of pregnancy.

Women of Non-childbearing Potential

Women of non-childbearing potential are defined as at least 1 of the following:

- Postmenopausal, defined as either:
  - >55 years of age with amenorrhea for at least 12 months without an alternative medical cause;

  OR

  - ≤55 years of age with amenorrhea for at least 12 months without an alternative medical cause and a serum follicle stimulating hormone (FSH) level >40 IU/L.
- Permanently surgically sterilized (e.g., tubal occlusion, hysterectomy, bilateral oophorectomy or bilateral salpingectomy);
- Otherwise incapable of pregnancy.

Highly Effective Contraception

Women of childbearing potential must be practicing a highly effective (failure rate of <1% per year when used consistently and correctly) method of birth control, prior to receiving study treatment, during the study, and for at least 12 weeks after receiving the last dose of study treatment, consistent with local regulations regarding the use of birth control methods for participants participating in clinical studies. Highly effective methods include:

- Established use of oral, patch, injected, or implanted hormonal methods of contraception;
- Placement of an intrauterine device or intrauterine system;
- Barrier methods including a male condom plus spermicidal foam/gel/film/cream/suppository (if available in their locale) or an occlusive cap (diaphragm or cervical/vault caps) plus spermicidal foam/gel/film/cream/suppository (if available in their locale);

- Male partner sterilization (the vasectomized partner should be the sole partner for that participant);
- True abstinence (when this is in line with the preferred and usual lifestyle of the participant).

  Note: If a female participant's childbearing potential changes after start of the study (e.g., a woman who is not heterosexually active becomes active), she must begin practicing a highly effective method of contraception, as described above.

Male Contraception

Male participants must be willing to use a condom when sexually active with a partner of childbearing potential during the study and for 12 weeks after the last dose of study treatment, unless permanently surgically sterile.

21.3 Appendix 3 SARS-CoV-2 (COVID-19) Guidance

Inhibitors of the IL-17 pathway and TNF-alpha inhibitors have not been associated with an increased susceptibility to Severe Acute Respiratory Syndrome Coronavirus 2 (SARS-CoV-2) infection [Schett G. et al., 2020].

Investigators and designees are to comply with local guidelines and regulations regarding monitoring participants for COVID-19 infections, management of participants who test positive for SARS-CoV-2, and minimizing the spread of COVID-19.

Vaccination status of participants will be recorded at screening. This includes the number and type of vaccines given.

Participants requiring vaccinations during the study period should receive them. If possible, the baseline assessments (Day 1/Week 0) should not be within 2 weeks of administration of SARS-CoV-2 vaccination. Vaccinations given during the study will ideally be planned to be administered in the middle of a dose interval. All vaccinations should be captured as concomitant medications.

Participants should be monitored for possible signs of COVID-19. In case of a newly diagnosed SARS-CoV-2 infection, local regulations and guidelines including times of quarantine will be adhered to. Participants may therefore miss a scheduled visit (Section 8.6). Participants may resume the study if asymptomatic and following a negative test (PCR or antigen), if the provisions in Section 8.6 are observed.

21.4 Appendix 4 Clinical Criteria for Diagnosing Anaphylaxis

Anaphylaxis is highly likely when any one of the following 3 criteria from the Second Symposium on the Definition and Management of Anaphylaxis [Sampson H.A. et al., 2006] are fulfilled:
1. Acute onset of an illness (minutes to several hours) with involvement of the skin, mucosal tissue, or both (e.g., generalized hives, pruritus, or flushing, swollen lips-tongue-uvula);
AND AT LEAST ONE OF THE FOLLOWING:
a. Respiratory compromise (e.g., dyspnea, wheeze-bronchospasm, stridor, reduced PEF, hypoxemia);
b. Reduced BP or associated symptoms of end-organ dysfunction (e.g., hypotonia [collapse], syncope, incontinence).
2. Two or more of the following that occur rapidly after exposure to a likely allergen for that patient (minutes to several hours):
a. Involvement of the skin-mucosal tissue (e.g., generalized hives, itch-flush, swollen lips-tongue-uvula);
b. Respiratory compromise (e.g., dyspnea, wheeze-bronchospasm, stridor, reduced PEF, hypoxemia);
c. Reduced BP or associated symptoms (e.g., hypotonia [collapse], syncope, incontinence);
d. Persistent gastrointestinal symptoms (e.g., crampy abdominal pain, vomiting).
3. Reduced BP after exposure to known allergen for that patient (minutes to several hours):
a. Infants and children: low systolic BP (age specific) or greater than 30% decrease in systolic BP.[a]
b. Adults: systolic BP of less than 90 mmHg or greater than 30% decrease from that person's baseline.

Abbreviations: BP = blood pressure; PEF = Peak expiratory flow.

a Low systolic BP for children is defined as less than 70 mmHg from 1 month to 1 year, less than (70 mmHg + [2 × age]) from 1 to 10 years, and less than 90 mmHg from 11 to 17 years.

**Protocol Title:**	Phase 2, Randomized, Parallel-group, Double-blind, Placebo-controlled Study of Sonelokimab in Patients with Active Psoriatic Arthritis
**Protocol Number:**	M1095-PSA-201
**Protocol Version, Date**	Final v1.2, 12 Jul 2022

[redacted]

**Document Version, Date:**	Final 1.2, 05FEB2024

Prepared by:

[redacted]

On behalf of:

MoonLake Immunotherapeutics AG

Confidentiality statement:

- The information provided in this document is strictly confidential.
- The recipients of the SAP must not disclose the confidential information contained within this document or any related information to other persons without the permission of the sponsor.
- In addition, the recipients of the SAP must keep this confidential document in a controlled environment which prevents unauthorized access to the document.

DocUUID : 3213b5be-8d63-4760-bea6-7e3ec175e71e

SIGNATURE PAGE

[redacted]

DocUUID : 3213b5be-8d63-4760-bea6-7e3ec175e71e

[redacted]

DocUUID : 3213b5be-8d63-4760-bea6-7e3ec175e71e

TABLE OF CONTENTS

SIGNATURE PAGE 2
REVISION HISTORY 3
TABLE OF CONTENTS 5
LIST OF ABBREVIATIONS 12
1 INTRODUCTION 15
2 STUDY OBJECTIVES 16
2.1 Primary Objective 16
2.2 Secondary Objectives 16
2.3 Exploratory Objective 16
3 STUDY DESIGN 17
3.1 General Study Design 17
3.2 Randomization and Blinding 19
3.2.1 Randomization – Part A 19
3.2.2 Treatment allocation – Part B 19
3.2.3 Maintenance of Blinding 20
3.3 Study Treatments and Assessments 20
3.3.1 Sonelokimab 21
3.3.2 Placebo 22
3.3.3 Adalimumab 22
4 STUDY ENDPOINTS 23
4.1 Primary Efficacy Endpoint 23
4.2 Secondary Efficacy Endpoints 23
4.3 Pharmacokinetic and Immunogenicity Endpoints 25
4.4 Exploratory Endpoint 25
4.5 Safety Endpoints 25
5 STUDY ESTIMANDS 26
5.1 Estimand for the Primary Endpoint 26
5.2 Sensitivity Estimand for the Primary Endpoint 27
5.3 Estimand for the Key Secondary Endpoints 28
5.4 Sensitivity Estimand for the Key Secondary Endpoints 29
5.5 Sample Size and power 32
6 ANALYSIS POPULATIONS 33
6.1 Part A 33
6.1.1 Enrolled Set 33
6.1.2 Safety Analysis Set (SAF) 33
6.1.3 Full Analysis Set (FAS) 33
6.1.4 Immunogenicity Analysis Set (IG Analysis Set) 33
6.1.5 Pharmacokinetic Analysis Set (PK Analysis Set) 33
6.2 Part B 33
6.2.1 Safety Analysis Set (SAF-B) 33
6.2.2 Full Analysis Set (FAS-B) 34
6.3 Protocol Deviations/Violations and Exclusions from Analysis Sets 34
7 STATISTICAL CONSIDERATIONS AND ANALYSIS 35

DocUUID : 3213b5be-8d63-4760-bea6-7e3ec175e71e

7.1 Derived Variables 35
7.2 Visit Windows 36
7.3 Handling of Missing Data and Randomization Stratification Errors 39
7.3.1 Missing Data Analysis Methods 39
7.3.2 Handling of Missing or Incomplete Dates 40
7.3.3 Randomization Stratification Errors 42
8 STATISTICAL METHODS 43
8.1 General Statistical Conventions 43
8.2 Participant Disposition 44
8.3 Protocol Deviations 45
8.4 Demographics and Baseline Characteristics 45
8.4.1 Demographics 45
8.4.2 Baseline and Disease Characteristics 46
8.4.3 Medical History 47
8.4.4 Psoriatic Arthritis Previous Therapies 47
8.4.5 Inquiries for extra-musculoskeletal PsA manifestation 47
8.4.6 Previous X-ray images of hands and feet 48
8.4.7 Prior and Concomitant Medications 48
8.5 Extent of Exposure 48
8.5.1 Treatment Duration 48
8.5.2 Treatment Compliance 48
8.6 Efficacy Analyses 49
8.6.1 Analysis Methods 49
8.6.1.1 Logistic Regression Model 49
8.6.1.2 Mixed Model Repeated Measures 50
8.6.1.3 Multiple Imputation 50
8.6.1.4 Multiplicity 51
8.6.1.5 Treatment-by-Center Interaction Analysis (Multi-Center Study) 52
8.6.2 Analysis of Primary Efficacy Endpoint 52
8.6.2.1 American College of Rheumatology 50 Clinical Response 52
8.6.2.2 TJC68 54
8.6.2.3 SJC66: 54
8.6.2.4 Health Assessment Questionnaire (HAQ-DI) 54
8.6.2.5 Patients Assessment of Arthritis pain 54
8.6.2.6 Patients Global Assessment of Disease Activity 54
8.6.2.7 Physician's Global Assessment of Disease Activity 55
8.6.2.8 Hs-CRP 55
8.6.2.9 ACR Components 55
8.6.3 Analysis of Key Secondary Efficacy Endpoints 55
8.6.3.1 American College of Rheumatology 20 Clinical Response 55
8.6.3.2 Psoriasis Area and Severity Index 90 56
8.6.4 Analysis of Other Secondary Efficacy Endpoints 57
8.6.4.1 American College of Rheumatology Clinical Response (ACR20/50) 57
8.6.4.2 American College of Rheumatology 70 Clinical Response (ACR70) 58
8.6.4.3 Psoriasis Area and Severity Index (PASI75/90/100) 58
8.6.4.4 Minimal Disease Activity 59

[redacted]

8.6.4.6 Leeds Enthesitis Index (LEI) and Spondyloarthritis Research Consortium of Canada Enthesitis Index (SPARCC) 60
8.6.4.7 Bath Ankylosing Spondylitis Disease Activity Index (BASDAI) 61
8.6.4.8 Psoriatic Assessment Impact of Disease-12 62
8.6.4.9 Leeds Dactylitis Index 63
8.6.4.10 Disease Activity Index for Psoriatic Arthritis 63
8.6.4.11 Modified Nail Psoriasis Severity Index 64
8.6.4.12 Functional Assessment of Chronic Illness Therapy-Fatigue (FACIT-Fatigue) 65
8.6.4.13 Short Form-36 Health Survey Version 2 65
[redacted]
8.6.4.15 ACR and PASI Composite Endpoints 68
8.7 Pharmacokinetic and Immunological Analyses 68
[redacted]
8.7.2 Pharmacokinetic Analyses 69
8.8 Analysis of Exploratory Endpoint 70
8.9 Safety Analyses 70
8.9.1 Adverse Events 71
8.9.2 Clinical Laboratory Evaluations 73
8.9.3 Vital Signs 73
8.9.4 Physical Examinations 74
8.9.5 Electrocardiograms 74
[redacted]
8.10 Subgroup Analysis 76
8.11 Interim Analysis 77
8.12 Primary Analysis Timing 77
8.13 Data Safety Monitoring Board 77
8.14 Development Safety Update Report 77
9 CHANGES TO PLANNED ANALYSIS FROM STUDY PROTOCOL 78
10 REFERENCES 79
11 APPENDICES 80
Appendix A 80
Appendix B 84
Appendix C 85
Appendix D 86
Appendix E 87
Appendix F 88
Appendix G 92
Appendix H 93
Appendix I 95
Appendix J 97
Appendix K 99
Appendix L 100
Appendix M 101

DocUUID : 3213b5be-8d63-4760-bea6-7e3ec175e71e

LIST OF ABBREVIATIONS

The following abbreviations will be used within this SAP.

Abbreviation	Explanation
ACR	American College of Rheumatology
ACR50	Response rate of participants achieving at least a 50% improvement in the American College of Rheumatology criteria
ACR20	Response rate of participants achieving at least a 20% improvement in the American College of Rheumatology criteria
ACR70	Response rate of participants achieving at least a 70% improvement in the American College of Rheumatology criteria
ADA	Anti-Drug Antibodies
AE	Adverse Event
AESI	Adverse Events of Special Interest
AN	Abscess and inflammatory nodule
ANCOVA	Analysis of Covariance
ATC	Anatomical Therapeutic Chemical
BASDAI	Bath Ankylosing Spondylitis Disease Activity Index
BLQ	Below the Quantifiable Limit
BMI	Body Mass Index
BP	Blood Pressure
BSA	Body Surface Area
CASPAR	Classification criteria for Psoriatic Arthritis
CI	Confidence Interval
COVID-19	Coronavirus disease 2019
CRF	Case Report Form
CRO	Contract Research Organization
CRP	C-Reactive Protein
CSR	Clinical Study Report
[redacted]	
CV	Coefficient of Variation
DAPSA	Disease Activity Index for Psoriatic Arthritis
DBP	Diastolic Blood Pressure
DMARD	Disease-Modifying Anti-Rheumatic Drug
DSMB	Data Safety Monitoring Board
DSUR	Development Safety Update Report
ECG	Electrocardiogram

EOT	End of Treatment
FACIT-F	Functional Assessment of Chronic Illness Therapy-Fatigue
FAS	Full Analysis Set
GLMM	Generalized linear mixed model
GM	Geometric Mean
HAQ-DI	Health Assessment Questionnaire Disability Index
hs-CRP	high sensitivity C-reactive protein
ICH	International Conference on Harmonization
IG	Immunogenicity
IHS4	International Hidradenitis Suppurativa severity score system
IMP	Investigational Medicinal Product
IRT	Interactive Response Technology
ITT	Intention-to-Treat
KM	Kaplan-Meier
LDI	Leeds Dactylitis Index
LEI	Leeds Enthesitis Index
LS	least squares
MAR	Missing at Random
max	Maximum
MCS	Mental Component Summary
MedDRA	Medical Dictionary for Regulatory Activities
min	Minimum
MMRM	Mixed Model for Repeated Measures
mNAPSI	Modified Nail Psoriasis Severity Index
nBLQ	Number of Concentrations Below the Quantifiable Limit
NCI CTCAE	National Cancer Institute Common Terminology Criteria for Adverse Events
NLR	Neutrophil to Lymphocyte Ratio
NRS	Numerical Rating Scale
OR	Odds Ratio
PASI	Psoriasis Area and Severity Index
PCR	Polymerase Chain Reaction
PCS	Physical Component Summary
PDCF	Protocol Deviation Criteria Form
PhGADA	Physician's Global Assessment of Disease Activity

DocUUID : 3213b5be-8d63-4760-bea6-7e3ec175e71e

[redacted]

PK	Pharmacokinetic
PsA	Psoriatic arthritis
PsAID	Psoriatic Arthritis Impact of Disease
PsO	Psoriasis
PT	Preferred Term
PtAAP	Patient's Assessment of Arthritis Pain
PtGADA	Patient's Global Assessment of Disease Activity
Q2W	once every 2 weeks
Q4W	once every 4 weeks
SAE	Serious Adverse Event
SAF	Safety analysis set
SAP	Statistical Analysis Plan
SBP	Systolic Blood Pressure
SC	Subcutaneous
SD	Standard Deviation
SE	Standard Error
SF-36v2	Short-Form-36 Health Survey Questionnaire-Version 2
SJC	Swollen Joint Count
SOA	Schedule of Activities
SOC	System Organ Class
SPARCC	Spondyloarthritis Research Consortium of Canada
TE	Treatment-Emergent
TEAE	Treatment-Emergent Adverse Event
TFLs	Tables, Figures and Listings
TJC	Tender Joint Count
VAS	Visual Analog Scale
WHODDE	World Health Organization Drug Dictionary Enhanced

DocUUID : 3213b5be-8d63-4760-bea6-7e3ec175e71e

1 INTRODUCTION

The purpose of this Statistical Analysis Plan (SAP) is to provide detailed descriptions of the statistical methods, data derivations and data displays for study protocol M1095-PSA-201, Version Final 1.1, "Phase 2, randomized, parallel-group, double-blind, placebo-controlled study of sonelokimab in patients with active psoriatic arthritis (PsA)" dated 12 Jul 2022 for final analysis in support of the Clinical Study Report (CSR), as well as describing the outputs that will used for the sonelokimab Data Safety Monitoring Board (DSMB) and Development Safety Update Report (DSUR). The table of contents and templates for the Tables, Figures and Listings (TFLs) will be produced in a separate document.

Any deviations from this SAP will be described and justified in the CSR.

The preparation of this SAP is based on International Conference on Harmonization (ICH) E9 guidelines.

All data analyses and generation of TFLs will be performed using SAS 9.4® or higher.

DocUUID : 3213b5be-8d63-4760-bea6-7e3ec175e71e

2 STUDY OBJECTIVES

2.1 Primary Objective

The primary objective of this study is to evaluate the efficacy of 3 different dose regimens of sonelokimab (120 mg once every 2 weeks [Q2W], 60 mg Q2W and 60 mg once every 4 weeks [Q4W]) compared with placebo in the treatment of participants with active PsA.

2.2 Secondary Objectives

The secondary objectives of this study are as follows:

- To evaluate the safety and tolerability of 3 different dose regimens of sonelokimab (120 mg Q2W,60 mg Q2W and 60 mg Q4W) compared with placebo in the treatment of participants with active PsA.
- To assess the pharmacokinetics (PK) and immunogenicity (IG) of 3 different dose regimens of sonelokimab (120 mg Q2W, 60 mg Q2W and 60 mg Q4W) in the treatment of participants with active PsA.

2.3 Exploratory Objective

The exploratory objective is to assess the effect of 3 different dose regimens of sonelokimab (120 mg Q2W, 60 mg Q2W and 60 mg Q4W) on soluble biomarkers of inflammation.

DocUUID : 3213b5be-8d63-4760-bea6-7e3ec175e71e

3 STUDY DESIGN

3.1 General Study Design

This is a phase 2, multi-center, randomized, parallel-group, double-blind, placebo-controlled study evaluating the efficacy, safety, PK and IG of sonelokimab in participants with active PsA. This study also has an active reference arm of adalimumab treatment; however, no formal comparison of sonelokimab vs adalimumab is planned.

For US participants, the study includes a screening period of up to 28 days, a 12-week treatment period (Part A) and an 8-week safety follow-up period after the administration of the last dose, which is scheduled to be administered at Week 10. An end of treatment (EOT) visit will be performed at Week 12. A safety follow-up visit will be performed 8 weeks (±7 days) after the administration of the last dose of study treatment. For non-US participants, the study will include screening period of up to 28 days, a treatment period of up to 24 weeks, and a safety follow-up period of 8 weeks after the last dose of study treatment. The treatment period will be divided into 2 parts: Part A (from baseline to Week 12) and Part B (from Week 12 to Week 24). The overall treatment period (Part A+B) will be from baseline to Week 24. The overall treatment period (Part A+B) will be from baseline to Week 24. The final dose of study treatment is scheduled to occur at Week 22. An End of Treatment (EOT) visit will be performed at Week 24. A safety follow-up visit will be performed 8 weeks (±7 days) after the administration of the final dose of study treatment.

On the first day of the Part A (Day 1/Week 0), eligible participants will be randomized 1:1:1:1:1 to one of 5 treatment arms as described in Figure 1.

Randomization will be stratified by sex (Male/Female) and exposure to biologic agents prior to the Screening Visit (Yes/No). The total number of participants with previous use of biologic agents will be capped at 30%.

Dosing in the sonelokimab arms will continue through Week 8. Adalimumab will be given through Week 10. All treatment arms will receive placebo as required to maintain the blinding of the study. The Part A treatment period will end at Week 12 when the primary efficacy analysis will be performed, comparing each of the sonelokimab treatment arms (sonelokimab 120 mg Q2W, sonelokimab 60 mg Q2W, sonelokimab 60 mg Q4W) vs placebo.

At the beginning of Part B (Week 12) TJC68 and SJC66 response will be assessed in all participants. A responder is defined as a participant who achieves at least a 20% reduction in each of the TJC68 and SJC66 assessments at Week 12 compared with baseline. A non-responder is defined as a participant who does not achieve at least a 20% reduction in each of the TJC68 and SJC66 assessments at Week 12 compared with baseline.

In Part B participants will be allocated to treatment based upon response at Week 12 via the Interactive Response Technology (IRT). Treatment assignments in Part A and the allocation for Part B by responders and non-responders at Week 12 are described in Figure 2 and Table 1.

On an ongoing basis, safety data will be reviewed by an independent DSMB

DocUUID : 3213b5be-8d63-4760-bea6-7e3ec175e71e

The study flow chart is presented in Figure 1 and Figure 2.

Figure 1: Study Flow Chart – Part A

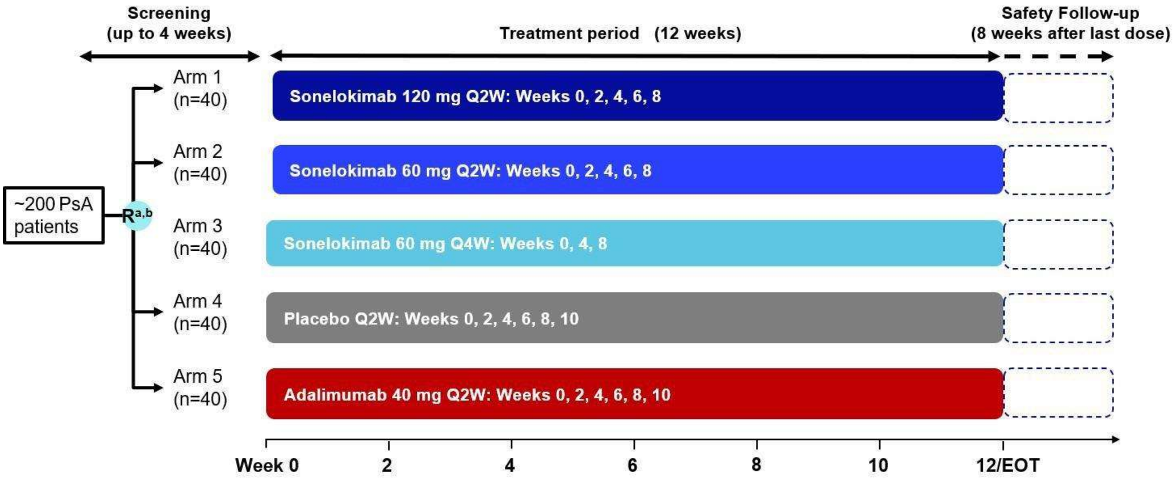

Abbreviations: EOT = End of Treatment; PsA = psoriatic arthritis; Q2W = once every 2 weeks; Q4W = once every 4 weeks; R = randomization; SFU = Safety Follow-Up.

a Randomization stratified by sex (Male/Female) and prior exposure to biologic agents (Yes/No).

b At the beginning of the treatment period at Week 0/Day 1, all eligible participants will be randomized 1:1:1:1:1.

DocUUID : 3213b5be-8d63-4760-bea6-7e3ec175e71e

Figure 2: Study Flow Chart – Part A + B

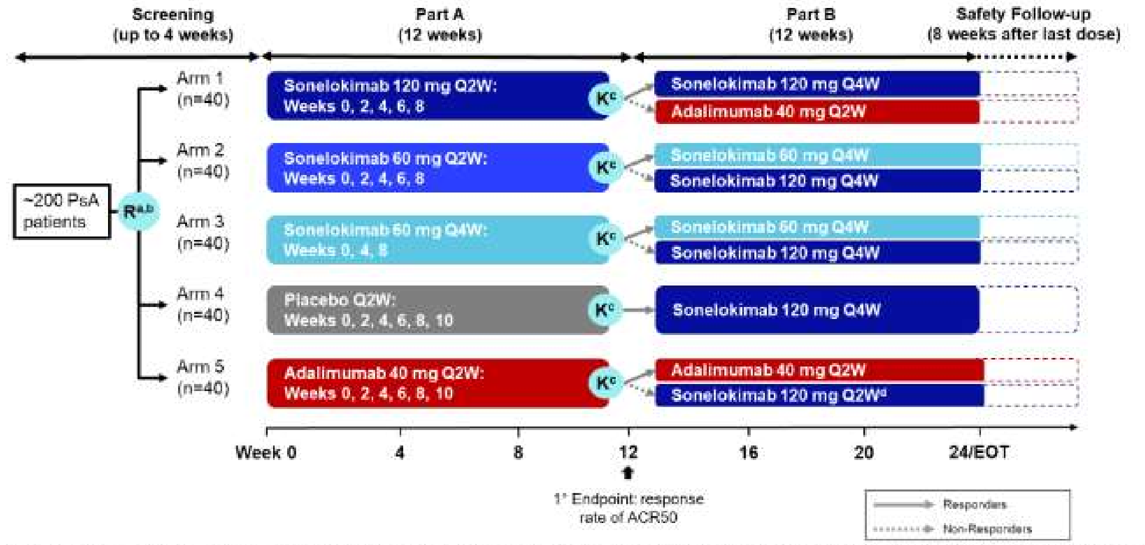

Abbreviations: ACR50 = response rate of at least 50% improvement in the American College of Rheumatology criteria; EOT = End-of-Treatment; IRT = Interactive Response Technology; K = crossover; PBO = placebo; PsA = psoriatic arthritis; Q2W = once every 2 weeks; Q4W = once every 4 weeks; R = randomization; SC = subcutaneous; SJC = swollen joint count; TJC = tender joint count.

a Randomization stratified by sex (Male/Female) and prior exposure to biologic agents (Yes/No).

b At the beginning of the treatment period at Week 0/Day 1, all eligible participants will be randomized 1:1:1:1:1.

c A responder is defined as a participant who achieves at least a 20% reduction in each of the TJC68 and SJC66 assessments at Week 12 compared with baseline. A non-responder is defined as a participant who does not achieve at least a 20% reduction in each of the TJC68 and SJC66 assessments at Week 12 compared with baseline. Treatment assignments in Part B will be allocated via the IRT based upon response at Week 12 as indicated in the study design diagram.

d In Part B non-responders in Arm 5 will receive sonelokimab 120 mg Q2W with SC injections at Weeks 12, 14, 16, 18, and 20.

3.2 Randomization and Blinding

3.2.1 Randomization – Part A

Randomization will take place on Day 1/Week 0 after confirmation that the participant continues to meet the inclusion/exclusion criteria. Participants will be randomized 1:1:1:1:1 to one of 5 treatment arms, stratified by sex and exposure to biologic agents prior to the screening visit.

The IRT will be responsible for the allocation of unique identification numbers to individual participants.

3.2.2 Treatment allocation – Part B

At the beginning of Part B (Week 12) TJC68 and SJC66 response will be assessed in all participants. A responder is defined as a participant who achieves at least a 20% reduction in each of the TJC68 and SJC66 assessments at Week 12 compared with baseline. A non-responder is defined as a participant who does not achieve at least a 20% reduction in each of the TJC68 and SJC66 assessments at Week 12 compared with baseline.

In Part B participants will be allocated to treatment based upon response at Week 12 via the IRT.

In Part B, the participants and investigators will remain double-blinded to participant treatment. Blinding of participants and investigators will be maintained in the same manner as in Part A.

DocUUID : 3213b5be-8d63-4760-bea6-7e3ec175e71e

3.2.3 Maintenance of Blinding

The blind will be maintained by the use of sonelokimab and matched placebo. The prefilled syringes used for all sonelokimab and placebo doses will be identical in appearance. The adalimumab injector will be different in appearance from syringes used for sonelokimab and placebo. However, the study will be blinded at the carton level. The outer packaging will be labelled in a blinded manner with no indication of the syringe contents (sonelokimab, placebo or adalimumab). Participants will be asked to wear an eye mask for all injections.

The primary analysis of 12-week data will occur at the completion of Part A while the study is still ongoing. All sponsor personnel will remain blinded until Week 12. No unblinded participant-level data will be supplied to the study sites until the final database lock after the conclusion of the study globally.

A DSMB will be setup to review the benefit-risk profile of sonelokimab and to independently safeguard the interests of participants in the studies and to enhance the integrity and credibility of the study. Members of the DSMB will be unblinded to study treatment as will the contract research organization (CRO) unblinded statistician and programmer to support the generation of unblinded outputs for the DSMB review. To maintain the blind, the CRO unblinded statistician and programmer will work in a secure environment not accessible to the CRO study team.

Laboratory personnel performing the bioanalytical PK sample analysis may receive an open randomization list to enable analysis of relevant samples. In addition, a PK analyst and modeling/simulation scientist may receive an open randomization list to enable preparation of modeling/simulation activities. These individuals will not interact with site or CRO personnel.

3.3 Study Treatments and Assessments

For the US participants, the planned study duration for individual participants will be up to 22 weeks, including a screening period of up to 4 weeks, a 12-week treatment period and a 8-week safety follow-up period after the administration of the last dose, which is scheduled to be administered at Week 10.

For the Non-US participants, the planned study duration for individual participants will be up to 34 weeks, including a screening period of up to 4 weeks, a 24-week treatment period, and an 8-week safety follow-up period after the administration of the last dose, which is scheduled to be administered at Week 22.

Randomized treatment assignment in Part A are:

- Arm 1: Sonelokimab 120 mg Q2W,
- Arm 2: Sonelokimab 60 mg Q2W,
- Arm 3: Sonelokimab 60 mg Q4W,
- Arm 4: Placebo,
- Armm 5: Adalimumab 40 mg Q2W.

Treatment Allocation in Part B Following Response Assessment at Week 12 are:

Table 1: Treatment Allocation in Part A+B

DocUUID : 3213b5be-8d63-4760-bea6-7e3ec175e71e

Treatment Assignment in Part A	Treatment Allocation in Part B (Based Upon Response at Week 12)	
	Responder	Non-responder
Arm 1: Sonelokimab 120 mg Q2W	Sonelokimab 120 mg Q4W	Adalimumab 40 mg Q2W
Arm 2: Sonelokimab 60 mg Q2W	Sonelokimab 60 mg Q4W	Sonelokimab 120 mg Q4W
Arm 3: Sonelokimab 60 mg Q4W	Sonelokimab 60 mg Q4W	Sonelokimab 120 mg Q4W
Arm 4: Placebo	Sonelokimab 120 mg Q4W	Sonelokimab 120 mg Q4W
Arm 5: Adalimumab 40 mg Q2W	Adalimumab 40 mg Q2W	Sonelokimab 120 mg Q2W

3.3.1 Sonelokimab

Sonelokimab drug product is supplied as a fixed-dose, prefilled syringe to be administered via subcutaneous (SC) injection. Syringes and cartons will be supplied in a blinded manner.

Sonelokimab will be administered by a site-approved, study treatment administrator, who is unblinded. Blinded study personnel will not be in attendance while study treatment is being prepared or administered.

In Part A, the 3 sonelokimab treatment arms are as follows:

- Arm 1 (sonelokimab 120 mg Q2W): Sonelokimab 120 mg will be given as SC injection at Weeks 0, 2, 4, 6 and 8. Participants will receive a placebo injection at Week 10 to maintain the blind;
- Arm 2 (sonelokimab 60 mg Q2W): Sonelokimab 60 mg will be given as SC injection at Weeks 0, 2, 4, 6 and 8. Participants will receive a placebo injection at Week 10 to maintain the blind;
- Arm 3 (sonelokimab 60 mg Q4W): Sonelokimab 60 mg will be given as SC injection at Weeks 0, 4 and 8. Participants will receive a placebo injection at Weeks 2, 6 and 10 to maintain the blind.

In Part B, after allocation of responders and non-responders, the sonelokimab treatment arms are as follows:

- Responders from Arm 1, non-responders from Arm 2, non-responders from Arm 3, and participants from Arm 4 will receive sonelokimab 120 mg Q4W until Week 20. Placebo injections will be given at Weeks 14, 18, and 22 to maintain the blind;
- Responders from Arm 2 and responders from Arm 3 will receive sonelokimab 60 mg Q4W until Week 20. Placebo injections will be given at Weeks 14, 18, and 22 to maintain the blind;

DocUUID : 3213b5be-8d63-4760-bea6-7e3ec175e71e

- Non-responders in Arm 5 will receive sonelokimab 120 mg Q2W with SC injections at Weeks 12, 14, 16, 18, and 20. Participants will receive a placebo injection at Week 22 to maintain the blind.

3.3.2 Placebo

Placebo will be identical in appearance and packaging to sonelokimab in a fixed-dose, prefilled syringe to be administered via SC injection.

As with the administration of sonelokimab, placebo will be administered by a site-approved, study treatment administrator, who is unblinded. Blinded study personnel will not be in attendance while study treatment is being prepared or administered.

During Part A, for participants randomized to Arm 4, placebo will be given SC every 2 weeks, with injections at Weeks 0, 2, 4, 6, 8 and 10.

3.3.3 Adalimumab

Adalimumab will be provided in prefilled syringes (40 mg) as sterile solution for SC injection. Adalimumab syringes will be relabelled and repackaged to facilitate maintenance of the study blinding.

Adalimumab will be administered by a site-approved, study treatment administrator, who is unblinded. Blinded study personnel will not be in attendance while study treatment is being prepared or administered.

During Part A, for participants randomized to Arm 5, adalimumab will be given as 40 mg SC every 2 weeks, with injections at Weeks 0, 2, 4, 6, 8 and 10 in accordance with the approved labelling.

In Part B, non-responders from Arm 1 and responders from Arm 5 will receive adalimumab 40 mg Q2W administered by SC injection at Weeks 12, 14, 16, 18, 20, and 22.

A detailed description of procedures and assessments to be conducted during this study is summarized in the Schedule of Activities (SOA) in Table 1 of the protocol.

DocUUID : 3213b5be-8d63-4760-bea6-7e3ec175e71e

4 STUDY ENDPOINTS

4.1 Primary Efficacy Endpoint

The primary efficacy endpoint of this study is response rate of at least 50% improvement in the American College of Rheumatology (ACR50) response criteria at Week 12 compared with baseline.

4.2 Secondary Efficacy Endpoints

The key secondary efficacy endpoints of this study are:

- ACR20 response rate at Week 12 compared with baseline;
- Psoriasis Area and Severity Index (PASI) 90 at Week 12 compared with baseline in the subgroup of participants with psoriasis (PsO) involving at least 3% body surface area (BSA) at baseline.

The other secondary endpoints this study are:

- Response rate compared with baseline at specified timepoints other than Week 12 for the following:
  - ACR20;
  - ACR50
  - PASI90.
- Response rate compared with baseline at specified timepoints for the following
  - ACR70;
  - Minimal disease activity at Week 12, defined as meeting 5 of the 7 following criteria:
    - Tender joint count (TJC) 68 ≤1;
    - Swollen joint count (SJC) 66 ≤1;
    - PASI ≤1 or PsO affecting ≤1% of BSA;
    - Patient's Assessment of Arthritis Pain (PtAAP) ≤15 on a 0 to 100 visual analog scale (VAS);
    - Patient's Global Assessment of Disease Activity (PtGADA) ≤20 on a 0 to 100 VAS;
    - Health Assessment Questionnaire Disability Index (HAQ-DI) ≤0.5;
    - Leeds Enthesitis Index (LEI) ≤1.
  - PASI75 at specified timepoints compared with baseline (in the subgroup of participants with PsO involving at least 3% BSA at baseline);
  - PASI100 at specified timepoints compared with baseline (in the subgroup of participants with PsO involving at least 3% BSA at baseline);

DocUUID : 3213b5be-8d63-4760-bea6-7e3ec175e71e

- Response rate of at least 50% improvement compared with baseline in the Bath Ankylosing Spondylitis Disease Activity Index (BASDAI);

[redacted]

- The change from baseline at specified timepoints for the following:
  - TJC68;
  - SJC66;
  - Enthesitis evaluation (LEI and Spondyloarthritis Research Consortium of Canada [SPARCC] enthesitis index);
  - Leeds Dactylitis Index (LDI);
  - Modified Nail Psoriasis Severity Index (mNAPSI);
  - High sensitivity C-reactive protein (hs-CRP);
  - PtGADA;
  - Physician's Global Assessment of Disease Activity (PhGADA);
  - Psoriatic Arthritis Impact of Disease (PsAID)-12;
  - PtAAP;
  - HAQ-DI;
  - Functional Assessment of Chronic Illness Therapy for Fatigue (FACIT-Fatigue);
  - Short-Form-36 Health Survey Questionnaire-Version 2 (SF-36v2) Mental Component Summary (MCS);
  - SF-36v2 Physical Component Summary (PCS);
  - BASDAI;

[redacted]

DocUUID : 3213b5be-8d63-4760-bea6-7e3ec175e71e

[redacted]

4.3 Pharmacokinetic and Immunogenicity Endpoints

The PK and IG endpoints of this study are:

- PK of sonelokimab (trough levels);
- Anti-drug antibodies (ADAs).

[redacted]

4.5 Safety Endpoints

The safety endpoints of this study are:

- Incidence, relatedness, severity and seriousness of adverse events (AEs);
- Withdrawal due to AEs;
- Clinically relevant abnormalities in vital signs (blood pressure [BP] and heart rate) and body weight;
- Standard 12-lead electrocardiogram (ECG) intervals (RR, PR, QRS, QT and QT intervals corrected for heart rate using Bazett's and Fridericia's formulas [QTcB and QTcF, respectively]) including clinically relevant abnormalities in ECG variables;
- Clinically relevant abnormalities in clinical laboratory variables (hematology, biochemistry, and urinalysis).

DocUUID : 3213b5be-8d63-4760-bea6-7e3ec175e71e

5 STUDY ESTIMANDS

5.1 Estimand for the Primary Endpoint

Attribute	Details
Treatments	Sonelokimab at 3 different doses:<br>• sonelokimab 120 mg Q2W or<br>• sonelokimab 60 mg Q2W or<br>• sonelokimab 60 mg Q4W
Target Population	• Adults with a confirmed diagnosis of PsA per the 2006 CASPAR [Taylor W. et al., 2006] with symptoms for ≥6 months prior to the Screening Visit<br>• Analysis Set: Full Analysis Set (FAS)
Endpoint	Percentage of participants achieving ACR50 at Week 12 compared with baseline.
Population-level summary	The primary estimator will be the improvement compared to placebo over the 12-week based on ACR50.
Intercurrent events and strategies to handle those	• Use of prohibited concomitant medication (Section 10.5.1 of protocol): participants will be analyzed as a treatment failure after taking prohibited medication, i.e., non-responder imputation will be performed after the first episode of prohibited medication for all future assessments (composite strategy);<br>• Withdrawal from treatment due to any reason (Section 9.6 of protocol): participants will be considered non-responders to treatment if they discontinue the study prior to the Week 12 (composite strategy);<br>• Study treatment non-adherence (missing doses) or drug administration error: participants will be analyzed in the same way as if the event had occurred or not (treatment policy strategy).

DocUUID : 3213b5be-8d63-4760-bea6-7e3ec175e71e

5.2 Sensitivity Estimand for the Primary Endpoint

The first sensitivity estimand will include data from participants in the target population of the primary estimand plus data collected after the withdrawal of study treatment through Week 12 from participants who discontinue study treatments prior to Week 12 for reasons other than lack of efficacy or response.

Attribute	Details
Treatments	Sonelokimab at 3 different doses:<br>• sonelokimab 120 mg Q2W or<br>• sonelokimab 60 mg Q2W or<br>• sonelokimab 60 mg Q4W
Target Population	• Adults with a confirmed diagnosis of PsA per the 2006 CASPAR [Taylor W. et al., 2006] with symptoms for ≥6 months prior to the Screening Visit<br>• Analysis Set: Full Analysis Set (FAS)
Endpoint	– Percentage of participants achieving ACR50, i.e., at least 50% improvement in the ACR response criteria at Week 12 compared with baseline.
Intercurrent events and strategies to handle those	• Use of prohibited concomitant medication (Section 10.5.1 of protocol): participants will be analyzed as a treatment failure after taking prohibited medication, i.e., non-responder imputation will be performed after the first episode of prohibited medication for all future assessments (composite strategy);<br>• Withdrawal from treatment due to lack of efficacy or adverse event (Section 9.6 of protocol): participants will be considered non-responders to treatment if they discontinue the study prior to the Week 12 (composite strategy);<br>• Withdrawal from treatment due to any reason other than lack of efficacy or adverse event (Section 9.6 of protocol): participants will continue to be followed and use their observed Week 12 Visit (treatment policy strategy). Any missing data for participants who do not have a Week 12 visit will be assumed to be missing at random;<br>• Study treatment non-adherence (missing doses) or drug administration error: participants will be analyzed in the same

DocUUID : 3213b5be-8d63-4760-bea6-7e3ec175e71e

	way as if the event had occurred or not (treatment policy strategy).

The second sensitivity estimand will include data from participants in the target population of the primary estimand targeting the treatment policy estimand such that the difference in response between treatments are analysed regardless of prohibited medication and withdrawal from treatment.

**Attribute**	**Details**
Treatments	Sonelokimab at 3 different doses:<br>• sonelokimab 120 mg Q2W or<br>• sonelokimab 60 mg Q2W or<br>• sonelokimab 60 mg Q4W
Target Population	• Adults with a confirmed diagnosis of PsA per the 2006 CASPAR [Taylor W. et al., 2006] with symptoms for ≥6 months prior to the Screening Visit<br>• Analysis Set: Full Analysis Set (FAS)
Endpoint	– Percentage of participants achieving ACR50, i.e., at least 50% improvement in the ACR response criteria at Week 12 compared with baseline.
Intercurrent events and strategies to handle those	• Use of prohibited concomitant medication (Section 10.5.1 of protocol): observed data after taking prohibited medication will used in the analysis (treatment policy strategy); Any missing data for participants who do not have a Week 12 visit will be assumed to be missing at random;<br>• Withdrawal from treatment for any reason (Section 9.6 of protocol): participants will continue to be followed and use their observed Week 12 Visit (treatment policy strategy). Any missing data for participants who do not have a Week 12 visit will be assumed to be missing at random;<br>• Study treatment non-adherence (missing doses) or drug administration error: participants will be analyzed in the same way as if the event had occurred or not (treatment policy strategy).

5.3 Estimand for the Key Secondary Endpoints

DocUUID : 3213b5be-8d63-4760-bea6-7e3ec175e71e

Attribute	Details
Treatments	Sonelokimab at 3 different doses:<br>• sonelokimab 120 mg Q2W or<br>• sonelokimab 60 mg Q2W or<br>• sonelokimab 60 mg Q4W
Target Population	• Adults with a confirmed diagnosis of PsA per the 2006 CASPAR [Taylor W. et al., 2006] with symptoms for ≥6 months prior to the Screening Visit;<br>• Analysis Set: FAS
Endpoint	The key secondary endpoints are as follows:<br>• ACR20 response rate at Week 12 compared with baseline;<br>• PASI90 at Week 12 compared with baseline in the subgroup of participants with PsO involving at least 3% BSA at baseline.
Population-level summary	The primary estimator will be the improvement compared to placebo over the 12-week based on the endpoint of interest.
Intercurrent events and strategies to handle those	• Use of prohibited concomitant medication (Section 10.5.1 of protocol): participants will be analyzed as a treatment failure after taking prohibited medication, i.e., non-responder imputation will be performed after the first episode of prohibited medication for all future assessments (composite strategy);<br>• Withdrawal from treatment due to any reason (Section 9.6 of protocol): participants will be considered non-responders to treatment if they discontinue the study prior to the Week 12 visit due to any reason (composite strategy);<br>• Study treatment non-adherence (missing doses) or drug administration error: participants will be analyzed in the same way as if the event had occurred or not (treatment policy strategy).

5.4 Sensitivity Estimand for the Key Secondary Endpoints

The first sensitivity estimand will include data from participants in the target population of the key secondary estimand plus data collected after the withdrawal of study treatment through Week 12 from

DocUUID : 3213b5be-8d63-4760-bea6-7e3ec175e71e

participants who discontinue study treatments prior to Week 12 for reasons other than lack of efficacy or response.

Attribute	Details
Treatments	Sonelokimab at 3 different doses:<br>• sonelokimab 120 mg Q2W or<br>• sonelokimab 60 mg Q2W or<br>• sonelokimab 60 mg Q4W
Target Population	• Adults with a confirmed diagnosis of PsA per the 2006 CASPAR [Taylor W. et al., 2006] with symptoms for ≥6 months prior to the Screening Visit;<br>• Analysis Set: FAS
Endpoint	The key secondary endpoints are as follows:<br>• ACR20 response rate at Week 12 compared with baseline;<br>• PASI90 at Week 12 compared with baseline in the subgroup of participants with PsO involving at least 3% BSA at baseline.
Intercurrent events and strategies to handle those	• Use of prohibited concomitant medication (Section 10.5.1 of protocol): participants will be analyzed as a treatment failure after taking prohibited medication, i.e., non-responder imputation will be performed after the first episode of prohibited medication for all future assessments (composite strategy);<br>• Withdrawal from treatment due to lack of efficacy or adverse event (Section 9.6 of protocol): participants will be considered non-responders to treatment if they discontinue the study prior to the Week 12 visit (composite strategy);<br>• Withdrawal from treatment due to any reason other than lack of efficacy or adverse event (Section 9.6 of protocol): participants will continue to be followed and use their observed Week 12 Visit (treatment policy strategy). Any missing data for participants who do not have a Week 12 visit will be assumed to be missing at random;<br>• Study treatment non-adherence (missing doses) or drug administration error: participants will be analyzed in the same

DocUUID : 3213b5be-8d63-4760-bea6-7e3ec175e71e

	way as if the event had occurred or not (treatment policy strategy).

The second sensitivity estimand will include data from participants in the target population of the primary estimand targeting the treatment policy estimand such that the difference in response between treatments are analysed regardless of prohibited medication and withdrawal from treatment.

**Attribute**	**Details**
Treatments	Sonelokimab at 3 different doses:<br>• sonelokimab 120 mg Q2W or<br>• sonelokimab 60 mg Q2W or<br>• sonelokimab 60 mg Q4W
Target Population	• Adults with a confirmed diagnosis of PsA per the 2006 CASPAR [Taylor W. et al., 2006] with symptoms for ≥6 months prior to the Screening Visit<br>• Analysis Set: Full Analysis Set (FAS)
Endpoint	The key secondary endpoints are as follows:<br>– ACR20 response rate at Week 12 compared with baseline;<br>– PASI90 at Week 12 compared with baseline in the subgroup of participants with PsO involving at least 3% BSA at baseline.
Intercurrent events and strategies to handle those	• Use of prohibited concomitant medication (Section 10.5.1 of protocol): observed data after taking prohibited medication will used in the analysis (treatment policy strategy); Any missing data for participants who do not have a Week 12 visit will be assumed to be missing at random;<br>• Withdrawal from treatment for any reason (Section 9.6 of protocol): participants will continue to be followed and use their observed Week 12 Visit (treatment policy strategy). Any missing data for participants who do not have a Week 12 visit will be assumed to be missing at random;<br>• Study treatment non-adherence (missing doses) or drug administration error: participants will be analyzed in the same

	way as if the event had occurred or not (treatment policy strategy).

5.5 Sample Size and power

The sample size calculation is based on the primary efficacy endpoint (i.e., ACR50 response rate at Week 12 compared with baseline) with the primary comparisons of interest being: sonelokimab 120 mg Q2W (Arm 1) vs placebo (Arm 4), sonelokimab 60 mg Q2W (Arm 2) vs placebo (Arm 4) and sonelokimab 60 mg Q4W (Arm 3) vs placebo (Arm 4) and determined based on assumed ACR50 response rates as follows:

- 40% with each sonelokimab dose regimen;
- 10% with placebo.

The estimated response rates for sample size were based on past IL-17 inhibitors for the treatment of participants with PsA. The Cosentyx FUTURE 2 study showed that ACR50 was achieved in 35% of secukinumab 300 mg and 37% of secukinumab 150 mg, vs 6% in the placebo arm [McInnes I.B. et al., 2015].

A sample size of n = 40 participants in each of the sonelokimab 120 mg Q2W, sonelokimab 60 mg Q2W, sonelokimab 60 mg Q4W and placebo arms (therefore, 160 participants in total) results in a power of more than 80%, while assuming an overall 2-sided alpha of 0.025, a placebo ACR50 response rate of 10% and a difference in ACR50 response rate of 30% between placebo and each sonelokimab dose regimen. An additional 40 participants will be randomized in the adalimumab arm. The adalimumab treatment will be used as an active reference arm, however, no formal comparison of sonelokimab or placebo vs adalimumab is planned.

Eligible participants will be randomized in a 1:1:1:1:1 ratio to sonelokimab 120 mg Q2W (Arm 1), sonelokimab 60 mg Q2W (Arm 2), sonelokimab 60 mg Q4W (Arm 3), placebo (Arm 4) or adalimumab 40 mg Q2W (Arm 5). The total proportion of participants with previous use of biologic agents will be capped at 30%.

Due to the use of the FAS with non-responder imputation, no accounting for dropout is required, therefore a total of approximately 200 participants will be randomized.

All calculations were performed in nQuery® Software version 8.5.1.0.

DocUUID : 3213b5be-8d63-4760-bea6-7e3ec175e71e

6 ANALYSIS POPULATIONS

6.1 Part A

6.1.1 Enrolled Set

The enrolled set is defined as all participants who signed an informed consent.

6.1.2 Safety Analysis Set (SAF)

The safety analysis set (SAF) is defined as all randomized participants who received at least one dose of study treatment. Participants will be analyzed based on the treatment that they actually received, regardless of the treatment they are randomized to.

The SAF will be used for all safety analyses related to the Part A and Part A+B.

6.1.3 Full Analysis Set (FAS)

The FAS is defined as all randomized participants. Participants will be analyzed in accordance with the intention-to-treat (ITT) principle, i.e., based on randomized treatment, irrespective of the treatment they actually received.

The FAS will be used for all efficacy analyses on efficacy endpoints related to the Part A and some Part A+B analyses and will serve as the primary analysis set.

6.1.4 Immunogenicity Analysis Set (IG Analysis Set)

The IG analysis set is defined as all participants who received at least one dose of sonelokimab and who have at least 1 sample obtained for the detection of antibodies to sonelokimab.

In the IG analyses, participants will be analyzed according to the treatment they actually received, regardless of the treatment they are randomized to.

The IG analysis set will be used for all immunogenicity analyses related to the Part A and A+B.

6.1.5 Pharmacokinetic Analysis Set (PK Analysis Set)

The PK analysis set is defined as all participants who received at least one complete dose of sonelokimab and had at least 1 valid blood sample drawn for PK analysis after their first dose of sonelokimab.

In the PK analyses, participants will be analyzed according to the treatment they actually received, regardless of the treatments they are randomized to.

The PK analysis set will be used for all PK analyses related to the Part A and A+B.

6.2 Part B

6.2.1 Safety Analysis Set (SAF-B)

The SAF-B is defined as all randomized participants who received at least one dose of study treatment during Part B. Participants will be analyzed based on the treatment that they actually received, regardless of the treatments they are randomized to.

The SAF-B will be used for all safety analyses related to Part B.

DocUUID : 3213b5be-8d63-4760-bea6-7e3ec175e71e

6.2.2 Full Analysis Set (FAS-B)

The FAS-B is defined as all randomized participants who are assigned treatment for Part B. Participants will be analyzed in according to the intention-to-treat principle, i.e., based on assigned treatment for Part B, irrespective of the treatment they actually received.
The FAS-B will be used for some efficacy analyses on endpoints related to the Part B.

6.3 Protocol Deviations/Violations and Exclusions from Analysis Sets

All protocol deviations will be identified prior to their associated database lock for any intentional or unintentional change or noncompliance with the approved protocol procedures or requirements as mentioned in protocol deviation criteria form (PDCF).

All protocol deviations will be classified as key or non-key and will be reviewed to determine their impact on the primary and key secondary endpoints during a data review meeting.

All exclusions from an analysis population will be documented prior to unblinding, exceptions to this will be protocol deviations based upon unblinded data (e.g PK analysis set) which will documented prior to release of the final outputs.

DocUUID : 3213b5be-8d63-4760-bea6-7e3ec175e71e

7 STATISTICAL CONSIDERATIONS AND ANALYSIS

7.1 Derived Variables

The below table provides the list of derived variables for demographic and baseline characteristics, various duration derivations, drug compliance, baseline derivations and other important derivations applicable for this study.

Variables	Formula
**Demographic and Baseline Characteristics**	
Body mass index (BMI) (kg/m^2)	weight (kg)/[height (m)^2]<br>Note: 1 lbs = 0.45359237 kg<br>1 in = 2.54 cm
Duration of PsA (Year)	[(Date of first study drug administration – Date of Initial diagnosis) + 1]/ 365.25<br>Details for imputing missing or partial start and/or stop dates of PsA are described in Section 7.3.2.
**Derivation of Duration**	
Study day at any visit	Date of interest – date of first dose of study drug. One day is added if this difference is ≥ 0.
Duration of Exposure (Days) for Part A	Date of Week 12 or EOT visit, whichever occurs first – Date of first randomized study medication intake at baseline + 1.
Duration of Exposure (Days) for Part B	Date of Week 24 or EOT visit, whichever occurs first – Date of first study medication intake for Part B + 1
Duration of Exposure (Days) for Part A+B	Date of Week 24 or EOT visit, whichever occurs first – Date of first sonelokimab intake + 1
**Drug Compliance**	
Compliance	Compliance (%) = 100 * (number of visits with treatment received/ number of visits with treatment planned) during the participant's participation in the study.<br>Compliance is calculated for Part A, Part B and Part A+B using the number of visits with treatment received during the respective Part and planned number in each Part also.
**Baseline Derivations**	
Baseline definition	Baseline will be defined as the last non-missing measurement prior to first dose of study medication in Part A For baseline characteristics table, if a subject is randomized and not treated then the latest record will be defined as the baseline.
Baseline definition for ADA	The last sample prior to the first dose of sonelokimab

DocUUID : 3213b5be-8d63-4760-bea6-7e3ec175e71e

Change from baseline	The value at each time point minus the value at baseline
**Other Derivations**	
Definition for the derived efficacy parameters are given in the respective efficacy analysis sections.	

7.2 Visit Windows

Visit windows will be used for all scheduled assessments. To allow for unscheduled and discontinuation assessments, the data will be summarized using the assessment (whether reported as a scheduled or unscheduled visit) closest to the nominal visit date, calculated from the first day of dosing. The visit result will be missing if no assessment was reported within the specified visit window around the scheduled date. If two assessments are equidistant from a scheduled visit, the earlier of the two will be used.

The visit windows will be calculated by bisecting the scheduled visit assessments. The upper limit of each window will be the mean of the two adjacent planned study days, rounded down to the nearest integer. The lower limit of each window will be the upper limit of the preceding window plus one, except for the first post-treatment visit which will start at 2.

Visit Windows for [redacted] PsAID-12, BASDAI, [redacted], PASI, LDI, Enthesitis evaluation (LEI+SPARCC enthesitis index)

Nominal Week	Nominal Study Day	Visit Window
0	1	All assessments prior to the start of study treatment
4	29	Day 2 – 43
8	57	Day 44 – 71
12	85	Day 72 – 99
16	113	Day 100 – 127
20	141	Day 128 - 155
24	169	Day 156+

Visit Windows for Safety Laboratory Testing (hematology and chemistry) and ESR

Nominal Week	Nominal Study Day	Visit Window
0	1	All assessments prior to the start of study treatment
4	29	Day 2 – 43
8	57	Day 44 – 71
12	85	Day 72 - 99
16	113	Day 100 - 127
20	141	Day 128 - 155

Visit Windows for Safety Laboratory Testing (hematology and chemistry) and ESR

Nominal Week	Nominal Study Day	Visit Window
24	169	Day 156 - 190
Safety Follow-up*	211	Day 191+

*Subjects who discontinued early, a safety follow-up period of 8 weeks after the last dose of study treatment

Visit Windows for Safety Laboratory Testing (Urinalysis)

Nominal Week	Nominal Study Day	Visit Window
0	1	All assessments prior to the start of study treatment
4	29	Day 2 – 57
12	85	Day 58 - 99
16	113	Day 100 - 141
24	169	Day 142 - 190
Safety Follow-up*	211	Day 191+

*Subjects who discontinued early, a safety follow-up period of 8 weeks after the last dose of study treatment

Visit Windows for PtGADA, PtAAP, HAQ-DI, hs-CRP, PhGADA, TJC68 and SJC66

Nominal Week	Nominal Study Day	Visit Window
0	1	All assessments prior to the start of study treatment
2	15	Day 2 – 22
4	29	Day 23 – 43
8	57	Day 44 – 71
12	85	Day 72 – 92
14	99	Day 93 - 106
16	113	Day 107 - 127
20	141	Day 128 - 155
24	169	Day 156+

DocUUID : 3213b5be-8d63-4760-bea6-7e3ec175e71e

Visit Windows for ADA and PK Sampling

Nominal Week	Nominal Study Day	Visit Window
0	1	All assessments prior to the start of study treatment
2	15	Day 2 – 22
4	29	Day 23 – 43
8	57	Day 44 – 71
12	85	Day 72 – 92
14	99	Day 93 - 106
16	113	Day 107 - 127
20	141	Day 128 - 155
24	169	Day 156 - 190
Safety Follow-up*	211	Day 191+

*Subjects who discontinued early, a safety follow-up period of 8 weeks after the last dose of study treatment

Visit Windows for SF-36v2, FACIT-Fatigue

Nominal Week	Nominal Study Day	Visit Window
0	1	All assessments prior to the start of study treatment
4	29	Day 2 – 57
12	85	Day 58 - 99
16	113	Day 100 - 127
20	141	Day 128+

Visit Windows for ECG

Nominal Week	Nominal Study Day	Visit Window
0	1	All assessments prior to the start of study treatment
12	85	Day 2 – 148
Safety Follow-up*	211	149+

*Subjects who discontinued early, a safety follow-up period of 8 weeks after the last dose of study treatment

Visit Windows for BSA and mNAPSI

Nominal Week	Nominal Study Day	Visit Window
0	1	All assessments prior to the start of study treatment
12	85	Day 2 – 127
24	169	128+

Visit Windows for Vital Signs

Nominal Week	Nominal Study Day	Visit Window
0	1	All assessments prior to the start of study treatment
2	15	Day 2 – 22
4	29	Day 23 – 36
6	43	Day 37 – 50
8	57	Day 51 – 64
10	71	Day 65 – 78
12	85	Day 79 – 92
14	99	Day 93 - 106
16	113	Day 107 - 120
18	127	Day 121 - 134
20	141	Day 135 - 148
22	155	Day 149 - 162
24	169	Day 163 - 190
Safety Follow-up*	211	Day 191+

*Subjects who discontinued early, a safety follow-up period of 8 weeks after the last dose of study treatment

7.3 Handling of Missing Data and Randomization Stratification Errors

7.3.1 Missing Data Analysis Methods

Missing data in this study may result from participants discontinuing from the study prematurely or missing intermediate visits or selected assessments while remaining on study. Every reasonable effort will be made to obtain the protocol-required data for all study assessments that are scheduled for all participants who have been enrolled.

Dichotomous Efficacy Endpoints

DocUUID : 3213b5be-8d63-4760-bea6-7e3ec175e71e

For all dichotomous endpoints, a non-responder imputation method will be used for the primary analysis. Participants will be considered non-responders if:

- They discontinue the study prior to the Week 12 visit for Part A or prior to the Week 24 visit for Part B and Part A+B (due to any reason). All future visits following the discontinuation will be imputed, or
- Have missing data for the endpoint of interest at baseline or at any intermediate time points. All visits with a missing assessment will be imputed, or
- Take prohibited protocol medication prior to the Week 12 visit for Part A or Week 24 visit for Part B and Part A+B. All future visits following the start date of the prohibited medication will be imputed.

In addition, two sensitivity approaches will be used for the dichotomous primary and key-secondary endpoints for analyses data:

1. The method for handling missing data will be dependent on the reason the data are missing. If a participant has discontinued due to lack of efficacy or an adverse event or starting prohibited medication the data will be imputed as a non-response. If the data are missing for any other reason the data will not be imputed. A multiple imputation for primary and key secondary analysis will be used including all available data up to Week 12 to enable the inclusion of participants with missing data at Week 12.
2. No data will be imputed and the data analysed by visit based on the observed data only.

Continuous Efficacy Endpoints

For continuous efficacy endpoints, missing observations will not be formally imputed. Mixed Model for Repeated Measures (MMRM) analysis will be used with all available data, including participants with partial data (i.e., with missing data) in order to arrive at an estimate of the mean treatment effect. MMRM is based on the assumption of missing at random (MAR), i.e., dropout would behave similarly to other participants in the same treatment group and possibly with similar covariate values, had they not dropped out.

Unless otherwise indicated, in other cases the observed data will be used and no imputation will be performed.

Scoring Derivations

Several instruments will be used in this study to measure some underlying construct. As such, missing data in this type of instruments can occur on the item scores. Missing data will be handled as described by the author of each instrument and detailed in the relevant endpoint section, or, if no information is stated by the author, no imputation will be done and such scores (and any resultant domain/total score) will remain missing.

7.3.2 Handling of Missing or Incomplete Dates

<u>Imputation rules for missing or partial AE start date</u> are defined below:

DocUUID : 3213b5be-8d63-4760-bea6-7e3ec175e71e

If only Day of AE start date is missing:

If the AE start year and month are the same as that for the first dose date, then:

- If the full (or partial) AE end date is NOT before the first dose date or AE end date is missing, then impute the AE start day as the day of first dose date; otherwise, impute the AE start day as 1.

If the AE start year and month are not same as that for the first dose date, then, impute the AE start day as 1.

Compare the imputed AE start date with treatment-emergent (TE) period to determine whether the AE is pre-treatment AE, treatment-emergent adverse event (TEAE) or post-treatment AE.

If Day and Month of AE start date are missing:

If AE start year = first dose year, then:

- If the full (or partial) AE end date is NOT before the first dose date or AE end date is missing, then impute the AE start Month and Day as the Month and Day of first dose date; otherwise, impute the AE start Month as January and the Day as 1.

If AE start year not same as first dose year, then, impute the AE start MONTH as January and the DAY as 1.

Compare the imputed AE start date with TE period to determine whether the AE is pre-treatment AE, TEAE or post-treatment AE.

If Year of AE start date is missing:

If the year of AE start date is missing or AE start date is completely missing, then query site with no imputation. Also compare the full (or partial) AE end date to the first dose date. If the AE end date is before the first dose date, then the AE should be considered as a pre-treatment AE. Otherwise, the AE will be considered as TEAE.

<u>Imputation rules for missing or partial medication start/stop dates</u> are defined below:

Missing or partial medication start date:

- If only DAY is missing, use the first day of the month.
- If DAY and Month are both missing, use the first day of the year.
- If DAY, Month and Year are all missing, use a date before the first dose date.

Missing or partial medication stop date:

- If only DAY is missing, use the last day of the month.
- If DAY and Month are both missing, use the last day of the year.
- If DAY, Month and year are all missing, then consider the medication is still ‘continuing’ and date will not be imputed.

<u>Imputation rules for missing or partial PsA start dates</u> are defined below:

Missing or partial PsA start date:

- If only DAY is missing, use the first day of the month.
- If DAY and Month are both missing, use the first day of the year.
- If DAY, Month and Year are all missing, use a date before the first dose date.

7.3.3 Randomization Stratification Errors

Randomization stratification errors can occur if the incorrect Sex or Prior Biologic Use is entered in the IRT system at the time of randomization. The FAS analyses will follow the ITT principle and analyse these participants according to the randomized stratum.

DocUUID : 3213b5be-8d63-4760-bea6-7e3ec175e71e

8 STATISTICAL METHODS

8.1 General Statistical Conventions

All statistical procedures will be completed using SAS version 9.4® or higher.

For continuous variables, descriptive statistics (number of observations, mean, standard deviation (SD), minimum (min), first quartile, median, third quartile and maximum (max) and 95% confidence intervals (CIs) as appropriate) will be presented. Summaries of hs-CRP and PK concentration will include the geometric mean (GM) and coefficient of variation (CV). The count of missing observations will be provided in all descriptive tables.

Categorical variables will be presented as counts and percentages with non-missing values in each category. The number of missing values will be presented as a separate category, if there is at least one missing value. Unless otherwise stated, all statistical testing will be two-sided and will be performed using a significance (alpha) level of 0.05. For all statistical analyses, p-values and 95% CIs will be provided. All sensitivity analyses will be regarded as exploratory and as such no formal adjustment for multiplicity will be made.

Number of decimal places will be presented as follow:

- continuous variables: for min and max, the decimals will be used as captured in the database; arithmetic mean, median and quartiles will be depicted with one more decimal than captured in the database; and SD will be depicted with two more decimals than captured in the database.
- categorical variables: percentages will be rounded to one decimal place and a percentage of 100% will be reported as 100%.
- the p-values will be rounded and displayed to 3 decimal places, values less than 0.001 will be displayed as 'p<0.001' and values higher than 0.999 will be displayed as 'p>0.999'.

This study has two parts Part A and Part B:

Part A Analysis: For Part A, summaries and analyses will be based on the data collected from the visits Week 0 to Week 12. Unless otherwise stated, the summary statistics and analyses will be presented for the following treatment groups:

- Sonelokimab 120 mg Q2W;
- Sonelokimab 60 mg Q2W;
- Sonelokimab 60 mg Q4W;
- Placebo;
- Adalimumab.
- Total sonelokimab (safety tables only)

Part B: For Part B no statistical testing will be performed. Summary statistics will be based on the data collected from the visits Week 12 to Week 24.

DocUUID : 3213b5be-8d63-4760-bea6-7e3ec175e71e

Unless otherwise stated, the summary statistics will be presented for the following treatment groups:

- Sonelokimab 120 mg Q2W→sonelokimab 120 mg Q4W;
- Sonelokimab 120 mg Q2W→adalimumab 40 mg Q2W;
- Sonelokimab 60 mg Q2W→sonelokimab 60 mg Q4W;
- Sonelokimab 60 mg Q2W→sonelokimab 120 mg Q4W;
- Sonelokimab 60 mg Q4W→sonelokimab 60 mg Q4W;
- Sonelokimab 60 mg Q4W→sonelokimab 120 mg Q4W;
- Placebo→sonelokimab 120 mg Q4W;
- Adalimumab 40 mg Q2W→adalimumab 40 mg Q2W;
- Adalimumab 40 mg Q2W→sonelokimab 120 mg Q2W.
- Total sonelokimab (safety tables only)

Part A+B: For Part A+B no statistical testing will be performed. Summary statistics will be based on the data collected from the visit Week 0 to Week 24. Part A+B tables will only be presented for adverse events.

Unless otherwise stated, the summary statistics will be presented for the following treatment groups:

- Placebo (Part A only)
- Total Sonelokimab 120mg
- Total Sonelokimab 60mg
- Total Adalimumab 40mg Q2W
- Total Sonelokimab

For the total groups they will have different subjects potentially for Part A and Part B and subjects will only have results shown for the Part when they took the relevant treatment.

All participant data, including those derived, will be presented in individual participant data listings. Unless otherwise stated, unscheduled visit results will be included in date/time chronological order, within participant listings only. All listings will be sorted by treatment group, investigational site, participant number, date/time, and visit. The treatment group as well as participant's sex, age and race will be stated on each listing. Unless otherwise stated, safety data listings will be based on SAF and efficacy data listings will be based on FAS.

8.2 Participant Disposition

The number and percentage of participants screened, randomized, randomized and treated, randomized and not treated and the number of participants in each of the analysis sets (including reasons for exclusion) separately for Part A and Part A+B (see section 7) will be summarized by treatment group on the enrolled set.

DocUUID : 3213b5be-8d63-4760-bea6-7e3ec175e71e

The number and percentage of participants will also be summarized for the following participant disposition categories:

- Completed Part A, defined as completing a visit in the Week 12 visit window or a later visit
- Completed study drug for Part A, defined as received the Week 10 dose or a dose at a later visit
- Discontinued study drug in Part A
- Received study drug in Part B, defined as received a dose at Week 12 or a later visit
- Completed study drug treatment for both part A and part B per-protocol;
- Discontinued study drug earlier during Part B (including the primary reason for discontinuation);
- Completed the study at week 24;
- Completed the study including post-treatment follow-up;
- Discontinued from study early (including the reason for discontinuation).

Given data from Part A and Part B will be combined together (including participants not entering Part B), all distinct treatment groups detailed in section 9.1 from Part A and B will be presented.

The time in days to study drug discontinuation due to any reason will be displayed with plot by initial randomized treatment for the FAS during Part A. The time to last visit will be defined as the discontinuation date minus the first dose date plus one.

The plot will be repeated for Part A+B to show the time to discontinuation of sonelokimab. This will only include subjects exposed to sonelokimab in either Part A or B. The time to discontinuation will be defined as the discontinuation date minus the first sonelokimab dose date plus one Subjects who complete the treatment at Week 24 will be censored at the completion day (Week 24).

A listing of participant disposition will be provided for enrolled set.

A listing of analysis set and reasons for exclusion from the analysis set will be provided for enrolled set.

8.3 Protocol Deviations

A summary will be provided for all key protocol deviations by deviation sub-category and treatment group based on the FAS will be presented for Part A and Part A + B separately.

In addition, all protocol deviations will be listed and protocol deviations occurring due to the COVID-19 pandemic will be listed separately for FAS.

8.4 Demographics and Baseline Characteristics

All demographic and baseline characteristics data will be listed and summarized for Part A by treatment group using the FAS. Inferential statistical analysis of demographic or baseline data will not be performed.

8.4.1 Demographics

Continuous variables such as age (years) at screening collected on the case report form (CRF), height (cm), weight (kg) and BMI (kg/m^2) will be summarized using descriptive statistics.

Categorical variables such as age categories (18-64, 65-84, >84), sex, ethnicity and race will be

summarized using counts and percentages.

Childbearing potential status, contraception methods used and reason for non-child-bearing potential status for female participants will be provided in the listing only.

8.4.2 Baseline and Disease Characteristics

The following variables will be summarized:

- Drug and alcohol history;
- Smoking history;
- PsA medical history;
    - Duration of PsA (Year)
    - Duration of PsA symptoms (Year)
    - Current active psoriasis (yes/no)
    - [redacted]
    - [redacted]
- Geographic region: North America, Western Europe and Eastern Europe;
- Number of prior biologic agents (0;1;2);
- Concomitant methotrexate use (yes/no);
- Concomitant non-biologic disease-modifying anti-rheumatic drug (DMARD) at baseline (yes/no);
- Concomitant corticosteroids at baseline (yes/no);
- Baseline BSA;
- Baseline BSA ≥ 3 (yes/no);
- Baseline TJC68 count;
- Baseline SJC66 count;
- Baseline PASI (in patients with BSA ≥ 3);
- Baseline PtAAP;
- [redacted]
- Baseline PtGADA;
- Baseline PhGADA;
- Baseline HAQ-DI;
- Baseline (PsAID)-12;
- Baseline BASDAI score;
- Baseline hs-CRP;
- Baseline hs-CRP ≥ 6 mg/L (yes/no);
- Baseline hs-CRP > 5 mg/L (yes/no);
- Baseline ESR;
- Baseline DAPSA;
- Baseline mNAPSI score (in patients with baseline mNAPSI ≥ 0);
- Presence of enthesitis at baseline (yes/no) [based on LEI];
- Baseline LEI score (in patients with baseline LEI ≥ 0);

DocUUID : 3213b5be-8d63-4760-bea6-7e3ec175e71e

- Presence of enthesitis at baseline (yes/no) [based on SPARCC];
- Baseline SPARCC (in patients with baseline SPARCC ≥ 0);
- Presence of dactylitis at baseline (yes/no);
- Baseline LDI (in patients with dactylitis);
- SF-36 PCS score
- Evidence of erosions on hands or feet in patients with a prior x-ray (yes/no);
- Evidence of juxta-articular bone formation on hands or feet in patients with a prior x-ray (yes/no)
- Baseline FACIT-fatigue

Continuous variables will be summarized using descriptive statistics and categorical variables will be summarized using counts and percentages. Percentage calculations will be based on the number of participants in FAS including participants with missing value in each treatment group.

8.4.3 Medical History

Medical history will be summarized by system organ class (SOC) and preferred term (PT) coded using Medical Dictionary for Regulatory Affairs® (MedDRA) Version 25.0 or higher. Counts and percentages will be presented by treatment group using the SAF.

8.4.4 Psoriatic Arthritis Previous Therapies

PsA previous therapies will be summarised by treatment group using the SAF using the following group terms with the individual drug names summarised within the group terms:

- anti-TNF biologics,
- anti-IL-17 biologicals,
- anti-IL23 biologicals
- other biologics coded by World Health Organization Drug Dictionary Enhanced (WHODDE)
- NSAIDs/COX-2 inhibitors
- Other NSAIDs/COX-2 inhibitors coded by WHODDE
- Non-biologic DMARDs
- Other non-biologic DMARDs coded by WHODDE
- Glucocorticoids
- Other glucocorticoids coded by WHODDE
- JAK inhibitors
- High potency opiods
- Systemic retinoids
- Phototherapy
- Inhaled marijuana
- Other treated coded by WHODDE

8.4.5 Inquiries for extra-musculoskeletal PsA manifestation

Inquiries for extra-musculoskeletal PsA manifestation will be listed by using the SAF.

DocUUID : 3213b5be-8d63-4760-bea6-7e3ec175e71e

8.4.6 Previous X-ray images of hands and feet

Previous X-ray images of hands and feet will be listed by using the SAF.

8.4.7 Prior and Concomitant Medications

Prior and concomitant medications will be summarized separately by WHODDE ATC level 2 and preferred name. Counts and percentages will be presented by treatment group using the SAF for Part A and Part A+B and using SAF-B for Part B. Prior medications will be summarized for Part A and concomitant medication will be summarized separately for Part A, Part B and Part A+B.

Prior medications: are defined as those medications with a start date and stop date prior to the first dose of study drug.

Concomitant medications:

- for part A, are defined as those medications with a start date on or after the first dose of study drug of part A but not after the first dose of Part B. A medication which started prior to first dose of part A and continued after first dose will also be considered as concomitant medications for part A.
- for part B, are defined as those medications with a start date on or after the first dose of study drug of part B. A medication which started prior to first dose of part B and continued after first dose of part B will also be considered as concomitant medications for part B.
- for part A+B, are defined as those medications with a start date on or after the first dose of study drug of part A. A medication which started prior to first dose of part A and continued after first dose will also be considered as concomitant medications for part A+B.

Details for imputing missing or partial start and/or stop dates of medication are described in Section 7.3.2.

8.5 Extent of Exposure

Extent of exposure will be listed and summarized using the SAF separately for Part A and Part A+B and SAF-B for Part B.

For Part A+B, only two groups will be presented:

- Sonelokimab 120 mg,
- Sonelokimab 60 mg.

8.5.1 Treatment Duration

The duration (days) (Section 7.1 for derivations) of exposure to study drug will be summarized using descriptive statistics by treatment group. In addition, the number of injections received will be summarized with count and percentages.

8.5.2 Treatment Compliance

Treatment compliance (See Section 7.1 for derivations) will be summarized by treatment group using descriptive statistics. Counts and percentages will be provided using the following categories: <80%; 80% ≤ x < 100%; 100%; 100% < x ≤ 120% and > 120%.

DocUUID : 3213b5be-8d63-4760-bea6-7e3ec175e71e

8.6 Efficacy Analyses

This section addresses the analyses to be conducted on the primary, secondary and exploratory efficacy variables.

8.6.1 Analysis Methods

8.6.1.1 Logistic Regression Model

For the primary analysis, the hypotheses to be tested are that the proportion of participants who achieve ACR50 at Week 12 compared with baseline in the sonelokimab 120 mg Q2W, sonelokimab 60 mg Q2W or sonelokimab 60 mg Q4W arms are different from that of the placebo arm.

The logistic regression will be used to test the pairwise comparisons (placebo and either sonelokimab 120 mg Q2W, sonelokimab 60 mg Q2W, sonelokimab 60 mg Q4W or adalimumab) including fixed effects for treatment and the stratification factors (sex and exposure to biologic agents prior to the screening visit). Baseline score will also be included as a covariate where there is a single score for the endpoint, for example PASI for the analysis of PASI90.

Results will be presented in terms of number and percentage of responders, adjusted response rate, odds ratios (active/placebo), as well as risk difference (active-placebo), 95% CI, and estimated p-value.

The OR is a ratio of two odds, the odds of the event of interest in individuals exposed to active treatment and the odds of the event of interest in individuals exposed to placebo. The OR can be interpreted as:

- An OR=1 indicates that the odds of having the event of interest (e.g. ACR50 responder) is the same in the active and placebo groups,
- An OR>1 indicates that the odds of having the event of interest (e.g. ACR50 responder) is greater in active group than in the placebo group,
- An OR<1 indicates that the odds of having the event of interest (e.g. ACR50 responder) is lower in active group than in the placebo group,

Using the procedure, the null hypothesis to be tested is that the OR is equal to one versus the OR is not equal to one.

In the case of having 0% or 100% response rate in one group, the estimated p-value (from a Fisher's Exact Test) will be presented, but the odds ratio and 95% CI will not be displayed.

The risk difference estimator will also be displayed and is derived from a logistic regression model. The adjusted response rates will be obtained from back transforming the estimates of the odds for each arm from the logistic regression model. The risk difference will be calculated as the difference (active-placebo) of the adjusted response rates. The delta method will be used to estimate the standard error of the risk difference from the logistic regression model (Ge et al. 2011). Appendix M has pseudo code used to create the logistic regression estimator approach.

DocUUID : 3213b5be-8d63-4760-bea6-7e3ec175e71e

8.6.1.2 Mixed Model Repeated Measures

Continuous efficacy endpoints assessed over time will be analyzed using an MMRM model including treatment arm, baseline, visit, sex, prior exposure to biologic agents and treatment by visit interaction as fixed effects and participant as random effect. An unstructured covariance matrix will be used for the MMRM analysis, unless the model does not converge, in which case the covariance matrix (e.g. variance components, autoregressive, compound symmetry, and Toeplitz) will be decided upon model convergence status and the Akaike information criterion.

The MMRM will be used to test for differences in the mean of the endpoint of interest (e.g. change from baseline in TJC68) between groups of interest (e.g. each active treatment group versus placebo).

From the model, the adjusted least squares (LS) means in each group and the difference in LS means for the group comparisons will be estimated together with 95% CIs for the differences and p-values for the pairwise comparisons. Treatment effect estimates will be also extracted by visit.

8.6.1.3 Multiple Imputation

As a sensitivity analysis of the primary and key secondary dichotomous efficacy endpoints (e.g. ACR50 responder), multiple imputation of missing data will be performed and analyzed using logistic regression. Multiple imputation uses a simulation based approach, replacing missing values from multiple draws from conditional distributions given the observed data and covariates. Assuming an arbitrary missing data pattern the missing values for each component will be imputed separately based on a fully conditional specification (FCS) approach, this method uses all variables in the imputation model for any other variable.

The imputations will be done using the corresponding baseline value, sex and exposure to biologic agents at baseline as covariates. If there are any missing values in those variables they will also be imputed, in the model a single component at a specific visit, will consider all previous visits as covariates. The full conditional specification regression model will be used for continuous variables and the number of imputations will be 100 with 50 burn in imputations and the seed for all imputations will be 4278491. For all visit results a minimum value of 0 will be added to the imputation. If the imputation does not input values within the specified range then the number of maximum iterations by step will be increased accordingly.

For each completed imputed dataset, the necessary derived variables will be computed using logistic regression as described in section 8.6.1.1 and the results obtained will be combined into one multiple imputation inference using proc MIANALYZE.

The SAS pseudo code for this imputation process is listed below:

[redacted]

[redacted]

8.6.1.4 Multiplicity

The following multiplicity adjustments will be used to control the overall type I error rate at 0.05 for the primary endpoint and key secondary endpoints. All sensitivity analyses will be regarded as exploratory and no formal adjustment for multiplicity will be made.

Primary Endpoints

DocUUID : 3213b5be-8d63-4760-bea6-7e3ec175e71e

To adjust for multiple testing and to control the family-wise error rate, the Bonferroni-Holm method will be applied first to sonelokimab 120 mg Q2W (Arm 1) and sonelokimab 60 mg Q2W(Arm 2), followed by a step-down procedure to test for sonelokimab 60 mg Q4W (Arm 3).

The Bonferroni-Holm procedure will work as follows:

1) Let $p_{SLK120mg,Q2W}$ and $p_{SLK60mg,Q2W}$ be the p-value resulting from the comparison between sonelokimab 120 mg,Q2W vs placebo and sonelokimab 60 mg,Q2W vs placebo groups respectively.
2) If the smallest p-value obtained in step 1 is greater than or equal to alpha/2 = 0.025, the procedure is stopped and no p-values are significant.
3) If the smallest p-value is declared significant (i.e p-value less than alpha/2 = 0.025), then compare the largest p-value with alpha = 0.05. If the p-value is less than 0.05, then declared as significant. Otherwise not significant.
4) If both $p_{SLK120mgQ2W}$ and $p_{SLK60mgQ2W}$ are significant then the testing procedure will continue with the key secondary endpoints. The Bonferroni-Holm procedure used for the primary endpoint will be applied to each key secondary endpoint sequentially.
5) If the procedure passes all the key secondary endpoints for both doses (Arm 1 and Arm 2), then sonelokimab 60 mg Q4W (Arm 3) will be compared to alpha = 0.05 first for the primary endpoint and then sequentially through the key secondary endpoints.

8.6.1.5 Treatment-by-Center Interaction Analysis (Multi-Center Study)

Unless otherwise specified, data from all investigational centers/sites will be pooled for analyses.

8.6.2 Analysis of Primary Efficacy Endpoint

The primary analysis will be performed using the FAS.

All sensitivity analyses will be performed using FAS population. Summaries for Part B will be performed using the FAS-B population.

8.6.2.1 American College of Rheumatology 50 Clinical Response

The ACR50 will be assessed at Week 2, Week 4, Week 8, Week 12, Week 14, Week 16, Week 20 and Week 24 however the primary endpoint is the proportion of ACR50 responders at Week 12.

ACR50 response criteria is defined as:

- ACR50 Responder: *participant who had improvement in the ACR response criteria ≥ 50%;*
- ACR50 Non-responder: *Otherwise.*

ACR50 response is:

• At least a 50% improvement in TJC68;

• At least a 50% improvement in SJC66;

• At least a 50% improvement in at least 3 of the following 5 variables:

- PtAAP;
- PtGADA;
- PhGADA;
- HAQ-DI;
- hs-CRP.

A Non-responder imputation method will be used for all missing data for the primary estimand as detailed in section 7.3.2.

The null hypotheses to be tested are that the proportion of ACR50 responders at Week 12 are no different for the following comparisons:

- sonelokimab 120 mg Q2W versus placebo
- sonelokimab 60 mg Q2W versus placebo
- sonelokimab 60 mg Q4W versus placebo

[redacted]

To test these hypotheses, a pairwise stratified logistic regression will be used as detailed in Section 8.6.1.1. This analysis will also be conducted for Weeks 2, 4 and 8.

The data for Part B will be presented by FAS-Part B and will be split by Part A and Part B treatment.. Non-responder imputation and As-Observed summaries will be presented. No statistical analysis will be presented. These results will also be presented in a line plot.

The following sensitivity analysis of the Part A data will be presented:

- the first sensitivity estimand will be analysed using logistic regression, with the same covariates as for the primary analysis. Missing data will be imputed as described in section 5.2 with multiple imputation applied as described in 8.6.1.3such that subjects who had prohibited medication or discontinued due to efficacy or adverse events are set as non-responders, all other missing data is imputed with multiple imputation methods.
- the second sensitivity estimand will be analysed using logistic regression, with the same covariates as for the primary analysis with multiple imputation applied as described in 8.6.1.3
- An observed analysis using logistic regression model but with no imputation of missing data.

Line plots for ACR50 response rate by week will also be provided, with week on the x axis and ACR50 response rate on the y axis. Graphs for Part A and Part B (FAS) will be presented.

The responders by visit and treatment group will be summarized descriptively for Part A and Part B

The change from baseline for each component of the ACR endpoint will be analyzed using the method described in 8.6.1.2, Descriptive summaries by treatment group and visit will also be displayed for Part A and Part B.

For Part A and B there will also be a NRI/LOCF analysis. Subjects who completed the study at Week 12 due to country-specific protocol requirements (US participants) or subjects who switched arm at Week 12 will have their Part B scheduled visits be imputed with LOCF from their Week 12 visits. All

DocUUID : 3213b5be-8d63-4760-bea6-7e3ec175e71e

other missing data is imputed as a non-response. A summary of the number of responders by Part A treatment arm for all scheduled visits will be displayed.

8.6.2.2 TJC68

The total number of tender joints from the 2 temporomandibular, 2 sternoclavicular, 2 acromioclavicular joints, 2 shoulders, 2 elbows, 2 wrists, 10 metacarpophalangeal, 10 proximal interphalangeal, 8 distal interphalangeal joints of the hands, the 2 hips, 2 knees, 2 ankles, 2 mid-tarsal, 10 metatarsophalangeal, and 10 proximal interphalangeal joints of the feet.

8.6.2.3 SJC66:

The total number of swollen joints from the same joints as assessed for tenderness, excluding the hip joints.

8.6.2.4 Health Assessment Questionnaire (HAQ-DI)

The health assessment questionnaire (HAQ-DI) is a twenty-item PRO instrument which assesses current physical function/disability. It covers 8 categories (dressing and grooming, hygiene, arising, reach, eating, grip, walking and common daily activities). There are 4 response options ranging from 0 (no difficulty) to 3 (unable to do) (See Appendix A).

To calculate the HAQ-DI, the highest sub-category score determines the value for each category, unless aids or devices are used. There must be at least 6 out of 8 categories answered or else the score cannot be calculated. Each category scored is averaged into an overall HAQ-DI score ranging from 0 to 3.

The aids used for adjustment by category are: dressing – devices used for dressing; rising – built up or special chars; eating – built up or special utensils; walking – canes, walkers or crutches; hygiene – raised toilet seats, bathtub seats, bathtub bars, long handles appliances in bathroom; reach – long-handled appliances for reaching; and grip – jar openers.

If an aid is required for any category, or another person is required to help for any category then that category score increases a score of 0 or 1 to a 2. Scores already at 2 and 3 are not modified. Aids and devices considered recorded in the 'other' category will not be considered in the analysis.

Scores of 0 to 1 represent mild to moderate disability, 1 to 2 represent moderate to severe disability and 2 to 3 indicate severe to very severe disability.

8.6.2.5 Patients Assessment of Arthritis pain

The patients' assessment of arthritis pain (PtAAP) is a self-administered pain visual analogue score (VAS). It is performed using a horizontal 100mm VAS, ranging from 0 (no pain) to 100 (severe pain) after the question: "Please indicate with a vertical mark (|) through the horizontal line the most pain you had from your psoriatic arthritis today. (See Appendix B)

8.6.2.6 Patients Global Assessment of Disease Activity

The patients global assessment of disease activity (PtGADA) is self-administered and performed using a horizontal 100mm VAS ranging from 0 (very well) to 100 (very poor) after the question "Considering

DocUUID : 3213b5be-8d63-4760-bea6-7e3ec175e71e

all the ways PsA affects you, please indicate with a vertical mark (|) through the horizontal line how well you are doing today.” (See Appendix C)

8.6.2.7 Physician’s Global Assessment of Disease Activity

The physician’s global assessment of disease activity (PhGADA) is assessed by the investigator or a qualified designee on a VAS ranging from 0 (very well) to 100 (very poor). (See Appendix D)

8.6.2.8 Hs-CRP

Hs-CRP values are assessed at Baseline, Week 2, Week 4, Week 8, Week 12, Week 14, Week 16, Week 20 and Week 24.

Hs-CRP values which are recorded as <LLQ (i.e <0.10 mg/L) will be set to be half the value of the LLQ for these values (i.e 0.05 mg/L) for analysis purpose.

To test hs-CRP, the data will be log transformed prior to using a MMRM including as detailed in section 8.6.1.2. The results will be back-transformed to be presented in terms of adjusted least-squares (LS) geometric means, and ratios in LS geometric means together with 95% CIs and p-values.

If the data suggest that either endpoint is normally distribution rather than log-normally distributed then the analysis will be performed on the change from baseline without log-transformation.

The CRP assessments as well as the corresponding ratio to baseline will also be summarized descriptively by treatment group and visit for Part A and Part B.

The hs-CRP analysis will also be repeated, only including subjects with a baseline hs-CRP >ULN (5mg/L).

8.6.2.9 ACR Components

For all the continuous components which make up the ACR as well as MMRM analysis, they will summarized by visit, treatment arm and Part.

For Part A and B there will also be a Last Observation Carried Forward (LOCF) summary of the ACR components to present the absolute, change from baseline and percent change from baseline by the randomized treatment arm. All missing data will be imputed using LOCF and subjects who completed the study at Week 12 due to country-specific protocol requirements (US participants) or subjects who switched arm at Week 12 will have their Part B scheduled visits be imputed with LOCF from their Week 12 visits.

8.6.3 Analysis of Key Secondary Efficacy Endpoints

For the key secondary endpoints, summaries and analyses will be performed using the FAS.

In addition, for the key secondary dichotomous endpoints, a sensitivity analysis will be performed using the FAS. Summaries of part B will be performed using the FAS-B population.

8.6.3.1 American College of Rheumatology 20 Clinical Response

A key secondary endpoint is the proportion of ACR20 responders at Week 12 compared with baseline.

ACR20 response criteria is defined as:

- ACR20 Responder: *participant who had improvement in the ACR response criteria ≥ 20%;*
- ACR20 Non-responder: *Otherwise.*

ACR20 response is:

- • At least a 20% improvement in TJC68;
- • At least a 20% improvement in SJC66;

At least a 20% improvement in at least 3 of the following 5 variables:

- PtAAP;
- PtGADA;
- PhGADA;
- HAQ-DI;

− hs-CRP.

ACR20 will be analysed using the same methods as used for ACR50.

8.6.3.2 Psoriasis Area and Severity Index 90

A key secondary endpoint is change from baseline in PASI90 at Week 12 in the subgroup of participants with psoriasis [PsO] involving at least 3% body surface area [BSA] at baseline. This is expected to be between 50% and 67% of the FAS.

The PASI is a validated tool to dynamically assess PsA severity.

The PASI score is an established measure of clinical efficacy for PsO medications, which provides a numeric scoring for the participant's overall PsO disease state, ranging from 0 to 72. It is a linear combination of percent of surface area of skin that is affected and the severity of erythema, infiltration and desquamation over 4 body regions (Head, Trunk, Upper extremities, Lower extremities).

These correspond to 10%, 30%, 20% and 40% of the total body area respectively.

The area of psoriatic involvement of these 4 areas (Ah, At, Au, and Al) is given a numerical value:

• 0 = No involvement;

• 1 = <10%;

• 2 = 10% to <30%;

• 3 = 30% to <50%;

• 4 = 50% to <70%;

• 5 = 70% to <90%;

• 6 = 90% to 100% involvement.

The signs of severity, erythema (E), infiltration (I), and desquamation (D) of lesions are assessed using a numeric scale 0 to 4 where 0 is a complete lack of cutaneous involvement and 4 is the

DocUUID : 3213b5be-8d63-4760-bea6-7e3ec175e71e

severest possible involvement; scores are made independently for each of the areas, h, t, u and l and represent a composite score for each area.

An illustration of judging erythema follows:

• 0 = No erythema;

• 1 = Slight erythema;

• 2 = Moderate erythema;

• 3 = Striking erythema;

• 4 = Exceptionally striking erythema.

The PASI score is calculated according to the following formula:

PASI = 0.1(Eh+Ih+Dh)Ah + 0.3(Et+It+Dt)At + 0.2(Eu+Iu+Du)Au + 0.4(El+Il+Dl)Al

PASI90 is defined as an improvement of 90% of more in the PASI from baseline score.

The PASI90 will be assessed at Baseline, Week 4, Week 8, Week 12, Week 16, Week 20 and Week 24, but the key secondary endpoint is the assessment at Week 12.

The null hypotheses to be tested are that the proportion of PASI90 responders at Week 12 are not different for the following comparisons:

- sonelokimab 120 mg Q2W versus placebo
- sonelokimab 60 mg Q2W versus placebo
- sonelokimab 60 mg Q4W versus placebo

[redacted]

To test these hypothesis, a logistic regression will be used as detailed in section 8.6.1.1. This analysis will also be conducted for Week 2, 4 and 8.

The same sensitivity as for the primary endpoint will be presented and the same graphs and descriptive statistics will also be displayed for Part A and Part B.

8.6.4 Analysis of Other Secondary Efficacy Endpoints

For the other secondary endpoints, no confirmatory statistical testing will be performed, but statistical analyses may be used for exploratory proposes.

FAS will be used for all other secondary endpoints analysis. Summaries of part B will be performed using the FAS-B population.

8.6.4.1 American College of Rheumatology Clinical Response (ACR20/50)

Other secondary endpoints are:

- Proportions of ACR20 responders over time.
- Proportions of ACR50 responders over time.

ACR20 and ACR50 response criteria are defined in section 8.6.3.1 and in section 8.6.2.1 respectively.

DocUUID : 3213b5be-8d63-4760-bea6-7e3ec175e71e

A Non-responder imputation method will be used for all missing data as detailed in section 7.3.1

The ACR20/50 will be assessed at Week 2, Week 4, Week 8, Week 12, Week 14, Week 16, Week 20 and Week 24. Only Part A will be included in logistic regression analysis.

The exploratory null hypothesis to be tested are that the proportion of ACR20/50 responders at each time point in Part A are not different for the comparisons of interest for Part A. For exploratory purposes, the comparisons of interest will be:

- sonelokimab 120 mg Q2W versus placebo
- sonelokimab 60 mg Q2W versus placebo
- sonelokimab 60 mg Q4W versus placebo

[redacted]

ACR20/50 responders will be also summarized for all weeks for Part A, Part B and will be displayed by treatment group and visit as described in section 8.1.

8.6.4.2 American College of Rheumatology 70 Clinical Response (ACR70)

Other secondary endpoint include the proportion of ACR70 responders over time.

ACR70 response criteria is defined as:

- ACR70 Responder: *participant who had improvement in the ACR response criteria ≥ 70%;*
- ACR70 Non-responder: *Otherwise.*

ACR70 response is:

• At least a 70% improvement in TJC68;

• At least a 70% improvement in SJC66;

• At least a 70% improvement in at least 3 of the following 5 variables:

  - PtAAP;
  - PtGADA;
  - PhGADA;
  - HAQ-DI;
  - hs-CRP.

The exploratory null hypotheses to be tested are that the proportion of ACR70 responders at each time point are not different for the comparisons of interest for Part A, a logistic regression will be performed as described in section 8.6.1.1. For exploratory purposes the comparison of interest will be:

- sonelokimab 120 mg Q2W versus placebo
- sonelokimab 60 mg Q2W versus placebo
- sonelokimab 60 mg Q4W versus placebo

[redacted]

ACR70 responders will also be summarized descriptively by treatment group and visit for Part A and Part B.

8.6.4.3 Psoriasis Area and Severity Index (PASI75/90/100)

Other secondary endpoints include the proportion of PASI75/90/100 responders over time.

PASI is derived as detailed in section 8.6.3.2. PASI75/90/100 are defined as an improvement in PASI score of 75%, 90% and 100% respectively.

The exploratory null hypotheses to be tested are that the proportion of PASI75/90/100 responders at each time point are not different for the comparisons of interest for Part A, a logistic regression will be performed as described in section 8.6.1.1. For exploratory purposes the comparison of interest will be:

- sonelokimab 120 mg Q2W versus placebo
- sonelokimab 60 mg Q2W versus placebo
- sonelokimab 60 mg Q4W versus placebo

[redacted]

PASI75/90/100 responders will also be summarized descriptively by treatment group and visit for Part A and Part B.

8.6.4.4 Minimal Disease Activity

Other secondary endpoints include the proportion of patients with minimal disease activity at Week 12.

Criteria for assessing minimal disease activity, listed below, covering all of the domains of the disease have been developed to determine whether the participant has reached minimal disease activity based on key outcome measures in PsA. Minimal disease activity is defined as meeting 5 of the 7 following criteria:

1. TJC68 ≤1;
2. SJC66 ≤1;
3. PASI ≤1 or PsO affecting ≤1% of BSA;
4. PtAAP ≤15 on a 0 to 100 VAS;
5. PtGADA ≤20 on a 0 to 100 VAS;
6. HAQ-DI ≤0.5.
7. Leeds Enthesitis Index (LEI) ≤1

The null hypotheses to be tested are that the proportion of responders at Week 12 are no different for the following comparisons:

- sonelokimab 120 mg Q2W versus placebo
- sonelokimab 60 mg Q2W versus placebo
- sonelokimab 60 mg Q4W versus placebo

[redacted]

To test the hypotheses, a logistic regression will be used as detailed in section 8.6.1.1. Descriptive summaries by treatment group and visit will also be displayed for Part A and Part B. An LOCF/NRI analysis by visit similar to in section 8.6.2.1 will also be displayed for all scheduled visits in Part A and B.

DocUUID : 3213b5be-8d63-4760-bea6-7e3ec175e71e

[redacted]

8.6.4.6 Leeds Enthesitis Index (LEI) and Spondyloarthritis Research Consortium of Canada Enthesitis Index (SPARCC)

The LEI is a validated enthesitis index which uses 6 sites for evaluation of enthesitis (lateral epicondyle humerus [R/L], Achilles tendon insertion [R/L] and medial condyle femur [R/L] giving an overall score range of 0 to 6 (see Appendix E).

The proportion of subjects with enthesitis resolution, defined as LEI = 0 will be tested using pairwise stratified logistic regression as detailed in section 8.6.1.1 in the subgroup of subjects with LEI>0 at baseline. .

The change from baseline in LEI will be analyzed using the method described in 8.6.1.2, including only subjects with LEI > 0 at baseline. Descriptive summaries by treatment group and visit will also be displayed for Part A and Part B.

The SPARCC Index uses 18 sites, greater trochanter (R/L), quadriceps tendon insertion into the patella (R/L), patellar ligament insertion into the patella or tibial tuberosity (R/L), achilles tendon insertion (R/L), plantar fascia insertion (R/L), medial epicondyle (R/L) and supraspinatus insertion into greater tuberosity of humerus (R/L). The maximum score is 16.

DocUUID : 3213b5be-8d63-4760-bea6-7e3ec175e71e

The change from baseline in SPARCC will be analyzed using the method described in 8.6.1.2, including subjects with SPARCC > 0 at baseline. Descriptive summaries by treatment group and visit will also be displayed for Part A and Part B. For Part A and B there will also be an LOCF summary of the LEI and SPARCC to present the absolute, change from baseline and percent change from baseline by the randomized treatment arm.

8.6.4.7 Bath Ankylosing Spondylitis Disease Activity Index (BASDAI)

The BASDAI will be assessed at Week 4, Week 8, Week 12, Week 16, Week 20 and Week 24 however the secondary endpoint is the proportion of BASDAI responders at Week 12.

BASDAI response criteria is defined as:

- BASDAI Responder: *participant who had improvement in the BASDAI response criteria ≥ 50%;*
- BSDAI Non-responder: *Otherwise.*

The BASDAI is a validated composite index consisting of 6 participant numerical rating scales (NRS) to measure severity of the following:

• Fatigue;

• Axial involvement;

• Peripheral articular involvement;

• Localized tenderness/enthesopathy;

• Severity of morning stiffness;

• Time of morning stiffness.

Each NRS has a range of 0 to 10 where 0 represents “none” and 10 represents “very severe.” For

Question 6, 0 represents no stiffness and 10 represents 2 or more hours of stiffness.

The composite BASDAI score is calculated as the average of questions 5 and 6 (Severity and time of morning stiffness), then as the mean of first 4 questions and the average of questions 5 and 6.

$$BASDAI = \frac{Q1 + Q2 + Q3 + Q4 + \frac{(Q5 + Q6)}{2}}{5}$$

A response rate of BASDAI is calculated as subjects with at least a 50% improvement in BASDAI.

A Non-responder imputation method will be used for all missing data as detailed in Section 8.3.1.

The null hypotheses to be tested are that the proportion of BASDAI50 responders at Week 12 are no different for the following comparisons:

- sonelokimab 120 mg Q2W versus placebo

- sonelokimab 60 mg Q2W versus placebo
- sonelokimab 60 mg Q4W versus placebo

[redacted]

To test these hypotheses, a pairwise stratified logistic regression will be used as detailed in Section 8.6.1.1. Descriptive summaries by treatment group and visit will also be displayed for Part A and Part B

The change from baseline in BASDAI will be analyzed using the method described in 8.6.1.2. Descriptive summaries by treatment group and visit will also be displayed for Part A and Part B.

8.6.4.8 Psoriatic Assessment Impact of Disease-12

The psoriatic assessment impact of disease-12 (PsAID-12) is self-administered by the participant and is a questionnaire including 12 health domains related to PsA. Each domain is evaluated on a score of 0 (None) to 10 (Extreme). The 12 domains are: pain, fatigue, skin problems, work and/or leisure activities, functional capacity, discomfort, sleep disturbance, coping, anxiety, embarrassment and/or shame, social participation, and depression (See Appendix F).

The PsAID-12 final value is calculated by summing each weighted domain which is calculated by multiplying the score (range of 0 to 10) by the weighted value as given in the table below and then dividing the total by 20:

Domain NRS Value	Multiplication
Pain	3
Fatigue	2
Skin	2
Work and/or leisure activities	2
Function	2
Discomfort	2
Sleep	2
Coping	1
Anxiety	1
Embarrassment	1
Social Life	1
Depression	1

A PsAID-12 ≤ 3 responder is a subject who has a final value of 3 of below.

A Non-responder imputation method will be used for all missing data as detailed in Section 8.3.1.

The exploratory null hypotheses to be tested are that the proportion of PsAID-12 ≤ 3 at Weeks 4, 8 and 12 are no different for the following comparisons:

DocUUID : 3213b5be-8d63-4760-bea6-7e3ec175e71e

- sonelokimab 120 mg Q2W versus placebo
- sonelokimab 60 mg Q2W versus placebo
- sonelokimab 60 mg Q4W versus placebo

[redacted]

To test these hypotheses, a pairwise stratified logistic regression will be used as detailed in Section 8.6.1.1. Descriptive summaries by treatment group and visit will also be displayed for Part A, Part B and Part A+B.

The change from baseline in PsAID-12 will be analyzed using the method described in 8.6.1.2. Descriptive summaries by treatment group and visit will also be displayed for Part A and Part B. For Part A and B there will also be an LOCF summary of the PsAID-12 to present the absolute, change from baseline and percent change from baseline by the randomized treatment arm.

8.6.4.9 Leeds Dactylitis Index

The Leeds Dactylitic index (LDI) is assessed by an individual suitably qualified and experienced in the administration of the instrument. It measures the ratio of the circumference of the affected digit to the circumference of the digit on the opposite hand or foot, using a minimum difference of 10% to define a dactylitic digit. The ratio of circumference is multiplied by a tenderness score, using a modification of LDA which is a binary score (1 for tender, 0 for non-tender) (See Appendix G). If both sides are considered involved, or the circumference of the contralateral digit cannot be obtained, the number will be compared to data provided in the standard reference table for hands and feet.

The dactylitis count is the number of fingers and toes with dactylitis, ranging from 0 to 20.

The final total LDI score is calculated as:

$$[\{(A/B) - 1\} \times 100] \times C$$

where

A = Total circumference involved digit

B = Contralateral Digit

C = Tenderness Score

Descriptive summaries by treatment group and visit will also be displayed for Part A and Part B, including subjects with presence of dactylitis at baseline (LDI >0). In the final total LDI score calculation, only digits with >=10% difference from contralateral score will be included.

[redacted]

[redacted]

8.6.4.11 Modified Nail Psoriasis Severity Index

The nNAPSI is a tool to assess nail involvement in participants with PsA with an index score ranging to 0 to 130 for all fingernails.

Three features or groups of features (pitting, onycholysis and oil-drop dyschromia and crumbling) or each fingernail will be graded on a score of 0 to 3.

The number of pits for each finger nail is scores as 0 = 0, 1-10 = 1, 11- 49 = 2 and >50 = 3.

The score percent of nail with crumbling present it scored as no crumbling = 0, 1-25% = 1, 26 – 50% = 2 and >50% = 3.

The score percent of nail with onycholysis and/or oil drop dyschromia is: none = 0, 10% = 1, 11-30% = 2, >30% = 3.

Four features (leukonychia, splinter hemorrhages, hyperkeratosis, and red spots in the lunula) will be graded as either present (1) or absent (0) in each fingernail.

The total score is calculated by summing the score for each feature and each fingernail.

DocUUID : 3213b5be-8d63-4760-bea6-7e3ec175e71e

The change from baseline in mNAPSI will be analyzed using the method described in 8.6.1.2 for subjects with nail psoriasis at baseline (mNAPSI > 0). Descriptive summaries by treatment group and visit will also be displayed for Part A and Part B. For Part A and B there will also be an LOCF summary of the mNAPSI to present the absolute, change from baseline and percent change from baseline by the randomized treatment arm.

8.6.4.12 Functional Assessment of Chronic Illness Therapy-Fatigue (FACIT-Fatigue)

Other secondary endpoints include:

- Change from baseline in Functional Assessment of Chronic Illness Therapy-Fatigue (FACIT-F) score

FACIT-Fatigue is a 13-item self-assessment of fatigue and the effect on daily activities and function over the previous 7 days (See Appendix H). The 13 items use a 5-point Likert-type scale (0 = "Not at all", 1 = "A little", 2 = "Somewhat", 3 = "Quite a bit", 4 = "Very much"). The FACIT-Fatigue assessment score range between 0-52 with 0 corresponding to the highest level of fatigue and 52 corresponding to the lowest level of fatigue. The scores for the 11 items where a higher score indicates more fatigue are reversed (4-score) before being summed together with the other 2 items to give the total. If any items are missed, then the total score is adjusted by multiplying by 13 and dividing by the number of items answered.

The FACIT-Fatigue will be self-administered by the participant at Baseline, Week 4, Week 12, Week 16 and Week 20.

The exploratory null hypotheses to be tested are that the mean change from baseline over time are not different for the comparisons of interest. To test these hypotheses, a MMRM will be used including the Part A visits as detailed in section 8.6.1.2

Descriptive summaries by treatment group and visit will also be displayed for Part A and Part B.

8.6.4.13 Short Form-36 Health Survey Version 2

Other secondary endpoints include:

- Short-Form-36 Health Survey Questionnaire-Version 2 (SF-36v2) Mental Component Summary (MCS);
- SF-36v2 Physical Component Summary (PCS);

The SF36v2 is a 36-item generic HRQoL instrument that uses a recall period of 4 weeks.

Each item on the survey is answered by subjects on scales of 1 to 2, 1 to 3, 1 to 5 or 1 to 6. Some of the answers are recoded that that across all questions, a higher score indicates a better health state.

Questions 2, 3A-3J, 4A-4D, 5A-5C, 9B, 9C, 9F, 9G, 9I, 10, 11A and 11C are scored as recorded. The other questions are transformed as shown in the table below:

Question	Original Code and re-code response

DocUUID : 3213b5be-8d63-4760-bea6-7e3ec175e71e

1 Original	1	2	3	4	5	
1 Recoded	5	4.4	3.4	2	1	
6, 11B, 11D Original	1	2	3	4	5	
6, 11B, 11D Recoded	5	4	3	2	1	
7 Original	1	2	3	4	5	6
7 Recoded	6	5.4	4.2	3.1	2.2	1
8 (if 7 is answered) original	1	1	2	3	4	5
Original Response to 7	1	2-6	1-6	1-6	1-6	1-6
Recoded Response	6	5	4	3	2	1
8 (if 7 is not answered) original	1	2	3	4	5	
8 Recoded	6	4.75	3.5	2.25	1	
9A, 9D, 9E, 9H Original	1	2	3	4	5	
9A, 9D, 9E, 9H Recoded	5	4	3	2	1	

The raw score of each of the 8 scales is calculated by summing the re-coded scores for its relevant questions. If <50% of questions within a scale are answered then the raw score is not calculated. If more than 50% of questions are answered but <100% then take the recoded values and sum them. Then the average score is calculated for the non-missing scores and the average scored is used for the value of the missing questions.

Physical Functioning: 3A to 3J

Role limitations due to physical health (RP): 4A to 4D

Bodily pain: 7, 8

General Health: 1, 11A to 11D

Vitality: 9A, 9E, 9G, 9I

Social Functioning: 6, 10

Role limitation due to emotional problems (RE): 5A to 5C

Mental health: 9B, 9C, 9D, 9F, 9H.

Once the raw score is calculated, then it is converted to a normalized score (on a scale of 0 to 100) using the following transformation:

(raw score – lowest possible raw score) / possible raw score range x 100.

The normalized scale score is then standardized using the following formula:

(Normalized score – A) / B.

The lowest possible raw score, possible raw score range, A and B for each scale are shown in the table below:

DocUUID : 3213b5be-8d63-4760-bea6-7e3ec175e71e

Scale	Lowest Possible Raw Score	Possible Raw Score Range	A	B
Physical Functioning	10	20	83.29094	23.75883
Limitation due to Physical health (RP)	4	16	82.50964	25.52028
Bodily Pain	2	10	71.32527	23.66224
General health	5	20	70.84570	20.97821
Vitality	4	16	58.31411	20.01923
Social Functioning	2	8	84.30250	22.91921
Limitation due to emotional problems (RE)	3	12	87.39733	21.43778
Mental health	5	20	74.98685	17.75604

The physical and mental component raw scores are then calculated based on multiplying each standardized scale score by a constant, and then adding the 8 scales together. The constants to use for the two component scores are shown below:

Score	Physical health component	Mental health Component
Physical Functioning	0.42402	-0.22999
Limitation due to Physical health (RP)	0.35119	-0.12329
Bodily Pain	0.31754	-0.09731
General health	0.24954	-0.01571
Vitality	0.02877	0.23534
Social Functioning	-0.00753	0.26876
Limitation due to emotional problems (RE)	-0.19206	0.43407
Mental health	-0.22069	0.48581

The physical and mental component norm based scores is calculated by multiplying the component raw scores by 100 and adding 50.

The change from baseline in the mental component summary (MCS) and physical component summary (PCS) will be analyzed using the method described in 8.6.1.2. Descriptive summaries by treatment group and visit will also be displayed for Part A and Part B.

DocUUID : 3213b5be-8d63-4760-bea6-7e3ec175e71e

[redacted]

8.6.4.15 ACR and PASI Composite Endpoints

4 composite endpoints will be created combining ACR and PASI endpoints:

- ACR50 and PASI90
- ACR50 and PASI100
- ACR70 and PASI90
- ACR70 and PASI100.

A subject is considered a responder for the composite endpoints at a particular timepoint if they are a responder for both of the relevant endpoints at that same timepoint. If either is missing or non-responder then the subject will be considered a non-responder.

To test these hypotheses, a pairwise stratified logistic regression will be used as detailed in Section 8.6.1.1. Descriptive summaries by treatment group and visit will also be displayed for Part A and Part B combined for NRI/LOCF.

8.7 Pharmacokinetic and Immunological Analyses

For PK and IG Analyses, no confirmatory statistical testing will be performed, but statistical tests may be used for exploratory proposes only. IG and PK analysis sets will be used.

DocUUID : 3213b5be-8d63-4760-bea6-7e3ec175e71e

8.7.1 Immunological Analyses

Antibodies to sonelokimab will be evaluated in serum samples collected for Arms 1, 2, & 3 (120 mg Q2W, 60 mg Q2W and 60 mg Q4W sonelokimab) at Baseline, Week 2, Week 4, Week 8, Week 12, Week 14, Week 16, Week 20, Week 24 and the safety follow-up visit. At dosing visits, the samples will be collected prior to dose administration.

Up to three separate ADA types may be assessed: ADAs that bind to sonelokimab ('ADA'); ADA to a modified version of sonelokimab ('mADA') and ADA that neutralise the activity of sonelokimab ('NAb ADA'). Results for mADA will only be available for ADA baseline positive participants. NAb ADA results will only be available for the ADA positive samples.

Summary tables for each ADA type will be prepared to record where applicable the number and percentage of participants who are:

- ADA positive at baseline;
- ADA positive at any visit post-baseline;
- ADA positive at baseline and any visit post-baseline;
- ADA positive post-baseline but not at baseline;
- Treatment-emergent ADA defined as either:
    - ADA positive post-baseline but not at baseline, or
    - Treatment boosted: > 4-fold increase in ADA titers at any post-baseline timepoint compared to baseline, if ADA positive at baseline. [10^(post-baseline log10 titer – baseline log10 titer) > 4]
- persistent positive (positive at more than one post-baseline visit where the first and last ADA-positive visits are separated by a period of 12 weeks or longer; or positive at last post-baseline assessment and not at baseline; or positive at a visit with less than 12 weeks before an ADA-negative last visit);
- transient positive (positive at only 1 post-baseline visit and not at baseline; or positive at two or more visits where the first and last ADA-positive visits are separated by a period less than 16 weeks and the last visit is negative).

The log10 titer values for the ADA and mADA (not NAb ADA) will be summarised by visit and treatment arm.

Additional analyses may be performed at the sponsor's discretion but they are beyond the scope of this SAP and will be specified in a separate document, if needed.

8.7.2 Pharmacokinetic Analyses

Blood samples will be collected for the measurement of trough serum sonelokimab levels at Baseline, Week 2, Week 4, Week 8, Week 12, Week 14, Week 16, Week 20 and Week 24. At dosing visits the samples will be collected prior to dose administration.

Serum concentrations of sonelokimab will be summarized by visit including number of observations, number of concentrations below the quantifiable limit (nBLQ), mean, SD, GM, CV, median, min and max. All concentration values reported as no results (not collected or ND) will be treated as missing. For the calculation of concentration summaries, all concentrations below the quantifiable limit (BLQ) will be set to the BLQ value/2.

DocUUID : 3213b5be-8d63-4760-bea6-7e3ec175e71e

The PK measurements for sonelokimab will be listed by-participant, by visit and by treatment group. Individual values of concentrations and PK parameters will be presented with 3 significant digits.

Two plots of the PK concentrations will be presented for Part A:

1. A plot of the individual PK concentration profiles with study week on the x-axis and concentration on the y-axis, with log10 spacing on the y-axis. One page will be used for each Sonelokimab arm, with the individual concentration profiles overlaid for each dose.
2. A single plot of the geometric mean concentration profiles for each Sonelokimab arm with study week on the x-axis and concentration on the y-axis, with log10 spacing on the y-axis.

Additional analyses may be performed at the sponsor's discretion, but they are beyond the scope of this SAP and will be specified in a separate document, if needed.

[redacted]

8.9 Safety Analyses

For all the safety endpoints, no formal statistical testing will be performed. Safety summaries will be presented by treatment group separately for the Part A (SAF), Part B (SAF-Part B), and Part A+B (SAF).

For Part A, 6 groups will be considered:

- Sonelokimab 120 mg (Q2W),
- Sonelokimab 60 mg (Q2W),
- Sonelokimab 60 mg (Q4W),
- Placebo,
- Adalimumab 40 mg (Q2W),
- Total sonelokimab.

For Part B and follow-up period, 10 groups will be considered:

- Sonelokimab 120 mg Q2W→sonelokimab 120 mg Q4W;
- Sonelokimab 120 mg Q2W→adalimumab 40 mg Q2W;
- Sonelokimab 60 mg Q2W→sonelokimab 60 mg Q4W;
- Sonelokimab 60 mg Q2W→sonelokimab 120 mg Q4W;
- Sonelokimab 60 mg Q4W→sonelokimab 60 mg Q4W;
- Sonelokimab 60 mg Q4W→sonelokimab 120 mg Q4W;
- Placebo→sonelokimab 120 mg Q4W;

DocUUID : 3213b5be-8d63-4760-bea6-7e3ec175e71e

- Adalimumab 40 mg Q2W→adalimumab 40 mg Q2W;
- Adalimumab 40 mg Q2W→sonelokimab 120 mg Q2W.
- Total sonelokimab

For Part A+B, 5 groups will be considered:

- Placebo (Part A only)
- Total Sonelokimab 120mg
- Total Sonelokimab 60mg
- Total Sonelokimab
- Total Adalimumab 40mg Q2W

The placebo group will include all subjects who were randomized to placebo in Part A and will only include Part A summary visits.

The total Sonelokimab 120mg group will include all subjects who were randomized to Sonelokimab 120mg Q2W in Part A and subjects who had Sonelokimab 120mg Q2W or Q4W in Part B. Subjects will only be included for summary visits during the Part they were assigned 120mg Q2W.

The total Sonelokimab 60mg group will include all subjects who were randomized to Sonelokimab 60mg Q2W or Q4W in Part A and subjects who had Sonelokimab 60mg during part B (Sonelokimab 60mg Q2W responders and Soenlokimab 60mg Q4W responders). Subjects will only be included for summary visits during the Part they were assigned Sonelokimab 60mg.

The total Adalimumab 40mg Q2W group will include all subjects who were randomized to Adalimumab 40mg Q2W during Part A and subjects who had Adalimumab 40mg Q2W during part B (Adalimumab 40mg Q2W responders and Sonelokimab 120mg Q2W non-responders).

The total Sonelokimab arm will include subjects who received sonelokimab at any time in the study. Subjects will only be included for summary visits during the Part they were assigned Sonelokimab.

8.9.1 Adverse Events

All AEs will be classified by Primary SOC and PT according to MedDRA Version 25.0 or higher and will be graded according to the National Cancer Institute Common Terminology Criteria for Adverse Events (NCI CTCAE) version 5.0.

AEs will be summarized by SOC and PT using counts and percentage (i.e., number and percentage of participants with an AE). AEs will be sorted by decreasing frequency within each SOC and PT as per the overall column.

Where a participant has the same AE, based on PT reported multiple times, the participant will only be counted once at that PT level. Where a participant has multiple AEs within the same SOC, the participant will only be counted once at that SOC level.

DocUUID : 3213b5be-8d63-4760-bea6-7e3ec175e71e

If a participant experiences the same AE more than once with different severity grades, then the event with the highest severity will be tabulated by maximum severity. In addition, AEs with a missing severity will be presented in the summary table under the category of 'Missing' and will not be imputed.

TEAEs are defined as any AE occurring or worsening on or after the first dose of study treatment.

TEAEs will be assigned to Part A, Part B and Part A+B using the event start date and time and will be defined as:

- Part A: events started on or after the first dose of study medication for Part A but not after Part B or events present prior to the first dose of study medication for Part A but increased in severity on or after dosing on Part A but not after Part B
- Part B: events started on or after the first dose of study medication for Part B or events present prior to the first dose of study medication for Part B but increased in severity on or after dosing on Part B

For Part A+B treatment emergent AEs relating to the Part when the subject was on the corresponding treatment are considered as TEAE.

Adverse events with an onset on Day 1 which are based upon pre-dose lab tests will be considered as not treatment emergent. These are defined as:

- Adverse events which begin on study day 1 with SOC = "Investigations" or PT in ("Hypercholesterolaemia" "Hyperlipidaemia" "Dyslipidaemia", "Type V hyperlipidaemia").
- The latest lab test taken on Day 1 has a time which is before the treatment start time.

Treatment-related AEs are those events with relationship to study drug 'Related' as recorded on the CRF. If the relationship to study drug is missing, the AE will be considered as treatment related.

[redacted]

Details for imputing missing or partial start dates of AEs are described in Section 8.3.2.

Separate summary tables by treatment will be presented with the total number of events and number and percentage of participants with:

- All TEAEs
- Grade 3/4/5 TEAEs
- TEAEs by maximum intensity
- Related TEAEs
- TEAE leading to treatment discontinuation

- TEAE leading to study discontinuation
- Serious adverse events (SAEs)
- Related SAEs
- Fatal AEs
- AESI.

AEs by DSUR safety topics (Hypersensitivity reactions SMQ; Opportunistic Infections SMQ; Gastrointestinal nonspecific inflammation and dysfunctional conditions SMQ).Where a participant has the same AE, based on PT reported multiple times, the participant will only be counted once at that PT level. Where a participant has multiple AEs within the same SMQ, the participant will only be counted once at that SMQ level. AEs by DSUR safety topics will only be presented for DSUR analysis.

An overall summary presenting the categories above will also be prepared by treatment group for Part A, Part B, and Part A+B. In addition, the total number of participant treatment years and exposure adjusted incidence rates [EAIR] will be provided as described in Appendix J.

8.9.2 Clinical Laboratory Evaluations

All central laboratory (hematology, biochemistry, urinalysis) parameters will be collected as described in Appendix I.

Observed and change from baseline values for clinical laboratory parameters will be summarized using descriptive statistics by treatment group and visit separately for Part A and Part B. Shift from baseline to each scheduled assessment and the worst post treatment value according to normal ranges criteria will be presented. Shift from baseline to each scheduled assessment and the worst post treatment value according to CTCAEv5.0 criteria will also be presented. CTCAEv5.0 criteria are described in Appendix L. Participants meeting any grade 3 criteria will also be listed.

In addition, the number and percentage of participants meeting the criteria for normal ranges in laboratory parameters will be summarized by treatment group separately for Part A and Part B.

Laboratory results together with the normal ranges will be listed, flagging values that are below or above the normal range. Serum/urine pregnancy tests assessments will be listed only.

Boxplots of the observed and change from baseline values for all clinical laboratory parameters will be presented by treatment group and visit for Part A and Part B. Reference lines for the normal ranges, that consistent across all participants being presented, will be displayed.

For participants with an AE of grade 3 or higher the hematology and chemistry lab parameters for that participant will be presented in a panel plot with a panel for each lab parameter. The x-axis will be the study week and the y-axis will be the absolute lab value.

8.9.3 Vital Signs

Vital sign parameters collected are weight (kg), systolic and diastolic blood pressure (SBP and DBP) (mmHg), heart rate (beats/min), respiratory rate (breaths/min) and temperature (°C).

DocUUID : 3213b5be-8d63-4760-bea6-7e3ec175e71e

Observed values and the corresponding change from baseline values will be summarized using descriptive statistics by treatment group and visit separately for Part A and Part B.

In addition, the number and percentage of participants meeting criteria for potentially clinically significant abnormalities in vital signs (SBP, DBP and heart rate) and body weight will be summarized by treatment group separately for Part A and Part B. The following table presents the Criteria for clinically relevant abnormalities.

Table: Criteria for Clinically Relevant Abnormalities in Vital Sign Parameters

**Parameter**	**Criteria**
SBP (mmHg)	Value ≤90 or decrease ≥20 mmHg from baseline<br>Value ≥180 or increase ≥20 mmHg from baseline
DBP (mmHg)	Value ≤50 or decrease ≥15 mmHg<br>Value ≥105 or increase ≥15 mmHg
Heart Rate (beats/min)	Value ≤50 bpm or decrease from baseline ≥20 bpm<br>Value ≥120 bpm or increase from baseline≥20 bpm
Weight (kg)	≥7% decrease from baseline<br>≥7% increase from baseline

8.9.4 Physical Examinations

The following parameters and body systems will be examined and any abnormalities described general appearance, skin (presence of rash), HEENT (head, ears, eyes, nose, throat), lungs (auscultation), heart (auscultation for presence of murmurs, gallops, rubs), lower extremity exam, abdomen (palpation and auscultation), neurologic (mental status, station, gait, reflexes, motor and sensory function, coordination) and lymph nodes.

All physical examination findings will be listed only.

8.9.5 Electrocardiograms

The standard 12-lead ECG parameters collected are: intervals (RR, PR, QRS, QT and QT intervals corrected for heart rate using Bazett's and Fridericia's formulas [QTcB and QTcF, respectively]).

Observed values and the corresponding change from baseline values will be summarized using descriptive statistics at each scheduled assessment by treatment group separately for Part A and Part B.

The overall ECG interpretation will be summarized by presenting the number and percentage of participants with 'Normal' and 'Abnormal' by visit and treatment group for Part A and Part B. Shift tables from baseline (Normal/Abnormal) to each visit and to the worst post-baseline ECG interpretation will be summarized.

DocUUID : 3213b5be-8d63-4760-bea6-7e3ec175e71e

ECG data will be listed by participants at each visit and time point collected with indicated clinically significant results.

In addition, the number and percentage of participants meeting the criteria for potentially clinically significant abnormalities in ECG parameters will be summarized by treatment group separately for Part A and Part B. The following table presents the criteria for clinically relevant abnormalities.

Table: Criteria for Clinically Relevant Abnormalities in ECG Parameters

**Parameter**	**Criteria**
PR Interval	>200 ms<br>>200 ms and increase from baseline ≥25%<br>> 220 ms<br>>220 ms and increase from baseline ≥25%<br>> 240 ms<br>> 240 ms and increase from baseline ≥25%
QRS Interval	>110 ms<br>>110 msec and increase from baseline ≥25%<br>>120 ms >120 ms and increase from baseline ≥25%
QT Interval	>500 ms
QTcB and QTcF	**Absolute Values**<br>>450 to ≤480 msec<br>>480 to ≤500 msec<br>>500 msec<br>**Change from baseline:**<br>Change from baseline >30 to ≤60 msec<br>Change from baseline >60 msec

[redacted]

DocUUID : 3213b5be-8d63-4760-bea6-7e3ec175e71e

[redacted]

8.10 Subgroup Analysis

Subgroup analyses for the primary endpoint and key secondary endpoints may be considered for the following baseline variables:

- Sex (male, female);
- Weight (< 100kg, ≥ 100kg);
- Prior exposure to biologic agents (yes/no);
- Concomitant DMARD use (yes/no)

These subgroup analyses will be carried out using the participants from FAS for Part A only.

DocUUID : 3213b5be-8d63-4760-bea6-7e3ec175e71e

Categorical variables will be analysed using a logistic regression model as per Section 8.6.1.1 but with the addition of the subgroup and subgroup by treatment interaction as covariates.

A forest plot will be used to display the results of the subgroup analyses.

If an individual subgroup for a variable includes fewer than 5% of the total planned study size, the subgroup analyses for that variable may be omitted.

Additional subgroup analyses may be performed at the sponsor's discretion. These additional analyses are beyond the scope of this SAP and will be specified in a separate document, if needed.

Subgroup analysis will be done pairwise to match with the main analysis. A logistic regression model including fixed effects for treatment and stratification factors sex and exposure to biologic agents prior to the screening visit is used for the odds ratio and 95% CI. The adjusted risk difference and 95% confidence interval will be found in the same way as for the main analysis, calculated for each combination of treatments within each subgroup category.

8.11 Interim Analysis

Not applicable.

8.12 Primary Analysis Timing

The primary analysis and unblinding of the data will be performed when all data up to and including Week 12 has been collected and database lock declared.

The study will be continued regardless of the results of the primary analysis.

8.13 Data Safety Monitoring Board

As required, a select number of safety outputs will be generated for the review of the DSMB. For full details of the scope of the DSMB, please see the DSMB charter. In addition a summary of the observed ACR50 responders will be presented for FAS.

8.14 Development Safety Update Report

As required, the below DSUR summaries will be generated (until unblinding only a blinded column will be presented):

- Number of participants randomized;
- Number of participants exposed by age and sex;
- Number of participants exposed by race;
- Number of SAEs within the DSUR reporting period;
- Number of SAEs;
- Listing of SAEs.

DocUUID : 3213b5be-8d63-4760-bea6-7e3ec175e71e

9 CHANGES TO PLANNED ANALYSIS FROM STUDY PROTOCOL

An additional sensitivity analysis targeting treatment policy where response is analyzed regardless of intercurrent events will also be performed on the primary endpoint.

The sensitivity estimand will also include subjects who discontinued due to adverse events.

Logistic regression estimator approach for population-level difference of proportions will be used instead of logistic regression with multiple covariates.

[redacted]

Composite endpoints for ACR+PASI are added.

LOCF/NRI analysis is added for responder parameters and LOCF for continuous.

BASDAI derivation is re-calculated according to SAP.

DocUUID : 3213b5be-8d63-4760-bea6-7e3ec175e71e

10 REFERENCES

1. ICH Topic E3: Structure and Content of Clinical Study Reports (CPMP/ICH/137/95- adopted December 1995).

2. ICH Topic E9: Statistical Principles for Clinical Trials (CPMP/ICH/363/96 – adopted March 1998).

3. Taylor W, Gladman D, Helliwell P, et al. Classification criteria for psoriatic arthritis: development of new criteria from a large international study. Arthritis Rheum 2006;54(8):2665-73.

4. Ge, Durham, Meyer, Xie, Thomas. Covariate-adjusted Difference in Proportions From Clinical Trials Using Logistic Regression and Weighted Risk Differences, DIG, 2011 45:481

DocUUID : 3213b5be-8d63-4760-bea6-7e3ec175e71e

11 APPENDICES

Appendix A: **HAQ-DI**

HEALTH ASSESSMENT QUESTIONNAIRE

Name______________________________________ **Date**____________________________________

In this section we are interested in learning how your illness affects your ability to function in daily life. Pleas

feel free to add any comments on the back of this page.

Please check the response which best describes your usual abilities OVER THE PAST WEEK:

	Without ANY Difficulty	With SOME Difficulty	With MUCH Difficulty	UNABLE To Do
**DRESSING & GROOMING**				
Are you able to:				
- Dress yourself, including tying shoelaces and doing buttons?	______	______	______	______
- Shampoo your hair?	______	______	______	______
**ARISING**				
Are you able to:				
- Stand up from a straight chair?	______	______	______	______
- Get in and out of bed?	______	______	______	______
**EATING**				
Are you able to:				
- Cut your meat?	______	______	______	______
- Lift a full cup or glass to your mouth?	______	______	______	______
- Open a new milk carton?	______	______	______	______
**WALKING**				
Are you able to:				
- Walk outdoors on flat ground?	______	______	______	______
- Climb up five steps?	______	______	______	______

Please check any AIDS OR DEVICES that you usually use for any of these activities:

DocUUID : 3213b5be-8d63-4760-bea6-7e3ec175e71e

______ Cane

______ Walker

______ Crutches

______ Wheelchair

______ Devices used for dressing (button hook, zipper pull, long-handled shoe horn, etc.)

______ Built up or special utensils

______ Special or built up chair

______ Other (Specify:________________________)

Please check any categories for which you usually need HELP FROM ANOTHER PERSON:

______ Dressing and Grooming

______ Arising

______ Eating

______ Walking

DocUUID : 3213b5be-8d63-4760-bea6-7e3ec175e71e

Please check the response which best describes your usual abilities OVER THE PAST WEEK:

	Without ANY Difficulty	With SOME Difficulty	With MUCH Difficulty	UNABLE To Do
**HYGIENE**				
Are you able to:				
- Wash and dry your body?	_____	_____	_____	_____
- Take a tub bath?	_____	_____	_____	_____
- Get on and off the toilet?	_____	_____	_____	_____
**REACH**				
Are you able to:				
- Reach and get down a 5 pound object (such as a bag of sugar) from just above your head?	_____	_____	_____	_____
- Bend down to pick up clothing from the floor?	_____	_____	_____	_____
**GRIP**				
Are you able to:				
- Open car doors?	_____	_____	_____	_____
- Open jars which have been previously opened?	_____	_____	_____	_____
- Turn faucets on and off?	_____	_____	_____	_____
**ACTIVITIES**				
Are you able to:				
- Run errands and shop?	_____	_____	_____	_____
- Get in and out of a car?	_____	_____	_____	_____
- Do chores such as vacuuming or yardwork?	_____	_____	_____	_____

Please check any AIDS OR DEVICES that you usually use for any of these activities:

_____ Raised toilet seat	_____ Bathtub bar
_____ Bathtub seat	_____ Long-handled appliances for reach
_____ Jar opener (for jars previously opened)	_____ Long-handled appliances in bathroom
	_____ Other (Specify: _______________________)

DocUUID : 3213b5be-8d63-4760-bea6-7e3ec175e71e

Please check any categories for which you usually need HELP FROM ANOTHER PERSON:

_____ Hygiene _____ Gripping and opening things

_____ Reach _____ Errands and chores

DocUUID : 3213b5be-8d63-4760-bea6-7e3ec175e71e

Appendix B: Patients Assessment of Arthritis Pain (PtAAP)

How much pain have you had because of your arthritis TODAY:

PLACE A VERTICAL (I) MARK ON THE LINE TO INDICATE THE SEVERITY OF THE PAIN.

NO PAIN 0	________________________________	MOST SEVERE PAIN 100

DocUUID : 3213b5be-8d63-4760-bea6-7e3ec175e71e

Appendix C**: Patients Global; Assessment of Disease Activity (PtAAP)**

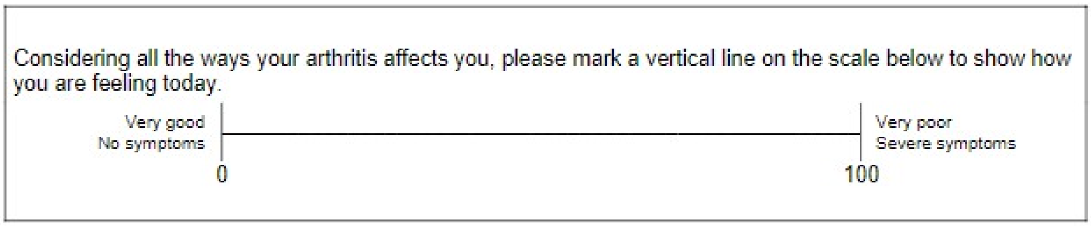

Considering all the ways your arthritis affects you, please mark a vertical line on the scale below to show how you are feeling today.

Very good
No symptoms

Very poor
Severe symptoms

0

100

DocUUID : 3213b5be-8d63-4760-bea6-7e3ec175e71e

Appendix D: Physician's Global; Assessment of Disease Activity (PtAAP)

Please indicate your assessment of the patient's overall disease activity by marking a vertical line through the line below.

Very good, asymptomatic and no limitation of normal activities	0 ———————————————— 100	Very poor, very severe symptoms which are intolerable and inability to carry out all normal activities

DocUUID : 3213b5be-8d63-4760-bea6-7e3ec175e71e

Appendix E: Leeds Enthesitis Index (LEI)

LEEDS ENTHESITIS INDEX (LEI)

1.	Date of LEI<br>[Date of assessment]	**[Date of assessment]**<br>**[Date of assessment] (DD/MM/YYYY)**<br>☐ Done<br>Date of LEI<br>__/__/____<br>☐ Not Done
2.	Lateral epicondyle - Left<br>[Lateral epicondyle - Left]	**[Lateral epicondyle - Left]**<br>☐ Present<br>☐ Absent<br>☐ Not Done<br>☐ Not Applicable
3.	Medial femoral condyle - Left<br>[Medial femoral condyle - Left]	**[Medial femoral condyle - Left]**<br>☐ Present<br>☐ Absent<br>☐ Not Done<br>☐ Not Applicable
4.	Achilles tendon insertion - Left<br>[Achilles tendon insertion - Left]	**[Achilles tendon insertion - Left]**<br>☐ Present<br>☐ Absent<br>☐ Not Done<br>☐ Not Applicable
5.	Lateral epicondyle - Right<br>[Lateral epicondyle - Right]	**[Lateral epicondyle - Right]**<br>☐ Present<br>☐ Absent<br>☐ Not Done<br>☐ Not Applicable
6.	Medial femoral condyle - Right<br>[Medial femoral condyle - Right]	**[Medial femoral condyle - Right]**<br>☐ Present<br>☐ Absent<br>☐ Not Done<br>☐ Not Applicable
7.	Achilles tendon insertion - Right	**[Achilles tendon insertion - Right]**<br>☐ Present<br>☐ Absent<br>☐ Not Done<br>☐ Not Applicable
8.	**LEI score:** ______	**LEI score:** ______

DocUUID : 3213b5be-8d63-4760-bea6-7e3ec175e71e

Appendix F: Psoriatic Assessment Impact of Disease-12

We want you to indicate how much your psoriatic arthritis impacts your health. Please tell us how you have been feeling this last week.

Pain

Circle the number that best describes the pain you felt due to your psoriatic arthritis during the last week:

None	0	1	2	3	4	5	6	7	8	9	10	Extreme

For Office use only

Result x3

☐

Fatigue

Circle the number that best describes the overall level of fatigue due to your psoriatic arthritis you have experienced during the last week:

No fatigue	0	1	2	3	4	5	6	7	8	9	10	Totally exhausted

Result x2

☐

Skin problems

Circle the number that best describes the skin problems including itching you felt due to your psoriatic arthritis du[illegible] week:

Result x2

☐

DocUUID : 3213b5be-8d63-4760-bea6-7e3ec175e71e

None	0	1	2	3	4	5	6	7	8	9	10	Extreme

Work and/or leisure activities

Circle the number that best describes the difficulties you had to participate fully in work and/or leisure activities due to your psoriatic arthritis during the last week:

None	0	1	2	3	4	5	6	7	8	9	10	Extreme

Result x2 ☐

Functional capacity

Circle the number that best describes the difficulty you had in doing daily physical activities due to your psoriatic [illegible]ng the last week:

Result x2 ☐

No difficulty	0	1	2	3	4	5	6	7	8	9	10	Extreme difficulty

Discomfort

Circle the number that best describes the feeling of discomfort and annoyance with everyday tasks due to your psoriatic arthritis during the last week:

Result x2 ☐

None	0	1	2	3	4	5	6	7	8	9	10	Extreme

DocUUID : 3213b5be-8d63-4760-bea6-7e3ec175e71e

Sleep disturbance

Circle the number that best describes the sleep difficulties (i.e., resting at night) you felt due to your psoriatic arthr[illegible] last week:

No difficulty	0	1	2	3	4	5	6	7	8	9	10	Extreme difficulty

Result x2 ☐

Coping

Considering your psoriatic arthritis overall, how well did you cope (manage, deal, make do) with your psoriatic a[illegible] the last week?

Very well	0	1	2	3	4	5	6	7	8	9	10	Very poorly

For Office use only

Result x1 ☐

Anxiety, fear and uncertainty

Circle the number that best describes the level of anxiety, fear and uncertainty (for example about the future, treatments, fear of loneliness) due to your psoriatic arthritis you have experienced during the last week:

None	0	1	2	3	4	5	6	7	8	9	10	Extreme

Result x1 ☐

Embarrassment and/or shame

Considering your psoriatic arthritis overall, circle the number that best describes the level of embarrassment and/or shame due to your appearance experienced during the last week:

Result x1 ☐

DocUUID : 3213b5be-8d63-4760-bea6-7e3ec175e71e

None	0	1	2	3	4	5	6	7	8	9	10	Extreme

Social participation

Circle the number that best describes the difficulties you had to participate fully in social activities (including relationships with family and/or people very close to you) due to your psoriatic arthritis during the last week:

None	0	1	2	3	4	5	6	7	8	9	10	Extreme

Result x1 ☐

Depression

Circle the number that best describes the level of depression due to your psoriatic arthritis you have experienced during the last week:

None	0	1	2	3	4	5	6	7	8	9	10	Extreme

Result x1 ☐

THANK YOU FOR ANSWERING THIS QUESTIONNAIRE

Final PsAID out of 20

Add up the ☐ and divide by 20:

DocUUID : 3213b5be-8d63-4760-bea6-7e3ec175e71e

Appendix G**: Leeds Diactylitis Score**

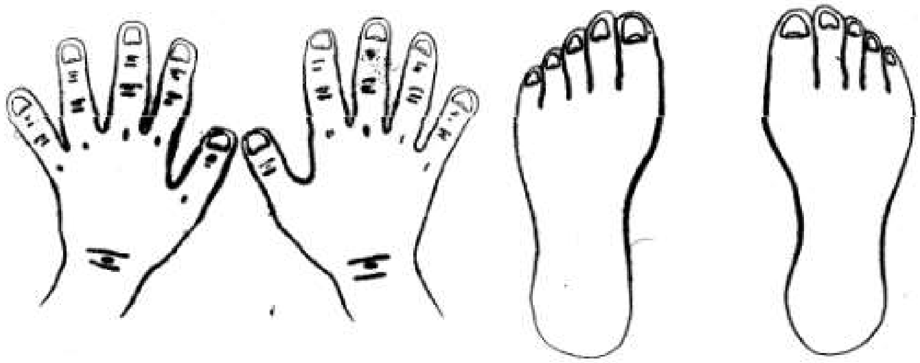

Finger / Toe	Circumference Involved Digit (A)	Contralateral Digit (or Tables) (B)	Tenderness Score (C)	Final Score: [{(A/B)-1}x100]xC
TOTAL	0	0	0	0

Standard reference: Table - hands

Digit	Men	Women
Thumb	70	58
Index	63	54
Middle	63	54
Ring	59	50
Little	52	44

Table – feet

Digit	Men	Women
Great toe	82	72
Second	52	46
Middle	50	44
Fourth	50	44
Little	52	45

Tenderness score: response to squeeze
0 no tenderness
1 tender
2 tender and wince
3 tender and withdraw

DocUUID : 3213b5be-8d63-4760-bea6-7e3ec175e71e

Appendix H: **FACIT-Fatigue Scale**

Below is a list of statements that other people with your illness have said are important. **Please select one number per line to indicate your response as it applies to the <u>past 7 days</u>.**

		Not at all	A little bit	Some-what	Quite a bit	Very much
HI7	I feel fatigued	0	1	2	3	4
HI12	I feel weak all over	0	1	2	3	4
An1	I feel listless ("washed out")	0	1	2	3	4
An2	I feel tired	0	1	2	3	4
An3	I have trouble starting things because I am tired	0	1	2	3	4

DocUUID : 3213b5be-8d63-4760-bea6-7e3ec175e71e

An4	I have trouble finishing things because I am tired	0	1	2	3	4
An5	I have energy	0	1	2	3	4
An7	I am able to do my usual activities	0	1	2	3	4
An8	I need to sleep during the day	0	1	2	3	4
An12	I am too tired to eat	0	1	2	3	4
An14	I need help doing my usual activities	0	1	2	3	4
An15	I am frustrated by being too tired to do the things I want to do	0	1	2	3	4
An16	I have to limit my social activity because I am tired	0	1	2	3	4

DocUUID : 3213b5be-8d63-4760-bea6-7e3ec175e71e

[redacted]

DocUUID : 3213b5be-8d63-4760-bea6-7e3ec175e71e

[redacted]

DocUUID : 3213b5be-8d63-4760-bea6-7e3ec175e71e

Appendix J: **Clinical Laboratory Tests**

**Laboratory Testing Profile**	**Tests Included**
Laboratory tests required prior to first study treatment dose only	QuantiFERON TB-gold test (or equivalent IGRA), HIV testing, hepatitis B serology (HBV PCR if required) including hepatitis B surface antigen and antibody and hepatitis B core antibody, hepatitis C serology, HCV antibodies, confirmatory HCV PCR, SARS-CoV-2 PCR, RF, CCP antibodies, FSH, serum pregnancy
Blood Chemistry	Sodium, potassium, chloride, bicarbonate, total protein, blood urea nitrogen, serum creatinine, albumin, alkaline phosphatase, AST, ALT, total bilirubin, direct bilirubin, indirect bilirubin, calcium, GGT, creatine kinase, hs-CRP
Hematology	Hemoglobin, hematocrit, WBC with differentials (monocytes, eosinophils, basophils, neutrophils, lymphocytes) as an absolute value, RBC count, platelet count, ESR
Urinalysis	<u>Local urine testing</u><br>Routine urinalysis will be performed locally with urine dipstick test (semi-quantitative "dipstick" evaluation of specific gravity, pH, glucose, protein, bilirubin, ketones, leukocytes, blood).<br>If the dipstick urine test indicates any abnormal findings, the urine sample will be sent to the central laboratory for further assessments, as appropriate (reflex testing).<br><u>Central lab urine testing (reflex testing)</u><br>Urine chemistry (albumin:creatinine and protein:creatinine ratios) to be performed if dipstick indicates protein;<br>Microscopy and/or culture to be performed if clinically indicated or if urinalysis results positive (blood, protein or leukocyte esterase/WBC)

Urinalysis	Note: Urine hCG pregnancy testing for WOCBP; may be repeated more frequently than indicated if required by local practice, if a menstrual cycle is missed, or if potential pregnancy is suspected
Fasting Lipid Panel and Glucose to be performed at Day 1/Week 0 and EOT	Fasting: glucose, total cholesterol, low density lipoprotein, high density lipoprotein, triglycerides,

Abbreviations: ALT: Alanine aminotransferase, AST: Aspartate aminotransferase, CCP = cyclic citrullinated peptide; EOT: End of Treatment; ESR: erythrocyte sedimentation rate; FSH: Follicle stimulating hormone; GGT: gamma-glutamyl transferase, HBV: hepatitis B virus; HCV: hepatitis C virus; HIV: Human immunodeficiency virus; hs-CRP: high-sensitivity C-reactive protein, IGRA = interferon-gamma release assay; PCR: Polymerase chain reaction; RBC: red blood cells; RF = rheumatoid factor; TB = tuberculosis; SARSCoV2: Severe Acute Respiratory Syndrome Coronavirus 2; WBC: white blood cells; WOCBP = women of childbearing potential

DocUUID : 3213b5be-8d63-4760-bea6-7e3ec175e71e

Appendix K: **Exposure adjusted incidence rate**

It will be assumed that for each of n participants in a clinical trial the time tj (j=1,...,n) to the first occurrence of a certain event is observed, or if the event was not experienced, the (censored) time to the end of the observation period for each treatment group. The sequence of first occurrences of an event will be modeled to follow approximately a Poisson process with constant intensity θ. The rate parameter θ will be estimated as λ=D/T, where $T = \sum_{j=1}^{n} t_j$ and D is the number of participants with at least one event. The end of the observation period for subjects randomized in Part A to sonelokimab is the last visit date in Part B or study discontinuation date. The end of the observation period for subjects randomized to placebo or Adalimumab will be the first dose of Part B study medication or study discontinuation if discontinued during Part A. The start of the observation period of sonelokimab for subjects who were randomized to placebo or Adalimumab in Part A will be the first dose of Part B study medication.

DocUUID : 3213b5be-8d63-4760-bea6-7e3ec175e71e

Appendix L**: CTCAE v5.0 laboratory grades**

Lab parameter	Units		Grade 1	Grade 2	Grade 3	Grade 4
**sodium**	mmol/L	low	130 - < LLN	125-129	120-124	<120
		high	>ULN - 150	>150 - 155	> 155-160	> 160
**potassium**	mmol/L	low	3.0 - < LLN	3.0 - < LLN	2.5 - < 3.0	< 2.5
		High	> ULN – 5.5	> 5.5 – 6.0	> 6.0 – 7.0	> 7.0
**Alanine aminotransferase**	U/L	High	>ULN – 3* ULN if baseline normal; >1.5 – 3*ULN if baseline abnormal	> 3 – 5*ULN	> 5 – 20*ULN	> 20*ULN
**Alkaline phosphatase**	U/L	High	>ULN – 2.5* ULN if baseline normal; >2.0 – 2.5*ULN if baseline abnormal	> 2.5 – 5*ULN	> 5 – 20*ULN	> 20*ULN
**Aspartate aminotransferase**	U/L	High	>ULN – 3* ULN if baseline normal; >1.5 – 3*ULN if baseline abnormal	> 3 – 5*ULN	> 5 – 20*ULN	> 20*ULN
**Total bilirubin**	umol/L	High	>ULN – 1.5* ULN	> 1.5 – 3*ULN	> 3 – 10*ULN	> 10*ULN
**Serum creatinine**	umol/L	High	> ULN – 1.5* ULN	> 1.5 – 3.0*ULN	> 3.0 – 6.0 ULN	> 6.0*ULN
**Creatine kinase**	U/L	High	> ULN – 2.5* ULN	> 2.5 – 5.0*ULN	> 5.0 – 10.0 ULN	> 10.0*ULN
**Albumin**	g/L	low	30 - < LLN	20 - <30	< 20	
**Calcium**	mmol/L	Low	2.0 - < LLN	1.75 - < 2.0	1.5 - < 1.75	< 1.5
		High	>ULN – 2.9	>2.9 – 3.1	>3.1 – 3.4	>3.4
**GGT**		High	>ULN – 2.5*ULN if baseline normal; >2.0 – 2.5*ULN if baseline abnormal	>2.5 – 5.0*ULN	>5.0-20.0*ULN	>20.0*ULN
**Hemoglobin**	g/L	low	100 - <LLN	80 - < 100	< 80	
		High	Increase >0-20	Increase >20-40	Increase >40	
**Neutrophils**	10^9/L	Low	1.5 - < LLN	1.0 - <1.5	0.5 - <1.0	<0.5
**Lymphocytes**	10^9/L	Low	0.8 - <LLN	0.5 - <0.8	0.2 - <0.5	<0.2
		High	-	>4-20	>20	-
**Platelets**	10^9/L	Low	75 - <LLN	50 - <75	25 - <50	<25

DocUUID : 3213b5be-8d63-4760-bea6-7e3ec175e71e

[redacted]

DocUUID : 3213b5be-8d63-4760-bea6-7e3ec175e71e

[redacted]

DocUUID : 3213b5be-8d63-4760-bea6-7e3ec175e71e

[redacted]

DocUUID : 3213b5be-8d63-4760-bea6-7e3ec175e71e

[redacted]

[redacted]

DocUUID : 3213b5be-8d63-4760-bea6-7e3ec175e71e
