# Supplementary material for: Sonelokimab, an IL-17A/IL-17F-inhibiting nanobody for active psoriatic arthritis: a randomized, placebo-controlled phase 2 trial
Source: Nat Med. 2025 Oct 6;31(12):4160–71. doi: 10.1038/s41591-025-03971-6 (PMC12705426; doi:10.1038/s41591-025-03971-6)
Supplement: Supplementary file 2 — Reporting Summary [file 41591_2025_3971_MOESM2_ESM.pdf]

nature portfolio

Corresponding author(s): Iain B. McInnes

Last updated by author(s): Aug 12, 2025

Reporting Summary

Nature Portfolio wishes to improve the reproducibility of the work that we publish. This form provides structure for consistency and transparency in reporting. For further information on Nature Portfolio policies, see our Editorial Policies and the Editorial Policy Checklist.

Statistics

For all statistical analyses, confirm that the following items are present in the figure legend, table legend, main text, or Methods section.

n/a	Confirmed	
☐	☒	The exact sample size (*n*) for each experimental group/condition, given as a discrete number and unit of measurement
☐	☒	A statement on whether measurements were taken from distinct samples or whether the same sample was measured repeatedly
☐	☒	The statistical test(s) used AND whether they are one- or two-sided<br>*Only common tests should be described solely by name; describe more complex techniques in the Methods section.*
☐	☒	A description of all covariates tested
☐	☒	A description of any assumptions or corrections, such as tests of normality and adjustment for multiple comparisons
☐	☒	A full description of the statistical parameters including central tendency (e.g. means) or other basic estimates (e.g. regression coefficient) AND variation (e.g. standard deviation) or associated estimates of uncertainty (e.g. confidence intervals)
☐	☒	For null hypothesis testing, the test statistic (e.g. *F*, *t*, *r*) with confidence intervals, effect sizes, degrees of freedom and *P* value noted<br>*Give P values as exact values whenever suitable.*
☒	☐	For Bayesian analysis, information on the choice of priors and Markov chain Monte Carlo settings
☒	☐	For hierarchical and complex designs, identification of the appropriate level for tests and full reporting of outcomes
☐	☒	Estimates of effect sizes (e.g. Cohen's *d*, Pearson's *r*), indicating how they were calculated

Our web collection on statistics for biologists contains articles on many of the points above.

Software and code

Policy information about availability of computer code

Data collection	N/A
Data analysis	N/A

For manuscripts utilizing custom algorithms or software that are central to the research but not yet described in published literature, software must be made available to editors and reviewers. We strongly encourage code deposition in a community repository (e.g. GitHub). See the Nature Portfolio guidelines for submitting code & software for further information.

Data

Policy information about availability of data

All manuscripts must include a data availability statement. This statement should provide the following information, where applicable:

- Accession codes, unique identifiers, or web links for publicly available datasets
- A description of any restrictions on data availability
- For clinical datasets or third party data, please ensure that the statement adheres to our policy

MoonLake Immunotherapeutics AG is committed to sharing clinical trial data for the purpose of supporting legitimate scientific research. Requests from qualified scientific researchers will be promptly reviewed by an internal committee of subject matter experts and/or an independent review panel to assess the feasibility and scientific validity of the request. Data that may be requested include non-identifiable patient- and study-level clinical trial data, clinical study reports, and protocols. All data provided will be anonymized to respect the privacy of trial participants and are subject to the

protection of patient privacy and informed consent. A data sharing agreement will need to be signed.
Data from this manuscript can be requested by qualified researchers 6 months after product approval in the USA and Europe (or after global development is discontinued) and 24 months after trial completion. Requests should be submitted to the corresponding author or to dataaccessrequests@moonlaketx.com. The trial protocol and statistical analysis plan can be found in the Supplementary Materials.

Research involving human participants, their data, or biological material

Policy information about studies with human participants or human data. See also policy information about sex, gender (identity/presentation), and sexual orientation and race, ethnicity and racism.

Reporting on sex and gender	In the manuscript, the term "sex" is used appropriately to refer to biological sex. The term "gender" is not used and is not part of any analysis. Sex was considered in the study design (randomization stratified by sex). Data have been reported according to the prespecified primary and secondary analysis of this study; post hoc analyses by sex have been conducted for key endpoints.
Reporting on race, ethnicity, or other socially relevant groupings	Outcomes are not reported by race or ethnicity.
Population characteristics	Baseline characteristics are reported in Table 1.
Recruitment	Between December 13, 2022 and May 23, 2023, 265 patients were screened at 42 clinical sites in eight countries (Bulgaria, Czechia, Estonia, Germany, Hungary, Poland, Spain, and the USA). Of the 265 patients screened, 207 were randomized into the study. Eligible patients were ≥18 years of age, with a confirmed diagnosis of PsA, active disease (defined as TJC68 ≥3 and SJC66 ≥3), and either currently active psoriasis or a dermatologist-confirmed history of psoriasis. Patients were excluded if they had prior exposure to >2 biologics of any type (e.g. IL-17, IL-23, and TNF inhibitors), previous failure of IL-17 or TNF inhibitor therapy, or a diagnosis of chronic inflammatory conditions other than psoriasis. Overall, 102 (49.3%) patients were female, 36 (17.4%) had previously received at least one bDMARD, and nine (4.3%) patients had received two prior bDMARDs.<br><br>Clinical trials enrollment may introduce bias, both through self-selection and Hawthorne effects. ARGO used a double-blind, placebo-controlled design to minimize the effect of any bias related to inclusion in a clinical study. Although results from clinical studies may not be fully generalizable due to self-selection bias, the population enrolled in ARGO was consistent with other Phase 2 PsA studies.
Ethics oversight	The study was conducted with the ethical principles of ICH/GCP, the provisions of the Declaration of Helsinki (October 2013), and FDA (Code of Federal Regulations, Sections 312.50 and 312.56), European Union (536/2014), and United Kingdom regulations (The Medicines for Human Use [Clinical Trials] Regulations 2004 [no. 1031]). All patients provided written informed consent.<br><br>The study protocol, amendments, and all recruitment materials were reviewed and approved by an Institutional Review Board (IRB) or Independent Ethics Committee (IEC) at each participating site. The IECs/IRBs were: Ethics Committee for Clinical Trials under the Minister of Health (Bulgaria); EC St. Anne's University Hospital in Brno and the Ethics Committee of the Institute for Clinical and Experimental Medicine and Thomayer University Hospital (Czech Republic); Ethics Committee for Medicinal Products (Estonia); Ethics Committee of the Medical Faculty (Germany); Ethics Committee for Clinical Pharmacology (Hungary); Bioethics Committee at the Wielkopolska Medical Chamber in Poznań (Poland); Medicinal Research Ethics Committee (CEIm of Parc Taulí; Spain); and Advarra (United States).

Note that full information on the approval of the study protocol must also be provided in the manuscript.

Field-specific reporting

Please select the one below that is the best fit for your research. If you are not sure, read the appropriate sections before making your selection.

☒ Life sciences ☐ Behavioural & social sciences ☐ Ecological, evolutionary & environmental sciences

For a reference copy of the document with all sections, see nature.com/documents/nr-reporting-summary-flat.pdf

Life sciences study design

All studies must disclose on these points even when the disclosure is negative.

Sample size	Sample size was calculated based on the assumed ACR50 response rates of 40% with each sonelokimab dose regimen and 10% with placebo, at Week 12. Based on these assumptions, a sample size of 40 patients in each of the sonelokimab arms and in the placebo arm resulted in a power of more than 80%, using an overall two-sided alpha of 0.025.
Data exclusions	None
Replication	Phase 3 studies of sonelokimab in PsA are ongoing to confirm the clinical findings of this Phase 2 study: the IZAR-1 study (NCT06641076) in patients with biologic-naive PsA, and the IZAR-2 study (NCT06641089) in patients with PsA and prior inadequate response or intolerance to biologic TNF inhibitors.
Randomization	Patients were randomized 1:1:1:1:1 to sonelokimab 120mg with induction, sonelokimab 60mg with induction, sonelokimab 60mg no

Randomization	induction, placebo, or adalimumab 40mg Q2W (active reference arm). Randomization was stratified by sex (male/female) and prior exposure to biologic agents (yes/no). Web-based interactive response technology was used to assign patients to treatment arms following a predetermined computer-generated randomization scheme (randomization block size was 10) that was approved by the sponsor biostatistician.
Blinding	To maintain study blinding, the prefilled syringes for sonelokimab and placebo were identical in appearance. The adalimumab injector was different in appearance from sonelokimab and placebo; however, the study was blinded at the carton level. Patients were also asked to wear an eye mask for all injections. All study personnel were blinded until Week 12.

Reporting for specific materials, systems and methods

We require information from authors about some types of materials, experimental systems and methods used in many studies. Here, indicate whether each material, system or method listed is relevant to your study. If you are not sure if a list item applies to your research, read the appropriate section before selecting a response.

Materials & experimental systems

n/a	Involved in the study
☒	☐ Antibodies
☒	☐ Eukaryotic cell lines
☒	☐ Palaeontology and archaeology
☒	☐ Animals and other organisms
☐	☒ Clinical data
☒	☐ Dual use research of concern
☒	☐ Plants

Methods

n/a	Involved in the study
☒	☐ ChIP-seq
☒	☐ Flow cytometry
☒	☐ MRI-based neuroimaging

Clinical data

Policy information about clinical studies

All manuscripts should comply with the ICMJE guidelines for publication of clinical research and a completed CONSORT checklist must be included with all submissions.

Clinical trial registration	NCT05640245
Study protocol	The study protocol has been submitted as a supplemental file along with the manuscript. Proprietary information has been redacted in accordance with journal guidelines.
Data collection	The trial was conducted at 42 clinical sites in Bulgaria, Czechia, Estonia, Germany, Hungary, Poland, Spain, and the USA. A total of 265 patients were screened between December 13, 2022 and May 23, 2023.
Outcomes	The primary endpoint was the proportion of patients achieving an ACR50 response at Week 12 and the key secondary endpoints were: the proportion of patients achieving ACR20 response at Week 12 compared with placebo and the proportion of patients achieving PASI 90 response at Week 12 compared with placebo in the subgroup of patients with psoriasis involving ≥3% BSA at baseline. ACR20/50 response was defined as ≥20/50% improvement in TJC68 and in SJC66, and ≥20/50% improvement in ≥3/5 additional variables (Patient's Global Assessment of Disease Activity, Physician's Global Assessment of Disease Activity, Patient's Assessment of Arthritis Pain, Health Assessment Questionnaire Disability Index, and high-sensitivity C-reactive protein and was measured using the standard ACR response criteria. The standard PASI score was used to assess the degree of a patient's psoriasis.

Plants

Seed stocks	*Report on the source of all seed stocks or other plant material used. If applicable, state the seed stock centre and catalogue number. If plant specimens were collected from the field, describe the collection location, date and sampling procedures.*
Novel plant genotypes	*Describe the methods by which all novel plant genotypes were produced. This includes those generated by transgenic approaches, gene editing, chemical/radiation-based mutagenesis and hybridization. For transgenic lines, describe the transformation method, the number of independent lines analyzed and the generation upon which experiments were performed. For gene-edited lines, describe the editor used, the endogenous sequence targeted for editing, the targeting guide RNA sequence (if applicable) and how the editor was applied.*
Authentication	*Describe any authentication procedures for each seed stock used or novel genotype generated. Describe any experiments used to assess the effect of a mutation and, where applicable, how potential secondary effects (e.g. second site T-DNA insertions, mosiacism, off-target gene editing) were examined.*
